# Anxiety disorder burden inequalities across middle- and high-income countries: 1990–2035 trends and projections

**DOI:** 10.1371/journal.pone.0352932

**Published:** 2026-07-21

**Authors:** Yuxuan He, Lijie Liu, Wenfu Song, Yuxi Li, Yimeng Gao, Wenwen Zhu, Runru Mai, Fei Tan, Fuping Xu, Zhimin Yang

**Affiliations:** 1 The Second Clinical College of Guangzhou University of Chinese Medicine, Guangzhou, China; 2 Chinese Medicine Guangdong Laboratory (Hengqin Laboratory), Zhuhai, China; 3 State Key Laboratory of Traditional Chinese Medicine Syndrome, The Second Clinical School, Guangzhou University of Chinese Medicine, Guangzhou, Guangdong, China; 4 Hospital of Chengdu University of Traditional Chinese Medicine, Chengdu, China; 5 The Second Affiliated Hospital of Guangzhou University of Chinese Medicine, Guangdong Provincial Hospital of Chinese Medicine, Guangzhou, China; Lorestan University, IRAN, ISLAMIC REPUBLIC OF

## Abstract

**Background:**

Anxiety disorders are among the most common mental health conditions worldwide, but their burden varies substantially across countries with different income levels. Evidence remains limited on how anxiety disorder burden, violence-related risk factors, and future trends differ across middle- and high-income countries (MHICs). This study examined inequalities in the burden of anxiety disorders across MHICs from 1990 to 2023, quantified the burden attributable to major risk factors, and projected prevalence trends to 2035.

**Methods:**

Data on incidence, prevalence, and disability-adjusted life years (DALYs) for anxiety disorders were obtained from the Global Burden of Disease 2023 database. We estimated counts, age-standardized incidence rates (ASIRs), age-standardized prevalence rates (ASPRs), and age-standardized DALY rates (ASDRs), with 95% uncertainty intervals (UIs), by income group, sex, age, country, and year. Risk-attributable DALYs were assessed for intimate partner violence (IPV), bullying victimization (BV), and sexual violence against children (SVAC). Temporal trends were evaluated using Joinpoint regression, and Bayesian age-period-cohort (BAPC) models were used to project prevalence rates to 2035.

**Results:**

In 2023, MHICs accounted for 91.3% (95% UI 60.7–137.2) of global prevalent cases, 90.8% (95% UI 50.4–153.7) of incident cases, and 91.2% (95% UI 44.8–177.6) of anxiety disorder DALYs. High-income countries (HICs) had the highest ASIRs, ASPRs, and ASDRs, whereas lower-middle-income countries (LMICs) had lower rates but larger absolute numbers of cases. From 1990 to 2023, the burden increased across MHICs, with the fastest growth observed in LMICs. Females had consistently higher ASIRs, ASPRs, and ASDRs than males, and the highest burden was observed among children and adolescents. IPV, BV, and SVAC were associated with substantial anxiety disorder DALYs, with marked differences by income group, age, and sex. BAPC projections suggested that prevalence rates may continue to increase through 2035, particularly among females and in lower-income settings.

**Conclusions:**

Anxiety disorder burden remains high and unequally distributed across MHICs. The burden is characterized by persistent differences by income group, sex, and age, and is associated with violence-related risk factors. These findings support the need for income-sensitive, gender-responsive, and age-targeted mental health strategies, with particular attention to prevention of violence-related risks and improved access to mental health services in lower-income settings.

## 1. Introduction

Anxiety disorders are among the most prevalent mental health conditions globally and are characterized by persistent or recurrent anxiety accompanied by autonomic dysfunction, motor disturbances, and other related symptoms [1]. Their high prevalence, chronic course, and frequent comorbidities make anxiety disorders an important contributor to the global burden of disease (GBD). In 2021, anxiety disorders ranked as the sixth largest cause of years lived with disability (YLDs) worldwide and were among the top 25 fastest-growing causes of disability-adjusted life years (DALYs). An estimated 35.9 million people were affected worldwide [2], and this number is expected to reach 87.36 million by 2050 [3]. These trends suggest that anxiety disorders may place increasing pressure on global public health and economic systems. In 2019, global spending on anxiety treatment reached USD 204 billion, representing one of the highest expenditures among mental disorders [4]. Anxiety disorders frequently coexist with other psychiatric conditions, particularly major depressive disorder. In the GBD 2019 assessment, depression and anxiety were among the leading contributors to the mental disorder burden [5].

Although many studies have described the global burden of anxiety disorders, most research has focused on global patterns or on low- and middle-income countries, whereas relatively limited attention has been paid to middle- and high-income countries (MHICs) [6,7]. Previous studies have reported higher prevalence and burden estimates of anxiety disorders in economically developed regions, suggesting substantial heterogeneity across countries with different socioeconomic conditions. These findings suggest that MHICs may exhibit distinct epidemiological and risk-related characteristics that require targeted analysis [8,9].The World Bank income classification demonstrates clear differences in anxiety burden across countries with different economic levels [10]. In low-income countries, limited healthcare access and underdiagnosis remain major barriers, whereas in MHICs, the burden of anxiety disorders has been associated with social and economic pressure, competitive living environments, urbanization, and widening income inequality [11]. Because of these differences, it is important to assess the burden of anxiety disorder in MHICs to support targeted mental health planning and resource allocation.

It is also important to understand how risk-related factors are associated with the burden of anxiety disorders. Sex- and age-related differences are well recognized, but relatively few studies have examined how modifiable social and behavioral factors are associated with these disparities [12,13]. In MHICs, healthcare resources are generally more available, although access and utilization remain uneven across populations. However, studies evaluating anxiety disorders in relation to modifiable risk factors remain limited. This evidence gap may hinder the development of targeted prevention strategies and contribute to mismatches between mental health resources and population needs. To address this gap, this study examined the burden of anxiety disorders in MHICs from 1990 to 2023 and produced projections through 2035. The goal was to provide references that can guide future policy decisions.

## 2. Methods

The GBD study provides a standardized and comparable framework for quantifying health loss associated with diseases, injuries, and risk factors across time, geography, and population subgroups. Estimates are generated by location, age, sex, and year and are regularly updated to incorporate newly available data and methodological improvements. The GBD 2023 study assessed 375 diseases and 88 risk factors across 204 countries and territories from 1990 to 2023, stratified by age and sex. This study utilized publicly available secondary data from GBD 2023. We followed the Guidelines for Accurate and Transparent Health Estimates Reporting (GATHER). As no direct patient involvement or primary data collection was undertaken, ethical approval was not required. Detailed analytical approaches, including the methods for quantifying uncertainty, are described in previous GBD methodological publications. The analysis included all individuals with anxiety disorders between 1990 and 2023. Data on incidence, prevalence, and DALYs, together with their 95% uncertainty intervals (UIs), were obtained from the GBD 2023 Results Tool (https://ghdx.healthdata.org/gbd-2023).

### 2.1. Country classification and disease definition

All estimates were stratified by age, region, and country. The study focused on 174 MHICs, defined according to the World Bank Fiscal Year 2025 income classification, which is based on gross national income (GNI) per capita in 2023 (Supplementary Table 2 in S1 file).

GNI reflects the total economic output of a country, calculated as gross domestic product (GDP) plus income earned by residents from foreign investments, minus income earned by foreign residents within the domestic economy. According to the World Bank 2023 classification, income levels were defined as follows: LMIC, with a GNI per capita ranging from $1146 to $4515; UMIC, with a GNI per capita ranging from $4516 to $14 005; and HIC, with a GNI per capita of $14 005 or more [14].The International Classification of Diseases codes used to define anxiety disorders are detailed in Supplementary Table 3 in S1 file [15].

### 2.2. Estimation of incidence, prevalence, and DALYs

Age-standardized incidence rates (ASIRs), prevalence rates (ASPRs), and DALY rates (ASDR) were extracted from GBD 2023, stratified by age, sex, country, and year (1990–2023). All rates were expressed per 100,000 population and standardized using the GBD 2023 reference population to ensure cross-national and temporal comparability.

Incidence was defined as the number of newly diagnosed anxiety disorder cases in a given year, whereas prevalence referred to the total number of existing cases within a defined time period (typically one year). DALYs quantified the total years of healthy life lost due to both premature mortality and disability, with disability weights derived from population-based surveys and expert assessments within the GBD framework [16].

### 2.3. Uncertainty and trend analysis

All estimates were presented with 95% UIs, accounting for variability in input data, model parameters, and sampling processes. Temporal trends in age-standardized rates from 1990 to 2023 were evaluated using Joinpoint regression, which computes the average annual percent change (AAPC) as the weighted mean of annual percent changes across identified segments. The AAPC summarizes overall temporal patterns and was estimated from the slope of a linear regression model fitted to the natural logarithm of age-standardized rates. A trend was considered statistically significant if the 95% confidence interval (CI) of the AAPC excluded zero [16,17].

### 2.4. Attributable burden analysis

The estimation of risk factor–attributable burden followed the comparative risk assessment framework established in the GBD study [16]. Three major risk factors were identified as contributors to anxiety disorder-related DALYs**:** intimate partner violence**,** sexual violence against children, and bullying victimization. For each risk-outcome pair, population attributable fractions (PAFs) were calculated using the equation:


$$\begin{array}{c} PAF=\frac{\sum_{i=\text{1}}^{k}P_{i}(RR_{i}-\text{1})}{\sum_{i=\text{1}}^{k}{(P}_{i}RR_{i})} \end{array}$$

Where Pi denotes the population proportion at exposure level i, RRi represents the relative risk at that exposure relative to the reference level, and k is the number of exposure categories. Relative risks were derived from meta-analyses of prospective cohort and randomized controlled trials within GBD 2023, with appropriate adjustments for confounding risk factors. PAFs were then applied to the total anxiety disorder DALYs to estimate attributable DALYs. Uncertainty in exposure prevalence, relative risks, and theoretical minimum risk exposure levels was propagated through 1,000 Monte Carlo simulations to generate 95% UIs. Analyses were stratified by age group, country, and year.

### 2.5. Statistical analysis

Temporal trends in age-standardized rates (ASRs) from 1990 to 2023 were analyzed using Joinpoint regression software (version 5.4.0, National Cancer Institute, USA, https://surveillance.cancer.gov/joinpoint/). Log-linear models (ln(rate) = β × year + ε) were fitted to estimate annual percent changes (APCs) and their 95% CIs, and AAPCs were calculated to summarize overall changes. A minimum of one joinpoint and a maximum of four joinpoints were allowed. The final model was selected based on Monte Carlo permutation tests, model parsimony, and trend interpretability. Increasing trends were defined when both the AAPC estimate and its 95% CI were greater than zero, and decreasing trends when both were below zero.

To further examine temporal variations, a Bayesian age-period-cohort (BAPC) model based on integrated nested Laplace approximation (INLA) was applied for projection analysis. This Bayesian framework enables detection of subtle generational shifts or hidden temporal trends that may not be captured by traditional AAPC analysis. By integrating AAPC and BAPC results, this study provides a comprehensive characterization of how anxiety disorder patterns evolve under changing health, social, and environmental contexts [18]. Sensitivity analyses were conducted using alternative BAPC specifications and a no-drift scenario to assess the robustness of projected trends.

All visualizations and statistical analyses were conducted using R software (version 4.5.1). The R scripts used for data processing, Joinpoint analysis, and BAPC projection are publicly available at Zenodo (https://doi.org/10.5281/zenodo.20551146).

### 2.6. Role of the funding source

The funding agencies had no involvement in the design, data collection, analysis, interpretation, or writing of this study.

## 3. Results

### 3.1. The current burden of anxiety disorders in 2023

In 2023, anxiety disorders remained a major mental health concern across MHICs (Table 1). MHICs accounted for 91.3% (95% UI 60.7–137.2) of global prevalent cases, 90.8% (95% UI 50.4–153.7) of incident cases, and 91.2% (95% UI 44.8–177.6) of anxiety disorder DALYs.

**Table 1:** Incidence, prevalence, and DALY counts of anxiety disorders in 2023 across MHICs.

Locations	Sex	Incidence number (95% UI)	Prevalence number (95% UI)	DALYs number (95% UI)
World Bank High Income	Male	4079338.6 (3162797.3 to 5299926.1)	35030201.4 (27197772.0 to 43021569.6)	4115925.7 (2718983.7 to 5825139.8)
	Female	6865720.0 (5332987.1 to 8916633.9)	61790585.7 (49623520.6 to 73853698.1)	7126866.2 (4822998.1 to 10044959.9)
	Both	10945058.6 (8492968.2 to 14231416.8)	96820787.1 (76815024.7 to 115991331.7)	11242792.0 (7541981.8 to 15834076.5)
World Bank Upper Middle Income	Male	7861936.1 (5,744,556.4 to 11,171,970.8)	60811792.4 (45788029.2 to 78302607.9)	7231338.1 (4736362.0 to 10805087.0)
	Female	11694798.1 (8,688,044.7 to 16,707,109.4)	96814128.2 (74267002.5 to 122599568.6)	11319907.6 (7549769.6 to 16704930.4)
	Both	19556734.2 (14432797.4 to 27833226.0)	157625920.7 (120055031.7 to 201214567.9)	18551245.6 (12286131.6 to 27825231.2)
World Bank Lower Middle Income	Male	7790936.9 (5569401.8 to 11121093.2)	67626622.8 (52075787.4 to 90232928.2)	8117682.5 (5117282.5 to 12195432.4)
	Female	12127058.8 (8732303.3 to 17412806.5)	106712073.9 (83921589.2 to 138970211.6)	12558173.3 (8180353.2 to 18690802.7)
	Both	19917995.7 (14269732.5 to 28638335.8)	174338696.7 (135997376.7 to 229390833.9)	20675855.8 (13314428.4 to 30619199.1)

**Fig 1:**
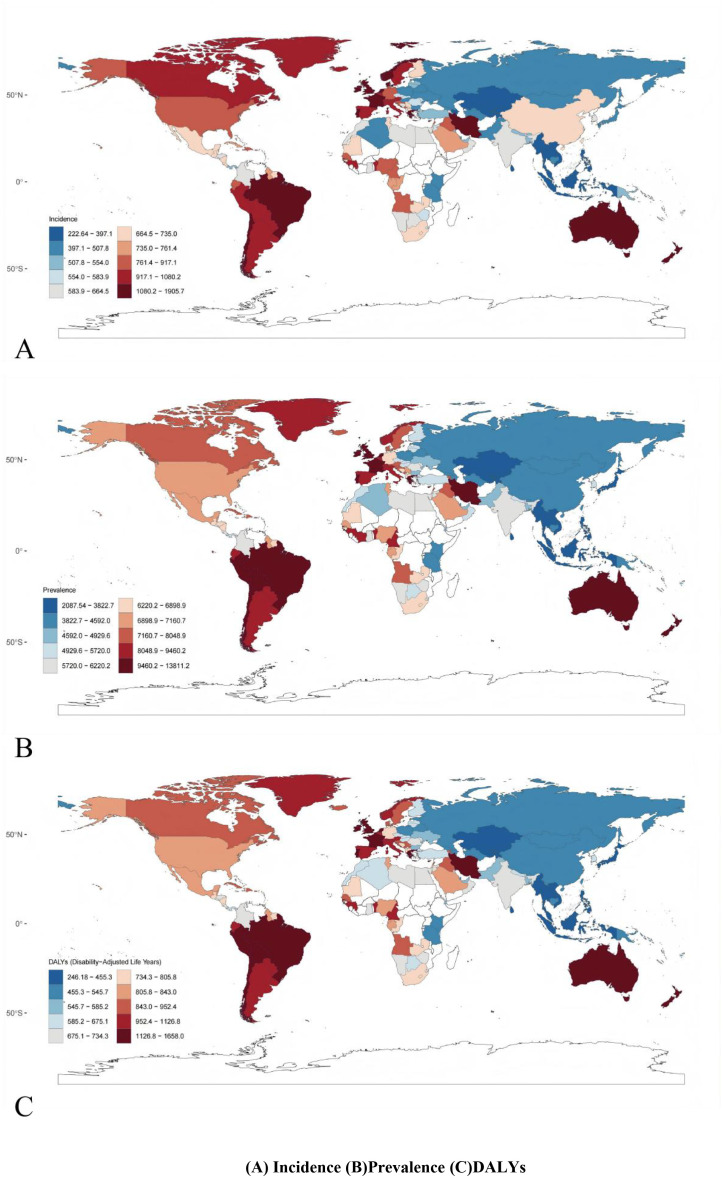
Global distribution of anxiety disorder incidence, prevalence, and DALY rates in 2023. The base map was generated using Natural Earth public domain data through the natural earth package in R. No copyrighted map or satellite imagery was used. (A) Incidence (B) Prevalence (C) DALYs.

Substantial regional heterogeneity was observed across income groups (Fig 1). LMICs exhibited lower ASIR, ASPR, and ASDR (Table 2), but had larger absolute numbers of cases because of their large populations. Within LMICs, India showed the highest burden, with 9.73 million incident cases (95% UI 7.25–13.36 million), 86.0 million prevalent cases (68.5–109.9 million), and 10.12 million DALYs cases (6.66–14.71 million). By contrast, HICs exhibited the highest ASIR, ASPR, and ASDR but smaller absolute numbers of cases. Malta, Portugal, and Australia showed the highest standardized rates among HICs. In Malta, the ASIR, ASPR, and ASDR were 1,905.7 (1,531.3–2,395.3), 13,811.2 (12,021.1–15,889.1), and 1,658.0 (1,164.8–2,319.9) per 100,000, respectively. In Portugal, the corresponding rates were 1,722.9 (1,132.2–2,520.8), 13,711.4 (10,176.1–17,733.1), and 1,635.6 (1,042.2–2,400.1) per 100,000. Among UMICs, Brazil had the highest standardized rates, whereas Mauritius had the lowest values of 222.6 (172.6–290.9), 2,087.5 (1,650.6–2,501.2), and 246.2 (164.5–356.7) per 100,000 respectively.

**Table 2:** Incidence, prevalence, and DALY rates of anxiety disorders in 2023 across MHICs.

Locations	Sex	Incidence rate per 100,000 (95% UI)	Prevalence rate per 100,000 (95% UI)	DALYs rate per 100,000 (95% UI)
World Bank High Income	Male	617.1（449.0 to 814.4）	5,008.6 (3,753.2 to 6,515.7)	598.3 (386.1 to 884.7)
	Female	1,059.0 (751.8 to 1,404.9)	8,710.7 (6,689.0 to 10,915.0)	1,026.6 (668.2 to 1,486.8)
	Both	834.0 (598.4 to 1,102.2)	6,833.5 (5,197.8 to 8,686.8)	809.1 (523.8 to 1,180.0)
World Bank Upper Middle Income	Male	555.2 (390.3 to 821.8)	4,161.1 (3,148.2 to 5,572.0)	498.4 (318.4 to 751.5)
	Female	834.5 (592.46 to 1,182.33)	6,621.1 (5,116.8 to 8,426.6)	782.4 (505.1 to 1,160.2)
	Both	692.3 (489.9 to 992.0)	5,374.5 (4,118.2 to 6,969.6)	638.3 (410.9 to 944.6)
World Bank Lower Middle Income	Male	490.6 (355.1 to 682.6)	4,283.5 (3,275.0 to 5,605.4)	510.6 (330.9 to 753.9)
	Female	776.2 (566.5 to 1,099.4)	6,850.3 (5,350.8 to 8,822.7)	801.7 (525.6 to 1,176.5)
	Both	632.2 (460.1 to 891.2)	5,562.3 (4,308.7 to 7,208.0)	655.4 (425.7 to 963.4)

**Fig 2:**
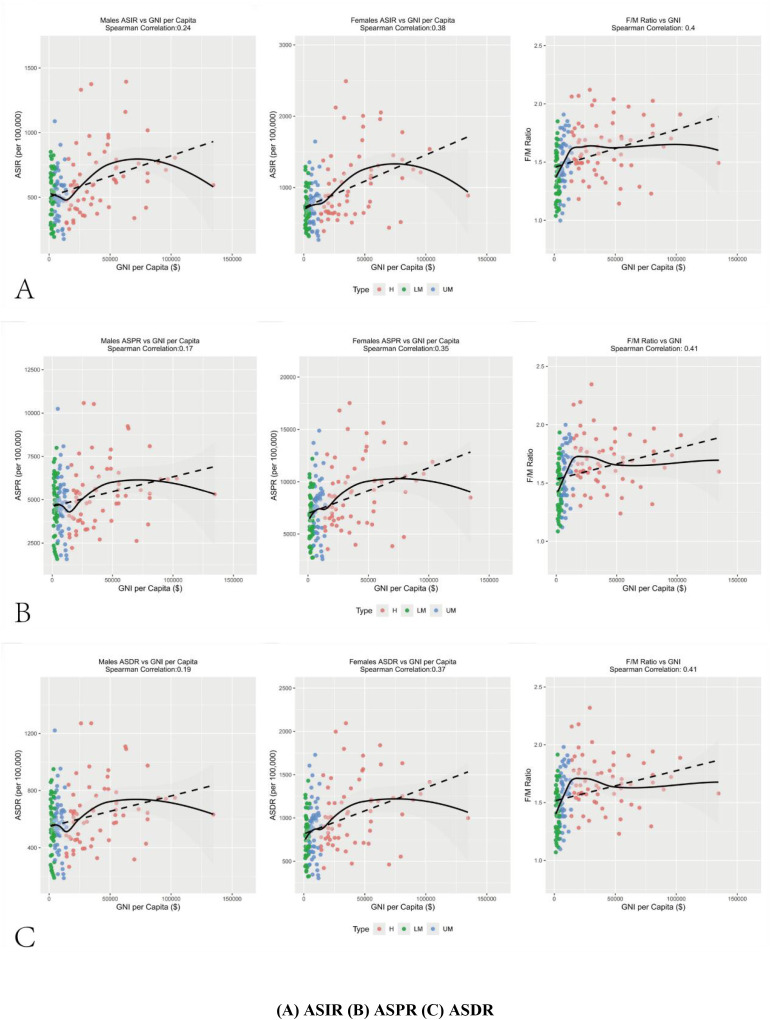
Association between national income level (GNI per capita) and sex differences in the burden of anxiety disorders across income groups. (A) ASIR (B) ASPR (C) ASDR.

Pronounced sex disparities were observed across MHICs (Supplementary Table 3 in S1 file). Females consistently exhibited higher ASIRs, ASPRs, and ASDRs than males. Spearman’s correlation analysis (Fig 2) showed positive association between the female-to-male (F/M) burden gap and GNI. Female anxiety burden increased with higher GNI, showing moderate correlations ASIR (r = 0.38), ASPR (r = 0.35), and ASDR (r = 0.37) (all p < 0.01), whereas weaker correlations were observed among males (r = 0.24, 0.17, 0.19, respectively). The F/M ratios of ASIRs, ASPRs, and ASDRs also increased significantly with rising GNI (r ≈ 0.40–0.41, p < 0.001), suggesting widening sex disparities in anxiety burden with increasing economic development.

**Fig 3:**
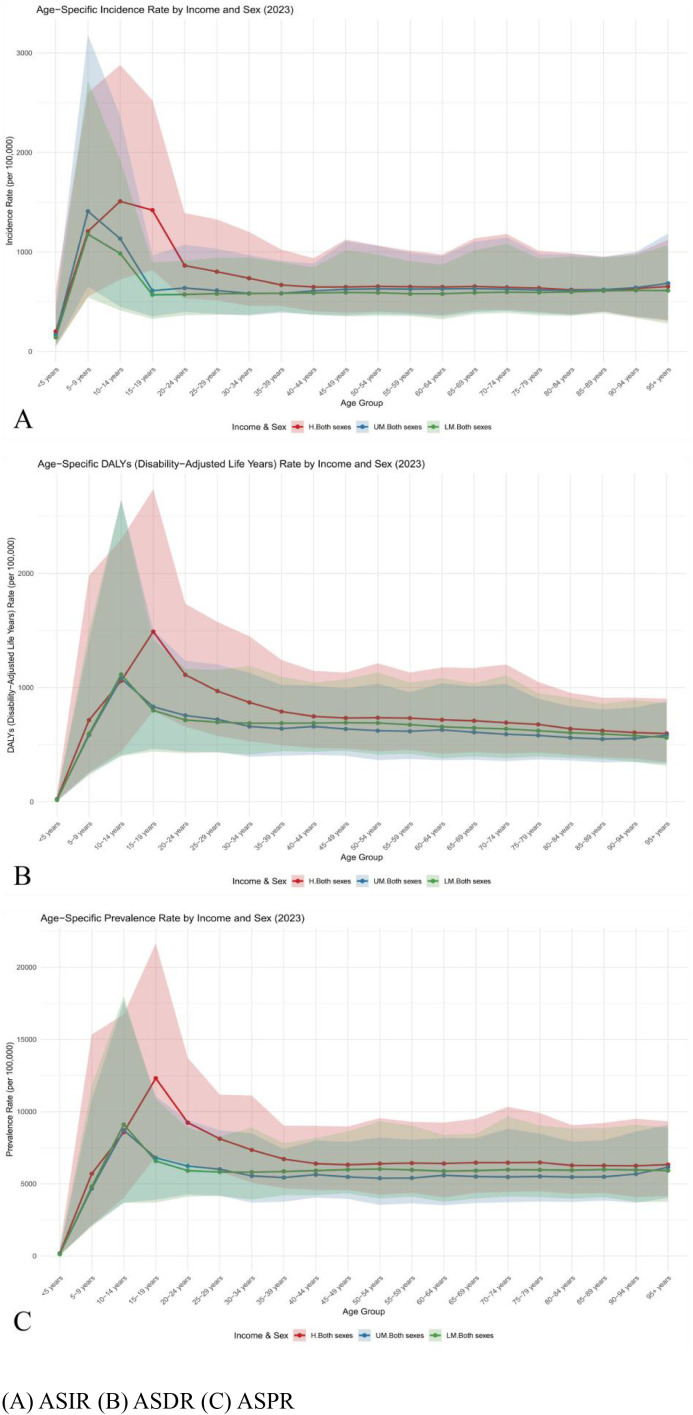
Age-specific incidence, DALY, and prevalence rates of anxiety disorders by income group and sex in 2023. (A) ASIR (B) ASDR (C) ASPR.

Age-specific analysis (Fig 3) indicated that anxiety disorders predominantly affected adolescents and children. In HICs, the highest prevalence burden was observed among individuals aged 15–19 years, whereas the peak prevalence occurred among those aged 10–14 years in UMICs and LMICs. The highest incidence and DALY rates were observed among individuals aged 10–14 years in HICs and 5–9 years in lower-income regions.

**Fig 4:**
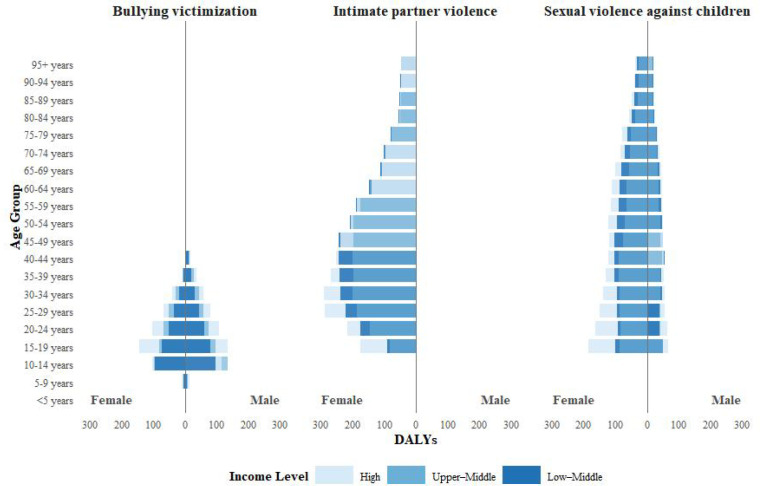
Age- and sex-specific DALY rates attributable to key risk factors for anxiety disorders across income groups in 2023.

In 2023, intimate partner violence (IPV), bullying victimization (BV), and sexual violence against children (SVAC) were associated with substantial anxiety disorder DALYs across MHICs. Among these factors, IPV showed the strongest association, particularly among females aged 25–49 years, whereas BV was more strongly associated with DALYs among adolescents and young adults (Fig 4).

### 3.2. Temporal trends of anxiety disorders from 1990 to 2023

Between 1990 and 2023, the burden of anxiety disorders increased substantially across MHICs. ASPRs and ASDRs increased steadily across all income groups, with an accelerated rise observed during 2019–2023 (Table 3). ASIRs also increased from 1990 to 2019, followed by a marked rise between 2019 and 2021 and a slight decline during 2021–2023.

**Table 3:** AAPC of anxiety disorders from 1990 to 2023 across MHICs.

Locations	Sex	ASIR AAPC (95%CI)	ASPR AAPC (95%CI)	ASDR AAPC (95%CI)
World Bank High Income	Both	0.6 (0.0 to 1.0)	1.3 (1.2 to 1.4)	1.3(1.2 to 1.4)
World Bank High Income	Female	0.6 (0.0 to 1.0)	1.3 (1.1 to 1.4)	1.3(1.2 to 1.4)
World Bank High Income	Male	0.6 (0.0 to 1.0)	1.5 (1.4 to 1.6)	1.6 (1.5 to 1.6)
World Bank Upper Middle Income	Both	0.6 (0.0 to 1.2)	1.3 （1.2 to 1.4）	1.3 (1.2 to 1.4)
World Bank Upper Middle Income	Female	0.7(−0.1 to 1.2)	1.4 (1.2 to 1.5)	1.3 (1.2 to 1.4)
World Bank Upper Middle Income	Male	0.7 (0.0 to 1.2)	1.3 (1.2 to 1.4)	1.3 (1.2 to 1.4)
World Bank Lower Middle Income	Both	1.3 (0.4 to 1.8)	2.1 (2.0 to 2.3)	2.1 (2.0 to 2.3)
World Bank Lower Middle Income	Female	1.4 (0.5 to 2.0)	2.2 (2.0 to 2.3)	2.2 (2.0 to 2.3)
World Bank Lower Middle Income	Male	1.1 (0.2 to 1.7)	2.0 (1.8 to 2.1)	2.0 (1.8 to 2.1)

Temporal trends varied considerably across income groups. The increase was most pronounced in LMICs, where ASPRs showed an AAPC of 2.1% (95% CI 2.0–2.3) and ASDRs an AAPC of 2.1% (2.0–2.3), whereas ASIRs increased more moderately (AAPC = 1.3% [0.4–1.8]). Within LMICs, sharp increases were observed in Nigeria, Bangladesh, and Egypt, whereas Palestine showed relatively limited change(Supplementary Table 3 in S1 file). In HICs, incidence and prevalence rates increased gradually but persistently, with the largest increases observed in Australia, Denmark, and New Zealand, whereas Bahrain showed a slight decline. In UMICs, the magnitude of increase was comparable to or slightly greater than that observed in HICs, with Mexico and Turkmenistan exhibiting the most pronounced increases, whereas Algeria and China showed relatively moderate growth.

The burden associated with violence- related risk factors also increased across MHICs, particularly in LMICs. DALY rates associated with IPV increased from 32.01 (95% UI 0–81.21) to 66.11 (0–169.94) per 100,000 (AAPC = 1.7 [95% UI 1.1–2.2]). DALY rates associated with BV from 13.59 (4.45 to 29.62) to 25.76 (9.70–56.04) per 100,000 (AAPC = 2.1 [1.3–3.0]). In addition, SVAC-associated DALY rates increased from 37.99 (0–123.86) to 48.95 (0–157.20) per 100,000 (AAPC = 0.9 [0.3–1.4]). In UMICs, IPV-associated DALY rates IPV increased from 39.83 (0–96.66) to 56.45 (0–155.87) per 100,000 (AAPC = 0.6 [0.1–1.1]), whereas BV-associated DALY rates increased from 25.74 (8.91 to 54.02) to 32.71 (11.91–69.92) (AAPC = 0.7 [0.1–1.4]). Similarly, SVAC- associated DALY rates increased from 34.92 (0–118.43) to 42.16 (0–136.58) per 100,000 (AAPC = 0.5 [0–1.1]). In contrast, HICs showed relatively smaller increases, IPV -associated DALY rates increased from 59.85 (0–144.38) to 74.76 (0–191.91) per 100,000 (AAPC = 1 [0.5–1.6]), whereas BV- and SVAC-associated DALY rates remained comparatively stable, with AAPCs close to zero (BV AAPC = −0.1 [−0.6–0.3], SVAC AAPC = 0.2 [−0.3–0.6]).

### 3.3. Projected burden of anxiety disorders to 2035

**Fig 5:**
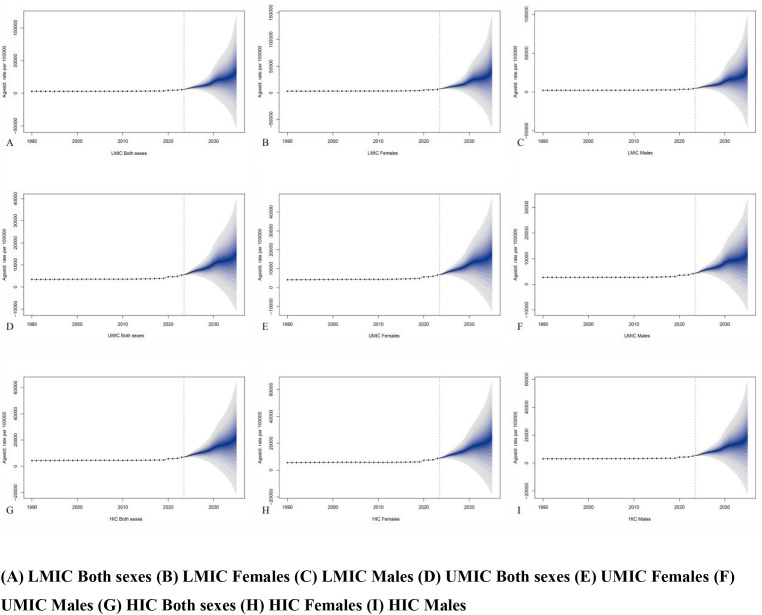
BAPC-projected prevalence rates of anxiety disorders by income group and sex through 20352035. (A) LMIC Both sexes (B) LMIC Females (C) LMIC Males (D) UMIC Both sexes (E) UMIC Females (F) UMIC Males (G) HIC Both sexes (H) HIC Females (I) HIC Males.

BAPC projections suggested a continued increase in anxiety disorder ASPRs across all income groups from 2024 to 2035 (Fig 5).

Among LMICs, the projected increase was most pronounced, with ASPRs expected to rise from approximately 5,562 (95% UI 4,309–7,208) per 100,000 in 2023–38,729 (33,195–44,263) in 2035. Females were projected to maintain substantially higher prevalence rates than males, with a more pronounced increase after 2030.

In UMICs, ASPRs were projected to increase gradually from approximately 5,374 (4,118–6,970) per 100,000 in 2023–13,870 (13,146–14,593) in 2035. Upward trends were observed in both sexes, although females consistently exhibited higher projected prevalence rates.

In HICs, ASPR s were also projected to continue increasing, increasing from approximately 6,833.5(5,198–8,687) per 100,000 in 2023–21,824 (21,808–21,840) in 2035. Although the projected increase was less pronounced than that observed in LMICs and UMICs, females continued to exhibit a substantially greater burden than males.

Sensitivity analyses using alternative BAPC specifications and a no-drift scenario yielded similar overall temporal patterns, supporting the robustness of the projected trends (Supplementary Fig. 1 in S1 file).

## 4. Discussion

In 2023, anxiety disorders continued to impose a substantial burden across MHICs, accounting for more than 90% of global incident cases, prevalent cases, and DALYs. The persistently high age-standardized incidence, prevalence, and DALY rates suggest that the psychological consequences associated with the COVID-19 pandemic may continue beyond the acute pandemic period. From 1990 to 2023, the burden of anxiety disorders increased steadily across MHICs, with a more pronounced rise observed after 2019 [19]. Although prevalence and DALYs continued to increase, incidence rates began to decline slightly after 2021. This pattern may reflect gradual improvements in public health responses, mental health awareness, and post-pandemic adaptation measures [20].

But the burden did not grow in the same way across all income regions. LMICs exhibited the most rapid increases, with particularly high burdens observed in India, Bangladesh, and Nigeria. These countries are undergoing rapid social and economic change, while simultaneously facing constraints in mental health resources and healthcare accessibility. In addition, underdiagnosis is likely related to limited healthcare access, insufficient mental health infrastructure, and variability in reporting systems, which may partly contribute to the observed increases in age-standardized rates as detection capacity improves [6,10]. In contrast, HICs showed high prevalence but relatively slower growth. More stable healthcare systems, broader mental health coverage, and earlier recognition of anxiety disorders may partly explain these patterns [21–23]. UMICs demonstrated the slowest growth, which may reflect gradual stabilization following earlier periods of rapid economic and social transition, together with continued improvement in health services [24].

Substantial disparities were also observed across sex and age groups. Females consistently exhibited higher incidence, prevalence, and DALYs than males across MHICs. Biological factors do not fully explain this. Many females experience substantial social and economic pressure, and they often take on more caregiving work [25]. These factors increase stress and raise the risk of anxiety [26,27]. Children and adolescents aged 5–24 years also represented a particularly vulnerable population. In HICs, academic pressure, social isolation, and expectations for performance play major roles [28]. In UMICs and LMICs, economic hardship, exposure to violence, and limited social support systems are more common drivers [29,30].

Violence-related risk factors are major contributors to DALYs. These include IPV, BV, and SCAV. Females in MHICs face higher levels of IPV and SVAC, especially in HICs. IPV disproportionately affects females and has been associated with persistent psychological distress and anxiety symptoms [31]. SVAC may damage an individual's sense of safety and stability and can create long-lasting fear. Previous studies suggest that childhood exposure to sexual violence may increase vulnerability to anxiety disorders later in life [32]. Attachment theory also shows the psychological mechanisms underlying this relationship. Anxious attachment and avoidant attachment both heighten vulnerability [33]. Bullying is another important factor. Boys experience bullying more often but may not report it because of shame or fear [34]. Girls report lower levels of bullying but tend to internalize stress and show more sensitivity to social pressure. Girls also face higher rates of cyberbullying [35].

BAPC projections suggest that the prevalence of anxiety disorders in MHICs may continue to rise through 2035. LMICs and females are projected to experience the fastest increases. Earlier GBD projections also suggested rising trends, but the increase observed in this study appears stronger. This may be associated with persistent post-pandemic psychological effects, changing work and educational environments, and broader social transitions [36]. These patterns suggest that anxiety disorders may remain an important public health issue in the next decades. In areas with weaker health systems and lower income, improvements in awareness, screening, and healthcare access may contribute to the identification of previously under-recognized cases.

These findings may have several references for policy and clinical practice. MHICs may need to strengthen monitoring systems and healthcare services for people across income groups [37]. They may also need to increase mental health service capacity. This includes tele-mental health programs, community-based support, and school-based screening strategies to help reduce the growing burden among adolescents can be reduced [38]. Additionally, MHICs may need to place greater emphasis on females’ mental health and services for young people. Agencies in health, education, and social protection should work together to reduce violence-related risks [39]. More investment in LMICs may also help reduce differences in anxiety burden across regions.

This study has several limitations. First, the study was based on estimates from the GBD 2023 database, which relies on heterogeneous data sources across countries and years. Some regions may still experience incomplete reporting, delayed data collection, or limited epidemiological surveillance. Second, anxiety disorder prevalence estimates partly rely on self-reported symptoms and survey-based assessments, which may introduce reporting bias and cross-country differences in symptom recognition. Third, the BAPC model is sensitive to assumptions regarding temporal trends and may be influenced by future policy changes, economic crises, healthcare reforms, or unexpected public health events. Finally, because the study period began in 1990, early estimates may have been affected by changes in diagnostic criteria, evolving mental health awareness, and improvements in healthcare systems over time.

## 5. Conclusion

Using the latest GBD 2023 estimates, our analysis indicates that anxiety disorders still place a heavy burden on MHICs in 2023. From 1990 to 2023, the burden kept rising and increased sharply after 2019. BAPC projections also suggest that this rise may continue quickly through 2035. Inequalities across income level, sex, and age remain and have become even wider. LMICs are projected to experience the fastest increase, while HICs are expected to show a more stable pattern. The burden is also projected to increase more quickly among females and adolescents. These findings may help inform policies that aim to reduce the rising burden of anxiety disorders in MHICs.

## Supporting information

S1 File Supplementary Tables. (DOCX)

## References

[1] Penninx BW, Pine DS, Holmes EA, Reif A. Anxiety disorders. Lancet. 2021;397(10277):914–27. doi: 10.1016/S0140-6736(21)00359-7 33581801 PMC9248771

[2] Global incidence, prevalence, years lived with disability (YLDs), disability-adjusted life-years (DALYs), and healthy life expectancy (HALE) for 371 diseases and injuries in 204 countries and territories and 811 subnational locations, 1990-2021: a systematic analysis for the Global Burden of Disease Study 2021. Lancet. 2024;403(10440):2133–61. 38642570 10.1016/S0140-6736(24)00757-8 PMC11122111

[3] Chen S, Huang W, Zhang M, Song Y, Zhao C, Sun H, et al. Dynamic changes and future trend predictions of the global burden of anxiety disorders: analysis of 204 countries and regions from 1990 to 2021 and the impact of the COVID-19 pandemic. EClinicalMedicine. 2024;79:103014. doi: 10.1016/j.eclinm.2024.103014 39834715 PMC11743809

[4] Mitchell AJ, Cogswell IE, Dalos J, Tsakalos G, Lei J, Oros A, et al. Estimating global direct health-care spending on neurological and mental health between 2000 and 2019: a modelling study. Lancet Public Health. 2025;10(5):e401–11. doi: 10.1016/S2468-2667(25)00089-1 40312084

[5] Global, regional, and national burden of 12 mental disorders in 204 countries and territories, 1990-2019: a systematic analysis for the Global Burden of Disease Study 2019. Lancet Psychiatry. 2022;9(2):137–50. 35026139 10.1016/S2215-0366(21)00395-3 PMC8776563

[6] Cénat JM, Dalexis RD, Guerrier M, Noorishad P-G, Derivois D, Bukaka J, et al. Frequency and correlates of anxiety symptoms during the COVID-19 pandemic in low- and middle-income countries: A multinational study. J Psychiatr Res. 2021;132:13–7. doi: 10.1016/j.jpsychires.2020.09.031 33035760 PMC7527178

[7] Nielsen-Scott M, Fellmeth G, Opondo C, Alderdice F. Prevalence of perinatal anxiety in low- and middle-income countries: A systematic review and meta-analysis. J Affect Disord. 2022;306:71–9. doi: 10.1016/j.jad.2022.03.032 35306121

[8] Bie F, Yan X, Xing J, Wang L, Xu Y, Wang G, et al. Rising global burden of anxiety disorders among adolescents and young adults: trends, risk factors, and the impact of socioeconomic disparities and COVID-19 from 1990 to 2021. Front Psychiatry. 2024;15:1489427. doi: 10.3389/fpsyt.2024.1489427 39691785 PMC11651023

[9] Wu Y, Li X, Ji X, Ren W, Zhu Y, Chen Z, et al. Trends in the epidemiology of anxiety disorders from 1990 to 2021: A global, regional, and national analysis with a focus on the sociodemographic index. J Affect Disord. 2025;373:166–74. doi: 10.1016/j.jad.2024.12.086 39732404

[10] Patel V, Saxena S, Lund C, Thornicroft G, Baingana F, Bolton P, et al. The Lancet Commission on global mental health and sustainable development. Lancet. 2018;392(10157):1553–98. doi: 10.1016/S0140-6736(18)31612-X 30314863

[11] Ruscio AM, Hallion LS, Lim CCW, Aguilar-Gaxiola S, Al-Hamzawi A, Alonso J, et al. Cross-sectional Comparison of the Epidemiology of DSM-5 Generalized Anxiety Disorder Across the Globe. JAMA Psychiatry. 2017;74(5):465–75. doi: 10.1001/jamapsychiatry.2017.0056 28297020 PMC5594751

[12] Zhang Z, Chen X, Wu S, Chen X, Wang X, Liu C, et al. Global, regional and national burden of anxiety and depression disorders from 1990 to 2021, and forecasts up to 2040. J Affect Disord. 2026;393(Pt A):120299. doi: 10.1016/j.jad.2025.120299 40935255

[13] Xiong P, Liu M, Liu B, Hall BJ. Trends in the incidence and DALYs of anxiety disorders at the global, regional, and national levels: Estimates from the Global Burden of Disease Study 2019. J Affect Disord. 2022;297:83–93. doi: 10.1016/j.jad.2021.10.022 34678404

[14] Bank W. Country and lending groups – 2024. Washington (DC): World Bank. 2024.

[15] First MB, Reed GM, Hyman SE, Saxena S. The development of the ICD-11 Clinical Descriptions and Diagnostic Guidelines for Mental and Behavioural Disorders. World Psychiatry. 2015;14(1):82–90. doi: 10.1002/wps.20189 25655162 PMC4329901

[16] Hay SI, Ong KL, Santomauro DF, A B, Aalipour MA, Aalruz H, et al. Burden of 375 diseases and injuries, risk-attributable burden of 88 risk factors, and healthy life expectancy in 204 countries and territories, including 660 subnational locations, 1990–2023: a systematic analysis for the Global Burden of Disease Study 2023. The Lancet. 2025;406(10513):1873–922. 10.1016/S0140-6736(25)01637-X PMC12535840 41092926

[17] Naghavi M, Kyu HH, A B, Aalipour MA, Aalruz H, Ababneh HS, et al. Global burden of 292 causes of death in 204 countries and territories and 660 subnational locations, 1990–2023: a systematic analysis for the Global Burden of Disease Study 2023. The Lancet. 2025;406(10513):1811–72. 10.1016/S0140-6736(25)01917-8 PMC12535838 41092928

[18] Knoll M, Furkel J, Debus J, Abdollahi A, Karch A, Stock C. An R package for an integrated evaluation of statistical approaches to cancer incidence projection. BMC Med Res Methodol. 2020;20(1):257. doi: 10.1186/s12874-020-01133-5 33059585 PMC7559591

[19] Yuan K, Zheng Y-B, Wang Y-J, Sun Y-K, Gong Y-M, Huang Y-T, et al. A systematic review and meta-analysis on prevalence of and risk factors associated with depression, anxiety and insomnia in infectious diseases, including COVID-19: a call to action. Mol Psychiatry. 2022;27(8):3214–22. doi: 10.1038/s41380-022-01638-z 35668158 PMC9168354

[20] Al-Aly Z, Davis H, McCorkell L, Soares L, Wulf-Hanson S, Iwasaki A, et al. Long COVID science, research and policy. Nat Med. 2024;30(8):2148–64. doi: 10.1038/s41591-024-03173-6 39122965

[21] Javaid SF, Hashim IJ, Hashim MJ, Stip E, Samad MA, Ahbabi AA. Epidemiology of anxiety disorders: global burden and sociodemographic associations. Middle East Curr Psychiatry. 2023;30(1). doi: 10.1186/s43045-023-00315-3

[22] Henking C, Reeves A, Chrisinger B. Global inequalities in mental health problems: understanding the predictors of lifetime prevalence, treatment utilisation and perceived helpfulness across 111 countries. Prev Med. 2023;177:107769. doi: 10.1016/j.ypmed.2023.107769 37952711

[23] Mental health atlas 2020. Geneva: World Health Organization. 2021.

[24] Gururaj S, Bird M-L, Borschmann K, Eng JJ, Watkins CL, Walker MF, et al. Evidence-based stroke rehabilitation: do priorities for practice change and feasibility of implementation vary across high income, upper and lower-middle income countries?. Disabil Rehabil. 2022;44(17):4611–8. doi: 10.1080/09638288.2021.1910737 33849357

[25] Krolick KN, Shi H. Estrogenic Action in Stress-Induced Neuroendocrine Regulation of Energy Homeostasis. Cells. 2022;11(5):879. doi: 10.3390/cells11050879 35269500 PMC8909319

[26] Blake KR, Brooks RC. Status anxiety mediates the positive relationship between income inequality and sexualization. Proc Natl Acad Sci U S A. 2019;116(50):25029–33. doi: 10.1073/pnas.1909806116 31767766 PMC6911179

[27] Rakesh D, Shiba K, Lamont M, Lund C, Pickett KE, VanderWeele TJ, et al. Economic Inequality and Mental Health: Causality, Mechanisms, and Interventions. Annu Rev Clin Psychol. 2025;21(1):353–77. doi: 10.1146/annurev-clinpsy-081423-025710 40333273

[28] Steare T, Gutiérrez Muñoz C, Sullivan A, Lewis G. The association between academic pressure and adolescent mental health problems: A systematic review. J Affect Disord. 2023;339:302–17. doi: 10.1016/j.jad.2023.07.028 37437728

[29] Gobbi G, Atkin T, Zytynski T, Wang S, Askari S, Boruff J, et al. Association of Cannabis Use in Adolescence and Risk of Depression, Anxiety, and Suicidality in Young Adulthood: A Systematic Review and Meta-analysis. JAMA Psychiatry. 2019;76(4):426–34. doi: 10.1001/jamapsychiatry.2018.4500 30758486 PMC6450286

[30] George AM, Zamboanga BL, Millington E, Ham LS. Social anxiety and drinking game behaviors among Australian university students. Addict Behav. 2019;88:43–7. doi: 10.1016/j.addbeh.2018.08.007 30138776

[31] Chandan JS, Thomas T, Bradbury-Jones C, Russell R, Bandyopadhyay S, Nirantharakumar K, et al. Female survivors of intimate partner violence and risk of depression, anxiety and serious mental illness. Br J Psychiatry. 2020;217(4):562–7. doi: 10.1192/bjp.2019.124 31171045

[32] Ahmadabadi Z, Najman JM, Williams GM, Clavarino AM, d’Abbs P, Tran N. Intimate partner violence and subsequent depression and anxiety disorders. Soc Psychiatry Psychiatr Epidemiol. 2020;55(5):611–20. doi: 10.1007/s00127-019-01828-1 31912167

[33] Stefania C, Rogier G, Beomonte Zobel S, Velotti P. The Relation of Anxiety and Avoidance Dimensions of Attachment to Intimate Partner Violence: A Meta-Analysis About Victims. Trauma Violence Abuse. 2023;24(2):1047–62. doi: 10.1177/15248380211050595 34779309

[34] Nodzenski M, Davis J. Frontline support services for boys who have experienced child sexual exploitation: A thematic review of survey data from seven countries. Child Abuse Negl. 2023;142(Pt 2):106077. doi: 10.1016/j.chiabu.2023.106077 36764889

[35] Piolanti A, Schmid IE, Fiderer FJ, Ward CL, Stöckl H, Foran HM. Global Prevalence of Sexual Violence Against Children: A Systematic Review and Meta-Analysis. JAMA Pediatr. 2025;179(3):264–72. doi: 10.1001/jamapediatrics.2024.5326 39804632 PMC11877167

[36] COVID-19 Mental Disorders Collaborators. Global prevalence and burden of depressive and anxiety disorders in 204 countries and territories in 2020 due to the COVID-19 pandemic. Lancet. 2021;398(10312):1700–12. doi: 10.1016/S0140-6736(21)02143-7 34634250 PMC8500697

[37] Williams JE, Pykett J. Mental health monitoring apps for depression and anxiety in children and young people: A scoping review and critical ecological analysis. Soc Sci Med. 2022;297:114802. doi: 10.1016/j.socscimed.2022.114802 35192989

[38] The burden of mental disorders across the states of India: the Global Burden of Disease Study 1990-2017. Lancet Psychiatry. 2020;7(2):148–61. 31879245 10.1016/S2215-0366(19)30475-4 PMC7029418

[39] Lutgendorf MA. Intimate Partner Violence and Women’s Health. Obstet Gynecol. 2019;134(3):470–80. doi: 10.1097/AOG.0000000000003326 31403968

