# Supplementary material for: Anxiety disorder burden inequalities across middle- and high-income countries: 1990–2035 trends and projections
Source: PLoS One. 2026 Jul 21;21(7):e0352932. doi: 10.1371/journal.pone.0352932 (PMC13387518; doi:10.1371/journal.pone.0352932)
Supplement: S1 File — (DOCX) [file pone.0352932.s001.docx]

Appendix 1 for “Burden, Trends, and Projections of Anxiety Disorders Across World Bank Middle- and High-Income Regions, 1990–2035: An Analysis Based on the Global Burden of Disease 2023 Study”

This appendix provides further methodological detail for “Burden, Trends, and Projections of Anxiety Disorders Across World Bank Middle- and High-Income Regions, 1990–2035: An Analysis Based on the Global Burden of Disease 2023 Study.”

Portions of this appendix have been reproduced or adapted from GBD 2023 Causes of Death Collaborators 1 and GBD 2023 Disease and Injury and Risk Factor Collaborators.2

目录

[Section 1 List of acronyms and abbreviations 3](#_Toc13479)

[Section 2 Guidelines for Accurate and Transparent Health Estimates Reporting (GATHER) compliance 4](#_Toc1888)

[Table S1 GATHER checklist.GATHER checklist of information that should be included in reports of global health estimates, with description of compliance and location of information for "The Evolving Burden and Future Projections of Male Genitourinary Cancers and Associated Risk Factors in Middle- and High-Income Countries, 1990–2050: A systematic analysis for the Global Burden of Disease Study 2023” 4](#_Toc20787)

[Section 3 References 6](#_Toc30387)

[Section 4 Supplementary tables 7](#_Toc25211)

[Table S2 Gross national income (GNI) per capita in 2023 7](#_Toc6839)

[Table S3 List of International Classification of Diseases (ICD) codes for anxiety disorders. 8](#_Toc3576)

[Table S4 Incidence, prevalence and DALYs number of anxiety disorder in 2023 9](#_Toc29767)

[Table S5 Age-standardized incidence, mortality and DALYs rate of anxiety disorder in 2023 33](#_Toc1361)

[Table S6. Average annual percent change of age-standardized incidence, prevalence and DALYs rate from 1990 to 2021 57](#_Toc14786)

[Table S7 Age-standardized DALYs rates for anxiety disorders attributable to risk factors in 2023. 70](#_Toc2932)

[Table S8 Age-standardized DALYs rate of average annual percent change attributable to risk factors for anxiety disorders from 1990 to 2023 631](#_Toc6202)

# Section 1 List of acronyms and abbreviations

| **Abbreviation/acronym** | **Full phrase** |
| --- | --- |
| APCs | annual percent changes |
| AAPC | average annual percent changes |
| ASDR | age-standardized disability-adjusted life year rate |
| ASIR | age-standardized incidence rate |
| ASPR | age-standardized **prevalence** rate |
| BAPC | Bayesian Age-Period-Cohort |
| BV | **bullying victimization** |
| CI | confidence interval |
| DALYs | disability-adjusted life-years |
| GATHER | Guidelines for Accurate and Transparent Health Estimates Reporting |
| GBD | Global Burden of Disease |
| GDP | gross domestic product |
| GNI | gross national income |
| HIC | high-income countries |
| **IPV** | **intimate partner violence** |
| LMIC | lower-middle-income countries |
| MHIC | middle- and high-income countries |
| MCMC | Markov Chain Monte Carlo |
| PAF | population attributable fraction |
| SVAC | **sexual violence against children** |

# Section 2 Guidelines for Accurate and Transparent Health Estimates Reporting (GATHER) compliance

This study complies with GATHER recommendations.3 See table S1 below for the GATHER checklist. The GATHER recommendations can be found on the[GATHER website.](https://www.who.int/data/gather)

## Table S1 GATHER checklist.GATHER checklist of information that should be included in reports of global health estimates, with description of compliance and location of information for "The Evolving Burden and Future Projections of Male Genitourinary Cancers and Associated Risk Factors in Middle- and High-Income Countries, 1990–2050: A systematic analysis for the Global Burden of Disease Study 2023”

| **Item #** | **Checklist item** | **Reported location** |
| --- | --- | --- |
| Objectives and funding | | |
| 1 | Define the indicator(s), populations (including age, sex, and geographic entities), and time period(s) for which estimates were made. | Manuscript (Methods) |
| 2 | List the funding sources for the work. | Manuscript (Funding) |
| Data Inputs | | |
| For all data inputs from multiple sources that are synthesized as part of the study: | | |
| 3 | Describe how the data were identified and how the data were accessed. | Methods: "Our analysis used publicly available secondary data from the GBD 2023 study." Data Sources Tool:https://ghdx.healthdata.org/gbd-2023/sources Results. https://vuzhub.healthdata.org/gbd-results |
| 4 | Specify the inclusion and exclusion criteria. Identify all ad-hoc exclusions. | No specific inclusion or exclusion criteria |
| 5 | Provide information on all included data sources and their main characteristics. For each data source used, report reference information or contact  name/institution, population represented, data collection method, year(s) of data collection, sex and age range, diagnostic criteria or measurement method, and  sample size, as relevant. | Methods: "Data sources used to produce GBD 2023 estimates are listed in the GBD 2023 Sources Tool (https://ghdx.healthdata.org/gbd-2023/sources)." Appendix 1 Table S2: World Bank income classification based on 2023 GNI per capita. Appendix 1 Table S3: ICD codes for anxiety disorders. |
| 6 | Identify and describe any categories of input data that have potentially important biases (e.g., based on characteristics listed in item 5). | Discussion: "Due to limited access to healthcare infrastructure in UMC and LMC, there is a delay in diagnosis, which in turn increases the disease burden." Limitations: "Availability and quality of raw data pose a limiting factor..." |
| For data inputs that contribute to the analysis but were not synthesized as part of the study: | | |
| 7 | Describe and give sources for any other data inputs. | Not applicable; all data used were directly from GBD 2023. No additional non-synthesized data were used. |
| For all data inputs: | | |
| 8 | Provide all data inputs in a file format from which data can be efficiently  extracted (e.g., a spreadsheet rather than a PDF), including all relevant meta-data listed in item 5. For any data inputs that cannot be shared because of ethical or  legal reasons, such as third-party ownership, provide a contact name or the name of the institution that retains the right to the data. | GBD results are publicly accessible via: https://vuzhub.healthdata.org/gbd-results  GBD code is available at: http://ghdx.healthdata.org/gbd-2023/code  All data are open-access and downloadable in machine-readable formats (CSV, Excel). |
| Data analysis | | |
| 9 | Provide a conceptual overview of the data analysis method. A diagram maybe helpful. | Methods: "We employed the APC model to analyze trends in anxiety disorders incidence across different age groups, time periods, and birth cohorts." "Bayesian Age-Period-Cohort (BAPC) model" was also used. Joinpoint regression for trend analysis. |
| 10 | Provide a detailed description of all steps of the analysis, including mathematical formulae. This description should cover, as relevant, data cleaning, data pre-  processing, data adjustments and weighting of data sources, and mathematical or statistical model(s). | Methods: - Incidence, prevalence, DALYs sourced from GBD 2023 with 95% UIs. - AAPC calculated using joinpoint regression: ln(rate) = β × year + ε. - BAPC model using Bayesian methods and MCMC simulation. - PAF calculation: Population Attributable Fraction formula provided. |
| 11 | Describe how candidate models were evaluated and how the final model(s) were selected. | Methods: "Simulations confirmed that estimates and uncertainty were not impacted by reducing draws from 500 to 250." Model selection based on standard GBD protocols and validation procedures described in GBD methods literature. |
| 12 | Provide the results of an evaluation of model performance, if done, as well as the results of any relevant sensitivity analysis. | Not explicitly reported in manuscript. However, GBD uses extensive validation and uncertainty quantification across multiple iterations. Reference to GBD methodology: "Analytical methods... are described in detail in the GBD methods literature." |
| 13 | Describe methods for calculating uncertainty of the estimates. State which sources of uncertainty were, and were not, accounted for in the uncertainty analysis. | Methods: "Uncertainty was introduced throughout the estimation process." "Mean estimates... represent the mean value across 250 draws... with 95% UIs calculated as the 2.5 and 97.5 percentile values." "Statistical significance determined by whether the 95% CI of the AAPC excludes zero." Uncertainty includes variability in data inputs, modeling assumptions, and parameter estimation. |
| 14 | State how analytic or statistical source code used to generate estimates can be accessed. | Methods: "The statistical code used in GBD 2023 is publicly available online (http://ghdx.healthdata.org/gbd-2023/code)." |
| Results and Discussion | | |
| 15 | Provide published estimates in a file format from which data can be efficiently extracted. | GBD 2023 results are available through online data visualization tools, the Global Health Data Exchange, and the online data query tool |
| 16 | Report a quantitative measure of the uncertainty of the estimates (e.g. uncertainty intervals). | Uncertainty intervals are provided with all results.Uncertinty is available at: https://ghdx.healthdata .org/record/ihme data/gbd-2023-cause specific-mortality-1990-2023 |
| 17 | Interpret results in light of existing evidence. If updating a previous set of estimates, describe the reasons for changes in estimates. | Discussion of methodological changes between GBD rounds provided in the narrative of the anuscript and methods appendix. Manuscript (Methods and Discussion) |
| 18 | Discuss limitations of the estimates. Include a discussion of any modelling assumptions or data limitations that affect interpretation of the estimates. | Discussion of limitations provided in the narrative of the manuscript, as well as in the methodological rite  ups in the methods appendix. Manuscript (Limitations) |

# Section 3 References

1 GBD 2023 Causes of Death Collaborators. Global burden of 294 causes of death in 204 countries and territories and 660 subnational locations, 1990–2023: a systematic analysis for the Global Burden of Disease Study 2023. *The Lancet* (in drafting).

2 GBD 2023 Disease and Injury and Risk Factor Collaborators. Global incidence, prevalence, non-fatal burden and health-adjusted life expectancy for 376 diseases and injuries, including risk-attributable burden for 88 risk factors in 204 countries and territories, and 660 subnational locations, 1990–2023: a systematic analysis for the Global

Burden of Disease Study 2023. *The Lancet* (in drafting).

3 Stevens GA, Alkema L, Black RE, *et al.* Guidelines for Accurate and Transparent Health Estimates Reporting: the GATHER statement. *Lancet* 2016; **388**: e19–23.

4 Page MJ, McKenzie JE, Bossuyt PM, *et al.* The PRISMA 2020 statement: an updated guideline for reporting systematic reviews. *BMJ* 2021; **372**: n71.

# Section 4 Supplementary tables

## Table S2 Gross national income (GNI) per capita in 2023

| *Bank's fiscal year:* | **FY25** |
| --- | --- |
| *Data for calendar year :* | **2023** |
| *Low income (L)* | <= 1,145 |
| *Lower middle income (LM)* | 1,146 - 4,515 |
| *Upper middle income (UM)* | 4,516 - 14,005 |
| *High income (H)* | > 14,005 |

## Table S3 List of International Classification of Diseases (ICD) codes for anxiety disorders.

| **Cause** | **ICD-10** | **ICD-11** |
| --- | --- | --- |
| **Anxiety disorders** | F40、F40.0、F40.1、F40.2、F41、F41.0、F41.1、F41.2、F41.3、F41.9 | 6B00、6B01、6B02、6B03、6B04、6B05、6B0Y、6B0Z |

**Table S4 Incidence, prevalence and DALYs cases of anxiety disorder in 2023**

| Locations | Cause | Sex | Incidence.number..95.UI | Prevalence.number..95.UI | DALYs.number..95.UI |
| --- | --- | --- | --- | --- | --- |
| World Bank High Income | Anxiety disorders | Males | 4079338.6 (3162797.3 to 5299926.1) | 35030201.4 (27197772.0 to 43021569.6) | 4115925.7 (2718983.7 to 5825139.8) |
|  | Anxiety disorders | Females | 6865720.0 (5332987.1 to 8916633.9) | 61790585.7 (49623520.6 to 73853698.1) | 7126866.2 (4822998.1 to 10044959.9) |
|  | Anxiety disorders | Both sexes | 10945058.6 (8492968.2 to 14231416.8) | 96820787.1 (76815024.7 to 115991331.7) | 11242792.0 (7541981.8 to 15834076.5) |
| World Bank Upper Middle Income | Anxiety disorders | Males | 7861936.1 (5,744,556.4 to 11,171,970.8) | 60811792.4 (45788029.2 to 78302607.9) | 7231338.1 (4736362.0 to 10805087.0) |
|  | Anxiety disorders | Females | 11694798.1 (8,688,044.7 to 16,707,109.4) | 96814128.2 (74267002.5 to 122599568.6) | 11319907.6 (7549769.6 to 16704930.4) |
|  | Anxiety disorders | Both sexes | 19556734.2 (14432797.4 to 27833226.0) | 157625920.7 (120055031.7 to 201214567.9) | 18551245.6 (12286131.6 to 27825231.2) |
| World Bank Lower Middle Income | Anxiety disorders | Males | 7790936.9 (5569401.8 to 11121093.2) | 67626622.8 (52075787.4 to 90232928.2) | 8117682.5 (5117282.5 to 12195432.4) |
|  | Anxiety disorders | Females | 12127058.8 (8732303.3 to 17412806.5) | 106712073.9 (83921589.2 to 138970211.6) | 12558173.3 (8180353.2 to 18690802.7) |
|  | Anxiety disorders | Both sexes | 19917995.7 (14269732.5 to 28638335.8) | 174338696.7 (135997376.7 to 229390833.9) | 20675855.8 (13314428.4 to 30619199.1) |
| Albania | Anxiety disorders | Males | 5074.4 (3869.4 to 6863.4) | 50237.7 (37512.6 to 65433.7) | 5929.3 (3725.1 to 8952) |
|  | Anxiety disorders | Females | 9967.3 (7576.9 to 13847.7) | 105556.5 (81792.3 to 134772) | 12310 (8295.3 to 17916.6) |
|  | Anxiety disorders | Both sexes | 15041.7 (11444.6 to 20797.8) | 155794.2 (119255.6 to 200523.1) | 18239.3 (12087.7 to 27037.6) |
| Algeria | Anxiety disorders | Males | 112675.5 (84562.6 to 149159) | 1024801.9 (814402.9 to 1306655.8) | 123168.7 (80390.2 to 182403.1) |
|  | Anxiety disorders | Females | 109732 (82625.5 to 145004.3) | 1132116.4 (893279.9 to 1388770.5) | 133188.2 (90254.5 to 193626) |
|  | Anxiety disorders | Both sexes | 222407.5 (167743.7 to 293764.6) | 2156918.4 (1724396.8 to 2695717.8) | 256356.9 (172558.2 to 373754.4) |
| American Samoa | Anxiety disorders | Males | 122.3 (80.4 to 198.4) | 1083.5 (762.5 to 1662.9) | 129.7 (74.5 to 217.3) |
|  | Anxiety disorders | Females | 156.9 (106 to 241.5) | 1442.7 (1031.7 to 2068.4) | 171.3 (104.8 to 274.6) |
|  | Anxiety disorders | Both sexes | 279.2 (187.2 to 439.4) | 2526.2 (1799.8 to 3740.5) | 301 (179.7 to 484.5) |
| Andorra | Anxiety disorders | Males | 372.4 (301.6 to 483.8) | 3339.3 (2874.8 to 3894.3) | 398.6 (277.3 to 557.3) |
|  | Anxiety disorders | Females | 550.1 (446.9 to 703.1) | 5060.5 (4452.9 to 5800) | 594.2 (428 to 835.3) |
|  | Anxiety disorders | Both sexes | 922.5 (757.6 to 1181.4) | 8399.8 (7269.2 to 9660.6) | 992.7 (703 to 1396.3) |
| Angola | Anxiety disorders | Males | 144595 (109266.2 to 199501.5) | 1228094.4 (966239.5 to 1597190) | 148664.4 (95639.6 to 220641.2) |
|  | Anxiety disorders | Females | 162456.4 (123188.5 to 216775.5) | 1484436 (1190460.9 to 1862538.3) | 176376 (112686.5 to 259506.5) |
|  | Anxiety disorders | Both sexes | 307051.3 (233958 to 415544.6) | 2712530.5 (2135367 to 3438844.5) | 325040.5 (208326.1 to 477061.7) |
| Antigua and Barbuda | Anxiety disorders | Males | 267.1 (201.4 to 361.8) | 2517.3 (1886.5 to 3105.2) | 296.8 (195.4 to 442.2) |
|  | Anxiety disorders | Females | 420.4 (313.5 to 582.1) | 4318.7 (3298.4 to 5350.3) | 501 (335.3 to 748.7) |
|  | Anxiety disorders | Both sexes | 687.4 (514.7 to 943.1) | 6836.1 (5185.8 to 8480.2) | 797.8 (529.7 to 1196.2) |
| Argentina | Anxiety disorders | Males | 184275 (132436.6 to 247462.6) | 1637173.6 (1236724.7 to 2109802) | 195651.8 (125577.1 to 289020.4) |
|  | Anxiety disorders | Females | 314786.7 (232839.9 to 430892.9) | 2992848.7 (2297633.3 to 3763018.9) | 351458.7 (228065.5 to 514684) |
|  | Anxiety disorders | Both sexes | 499061.6 (365423.9 to 681012.1) | 4630022.3 (3552332.9 to 5890496) | 547110.5 (354021.5 to 804171) |
| Armenia | Anxiety disorders | Males | 8794.1 (6640.4 to 12210.6) | 85101.1 (64722.4 to 111228.5) | 10076.1 (6361.9 to 15085.6) |
|  | Anxiety disorders | Females | 13017.1 (9711.1 to 18084.5) | 137919.1 (107125.5 to 176638.3) | 16048.9 (10412.1 to 23754.5) |
|  | Anxiety disorders | Both sexes | 21811.1 (16361.9 to 30131.5) | 223020.2 (172268.6 to 290825.9) | 26125 (16864.6 to 38773.6) |
| Australia | Anxiety disorders | Males | 168247.9 (132674.3 to 212964.5) | 1169991.3 (995533.6 to 1386882.3) | 138906.4 (94426.4 to 193484.3) |
|  | Anxiety disorders | Females | 250247.9 (204810.6 to 309353.3) | 1851867.7 (1604521 to 2102757.4) | 215233.2 (149374.7 to 292340.5) |
|  | Anxiety disorders | Both sexes | 418495.8 (338473.6 to 522627.3) | 3021859 (2601628.3 to 3476572.5) | 354139.6 (242051.8 to 487031.6) |
| Austria | Anxiety disorders | Males | 28661 (21568 to 37517.1) | 247545.7 (184382.2 to 309232.7) | 29313.9 (19011.8 to 42941.8) |
|  | Anxiety disorders | Females | 44284.6 (30836.8 to 61863.5) | 411028.9 (300779.2 to 518842.3) | 48125.8 (31619.1 to 70826.3) |
|  | Anxiety disorders | Both sexes | 72945.5 (51979.5 to 99329.1) | 658574.5 (485161.4 to 824713.7) | 77439.7 (50766.5 to 113034.3) |
| Azerbaijan | Anxiety disorders | Males | 18711.9 (13519.9 to 25813.4) | 186741.4 (132272.9 to 247570.2) | 22428.2 (14049.1 to 34068) |
|  | Anxiety disorders | Females | 19704.4 (14657 to 27431.5) | 236847.1 (173442.6 to 305407.2) | 27954.1 (17786.3 to 41975.6) |
|  | Anxiety disorders | Both sexes | 38416.3 (28220.8 to 53492.5) | 423588.5 (305452.2 to 554089.5) | 50382.2 (31989.1 to 76129.7) |
| Bahamas | Anxiety disorders | Males | 1201.3 (878.3 to 1636) | 11833 (8915.2 to 14892.6) | 1402.2 (905.3 to 2096) |
|  | Anxiety disorders | Females | 1976.2 (1436.6 to 2764.2) | 20872.1 (16047.2 to 26153.1) | 2436.3 (1608.7 to 3662.9) |
|  | Anxiety disorders | Both sexes | 3177.5 (2314.2 to 4386.5) | 32705.1 (24964.6 to 40802.1) | 3838.5 (2528.3 to 5757.2) |
| Bahrain | Anxiety disorders | Males | 4237.8 (3253.7 to 5764.4) | 45757.2 (36604.4 to 55990.6) | 5481.8 (3650.2 to 7917.4) |
|  | Anxiety disorders | Females | 3587.6 (2665.6 to 4775.4) | 39156.1 (30777.4 to 47634.2) | 4599.3 (3130.5 to 6598.6) |
|  | Anxiety disorders | Both sexes | 7825.5 (5937.5 to 10647.3) | 84913.3 (67381.7 to 103316.1) | 10081.1 (6785 to 14403) |
| Bangladesh | Anxiety disorders | Males | 415756.4 (286592.1 to 592820.1) | 3724966.9 (2722632.8 to 5003663.4) | 447734.8 (275342.5 to 682799) |
|  | Anxiety disorders | Females | 518395 (365347.6 to 717021) | 4898033.5 (3666280.8 to 6334817.6) | 577817.7 (369099.2 to 881622.1) |
|  | Anxiety disorders | Both sexes | 934151.5 (651495.2 to 1295020.9) | 8623000.5 (6388913.5 to 11319966.7) | 1025552.4 (645083.1 to 1557809.4) |
| Barbados | Anxiety disorders | Males | 872.4 (671.5 to 1177.3) | 8271.7 (6385 to 10053) | 969.9 (651.7 to 1414.9) |
|  | Anxiety disorders | Females | 1406.5 (1071 to 1891.6) | 14644.2 (11205.6 to 17610.7) | 1687.1 (1165.4 to 2513.2) |
|  | Anxiety disorders | Both sexes | 2278.8 (1744 to 3077.9) | 22915.9 (17655.9 to 27597.4) | 2657 (1810.4 to 3917.1) |
| Belarus | Anxiety disorders | Males | 13344.7 (9851.9 to 18835.6) | 127871.8 (94881 to 165702.8) | 15135.3 (9690.6 to 22835.9) |
|  | Anxiety disorders | Females | 25139.4 (18916.8 to 34684) | 258058.5 (200886.1 to 330479.3) | 29887.7 (19652.6 to 44691) |
|  | Anxiety disorders | Both sexes | 38484.1 (29118.4 to 53602.3) | 385930.3 (299739.2 to 497241.7) | 45023 (29358.7 to 68082.9) |
| Belgium | Anxiety disorders | Males | 42242.1 (31550.2 to 55408.6) | 368683.8 (276364.4 to 462771.9) | 43609.4 (28125.6 to 64054.3) |
|  | Anxiety disorders | Females | 59932.8 (42427.9 to 81663.4) | 568297.1 (421911.1 to 714667.6) | 66028.9 (43288.4 to 95743.3) |
|  | Anxiety disorders | Both sexes | 102174.9 (73996.8 to 137217.8) | 936980.9 (697784.7 to 1176175.6) | 109638.2 (71556.2 to 159126.4) |
| Belize | Anxiety disorders | Males | 1249.3 (892.6 to 1714.9) | 11831.2 (8911.6 to 15325) | 1414.8 (894.1 to 2123.2) |
|  | Anxiety disorders | Females | 1853.2 (1328.6 to 2554) | 18227 (14028.3 to 23025.2) | 2144.3 (1393.5 to 3205.2) |
|  | Anxiety disorders | Both sexes | 3102.4 (2198.4 to 4240.4) | 30058.2 (22939.9 to 37987.2) | 3559 (2297.2 to 5344.9) |
| Benin | Anxiety disorders | Males | 60605.8 (40581.7 to 96055) | 443854.7 (324748.5 to 645227.8) | 53851.2 (32732.1 to 83103.7) |
|  | Anxiety disorders | Females | 102044.4 (66475.7 to 165017.5) | 764987.5 (566747.2 to 1047221.7) | 91576.1 (57174.6 to 144784.2) |
|  | Anxiety disorders | Both sexes | 162650.2 (107759.8 to 262611.8) | 1208842.2 (897533.9 to 1694456.7) | 145427.4 (89785.4 to 227160.6) |
| Bermuda | Anxiety disorders | Males | 184.3 (141.7 to 255.9) | 1760.9 (1346.5 to 2179.5) | 205.1 (137.5 to 294.6) |
|  | Anxiety disorders | Females | 287.6 (216.8 to 393.6) | 3035.3 (2304 to 3704.6) | 349.3 (240 to 518.1) |
|  | Anxiety disorders | Both sexes | 471.9 (357.5 to 649.5) | 4796.2 (3651.6 to 5841.9) | 554.3 (377.1 to 814.5) |
| Bhutan | Anxiety disorders | Males | 1838.4 (1365.2 to 2471.9) | 14789.2 (11542.8 to 19093.3) | 1783.2 (1173.7 to 2744) |
|  | Anxiety disorders | Females | 2119.4 (1580.8 to 2801.7) | 18327.6 (14662.4 to 23676) | 2152.4 (1470.2 to 3273.8) |
|  | Anxiety disorders | Both sexes | 3957.8 (2942.2 to 5266.8) | 33116.8 (26399.4 to 42723.4) | 3935.6 (2673.4 to 5998.5) |
| Bolivia (Plurinational State of) | Anxiety disorders | Males | 49548.1 (35153.3 to 67322.6) | 479212.4 (358123.5 to 615355.7) | 57314.2 (36222.8 to 85809.4) |
|  | Anxiety disorders | Females | 73956.5 (51954.3 to 101327.2) | 753756.8 (566651.3 to 939946.1) | 88598.9 (56620.4 to 131655.4) |
|  | Anxiety disorders | Both sexes | 123504.6 (87074.6 to 169239.2) | 1232969.2 (924911.6 to 1533498.6) | 145913.1 (92859.2 to 217031.8) |
| Bosnia and Herzegovina | Anxiety disorders | Males | 6862.4 (5216.8 to 9395.6) | 70450.6 (53401.6 to 90253.1) | 8225.9 (5438.7 to 12227.5) |
|  | Anxiety disorders | Females | 12918.9 (9798.1 to 17921.9) | 143962.6 (115055.9 to 182205.6) | 16624.3 (11160 to 24079.6) |
|  | Anxiety disorders | Both sexes | 19781.2 (15009.4 to 27439.3) | 214413.2 (168493.4 to 272352.3) | 24850.2 (16637.2 to 36189.7) |
| Botswana | Anxiety disorders | Males | 6159.1 (4290.3 to 8598.7) | 57442.5 (42894.1 to 77105.7) | 6872 (4337.1 to 10369.3) |
|  | Anxiety disorders | Females | 8302.5 (5880.8 to 11615.1) | 79154.2 (60025.4 to 102874.7) | 9308.4 (5920.6 to 13942.6) |
|  | Anxiety disorders | Both sexes | 14461.7 (10082.9 to 20390) | 136596.7 (103116.1 to 178641.4) | 16180.4 (10136.7 to 24098.2) |
| Brazil | Anxiety disorders | Males | 957040.1 (734792.4 to 1290982.6) | 8773672.4 (6798438 to 10743592.5) | 1030070.7 (689142.9 to 1525918.3) |
|  | Anxiety disorders | Females | 1846052.5 (1405372.1 to 2538612) | 17517344.6 (13792042.6 to 21267875.9) | 2019238.9 (1379730.1 to 2970454.4) |
|  | Anxiety disorders | Both sexes | 2803092.5 (2140154.9 to 3825209.8) | 26291017 (20676211.4 to 31985344.4) | 3049309.6 (2068433.1 to 4500070.8) |
| Brunei Darussalam | Anxiety disorders | Males | 1025.1 (724.4 to 1429.7) | 8187.7 (5977.2 to 11243.5) | 989 (597 to 1533.6) |
|  | Anxiety disorders | Females | 1314.1 (930.9 to 1824.8) | 11426.6 (8355.2 to 15181.2) | 1364.2 (852.9 to 2037.2) |
|  | Anxiety disorders | Both sexes | 2339.2 (1655.5 to 3221.2) | 19614.3 (14333.7 to 26447.5) | 2353.1 (1448 to 3557.1) |
| Bulgaria | Anxiety disorders | Males | 9963.2 (7496.4 to 14022.6) | 103236.6 (79203.2 to 135274.7) | 12065.4 (7931.2 to 18269.1) |
|  | Anxiety disorders | Females | 21939.6 (16742.4 to 30574.2) | 246156.3 (195506.2 to 315262.6) | 28352.1 (18951 to 42151.2) |
|  | Anxiety disorders | Both sexes | 31902.7 (24323.2 to 44687.1) | 349392.8 (277775.2 to 450452.2) | 40417.5 (26883 to 60460.3) |
| Cabo Verde | Anxiety disorders | Males | 1814.5 (1322.2 to 2556.7) | 16715 (13016.2 to 21723.8) | 2020.4 (1281.9 to 2991.4) |
|  | Anxiety disorders | Females | 2615.6 (1841.7 to 3808.8) | 24689 (19130.5 to 32506.2) | 2941.8 (1916 to 4463.9) |
|  | Anxiety disorders | Both sexes | 4430.1 (3196.8 to 6369) | 41404 (32616.1 to 53535.2) | 4962.2 (3205 to 7454.5) |
| Cambodia | Anxiety disorders | Males | 30465.9 (23445.2 to 39932.5) | 240701.9 (188358.5 to 296478.3) | 29059.3 (18915.4 to 41887.2) |
|  | Anxiety disorders | Females | 49540.8 (37831 to 65876.6) | 417440.4 (324636.4 to 513029.3) | 49372.7 (32938.4 to 71581.2) |
|  | Anxiety disorders | Both sexes | 80006.7 (61276.2 to 105735.1) | 658142.2 (512994.9 to 801879.3) | 78432 (51809.6 to 112869.5) |
| Cameroon | Anxiety disorders | Males | 132322.5 (89200.9 to 207269.5) | 1121631.2 (823960 to 1592037.1) | 135954.5 (81366.5 to 207252) |
|  | Anxiety disorders | Females | 190044.5 (123874.4 to 305958.2) | 1659598 (1216751.9 to 2282753.5) | 198491 (122928.8 to 315715.8) |
|  | Anxiety disorders | Both sexes | 322367 (214111.9 to 516240.5) | 2781229.2 (2056212.1 to 3882363.7) | 334445.6 (204114.8 to 522653.2) |
| Canada | Anxiety disorders | Males | 130170.8 (98785.1 to 168872.6) | 1054886.4 (838595.9 to 1323944.8) | 123845.2 (80868.6 to 183535.1) |
|  | Anxiety disorders | Females | 209474.6 (159246.9 to 272566.3) | 1829809 (1433371.1 to 2200004.9) | 209997.5 (140295.1 to 302435.8) |
|  | Anxiety disorders | Both sexes | 339645.4 (258031.9 to 440758.1) | 2884695.4 (2274074.8 to 3531656.8) | 333842.7 (220987.6 to 485137.1) |
| Chile | Anxiety disorders | Males | 74368.9 (55252.2 to 100844.5) | 681779.6 (508126.3 to 869614.7) | 81290.3 (52611.4 to 121552.3) |
|  | Anxiety disorders | Females | 128190.7 (95341.9 to 175724.8) | 1247235.6 (965936.9 to 1555908.6) | 146061.2 (97218.3 to 215255.3) |
|  | Anxiety disorders | Both sexes | 202559.7 (150919.2 to 275852.3) | 1929015.2 (1471768.3 to 2421978.6) | 227351.5 (149955.2 to 338644) |
| China | Anxiety disorders | Males | 3944115.1 (2728654.8 to 6046681.5) | 24142396.1 (18138757 to 33046075.8) | 2876583.6 (1857824.3 to 4455218.9) |
|  | Anxiety disorders | Females | 5384778.5 (3671846.3 to 8256228) | 34527411.6 (26407142.1 to 46260554.2) | 4064368.2 (2662029.3 to 6253301.4) |
|  | Anxiety disorders | Both sexes | 9328893.6 (6368533.1 to 14302909.5) | 58669807.7 (44481130.1 to 79260456.2) | 6940951.8 (4521052.9 to 10656158.2) |
| Colombia | Anxiety disorders | Males | 123238.6 (92826.7 to 168112.1) | 1150286.5 (884603 to 1441974.5) | 136372.7 (91083.3 to 205403.1) |
|  | Anxiety disorders | Females | 229224 (173533.6 to 316561.1) | 2283590 (1796189.5 to 2836399.1) | 265971 (180205.2 to 395193.2) |
|  | Anxiety disorders | Both sexes | 352462.6 (266332.1 to 484673.2) | 3433876.4 (2681524.6 to 4281883.7) | 402343.8 (271552.8 to 600596.3) |
| Comoros | Anxiety disorders | Males | 2818.8 (2062.6 to 3617.6) | 23954.8 (18481.8 to 29436.5) | 2895.8 (1885.1 to 4312) |
|  | Anxiety disorders | Females | 3034.5 (2264.3 to 3947.4) | 27182.2 (21568.7 to 32854.9) | 3228.1 (2133.1 to 4701.6) |
|  | Anxiety disorders | Both sexes | 5853.3 (4314.5 to 7533.6) | 51136.9 (40074.1 to 62746) | 6123.9 (4016.9 to 8919.9) |
| Congo | Anxiety disorders | Males | 21374.6 (16270.4 to 28321.3) | 180789.4 (142055.9 to 231099.8) | 21764.5 (14144.4 to 32048.7) |
|  | Anxiety disorders | Females | 25329.9 (19080.6 to 33609.5) | 229188.5 (183103.6 to 288375.6) | 27125.8 (17831.6 to 39279.9) |
|  | Anxiety disorders | Both sexes | 46704.5 (35402.9 to 61566.3) | 409977.8 (324978.6 to 518661.8) | 48890.3 (31756 to 70657.2) |
| Costa Rica | Anxiety disorders | Males | 14183.1 (10749.4 to 19367) | 127663.1 (95872.3 to 160283.7) | 15050 (9945.7 to 22689.1) |
|  | Anxiety disorders | Females | 22489.5 (16896.9 to 31516.2) | 218509.3 (168027.6 to 274509.8) | 25358.5 (16841 to 37911.9) |
|  | Anxiety disorders | Both sexes | 36672.6 (27624.4 to 50845.6) | 346172.4 (263899.9 to 434815.1) | 40408.4 (26834.4 to 61043.5) |
| C么te d'Ivoire | Anxiety disorders | Males | 139203.1 (96929.3 to 211032.8) | 1129503.4 (836108.5 to 1555882.9) | 136945.2 (84124.8 to 207473.7) |
|  | Anxiety disorders | Females | 197852.3 (131722.8 to 312948.3) | 1633807.6 (1209318.3 to 2207355.3) | 194757.4 (121148.2 to 298961.3) |
|  | Anxiety disorders | Both sexes | 337055.4 (229685.2 to 526879.2) | 2763311 (2061144 to 3781931.4) | 331702.6 (207206.4 to 505074.1) |
| Croatia | Anxiety disorders | Males | 11210.9 (8510.6 to 15196.6) | 104086.5 (80995.3 to 134198.4) | 12113.6 (7825.4 to 18052.9) |
|  | Anxiety disorders | Females | 20408.4 (15421.8 to 27994) | 206566.3 (166524.1 to 262261.4) | 23752.4 (16097.5 to 34400.9) |
|  | Anxiety disorders | Both sexes | 31619.3 (23939.5 to 43377.9) | 310652.7 (248844.7 to 395203.4) | 35866 (23936.3 to 52459.5) |
| Cuba | Anxiety disorders | Males | 34402.7 (26609.6 to 46451.5) | 343863.3 (266624 to 420141.8) | 40354.3 (26808.2 to 59263) |
|  | Anxiety disorders | Females | 51546.6 (39388.8 to 69279) | 573573 (440160.5 to 699044.2) | 66241.9 (45257.4 to 97998.6) |
|  | Anxiety disorders | Both sexes | 85949.2 (65851.8 to 115841.6) | 917436.3 (707510.6 to 1110120.3) | 106596.1 (72346.8 to 158040.2) |
| Cyprus | Anxiety disorders | Males | 5988.8 (4790.3 to 7837.1) | 52359.8 (45293.2 to 61341.8) | 6273.6 (4401.1 to 8875.3) |
|  | Anxiety disorders | Females | 10860.7 (8759.5 to 13897.3) | 94582 (83481.4 to 108846.1) | 11173.6 (8023.4 to 15506.4) |
|  | Anxiety disorders | Both sexes | 16849.5 (13702.2 to 21767.7) | 146941.8 (129204.6 to 169366.6) | 17447.1 (12498 to 24506.1) |
| Czechia | Anxiety disorders | Males | 18513.1 (14022.9 to 26319.7) | 179483.9 (134741.5 to 235378.5) | 20988 (13757.9 to 31717.4) |
|  | Anxiety disorders | Females | 40818.4 (30896.7 to 57438.5) | 450630.9 (358159.6 to 574446.7) | 51832.8 (35158.7 to 76138) |
|  | Anxiety disorders | Both sexes | 59331.5 (44919.6 to 83514.1) | 630114.7 (492416.2 to 810443.1) | 72820.9 (49003.1 to 107532.6) |
| Denmark | Anxiety disorders | Males | 20130.4 (16168.6 to 25771.2) | 157152.5 (138370.1 to 180119.9) | 18658.9 (12630.4 to 25858.3) |
|  | Anxiety disorders | Females | 34220.2 (27939.1 to 43035.1) | 278732.8 (243469.5 to 319141.6) | 32749.2 (23574.3 to 45650.3) |
|  | Anxiety disorders | Both sexes | 54350.6 (44480.9 to 67869.9) | 435885.4 (381930.4 to 496630.2) | 51408.1 (36256.2 to 70870.9) |
| Djibouti | Anxiety disorders | Males | 4070.8 (2977.8 to 5240.4) | 34091.6 (26486 to 41934.3) | 4127.3 (2674.2 to 6091.4) |
|  | Anxiety disorders | Females | 3815.8 (2879.4 to 5024.6) | 32671 (26059.8 to 41167.8) | 3890 (2608.7 to 5902.5) |
|  | Anxiety disorders | Both sexes | 7886.6 (5857.5 to 10264.9) | 66762.6 (52554.8 to 82384.8) | 8017.3 (5243.8 to 11917.5) |
| Dominica | Anxiety disorders | Males | 214.5 (163.3 to 288.5) | 2028.3 (1521.8 to 2522.9) | 239 (158.2 to 353.5) |
|  | Anxiety disorders | Females | 309.3 (232.5 to 420.7) | 3137.5 (2382.6 to 3828.3) | 363.6 (246.5 to 549.1) |
|  | Anxiety disorders | Both sexes | 523.8 (395.8 to 705.9) | 5165.8 (3897.5 to 6274.2) | 602.5 (404.7 to 904.5) |
| Dominican Republic | Anxiety disorders | Males | 36372.2 (26412.4 to 49828.8) | 329359.4 (251361.2 to 424859.2) | 39173.1 (25205 to 59468.8) |
|  | Anxiety disorders | Females | 61985.5 (44413.7 to 85827.9) | 589416.3 (455585.7 to 736813.6) | 68928 (44525.6 to 102919.2) |
|  | Anxiety disorders | Both sexes | 98357.7 (70767 to 134938.2) | 918775.6 (707244.1 to 1144228.5) | 108101.1 (69706.8 to 160773.6) |
| Ecuador | Anxiety disorders | Males | 63995.7 (46509.5 to 85984.5) | 611796.2 (450808.8 to 785027.1) | 73079 (46338.3 to 110116.6) |
|  | Anxiety disorders | Females | 98504.1 (70472.7 to 134954.8) | 996402.8 (740545.5 to 1236515) | 117257 (74760.1 to 173451.9) |
|  | Anxiety disorders | Both sexes | 162499.8 (116944.2 to 220454.5) | 1608199 (1191357.3 to 1986949.8) | 190336 (120838.1 to 282740) |
| Egypt | Anxiety disorders | Males | 318828.8 (209959.5 to 503889.1) | 2996962.2 (2169457.6 to 4200940.9) | 360917.5 (209929 to 559576.4) |
|  | Anxiety disorders | Females | 375031.8 (247206.1 to 568109.7) | 3746039.5 (2754475 to 5140246.2) | 443453.9 (274334.1 to 660232.1) |
|  | Anxiety disorders | Both sexes | 693860.6 (457007.6 to 1079976.4) | 6743001.8 (4947304.9 to 9334287.2) | 804371.3 (484263 to 1226765) |
| El Salvador | Anxiety disorders | Males | 13724.5 (10142.7 to 18837) | 130760.7 (98963.9 to 164338.1) | 15468.6 (10004.3 to 23252.1) |
|  | Anxiety disorders | Females | 26114.7 (19415.7 to 36875.2) | 269966 (207373 to 339120.4) | 31582 (21032.1 to 47064.5) |
|  | Anxiety disorders | Both sexes | 39839.2 (29520.2 to 55838.8) | 400726.8 (307689.9 to 502228.7) | 47050.6 (31053.4 to 69945.6) |
| Equatorial Guinea | Anxiety disorders | Males | 6450.8 (4801.4 to 8583.1) | 56941.4 (43379.2 to 73773.4) | 6881.9 (4391.2 to 10132.4) |
|  | Anxiety disorders | Females | 5952.6 (4461.4 to 7901) | 54999.7 (43847.7 to 69197.2) | 6535.5 (4268.8 to 9460.6) |
|  | Anxiety disorders | Both sexes | 12403.4 (9266.7 to 16411.1) | 111941 (86547.6 to 142661.7) | 13417.4 (8714.8 to 19633.2) |
| Estonia | Anxiety disorders | Males | 2198.8 (1658.2 to 3100.5) | 20824.8 (15765.5 to 26994) | 2456.6 (1592.2 to 3690.5) |
|  | Anxiety disorders | Females | 3605 (2743.3 to 4927.7) | 37645.9 (29448.6 to 48646.4) | 4343.1 (2791.2 to 6457.4) |
|  | Anxiety disorders | Both sexes | 5803.8 (4442.3 to 7988.1) | 58470.8 (45684.6 to 75616.8) | 6799.7 (4381.1 to 10151.5) |
| Eswatini | Anxiety disorders | Males | 2769.8 (1927.1 to 3921.4) | 28049.7 (20864.6 to 37419) | 3369.1 (2046.4 to 5098.9) |
|  | Anxiety disorders | Females | 4104.5 (2885.4 to 5847.4) | 41807.9 (31647.7 to 55102.8) | 4914.5 (3086.5 to 7510.4) |
|  | Anxiety disorders | Both sexes | 6874.3 (4773.6 to 9790.9) | 69857.6 (52572.8 to 91591.2) | 8283.7 (5134.3 to 12583.9) |
| Fiji | Anxiety disorders | Males | 2282.1 (1492 to 3704.1) | 19562.4 (14041.5 to 29481.9) | 2342.2 (1392.5 to 3877.3) |
|  | Anxiety disorders | Females | 2972.2 (1985.4 to 4525) | 26654 (19239 to 37456.1) | 3148.7 (1932 to 4994.2) |
|  | Anxiety disorders | Both sexes | 5254.3 (3474.8 to 8196.9) | 46216.4 (33280.5 to 66941.3) | 5490.9 (3330.4 to 8829.9) |
| Finland | Anxiety disorders | Males | 16040.7 (12212.7 to 20838.8) | 126598 (95393.5 to 159066.9) | 14884.5 (9507.5 to 21525) |
|  | Anxiety disorders | Females | 17433.9 (12613.4 to 24090.6) | 154331.5 (113842 to 198470.3) | 17973.7 (11575.2 to 26279.3) |
|  | Anxiety disorders | Both sexes | 33474.6 (24807.8 to 45344.9) | 280929.5 (209183.2 to 357054.5) | 32858.2 (21009.3 to 47515.4) |
| France | Anxiety disorders | Males | 274499.5 (205392.4 to 358747.4) | 2286332.1 (1730421.9 to 2884834.4) | 269734.2 (177430.3 to 396708.8) |
|  | Anxiety disorders | Females | 458705.2 (325741.2 to 623680) | 4284843.4 (3260381 to 5333825.2) | 498615.7 (330070.8 to 718584.4) |
|  | Anxiety disorders | Both sexes | 733204.7 (532215.5 to 984635.1) | 6571175.5 (4989358.9 to 8202981.8) | 768349.8 (512440.5 to 1116758) |
| Gabon | Anxiety disorders | Males | 6661.9 (5123.2 to 8729.8) | 57529.9 (45706.9 to 71768.7) | 6908.3 (4459.5 to 9964.1) |
|  | Anxiety disorders | Females | 7988.5 (6079.1 to 10648.9) | 73938.7 (59186.8 to 92054.6) | 8685.8 (5784.4 to 12647) |
|  | Anxiety disorders | Both sexes | 14650.4 (11208.4 to 19195.5) | 131468.6 (104307.9 to 163280.7) | 15594 (10251.5 to 22619.1) |
| Georgia | Anxiety disorders | Males | 5494.4 (4146.8 to 7725) | 53133.5 (39786.4 to 69844.6) | 6286.9 (3984 to 9436) |
|  | Anxiety disorders | Females | 9354.6 (6972 to 12970.4) | 98284.6 (75100.8 to 129638.9) | 11372.4 (7343.9 to 16726.4) |
|  | Anxiety disorders | Both sexes | 14848.9 (11133.7 to 20731.6) | 151418.1 (115573.3 to 197339.4) | 17659.3 (11301.3 to 26119.6) |
| Germany | Anxiety disorders | Males | 223447.2 (179767.2 to 280541) | 1878458.6 (1602224.6 to 2193351.8) | 221279.6 (150206.5 to 312270.7) |
|  | Anxiety disorders | Females | 343419 (271837.4 to 441401.1) | 3011841.4 (2616368.6 to 3505957.6) | 349529.4 (242279.5 to 491750.1) |
|  | Anxiety disorders | Both sexes | 566866.2 (452393.2 to 722427.3) | 4890300 (4225263.9 to 5701475) | 570809 (393531.5 to 806208.7) |
| Ghana | Anxiety disorders | Males | 85403.5 (59908.6 to 128067.6) | 731123.9 (550123.4 to 1010155.7) | 88442.3 (54953.6 to 133918.8) |
|  | Anxiety disorders | Females | 135251.4 (92444.9 to 208361.9) | 1236440.6 (919133.2 to 1679871.6) | 147498.7 (91821.9 to 229080.9) |
|  | Anxiety disorders | Both sexes | 220654.9 (152917.3 to 338113.9) | 1967564.5 (1474992.5 to 2684656.8) | 235941 (147321.8 to 362972.9) |
| Greece | Anxiety disorders | Males | 43835.1 (33308.7 to 56958.4) | 392977.9 (305803.1 to 482354.2) | 46280.2 (30580 to 66935.3) |
|  | Anxiety disorders | Females | 65017.8 (47323.3 to 88490.6) | 635546.4 (493720 to 781544.1) | 73564.4 (49634.1 to 105523.9) |
|  | Anxiety disorders | Both sexes | 108853 (80001.5 to 144788.4) | 1028524.4 (799234.4 to 1262827.5) | 119844.6 (80361.5 to 170981.8) |
| Greenland | Anxiety disorders | Males | 217.5 (162.3 to 289.6) | 1836 (1369.8 to 2360.1) | 216.7 (140.5 to 325.3) |
|  | Anxiety disorders | Females | 351 (257.1 to 479.5) | 2984.2 (2277.4 to 3656) | 346.5 (232.1 to 511.7) |
|  | Anxiety disorders | Both sexes | 568.5 (421.4 to 768) | 4820.2 (3646.8 to 5986.4) | 563.1 (375 to 837) |
| Grenada | Anxiety disorders | Males | 352.5 (268.7 to 474.1) | 3416.2 (2577.6 to 4241.9) | 400.9 (260.4 to 589) |
|  | Anxiety disorders | Females | 500.7 (377 to 681.6) | 5211.2 (3953.1 to 6369.6) | 604.6 (405.3 to 899.9) |
|  | Anxiety disorders | Both sexes | 853.2 (645.6 to 1151.7) | 8627.4 (6512.9 to 10498) | 1005.6 (668.5 to 1487.5) |
| Guam | Anxiety disorders | Males | 368.8 (258.1 to 570.4) | 3232.6 (2289.2 to 4704.1) | 386.2 (234.1 to 618.2) |
|  | Anxiety disorders | Females | 487.8 (350.4 to 730.5) | 4482.3 (3186.9 to 6223.9) | 530.4 (335.2 to 823.4) |
|  | Anxiety disorders | Both sexes | 856.6 (610 to 1302.6) | 7715 (5463.2 to 10866) | 916.6 (566.4 to 1431.8) |
| Guatemala | Anxiety disorders | Males | 39088.1 (28398.2 to 53793.9) | 365984.2 (273885 to 473107.6) | 43363.6 (27423.8 to 65874.4) |
|  | Anxiety disorders | Females | 72327.7 (52203.9 to 102725.4) | 710658.1 (544266.8 to 900856.1) | 83258.2 (54626 to 124103.4) |
|  | Anxiety disorders | Both sexes | 111415.8 (80448.4 to 156659.5) | 1076642.3 (818260.5 to 1360772.4) | 126621.8 (82256 to 189781.8) |
| Guinea | Anxiety disorders | Males | 62743.8 (41742.6 to 100407.6) | 486346 (355325.7 to 695893.9) | 58914.1 (35348.4 to 91009.9) |
|  | Anxiety disorders | Females | 100078.3 (64875.7 to 161995.6) | 796962.7 (585909.3 to 1091426.1) | 95327.2 (59506.5 to 149944.4) |
|  | Anxiety disorders | Both sexes | 162822.1 (107149.9 to 263984.7) | 1283308.7 (952333.8 to 1794888.4) | 154241.4 (94981.2 to 240417.6) |
| Guyana | Anxiety disorders | Males | 2407.5 (1747.4 to 3285.2) | 22580.8 (16985.8 to 29291.3) | 2681.4 (1723.2 to 4063.1) |
|  | Anxiety disorders | Females | 3694.2 (2647.8 to 5136.2) | 36707.8 (28087.7 to 46195.2) | 4279 (2780.9 to 6400) |
|  | Anxiety disorders | Both sexes | 6101.7 (4393.8 to 8398.1) | 59288.6 (45073.5 to 74080.6) | 6960.4 (4521 to 10509.7) |
| Haiti | Anxiety disorders | Males | 43564.8 (30779 to 60243.7) | 346755.1 (263990.2 to 457519.3) | 41496.2 (26025.5 to 63197.1) |
|  | Anxiety disorders | Females | 59994.2 (43330 to 82086.1) | 522182.6 (403190.9 to 658252.4) | 60777.3 (39775.6 to 90920) |
|  | Anxiety disorders | Both sexes | 103559 (74108.9 to 141497.6) | 868937.7 (669193.6 to 1104248.4) | 102273.5 (65924.5 to 154841.9) |
| Honduras | Anxiety disorders | Males | 23222.8 (16845.9 to 31989.8) | 222676 (166383.8 to 287872.7) | 26580 (16905.8 to 40286.2) |
|  | Anxiety disorders | Females | 42627 (30768.1 to 60729.5) | 436779.1 (336019.4 to 557516.2) | 51387 (33642.6 to 77052.4) |
|  | Anxiety disorders | Both sexes | 65849.8 (47420.7 to 92726) | 659455.2 (502531.9 to 837387.4) | 77967 (50288.5 to 117214) |
| Hungary | Anxiety disorders | Males | 14120.1 (10797.8 to 19451.3) | 147655 (111540.7 to 188570.8) | 17296.8 (11363.8 to 25801.2) |
|  | Anxiety disorders | Females | 32001.2 (24325.4 to 44212.5) | 364476.9 (293540.9 to 460574.4) | 42002.8 (28654.3 to 62385.8) |
|  | Anxiety disorders | Both sexes | 46121.3 (35152.6 to 63878.6) | 512131.9 (407882.2 to 648370.8) | 59299.7 (40337.4 to 88259.1) |
| Iceland | Anxiety disorders | Males | 1262 (1007.5 to 1626.2) | 10445.5 (9015.2 to 12125.2) | 1246.7 (861.4 to 1750.7) |
|  | Anxiety disorders | Females | 2220.8 (1801.3 to 2805.5) | 18669.8 (16395.8 to 21206.3) | 2196.9 (1589.3 to 3073.4) |
|  | Anxiety disorders | Both sexes | 3482.7 (2834.8 to 4410.1) | 29115.3 (25210.5 to 33176.6) | 3443.5 (2452.8 to 4788.4) |
| India | Anxiety disorders | Males | 3623129.5 (2676590.7 to 4965236.4) | 31917467.1 (25188438.1 to 41094215.6) | 3810692.2 (2500707.7 to 5590874.3) |
|  | Anxiety disorders | Females | 6105234.3 (4577990.9 to 8304038.2) | 54109990.1 (43312952.6 to 68812171) | 6309812.9 (4182979.8 to 9090586.6) |
|  | Anxiety disorders | Both sexes | 9728363.8 (7252892 to 13360184.3) | 86027457.3 (68503049.9 to 109930131) | 10120505.2 (6664701.4 to 14713281) |
| Indonesia | Anxiety disorders | Males | 440501 (330771.5 to 588552.7) | 3848168.6 (2926521.6 to 4742073) | 462749.9 (302834.2 to 684234.6) |
|  | Anxiety disorders | Females | 619511.5 (460172.1 to 833063.9) | 5736829.8 (4400202.8 to 7160400.8) | 678567.9 (447983 to 1022769.8) |
|  | Anxiety disorders | Both sexes | 1060012.5 (790807.1 to 1421616.6) | 9584998.3 (7328669.9 to 11895946.8) | 1141317.8 (750066.7 to 1707004.4) |
| Iran (Islamic Republic of) | Anxiety disorders | Males | 484285.8 (347167.5 to 682663.4) | 4676842.9 (3667375.3 to 6014776.7) | 557295.7 (365856 to 811190.9) |
|  | Anxiety disorders | Females | 583909.1 (423676.2 to 787323.6) | 6119733 (4929525.1 to 7731488.5) | 713767.7 (477656.8 to 1040656.8) |
|  | Anxiety disorders | Both sexes | 1068194.9 (771171.1 to 1465642.2) | 10796575.9 (8596900.4 to 13613244.4) | 1271063.4 (841548.4 to 1844603.1) |
| Iraq | Anxiety disorders | Males | 139843.3 (94252.9 to 205836.3) | 1313933.9 (963888.8 to 1842960.8) | 157825.9 (94675.3 to 240025.3) |
|  | Anxiety disorders | Females | 219172.5 (146804 to 323933.9) | 2117602.2 (1589566.1 to 2869139.1) | 250395.8 (156088.1 to 373180.3) |
|  | Anxiety disorders | Both sexes | 359015.8 (241217 to 542798.6) | 3431536.1 (2553455 to 4753619.2) | 408221.7 (250581.7 to 613736.5) |
| Ireland | Anxiety disorders | Males | 24679.4 (17597.3 to 36225.9) | 204872.5 (153221.3 to 278008.3) | 24483.6 (15806.7 to 36471.2) |
|  | Anxiety disorders | Females | 41078.5 (26772.3 to 60233) | 343815.4 (265581.6 to 434875.1) | 40614.9 (26575.7 to 59041) |
|  | Anxiety disorders | Both sexes | 65757.9 (44562 to 97003.8) | 548687.9 (416182.7 to 713525.9) | 65098.5 (43149.5 to 95118.6) |
| Israel | Anxiety disorders | Males | 31523 (22451.5 to 42011.5) | 260732.7 (189772 to 343349.6) | 31334.1 (19730.5 to 47004.8) |
|  | Anxiety disorders | Females | 39214.8 (24781.9 to 59898.9) | 352968.7 (250771 to 475410.9) | 41836.8 (25984.5 to 63008.7) |
|  | Anxiety disorders | Both sexes | 70737.8 (47922.6 to 101949.2) | 613701.4 (440543 to 824794.9) | 73170.9 (45709.6 to 109514.2) |
| Italy | Anxiety disorders | Males | 202988.1 (154310.4 to 264522.8) | 1786605.7 (1401974.2 to 2218972) | 210737.7 (138992.7 to 307370.2) |
|  | Anxiety disorders | Females | 331520.2 (242419.5 to 451434.2) | 3220282.8 (2549095.3 to 4034124.5) | 373876.7 (250047.5 to 549471.4) |
|  | Anxiety disorders | Both sexes | 534508.3 (395113.1 to 706686.5) | 5006888.5 (3954679.6 to 6266537.6) | 584614.3 (389546.9 to 856961.7) |
| Jamaica | Anxiety disorders | Males | 6812.6 (5060.3 to 9262.9) | 65632.3 (49403.7 to 83106.3) | 7804.7 (5048.8 to 11523.7) |
|  | Anxiety disorders | Females | 10179.2 (7475.2 to 14204.4) | 111925.5 (85997 to 141182) | 13057 (8590.2 to 19421.9) |
|  | Anxiety disorders | Both sexes | 16991.8 (12529.3 to 23476.4) | 177557.8 (135483.5 to 222656.8) | 20861.7 (13621 to 31461.4) |
| Japan | Anxiety disorders | Males | 203620.6 (156520.3 to 273045.2) | 1574359.3 (1264811.6 to 1995114.6) | 185086.5 (120156.9 to 271537) |
|  | Anxiety disorders | Females | 291778.8 (223319.3 to 396346.2) | 2507441.1 (2020112.8 to 3108398.1) | 289438.8 (194600.8 to 418129.1) |
|  | Anxiety disorders | Both sexes | 495399.4 (379918.7 to 669355.6) | 4081800.4 (3295443.3 to 5095001.8) | 474525.3 (314249.6 to 689075.4) |
| Jordan | Anxiety disorders | Males | 35752 (26810.5 to 47354.8) | 360658.7 (280406.9 to 474398.9) | 43614.4 (29066.6 to 64621.7) |
|  | Anxiety disorders | Females | 39526.4 (28891.2 to 52797.9) | 417916.8 (321771.9 to 533247.2) | 49387.1 (33170.2 to 72638.4) |
|  | Anxiety disorders | Both sexes | 75278.4 (55680.9 to 98902.2) | 778575.6 (598215.2 to 1003677.2) | 93001.5 (62864.2 to 137306.1) |
| Kazakhstan | Anxiety disorders | Males | 24745.6 (17255 to 34868.5) | 240112.6 (168406.7 to 324362.7) | 28901.6 (17743.6 to 45098.1) |
|  | Anxiety disorders | Females | 37916.1 (27451.3 to 52587.8) | 408173.8 (303156.8 to 538148.3) | 48301.4 (30358.5 to 73468.4) |
|  | Anxiety disorders | Both sexes | 62661.7 (44721.8 to 87223.8) | 648286.4 (470827.6 to 861207.8) | 77203 (48642.5 to 118229.6) |
| Kenya | Anxiety disorders | Males | 107516.5 (79293 to 138767.1) | 1015717.2 (806106.3 to 1234395) | 122848.8 (79535.6 to 180523.4) |
|  | Anxiety disorders | Females | 132421 (100373.9 to 172687.7) | 1251214.8 (1013523.5 to 1537159.5) | 149204.7 (98112.6 to 219142.2) |
|  | Anxiety disorders | Both sexes | 239937.6 (179666.9 to 309242.3) | 2266932.1 (1819629.8 to 2769273.5) | 272053.5 (178070.4 to 395718.3) |
| Kiribati | Anxiety disorders | Males | 309.9 (200.7 to 524.7) | 2602.7 (1824.4 to 3995.1) | 314.6 (184.5 to 526.3) |
|  | Anxiety disorders | Females | 414.7 (277.7 to 635.3) | 3662.6 (2672.9 to 5162) | 436.1 (262.6 to 689.7) |
|  | Anxiety disorders | Both sexes | 724.6 (482.9 to 1160.3) | 6265.3 (4546.5 to 9161) | 750.7 (445.8 to 1205.2) |
| Kuwait | Anxiety disorders | Males | 12294.6 (9418.8 to 16651.9) | 121923.4 (95260.5 to 150240.1) | 14551.6 (9526.5 to 21410.6) |
|  | Anxiety disorders | Females | 10845.8 (8040.3 to 14487) | 109618.4 (85005.3 to 134764.9) | 12881.7 (8646.5 to 18939.5) |
|  | Anxiety disorders | Both sexes | 23140.4 (17525 to 31607) | 231541.8 (180265.8 to 287293.4) | 27433.3 (18194.9 to 40148.4) |
| Kyrgyzstan | Anxiety disorders | Males | 8038.8 (5514.1 to 11676.5) | 78442.1 (55034.8 to 108949.9) | 9519.5 (5690.6 to 15278.5) |
|  | Anxiety disorders | Females | 13478.2 (9515.7 to 18813.2) | 140223.5 (99689 to 190183.3) | 16729.3 (10237.8 to 26108.2) |
|  | Anxiety disorders | Both sexes | 21517 (15021.4 to 30583.1) | 218665.5 (153435.2 to 299961) | 26248.8 (15996.7 to 41249.6) |
| Lao People's Democratic Republic | Anxiety disorders | Males | 9887.6 (6895.1 to 14409.5) | 74919.2 (55916.9 to 102805) | 9093.6 (5521 to 13757.3) |
|  | Anxiety disorders | Females | 15990.6 (11240.6 to 22284) | 121252.2 (90126.2 to 158894.5) | 14471.7 (9312.2 to 22395.9) |
|  | Anxiety disorders | Both sexes | 25878.2 (18137.1 to 36097.4) | 196171.4 (146043.1 to 263910.3) | 23565.2 (15024.3 to 35891.9) |
| Latvia | Anxiety disorders | Males | 3481.6 (2627.9 to 4927.4) | 32929.1 (25106.8 to 43007.5) | 3883 (2553.7 to 5809.6) |
|  | Anxiety disorders | Females | 6106.3 (4619 to 8355) | 63695.8 (49981.1 to 81732.5) | 7319.4 (4844.8 to 10921) |
|  | Anxiety disorders | Both sexes | 9587.8 (7286.6 to 13181) | 96624.9 (75530 to 124431.6) | 11202.4 (7370.1 to 16660.8) |
| Lebanon | Anxiety disorders | Males | 20962.1 (14372.8 to 30903.7) | 183486.7 (135411.2 to 246260.8) | 21874.8 (13883.1 to 33027) |
|  | Anxiety disorders | Females | 28142.1 (19582.4 to 40203.2) | 274558.6 (207731 to 350990.7) | 32057.6 (20702.1 to 47375.6) |
|  | Anxiety disorders | Both sexes | 49104.2 (34043.9 to 70519) | 458045.3 (344377.8 to 597583.3) | 53932.3 (34585.2 to 80010.1) |
| Lesotho | Anxiety disorders | Males | 6230.6 (4312.8 to 8797.6) | 55147.1 (40544.6 to 73339.9) | 6605.4 (4000.9 to 10036.2) |
|  | Anxiety disorders | Females | 7455.6 (5251.5 to 10519.3) | 69506 (52029.9 to 91879.9) | 8169.4 (5168.2 to 12462.1) |
|  | Anxiety disorders | Both sexes | 13686.2 (9469 to 19337.1) | 124653 (92590.8 to 164284.3) | 14774.7 (9112.1 to 22264) |
| Libya | Anxiety disorders | Males | 18806.8 (14241 to 25306.3) | 188024.3 (148671.4 to 240926.9) | 22467.2 (14806.2 to 32744) |
|  | Anxiety disorders | Females | 25237.4 (18752.8 to 33618.3) | 264646.8 (205558.1 to 325521.4) | 30953.7 (21103.7 to 44954.4) |
|  | Anxiety disorders | Both sexes | 44044.2 (33323.2 to 59092.2) | 452671.2 (352017.9 to 566392.2) | 53420.9 (35940.6 to 78223.1) |
| Lithuania | Anxiety disorders | Males | 4783 (3621.1 to 6711.8) | 47782.5 (36387.3 to 62008.1) | 5619 (3661.6 to 8458.1) |
|  | Anxiety disorders | Females | 8937.1 (6770.2 to 12382.6) | 95170.6 (75057.3 to 121600.3) | 10958.7 (7370.7 to 16182.5) |
|  | Anxiety disorders | Both sexes | 13720 (10418.3 to 19083.5) | 142953.1 (112235.9 to 183197.3) | 16577.8 (11083.3 to 24389.5) |
| Luxembourg | Anxiety disorders | Males | 2291 (1840.2 to 2977.3) | 19747 (17067.4 to 23129.9) | 2360.4 (1626.6 to 3290.1) |
|  | Anxiety disorders | Females | 3259.6 (2637.2 to 4166.1) | 29852.8 (26306.5 to 34249.6) | 3517.7 (2515.7 to 4928.7) |
|  | Anxiety disorders | Both sexes | 5550.6 (4531.5 to 7103.1) | 49599.7 (42948.3 to 57104.6) | 5878 (4154.9 to 8204.2) |
| Malaysia | Anxiety disorders | Males | 37975.2 (29413 to 50530.9) | 328272 (255029.5 to 400825.3) | 39416.3 (25795.4 to 56134.3) |
|  | Anxiety disorders | Females | 53227.6 (40697.2 to 71362) | 484997 (373119.3 to 601219.8) | 57312.2 (38438.8 to 84862.5) |
|  | Anxiety disorders | Both sexes | 91202.8 (70028.4 to 121826.5) | 813269 (627448.5 to 998983.7) | 96728.5 (63981.9 to 142052.8) |
| Maldives | Anxiety disorders | Males | 811.2 (605.4 to 1123.8) | 6902.5 (5545.2 to 8860.9) | 837.2 (557.7 to 1255.3) |
|  | Anxiety disorders | Females | 831.4 (619.6 to 1142.5) | 7233.8 (5660.2 to 9242.5) | 860.2 (575.2 to 1273.8) |
|  | Anxiety disorders | Both sexes | 1642.6 (1222.4 to 2290.3) | 14136.3 (11205.6 to 18114.5) | 1697.4 (1135.9 to 2524.5) |
| Malta | Anxiety disorders | Males | 3520.6 (2824 to 4616.2) | 29509.2 (25795.3 to 34307.4) | 3520.6 (2489.8 to 4947.1) |
|  | Anxiety disorders | Females | 4948.2 (4010.2 to 6307.5) | 41326.8 (36503.3 to 47095.5) | 4854.6 (3485.9 to 6680.4) |
|  | Anxiety disorders | Both sexes | 8468.8 (6875.4 to 10867.5) | 70835.9 (62412.3 to 81302) | 8375.2 (5980.2 to 11653.9) |
| Marshall Islands | Anxiety disorders | Males | 104.3 (67.7 to 175.1) | 896.4 (627.5 to 1384) | 108.1 (61.9 to 183.3) |
|  | Anxiety disorders | Females | 131.4 (87.4 to 202.7) | 1166.7 (851.9 to 1666.4) | 139.1 (84.4 to 220.8) |
|  | Anxiety disorders | Both sexes | 235.7 (155.3 to 377.2) | 2063.1 (1497 to 3060.9) | 247.2 (144.4 to 397) |
| Mauritania | Anxiety disorders | Males | 13389.1 (9089.6 to 20968.3) | 113831.6 (82917.5 to 158728.1) | 13817.8 (8302.5 to 21553.3) |
|  | Anxiety disorders | Females | 22197.9 (14582.8 to 35563.7) | 191286.8 (138614 to 269177.4) | 22935.6 (14105.3 to 36155.3) |
|  | Anxiety disorders | Both sexes | 35587 (23756.5 to 56832) | 305118.5 (223922.3 to 430138.2) | 36753.4 (22526.3 to 57741.7) |
| Mauritius | Anxiety disorders | Males | 1117.6 (877 to 1482.7) | 10395.5 (8072.8 to 12739.7) | 1223.2 (826.6 to 1757.5) |
|  | Anxiety disorders | Females | 1788.3 (1383.8 to 2390.3) | 18502.7 (14097.4 to 22704.7) | 2139 (1476.7 to 3176.2) |
|  | Anxiety disorders | Both sexes | 2905.9 (2264.5 to 3871.4) | 28898.2 (22173.5 to 35353.4) | 3362.2 (2314.2 to 4905.7) |
| Mexico | Anxiety disorders | Males | 320456.8 (231678.3 to 439513.4) | 3217915.6 (2383437.8 to 4061131) | 379333.9 (240931 to 575687.8) |
|  | Anxiety disorders | Females | 614665.5 (448743.7 to 868341.8) | 6492817.3 (4921545.6 to 8319590.4) | 753719.3 (496728.1 to 1116883.5) |
|  | Anxiety disorders | Both sexes | 935122.3 (679364.2 to 1312391.9) | 9710732.9 (7304983.4 to 12349513.7) | 1133053.2 (735390.4 to 1698105.7) |
| Micronesia (Federated States of) | Anxiety disorders | Males | 253 (165.2 to 410.7) | 2209.9 (1588.4 to 3350.6) | 264.9 (153.6 to 441.3) |
|  | Anxiety disorders | Females | 326 (215.7 to 492.6) | 2954 (2137.2 to 4173.4) | 351.3 (210.9 to 556.1) |
|  | Anxiety disorders | Both sexes | 578.9 (380.6 to 899.6) | 5163.9 (3726.7 to 7531.2) | 616.1 (363.5 to 990.5) |
| Monaco | Anxiety disorders | Males | 147 (120.2 to 187.9) | 1302.8 (1143.4 to 1500.3) | 153.7 (105.4 to 211.9) |
|  | Anxiety disorders | Females | 239.1 (193.3 to 303.7) | 2191.9 (1949 to 2541.9) | 255.4 (184.7 to 351.6) |
|  | Anxiety disorders | Both sexes | 386.1 (315.6 to 488.4) | 3494.7 (3100.2 to 4043.9) | 409.1 (291.1 to 566.5) |
| Mongolia | Anxiety disorders | Males | 6821 (4610.8 to 9911.3) | 58640.9 (41692.3 to 80732.5) | 7107.1 (4243.4 to 11313.5) |
|  | Anxiety disorders | Females | 9349.5 (6490.2 to 13039.9) | 87805.2 (63271 to 116669.2) | 10521.6 (6525.7 to 16312.3) |
|  | Anxiety disorders | Both sexes | 16170.5 (11093.4 to 23011) | 146446.1 (104397.8 to 195974.6) | 17628.8 (10918.7 to 27724.8) |
| Montenegro | Anxiety disorders | Males | 1850.7 (1390.1 to 2545.8) | 18236 (13488.8 to 23742.9) | 2150.6 (1384.4 to 3216.6) |
|  | Anxiety disorders | Females | 3287.7 (2513 to 4528) | 34870.6 (27025 to 44294.7) | 4063.1 (2724.9 to 5973.7) |
|  | Anxiety disorders | Both sexes | 5138.5 (3910.9 to 7051.4) | 53106.6 (40537.2 to 67714.6) | 6213.7 (4122.9 to 9223.6) |
| Morocco | Anxiety disorders | Males | 97124.4 (73749.9 to 129153.7) | 873808 (697975.1 to 1114848.5) | 104815.8 (67379.2 to 153036.5) |
|  | Anxiety disorders | Females | 123298.1 (93101.7 to 162901) | 1178876.5 (915932.7 to 1454900.1) | 138332.8 (93716.1 to 199774.9) |
|  | Anxiety disorders | Both sexes | 220422.5 (166528.6 to 292566.2) | 2052684.5 (1613837.5 to 2587574.5) | 243148.5 (161562 to 351877.9) |
| Myanmar | Anxiety disorders | Males | 78760.7 (60937 to 102736.3) | 647742.3 (501205.3 to 800564.6) | 77561.9 (50813.9 to 115471.9) |
|  | Anxiety disorders | Females | 129545.6 (99203.9 to 171380.6) | 1133197.3 (882867.3 to 1404403.4) | 133867 (90029.7 to 198127.4) |
|  | Anxiety disorders | Both sexes | 208306.3 (160140.9 to 273943.8) | 1780939.6 (1380468.8 to 2187319.4) | 211428.9 (140576 to 311353.5) |
| Namibia | Anxiety disorders | Males | 7455.3 (5167.2 to 10779.1) | 72410.7 (53479.5 to 100521.5) | 8718.4 (5401.2 to 13065.3) |
|  | Anxiety disorders | Females | 10854.6 (7610 to 15813.3) | 105711.3 (79795 to 139955.6) | 12562.8 (7759 to 19341.8) |
|  | Anxiety disorders | Both sexes | 18309.9 (12736.9 to 26738.9) | 178122 (133575 to 238671.7) | 21281.2 (13208.8 to 32506.2) |
| Nauru | Anxiety disorders | Males | 30.7 (19.5 to 52.7) | 260.7 (180 to 401.1) | 31.6 (17.8 to 54.8) |
|  | Anxiety disorders | Females | 39.4 (26.5 to 62) | 346.9 (252.9 to 497.3) | 41.6 (24.7 to 65.7) |
|  | Anxiety disorders | Both sexes | 70 (46.4 to 115.1) | 607.6 (433 to 901.2) | 73.2 (42.9 to 120.7) |
| Nepal | Anxiety disorders | Males | 75230.9 (55943.8 to 101891.4) | 670928.5 (516030.3 to 882103) | 80642.2 (52924.1 to 125316.1) |
|  | Anxiety disorders | Females | 95983.4 (71804.5 to 127716.7) | 911032.5 (710427.9 to 1197120.5) | 107877.6 (73069.4 to 165736.2) |
|  | Anxiety disorders | Both sexes | 171214.3 (127804.7 to 233548.4) | 1581960.9 (1226623.6 to 2079223.5) | 188519.8 (126009 to 291444.8) |
| Netherlands | Anxiety disorders | Males | 93655.6 (76727.3 to 117098.4) | 794095.3 (677595.1 to 925464.8) | 93978.4 (64790.9 to 131849.5) |
|  | Anxiety disorders | Females | 152857 (123652.7 to 194941.6) | 1353672.1 (1185673.2 to 1580026) | 156759.6 (111484.7 to 214361.7) |
|  | Anxiety disorders | Both sexes | 246512.6 (199627.5 to 311799.2) | 2147767.5 (1863268.3 to 2508721.2) | 250738.1 (176513.8 to 344662.7) |
| New Zealand | Anxiety disorders | Males | 23635.1 (16894.9 to 32367) | 175537.7 (129853.3 to 219356.1) | 20734.8 (13347.7 to 30410.8) |
|  | Anxiety disorders | Females | 44858 (32307.9 to 61706.8) | 346904.8 (264213.5 to 416366.5) | 40138.3 (26660.8 to 58196.2) |
|  | Anxiety disorders | Both sexes | 68493 (49217.2 to 94204.7) | 522442.6 (391959.4 to 632293.3) | 60873.1 (40010.1 to 88503.6) |
| Nicaragua | Anxiety disorders | Males | 18068.1 (13093.5 to 24986.6) | 166629.6 (126198 to 212275.8) | 19838.6 (12515 to 30020.3) |
|  | Anxiety disorders | Females | 29875.8 (21691.2 to 42385) | 300204.2 (233741.9 to 379710.7) | 35193 (23066 to 52600.9) |
|  | Anxiety disorders | Both sexes | 47943.9 (34726.2 to 67440.1) | 466833.9 (359960.8 to 590772.8) | 55031.6 (35782.8 to 82512.6) |
| Nigeria | Anxiety disorders | Males | 822575 (486180.9 to 1459171.9) | 6410257 (4238166 to 10270763.9) | 776858.2 (439745.8 to 1357026.1) |
|  | Anxiety disorders | Females | 1646975.3 (799940.3 to 3391397.6) | 12280361.6 (7356920.2 to 19877320.6) | 1468964.1 (757891.5 to 2621082.8) |
|  | Anxiety disorders | Both sexes | 2469550.3 (1277982.1 to 4894007.8) | 18690618.6 (11770793.1 to 29980335.8) | 2245822.3 (1205554.4 to 3931885.3) |
| North Macedonia | Anxiety disorders | Males | 4447.4 (3375.6 to 6089.3) | 43965.5 (33105.9 to 57513.2) | 5180.2 (3357.8 to 7716.8) |
|  | Anxiety disorders | Females | 7765.2 (5928.7 to 10725.9) | 83511.4 (65007.2 to 106966.6) | 9722.1 (6533.8 to 14205) |
|  | Anxiety disorders | Both sexes | 12212.7 (9332.4 to 16808.7) | 127476.9 (98116.6 to 164111.1) | 14902.3 (9961.9 to 21854.3) |
| Northern Mariana Islands | Anxiety disorders | Males | 116.4 (79.2 to 181.8) | 1035.1 (733.2 to 1521.7) | 124 (74 to 201.6) |
|  | Anxiety disorders | Females | 144.7 (100.2 to 214.8) | 1341.3 (952.8 to 1853) | 159.2 (98.9 to 249.4) |
|  | Anxiety disorders | Both sexes | 261 (179.9 to 398.6) | 2376.4 (1686 to 3372) | 283.2 (172.5 to 450.6) |
| Norway | Anxiety disorders | Males | 20775.3 (16061.1 to 26655.7) | 170400.2 (131908.7 to 210401.5) | 20214.2 (13111.4 to 28922.8) |
|  | Anxiety disorders | Females | 36794.9 (27220 to 50285) | 313980.1 (246416.3 to 385677.3) | 36712.3 (24510.2 to 51203.3) |
|  | Anxiety disorders | Both sexes | 57570.2 (43252.3 to 76494.4) | 484380.2 (378261.8 to 595908) | 56926.6 (37592 to 80549) |
| Oman | Anxiety disorders | Males | 15625.5 (11763.5 to 21325) | 151411.9 (122324.6 to 188871.1) | 18245.9 (12110.3 to 27194.3) |
|  | Anxiety disorders | Females | 12641.9 (9181 to 16809.4) | 127441.2 (100536.3 to 158325.5) | 15132.5 (10119.6 to 22069.3) |
|  | Anxiety disorders | Both sexes | 28267.4 (20971.8 to 38660.3) | 278853 (223630.4 to 342229.3) | 33378.4 (22157.5 to 49285.6) |
| Pakistan | Anxiety disorders | Males | 602819.1 (421613.9 to 860794.5) | 5258727 (3882957.9 to 7262462.8) | 632836.8 (403001.2 to 994915.9) |
|  | Anxiety disorders | Females | 650485.4 (463987.9 to 903479.7) | 6149001.5 (4665622.4 to 8328560.9) | 727884.1 (475156 to 1137092.9) |
|  | Anxiety disorders | Both sexes | 1253304.4 (885601.8 to 1780600.3) | 11407728.5 (8517989.2 to 15453223.9) | 1360720.8 (878550.5 to 2136087.3) |
| Palau | Anxiety disorders | Males | 43 (30.4 to 64.2) | 389.1 (279 to 540.1) | 46 (29.1 to 73.5) |
|  | Anxiety disorders | Females | 50.8 (36.5 to 75.1) | 477.9 (339.6 to 653.4) | 56 (36.2 to 86.2) |
|  | Anxiety disorders | Both sexes | 93.9 (66.8 to 138.8) | 867 (618.6 to 1190.2) | 102 (65.2 to 158) |
| Palestine | Anxiety disorders | Males | 16866 (12257 to 23271.2) | 157165.3 (122387.2 to 212328.3) | 18983 (12267.1 to 29177.5) |
|  | Anxiety disorders | Females | 18797.2 (13532.5 to 25586.8) | 188526.4 (146086 to 243520) | 22324 (14724.9 to 33047.3) |
|  | Anxiety disorders | Both sexes | 35663.2 (26095.3 to 48932.9) | 345691.7 (265351.4 to 455110.5) | 41307 (26992 to 61812) |
| Panama | Anxiety disorders | Males | 9282.1 (6997.1 to 12550.9) | 83023.9 (62260.5 to 104209.4) | 9821.5 (6410.9 to 14887.7) |
|  | Anxiety disorders | Females | 14261 (10652 to 19782.3) | 141529.5 (108132.1 to 177387) | 16545.8 (11112.6 to 24612.5) |
|  | Anxiety disorders | Both sexes | 23543.1 (17630 to 32259) | 224553.4 (169739.3 to 281330.4) | 26367.3 (17460.8 to 39577.1) |
| Papua New Guinea | Anxiety disorders | Males | 28222.2 (17875.7 to 48441.7) | 218354.5 (152665.9 to 338619.3) | 26418 (15018.6 to 43634.1) |
|  | Anxiety disorders | Females | 34023.8 (23014.4 to 54058.7) | 274587.1 (200785.4 to 392327.8) | 32878 (19652.9 to 52165.2) |
|  | Anxiety disorders | Both sexes | 62245.9 (40964.6 to 103079.5) | 492941.6 (353733.4 to 732317.5) | 59296 (34828.7 to 94757) |
| Paraguay | Anxiety disorders | Males | 22887.3 (17189.5 to 31263.2) | 215640.4 (163915.1 to 267889.9) | 25567.6 (16885.1 to 37848.3) |
|  | Anxiety disorders | Females | 38600.1 (28676.2 to 53872.3) | 378958.6 (293057 to 467378.5) | 44325.8 (29992.1 to 66192.8) |
|  | Anxiety disorders | Both sexes | 61487.4 (45865.8 to 85335.6) | 594598.9 (457099.1 to 735268.3) | 69893.4 (46647 to 104435.6) |
| Peru | Anxiety disorders | Males | 139216.9 (100830.7 to 189514.8) | 1402144.2 (1048502.3 to 1775173.4) | 167088.9 (108669.2 to 244889.5) |
|  | Anxiety disorders | Females | 230299.9 (165683.1 to 313446.4) | 2354844.7 (1793438.1 to 2877425.1) | 276385.9 (181636.9 to 405344.1) |
|  | Anxiety disorders | Both sexes | 369516.8 (266079.6 to 503069) | 3756989 (2842463.6 to 4607837.8) | 443474.8 (289926.3 to 645600.9) |
| Philippines | Anxiety disorders | Males | 165022.6 (125769.1 to 216718) | 1447985.1 (1128340.4 to 1795965) | 175217.3 (114100 to 256058.9) |
|  | Anxiety disorders | Females | 238204.6 (179700.3 to 317307.5) | 2197310.9 (1697818.7 to 2723281.4) | 261503.2 (172918.8 to 385297) |
|  | Anxiety disorders | Both sexes | 403227.2 (305311.2 to 533246.7) | 3645296 (2826204.9 to 4460105) | 436720.5 (287286.6 to 638372.1) |
| Poland | Anxiety disorders | Males | 54975.6 (41721.7 to 74647.9) | 564105.7 (420890 to 728540.3) | 66170.7 (42695.4 to 98686.8) |
|  | Anxiety disorders | Females | 107896.2 (82041.6 to 147730.2) | 1235733.3 (985030.5 to 1566622.5) | 142615.4 (96996 to 210270.3) |
|  | Anxiety disorders | Both sexes | 162871.8 (124050.9 to 223141.4) | 1799839 (1405544.6 to 2282219.2) | 208786.1 (140024.2 to 309662.3) |
| Portugal | Anxiety disorders | Males | 60625.6 (46328.4 to 78532.6) | 521436.9 (407529.8 to 634182.2) | 61402.6 (40206.8 to 87797.6) |
|  | Anxiety disorders | Females | 95098.6 (68316.1 to 127687.5) | 879140.6 (696688.9 to 1078069.6) | 102002.4 (69195.4 to 144051.1) |
|  | Anxiety disorders | Both sexes | 155724.2 (114243.8 to 203843.4) | 1400577.5 (1103065 to 1710720.3) | 163405 (109441 to 231848.7) |
| Puerto Rico | Anxiety disorders | Males | 10727.9 (8197 to 15017.7) | 100590.3 (77699 to 127245.3) | 11608.1 (7845.7 to 16909.9) |
|  | Anxiety disorders | Females | 19060.2 (14584.5 to 25784.7) | 191124 (148203.8 to 230748.8) | 21825.8 (14919.7 to 31693.6) |
|  | Anxiety disorders | Both sexes | 29788 (22945 to 40998.3) | 291714.3 (225036.1 to 356810.8) | 33433.9 (22737.4 to 48347.9) |
| Qatar | Anxiety disorders | Males | 7353.1 (5459 to 9935.1) | 67668.2 (53670.9 to 84791.3) | 8134.1 (5309.6 to 11939.1) |
|  | Anxiety disorders | Females | 4717.9 (3321.9 to 6768.5) | 43661.8 (33769.9 to 56558.5) | 5172.4 (3314.8 to 7465.2) |
|  | Anxiety disorders | Both sexes | 12071 (8889 to 16423.3) | 111330 (87443.5 to 138114.6) | 13306.4 (8747.9 to 19330.4) |
| Republic of Korea | Anxiety disorders | Males | 106918.1 (81641.3 to 143515) | 885230.7 (674613.1 to 1129410.5) | 105424.6 (67704.8 to 158500) |
|  | Anxiety disorders | Females | 180755.8 (137219.5 to 239450.2) | 1669382.9 (1326866.4 to 2047530.7) | 196172.6 (130411.6 to 279275.1) |
|  | Anxiety disorders | Both sexes | 287673.9 (218846.2 to 382749) | 2554613.6 (2003369.9 to 3223765.7) | 301597.2 (197168.7 to 442779.9) |
| Republic of Moldova | Anxiety disorders | Males | 7495.9 (5606.4 to 10431.7) | 77263.4 (57524.1 to 100815.6) | 9157.8 (5904 to 13548.4) |
|  | Anxiety disorders | Females | 12106.8 (9173.5 to 16817.4) | 132463.9 (103701 to 168485) | 15376.8 (10146.5 to 22898.1) |
|  | Anxiety disorders | Both sexes | 19602.8 (14990.1 to 27247.1) | 209727.3 (163529 to 266459.1) | 24534.6 (15992 to 36741.8) |
| Romania | Anxiety disorders | Males | 37222.7 (28207.5 to 53146.5) | 380424.5 (282222.3 to 498238.6) | 44681.1 (28569.4 to 66353.8) |
|  | Anxiety disorders | Females | 70347.7 (53193.2 to 99999.9) | 780410.8 (620992.9 to 1005672.2) | 90721.2 (61064.6 to 134621.6) |
|  | Anxiety disorders | Both sexes | 107570.4 (81826.4 to 153146.4) | 1160835.4 (903124.3 to 1507899.3) | 135402.4 (90413.1 to 200585.2) |
| Russian Federation | Anxiety disorders | Males | 196852.2 (147040.4 to 276525.9) | 1941919 (1428021.9 to 2542566.8) | 229143.1 (147027.3 to 346775.8) |
|  | Anxiety disorders | Females | 388141.6 (294742.9 to 536636.6) | 4177217 (3252101.8 to 5380678.1) | 482856.7 (318757.8 to 717571.6) |
|  | Anxiety disorders | Both sexes | 584993.9 (446960.4 to 808889.7) | 6119136 (4745314.1 to 7931544) | 711999.8 (466872.4 to 1066756.3) |
| Saint Kitts and Nevis | Anxiety disorders | Males | 138.6 (104.2 to 186.5) | 1310.1 (979 to 1618.3) | 154.1 (100.9 to 230) |
|  | Anxiety disorders | Females | 225.7 (166.9 to 312.8) | 2325.1 (1777.9 to 2895.1) | 270.7 (180.5 to 406.6) |
|  | Anxiety disorders | Both sexes | 364.3 (271.1 to 498.2) | 3635.1 (2756.9 to 4513.6) | 424.8 (281 to 638.7) |
| Saint Lucia | Anxiety disorders | Males | 537.5 (409.2 to 726.3) | 5233.9 (3957.3 to 6433.3) | 613.4 (405.6 to 919.8) |
|  | Anxiety disorders | Females | 812.3 (611.1 to 1120) | 8594 (6554.9 to 10598.9) | 994.1 (666 to 1494.2) |
|  | Anxiety disorders | Both sexes | 1349.8 (1020.6 to 1846.1) | 13827.8 (10463.4 to 17063.8) | 1607.6 (1072.2 to 2413.2) |
| Saint Vincent and the Grenadines | Anxiety disorders | Males | 359.2 (274.2 to 483.1) | 3239.8 (2449.8 to 4053.1) | 380.1 (249.7 to 552.8) |
|  | Anxiety disorders | Females | 514.9 (383.3 to 702.3) | 5004.9 (3797.8 to 6151.7) | 580.3 (385 to 867.2) |
|  | Anxiety disorders | Both sexes | 874 (657.5 to 1180.1) | 8244.7 (6220.8 to 10039) | 960.4 (636.8 to 1418.6) |
| Samoa | Anxiety disorders | Males | 570.1 (359.5 to 1000.9) | 4662.6 (3193.8 to 7402.8) | 564.5 (314.4 to 986.1) |
|  | Anxiety disorders | Females | 703.5 (464 to 1129) | 6048.5 (4365 to 8837.4) | 723.7 (425.7 to 1155.1) |
|  | Anxiety disorders | Both sexes | 1273.7 (830.7 to 2130) | 10711.1 (7644 to 16252.5) | 1288.2 (744 to 2081.6) |
| San Marino | Anxiety disorders | Males | 136.1 (111.6 to 173.3) | 1263.8 (1107.1 to 1442.5) | 149.2 (103.7 to 207) |
|  | Anxiety disorders | Females | 217.3 (175.8 to 273.9) | 2088.4 (1831.4 to 2384.4) | 243.6 (175.4 to 338.2) |
|  | Anxiety disorders | Both sexes | 353.4 (288 to 441.1) | 3352.1 (2938.9 to 3822.1) | 392.8 (277.3 to 544.7) |
| Sao Tome and Principe | Anxiety disorders | Males | 844.4 (593.3 to 1256.6) | 7618.9 (5709.6 to 10452.7) | 924.4 (571.2 to 1397.6) |
|  | Anxiety disorders | Females | 1263.7 (855 to 1955.8) | 11687.2 (8716.4 to 15825.1) | 1405.6 (890.2 to 2160.8) |
|  | Anxiety disorders | Both sexes | 2108.1 (1453 to 3222.6) | 19306.2 (14539.7 to 26368.6) | 2330 (1464.9 to 3528.6) |
| Saudi Arabia | Anxiety disorders | Males | 113958 (79557.9 to 161055.4) | 1109134.3 (838104.8 to 1408572.8) | 132938.9 (85184.1 to 199010.8) |
|  | Anxiety disorders | Females | 125436.3 (75045.8 to 196559.8) | 1197039.9 (835522.2 to 1625142.7) | 142197.9 (83973.6 to 224480.9) |
|  | Anxiety disorders | Both sexes | 239394.2 (156488.6 to 354525.1) | 2306174.2 (1708335.1 to 3011607.4) | 275136.8 (173647.7 to 422739) |
| Senegal | Anxiety disorders | Males | 62228.1 (42326.8 to 97449.9) | 543993.2 (399090.9 to 779159.4) | 65944.4 (39159.1 to 103678.1) |
|  | Anxiety disorders | Females | 94618.5 (62889.5 to 150113.2) | 830867.6 (609913.1 to 1148283.6) | 99158.2 (60617 to 155922) |
|  | Anxiety disorders | Both sexes | 156846.6 (105620.9 to 248947.6) | 1374860.7 (1017903.1 to 1937009.2) | 165102.6 (99816.6 to 258888.9) |
| Serbia | Anxiety disorders | Males | 21166 (16091.3 to 28777.2) | 225829.4 (168230 to 288056.6) | 26610.3 (17125.6 to 39939.3) |
|  | Anxiety disorders | Females | 38947.9 (29496.9 to 53877.1) | 437845 (344164.4 to 551168.6) | 51019.4 (34610 to 73566.1) |
|  | Anxiety disorders | Both sexes | 60114 (45586.1 to 82982.4) | 663674.4 (512394.4 to 839700.8) | 77629.7 (51885.9 to 113102.8) |
| Seychelles | Anxiety disorders | Males | 149 (116.8 to 196.7) | 1326 (1032.2 to 1621.8) | 158.7 (106 to 238.8) |
|  | Anxiety disorders | Females | 190.8 (148.5 to 250.8) | 1788.3 (1391.6 to 2186.1) | 210.3 (141.5 to 307.3) |
|  | Anxiety disorders | Both sexes | 339.8 (266.2 to 444.9) | 3114.4 (2448.7 to 3804.9) | 369 (246.2 to 545.8) |
| Singapore | Anxiety disorders | Males | 8541.6 (6408.1 to 11476.7) | 69379.9 (51422 to 88635.1) | 8296.4 (5325.9 to 12292) |
|  | Anxiety disorders | Females | 10895.3 (8085.6 to 14707.9) | 105206.8 (79977.7 to 131008.9) | 12489.3 (8181.8 to 18025.9) |
|  | Anxiety disorders | Both sexes | 19436.9 (14506 to 26057.8) | 174586.7 (131467.2 to 220193.3) | 20785.8 (13534.4 to 30126.7) |
| Slovakia | Anxiety disorders | Males | 10312.6 (7779.8 to 14129) | 101170 (75308.2 to 130485.4) | 11888.2 (7739.5 to 17913.8) |
|  | Anxiety disorders | Females | 19665.9 (15001.3 to 27127.2) | 210454.5 (167272.5 to 268023) | 24395.9 (16620.7 to 35028.6) |
|  | Anxiety disorders | Both sexes | 29978.5 (22900.8 to 41410) | 311624.5 (242659.1 to 397719.4) | 36284.1 (24241.7 to 52829.3) |
| Slovenia | Anxiety disorders | Males | 5544.4 (4201.1 to 7571.4) | 52407.4 (40387.9 to 67752) | 6118.8 (4004.8 to 9119.4) |
|  | Anxiety disorders | Females | 11017.5 (8311.5 to 15136.1) | 108749.2 (88247.4 to 138709.8) | 12520.8 (8471.7 to 18129.9) |
|  | Anxiety disorders | Both sexes | 16561.8 (12516.3 to 22799.5) | 161156.6 (129099.1 to 205610.3) | 18639.6 (12567.4 to 27167.3) |
| Solomon Islands | Anxiety disorders | Males | 2163.1 (1380.5 to 3698.3) | 17484.4 (12215.6 to 27169.5) | 2122.1 (1221.7 to 3617.6) |
|  | Anxiety disorders | Females | 2690.5 (1803.2 to 4241.9) | 22648.6 (16525.5 to 32793.5) | 2722.1 (1628.7 to 4302.8) |
|  | Anxiety disorders | Both sexes | 4853.6 (3228.1 to 7966.8) | 40133 (28881.2 to 60129.3) | 4844.1 (2886.3 to 7885) |
| South Africa | Anxiety disorders | Males | 174220.8 (120900 to 241761.8) | 1721446.9 (1283161.4 to 2254123.9) | 204970 (128977 to 312641.4) |
|  | Anxiety disorders | Females | 254804 (180891.7 to 361759.3) | 2588408.1 (1950176.7 to 3437876.7) | 301681.1 (197534.6 to 453446) |
|  | Anxiety disorders | Both sexes | 429024.8 (300793.2 to 603188.3) | 4309855 (3233446.8 to 5701013.2) | 506651.1 (326734.6 to 770258.2) |
| Spain | Anxiety disorders | Males | 182051.3 (139004.1 to 237859.6) | 1592317.6 (1218999.5 to 1960975.2) | 188228.6 (124092.6 to 269567) |
|  | Anxiety disorders | Females | 250477.5 (181091.3 to 337334.7) | 2469237.8 (1886906.3 to 3047632.3) | 287085.3 (192107 to 419686.6) |
|  | Anxiety disorders | Both sexes | 432528.8 (321135.3 to 574077.9) | 4061555.3 (3105957.2 to 4993125.8) | 475313.9 (317993.8 to 684259.7) |
| Sri Lanka | Anxiety disorders | Males | 25524.6 (20039.6 to 33487.3) | 197192.3 (157375.1 to 238578.6) | 23440 (15414 to 34195.9) |
|  | Anxiety disorders | Females | 40974.6 (31715.8 to 54384.6) | 342700.7 (266599.8 to 416904.1) | 40233.2 (26840.5 to 58971.7) |
|  | Anxiety disorders | Both sexes | 66499.2 (51794.6 to 87922.9) | 539893 (425922.2 to 651271.5) | 63673.1 (42364.3 to 93075.2) |
| Suriname | Anxiety disorders | Males | 1685.4 (1240.2 to 2256.5) | 16101.4 (11942.7 to 20571.3) | 1904.2 (1222.5 to 2827.6) |
|  | Anxiety disorders | Females | 2537.2 (1858.6 to 3466.3) | 25965 (19637.1 to 32153) | 3009.4 (1973.4 to 4492.1) |
|  | Anxiety disorders | Both sexes | 4222.7 (3098.4 to 5719.1) | 42066.3 (31584.5 to 51700.9) | 4913.7 (3201 to 7320.9) |
| Sweden | Anxiety disorders | Males | 33192 (24989.5 to 43568.9) | 274802 (205933.2 to 346586.7) | 32447.7 (21291.4 to 47825.6) |
|  | Anxiety disorders | Females | 56275.2 (45760.8 to 71153) | 476104.6 (414355.3 to 550393.4) | 55915.7 (40417.9 to 77173.3) |
|  | Anxiety disorders | Both sexes | 89467.2 (70690.9 to 113805.6) | 750906.6 (618177.7 to 887686.7) | 88363.4 (61303.7 to 122732.3) |
| Switzerland | Anxiety disorders | Males | 28889 (22023.6 to 37005.2) | 271049.8 (211327.8 to 328984.4) | 32010.1 (21644.1 to 45847.7) |
|  | Anxiety disorders | Females | 44624.5 (31831.5 to 61880.9) | 453707.3 (358783 to 554017.3) | 52868.9 (35250.8 to 75419.8) |
|  | Anxiety disorders | Both sexes | 73513.5 (53995.2 to 97314) | 724757.1 (576561.7 to 879027.7) | 84879 (56951.9 to 120066.7) |
| Taiwan | Anxiety disorders | Males | 40997.5 (30427 to 60477.4) | 328426.4 (251643.9 to 427296.7) | 38767.4 (25873.9 to 59724.1) |
|  | Anxiety disorders | Females | 65964.7 (46561.3 to 100194.5) | 565507.4 (437000.6 to 729620.6) | 66301.6 (45224.3 to 100128.5) |
|  | Anxiety disorders | Both sexes | 106962.2 (77047.2 to 161331.5) | 893933.8 (687767.3 to 1154960.8) | 105069.1 (71943.9 to 159416.4) |
| Tajikistan | Anxiety disorders | Males | 17072.3 (11572.4 to 25473) | 155127 (108207.5 to 220411) | 18880.3 (10987.5 to 30501.3) |
|  | Anxiety disorders | Females | 26312.3 (18020.9 to 38135.8) | 250005.1 (177032.8 to 344151) | 30147.2 (18351.5 to 48845.6) |
|  | Anxiety disorders | Both sexes | 43384.6 (29605.6 to 63825.4) | 405132.2 (286325.8 to 560414.8) | 49027.5 (29191.2 to 78867.6) |
| Thailand | Anxiety disorders | Males | 76151.3 (57503.9 to 107986.6) | 596859.2 (452499.7 to 782277.8) | 70376.4 (46475.7 to 107625.2) |
|  | Anxiety disorders | Females | 134796.7 (102082 to 186145.5) | 1099374.9 (836673.5 to 1393613) | 128068.7 (85555.3 to 190338.5) |
|  | Anxiety disorders | Both sexes | 210948 (159808.9 to 295227.5) | 1696234.1 (1297371.6 to 2172137.3) | 198445 (132761.4 to 291373.4) |
| Timor-Leste | Anxiety disorders | Males | 1834.7 (1413.3 to 2409.6) | 14083.2 (11010.7 to 17744.9) | 1706.4 (1089.4 to 2551.6) |
|  | Anxiety disorders | Females | 2664.3 (2009.7 to 3543) | 21362.2 (16693.9 to 26076.6) | 2560.1 (1666.1 to 3781.2) |
|  | Anxiety disorders | Both sexes | 4499 (3425.9 to 5934.4) | 35445.4 (27704.6 to 43949.2) | 4266.5 (2755.1 to 6301.5) |
| Tonga | Anxiety disorders | Males | 337.8 (185.5 to 732.9) | 2791.4 (1695.7 to 5122.5) | 338.6 (177.2 to 713.6) |
|  | Anxiety disorders | Females | 503.2 (288.6 to 1055.3) | 4319.1 (2793.5 to 7413.2) | 518 (285.7 to 973.5) |
|  | Anxiety disorders | Both sexes | 840.9 (475.6 to 1758.6) | 7110.6 (4481.5 to 12584.6) | 856.7 (465.9 to 1683.6) |
| Trinidad and Tobago | Anxiety disorders | Males | 3794.5 (2884 to 5125.7) | 35856.9 (26842.4 to 44207.2) | 4196 (2740.3 to 6278.4) |
|  | Anxiety disorders | Females | 5501.2 (4150.9 to 7483.7) | 56524.2 (42773.4 to 69861.9) | 6514.3 (4353.1 to 9736.2) |
|  | Anxiety disorders | Both sexes | 9295.7 (7042.1 to 12562.2) | 92381 (69479.3 to 113984.2) | 10710.3 (7101.4 to 16014.6) |
| Tunisia | Anxiety disorders | Males | 39893.9 (30407.4 to 53484.2) | 379066.9 (302967.8 to 473161) | 45141.8 (29728.5 to 65689.5) |
|  | Anxiety disorders | Females | 43837.3 (33433.5 to 58060.6) | 467519.1 (372670.6 to 571652.9) | 54607.6 (36959.1 to 79235.5) |
|  | Anxiety disorders | Both sexes | 83731.2 (63640.5 to 111965.9) | 846586.1 (681041.6 to 1039654.2) | 99749.4 (66687.6 to 143266.9) |
| T眉rkiye | Anxiety disorders | Males | 198196.4 (145040.2 to 275181.7) | 1898561.6 (1414393.7 to 2452723) | 227615.2 (145964.9 to 339027.9) |
|  | Anxiety disorders | Females | 236863.1 (172906.3 to 311973.4) | 2477354.1 (1837024 to 3061846.8) | 288579.9 (189170.5 to 420637.6) |
|  | Anxiety disorders | Both sexes | 435059.4 (317686.2 to 585491.3) | 4375915.6 (3247434.1 to 5497056.8) | 516195.2 (335494.6 to 769711.7) |
| Turkmenistan | Anxiety disorders | Males | 10150.4 (7125.1 to 14411.4) | 91191.2 (63899.7 to 126189.6) | 11027 (6642.7 to 17289.5) |
|  | Anxiety disorders | Females | 13459.8 (9660.1 to 18669.7) | 125519.3 (89993.6 to 166815.2) | 15000.8 (9554.5 to 23351) |
|  | Anxiety disorders | Both sexes | 23610.2 (16785.3 to 33191.5) | 216710.5 (152926 to 292266.8) | 26027.8 (16435.7 to 40660.1) |
| Tuvalu | Anxiety disorders | Males | 27.7 (18.1 to 45.6) | 230.7 (165 to 349) | 27.8 (16.6 to 45.9) |
|  | Anxiety disorders | Females | 34.2 (22.9 to 52.1) | 299.9 (215.2 to 421.4) | 35.7 (21.6 to 56.5) |
|  | Anxiety disorders | Both sexes | 61.9 (40.8 to 97.7) | 530.6 (380.2 to 770.8) | 63.5 (38.3 to 102.4) |
| Ukraine | Anxiety disorders | Males | 57583.1 (42659.4 to 82965.2) | 600406.8 (437353 to 790714.6) | 70726.9 (45308.2 to 107193) |
|  | Anxiety disorders | Females | 119468.6 (90709 to 165136.4) | 1291989.1 (1020445.8 to 1657625.2) | 148626 (97743.6 to 219013.9) |
|  | Anxiety disorders | Both sexes | 177051.6 (135734.4 to 249389.2) | 1892395.9 (1470810.4 to 2479386.8) | 219352.9 (144869.6 to 325728.6) |
| United Arab Emirates | Anxiety disorders | Males | 26336.2 (19043.3 to 37000.7) | 252875.4 (203833.8 to 322880.8) | 30027 (20318.2 to 43214.3) |
|  | Anxiety disorders | Females | 17618.3 (11168.9 to 26245.5) | 167834.5 (126183.3 to 219934.4) | 19727.6 (12432.5 to 29663.7) |
|  | Anxiety disorders | Both sexes | 43954.5 (31200.2 to 61736.8) | 420709.8 (331347.2 to 525492.2) | 49754.6 (32763.6 to 73112.9) |
| United Kingdom | Anxiety disorders | Males | 323013.1 (253199.6 to 424764.5) | 2740928.9 (2218184.2 to 3348680.5) | 321888.5 (214405.1 to 459066.3) |
|  | Anxiety disorders | Females | 608605.2 (482798.4 to 746651.1) | 5042991.3 (4281704.2 to 5909910.2) | 582451.6 (409457.7 to 814741.3) |
|  | Anxiety disorders | Both sexes | 931618.4 (736856.2 to 1168523.3) | 7783920.2 (6504888.3 to 9244527.2) | 904340 (624261.9 to 1264789.4) |
| United Republic of Tanzania | Anxiety disorders | Males | 120108.8 (87752.4 to 155043.7) | 1070815.3 (836304.9 to 1339685.6) | 128928.2 (81976.4 to 188841.2) |
|  | Anxiety disorders | Females | 159192.1 (120197.9 to 207250.2) | 1462091.1 (1167900.1 to 1809190.8) | 173564.5 (115023.4 to 251505.8) |
|  | Anxiety disorders | Both sexes | 279301 (208512.9 to 362989.9) | 2532906.4 (2006041.2 to 3139798.8) | 302492.7 (195490.2 to 440267.9) |
| United States of America | Anxiety disorders | Males | 1044042.4 (798290.1 to 1348979.6) | 8921916.8 (6856920.8 to 10824412.5) | 1032288.1 (682685.4 to 1491229.5) |
|  | Anxiety disorders | Females | 1959992.4 (1457765.2 to 2541592.1) | 16533118.8 (12882901.6 to 19786591.6) | 1870889.4 (1259027.7 to 2724534.1) |
|  | Anxiety disorders | Both sexes | 3004034.8 (2258223.1 to 3878633.5) | 25455035.6 (19854272.9 to 30361642) | 2903177.5 (1933671.4 to 4215763.6) |
| United States Virgin Islands | Anxiety disorders | Males | 246.8 (190.2 to 346) | 2321.3 (1792.8 to 2936.9) | 268.9 (179 to 389.8) |
|  | Anxiety disorders | Females | 378.8 (288.6 to 522.8) | 3939.3 (2995.5 to 4816.8) | 452.8 (311.4 to 674.3) |
|  | Anxiety disorders | Both sexes | 625.6 (475.2 to 872.7) | 6260.6 (4788.3 to 7705.5) | 721.7 (493.6 to 1052.2) |
| Uruguay | Anxiety disorders | Males | 11992.4 (8769.3 to 16204.2) | 106440.5 (78863.1 to 134759.1) | 12685.9 (8147 to 19169.7) |
|  | Anxiety disorders | Females | 21064.6 (15359.3 to 28350.2) | 204414.6 (157579.6 to 249962.8) | 23897.6 (15679.2 to 34983.3) |
|  | Anxiety disorders | Both sexes | 33057 (24169.1 to 44489) | 310855.2 (236260.1 to 387151) | 36583.5 (23840.7 to 53884.3) |
| Uzbekistan | Anxiety disorders | Males | 38330.8 (26873.3 to 54665.4) | 320534.6 (227541.6 to 435122.4) | 38822.2 (23578.7 to 61069.8) |
|  | Anxiety disorders | Females | 70598.4 (50374.5 to 99001.2) | 622187.6 (443923.9 to 811946.3) | 74355 (45528.7 to 114325.9) |
|  | Anxiety disorders | Both sexes | 108929.1 (77196.1 to 152840.4) | 942722.2 (671465.5 to 1244180.5) | 113177.1 (69615 to 173996) |
| Vanuatu | Anxiety disorders | Males | 825.2 (526.1 to 1418.1) | 6616 (4646.6 to 10298) | 802.7 (451.6 to 1374.3) |
|  | Anxiety disorders | Females | 1053.2 (705 to 1655.3) | 8849.5 (6454 to 12631.2) | 1064.3 (642 to 1684.8) |
|  | Anxiety disorders | Both sexes | 1878.4 (1246.7 to 3083.2) | 15465.5 (11223.5 to 22991.2) | 1867 (1104 to 3035.2) |
| Viet Nam | Anxiety disorders | Males | 100593.4 (74327.2 to 133100.6) | 828086 (632438.7 to 1035997) | 99345.3 (65319.3 to 146507.7) |
|  | Anxiety disorders | Females | 169078.5 (128880.6 to 225814.4) | 1506714.1 (1163135.7 to 1855150.7) | 178376.1 (119798.8 to 258566.8) |
|  | Anxiety disorders | Both sexes | 269672 (203599.3 to 359944.6) | 2334800.1 (1795574.4 to 2864827.5) | 277721.4 (184853.2 to 402844) |
| Zambia | Anxiety disorders | Males | 72975.2 (52249.2 to 95553.9) | 619627.2 (473286.4 to 784828.1) | 74465.3 (47812.8 to 108484.8) |
|  | Anxiety disorders | Females | 76877.2 (57157 to 99898.5) | 691127 (550070.4 to 852300.4) | 82042.4 (53236.9 to 119510.4) |
|  | Anxiety disorders | Both sexes | 149852.4 (109005.1 to 193605.6) | 1310754.2 (1023889 to 1631773.1) | 156507.7 (100750.8 to 229052.9) |
| Zimbabwe | Anxiety disorders | Males | 36867.1 (24887.7 to 55198.5) | 355417.7 (262211.9 to 489008.5) | 42943 (25684.5 to 66419.2) |
|  | Anxiety disorders | Females | 56526.2 (37775.9 to 91154) | 548271.5 (400259.8 to 797756.4) | 65147.5 (38243.9 to 102764.9) |
|  | Anxiety disorders | Both sexes | 93393.3 (63007 to 148197.5) | 903689.1 (664040.6 to 1283688.6) | 108090.6 (63928.4 to 168776.3) |

**Table S5 Age-standardized incidence, mortality and DALYs rate of anxiety disorder in 2023**

| Locations | Cause | Sex | Incidence.number..95.UI | Prevalence.number..95.UI | DALYs.number..95.UI |
| --- | --- | --- | --- | --- | --- |
| World Bank High Income | Anxiety disorders | Males | 617.1（449.0 to 814.4） | 5,008.6 (3,753.2 to 6,515.7) | 598.3 (386.1 to 884.7) |
|  | Anxiety disorders | Females | 1,059.0 (751.8 to 1,404.9) | 8,710.7 (6,689.0 to 10,915.0) | 1,026.6 (668.2 to 1,486.8) |
|  | Anxiety disorders | Both sexes | 834.0 (598.4 to 1,102.2) | 6,833.5 (5,197.8 to 8,686.8) | 809.1 (523.8 to 1,180.0) |
| World Bank Upper Middle Income | Anxiety disorders | Males | 555.2 (390.3 to 821.8) | 4,161.1 (3,148.2 to 5,572.0) | 498.4 (318.4 to 751.5) |
|  | Anxiety disorders | Females | 834.5 (592.46 to 1,182.33) | 6,621.1 (5,116.8 to 8,426.6) | 782.4 (505.1 to 1,160.2) |
|  | Anxiety disorders | Both sexes | 692.3 (489.9 to 992.0) | 5,374.5 (4,118.2 to 6,969.6) | 638.3 (410.9 to 944.6) |
| World Bank Lower Middle Income | Anxiety disorders | Males | 490.6 (355.1 to 682.6) | 4,283.5 (3,275.0 to 5,605.4) | 510.6 (330.9 to 753.9) |
|  | Anxiety disorders | Females | 776.2 (566.5 to 1,099.4) | 6,850.3 (5,350.8 to 8,822.7) | 801.7 (525.6 to 1,176.5) |
|  | Anxiety disorders | Both sexes | 632.2 (460.1 to 891.2) | 5,562.3 (4,308.7 to 7,208.0) | 655.4 (425.7 to 963.4) |
| Albania | Anxiety disorders | Males | 438.7 (304.8 to 646.6) | 4134.8 (3002.4 to 5660.7) | 497.1 (296.9 to 743.2) |
|  | Anxiety disorders | Females | 837.1 (584.7 to 1228.2) | 8265.8 (6212.6 to 11100.1) | 984.6 (617.3 to 1469.8) |
|  | Anxiety disorders | Both sexes | 637.3 (445.7 to 935.3) | 6208.2 (4610.9 to 8480.9) | 741.4 (454.6 to 1097.1) |
| Algeria | Anxiety disorders | Males | 506.5 (381.8 to 673.6) | 4652 (3659.1 to 5937.3) | 558.1 (362.8 to 824.5) |
|  | Anxiety disorders | Females | 505.6 (379.5 to 662.6) | 5207.3 (4125.8 to 6456.9) | 612.4 (414.9 to 886.4) |
|  | Anxiety disorders | Both sexes | 506.1 (381.3 to 668.4) | 4929.2 (3948.1 to 6222.9) | 585.2 (392 to 850.6) |
| American Samoa | Anxiety disorders | Males | 475.5 (318.3 to 762.2) | 4133.7 (2979.1 to 6162.4) | 493.2 (293.8 to 795) |
|  | Anxiety disorders | Females | 635.4 (431.5 to 959.2) | 5758.2 (4184.5 to 8069.9) | 683.3 (417.8 to 1079.4) |
|  | Anxiety disorders | Both sexes | 554.2 (374.1 to 864.5) | 4935.4 (3574.5 to 7123.5) | 586.9 (357.3 to 936.5) |
| Andorra | Anxiety disorders | Males | 907.3 (711.9 to 1174.3) | 7416 (6363.4 to 8974.6) | 897.6 (612.1 to 1287.5) |
|  | Anxiety disorders | Females | 1664.2 (1342.8 to 2095.7) | 12960.1 (11323.6 to 14733.1) | 1547.2 (1102 to 2180.9) |
|  | Anxiety disorders | Both sexes | 1275.2 (1024.7 to 1600.3) | 10081.4 (8741.6 to 11655.8) | 1210 (847.4 to 1690.4) |
| Angola | Anxiety disorders | Males | 797.1 (614.1 to 1029.7) | 7359 (5928.2 to 8973.1) | 873.5 (582.1 to 1268.6) |
|  | Anxiety disorders | Females | 856.5 (655.2 to 1127.7) | 8439.9 (6745.1 to 10131.6) | 985.6 (652.3 to 1407.6) |
|  | Anxiety disorders | Both sexes | 828.6 (635.8 to 1079.5) | 7934.8 (6369.7 to 9579.2) | 933.2 (619.7 to 1345.4) |
| Antigua and Barbuda | Anxiety disorders | Males | 594.8 (439.1 to 807.4) | 5370.4 (4045.3 to 6924.4) | 637.1 (410 to 971.9) |
|  | Anxiety disorders | Females | 887.6 (639 to 1215) | 8574 (6561.2 to 10659.4) | 1002.8 (654.1 to 1487.5) |
|  | Anxiety disorders | Both sexes | 742.7 (540.5 to 1011.7) | 7004.2 (5331 to 8778.4) | 823.4 (533.9 to 1226.3) |
| Argentina | Anxiety disorders | Males | 793.5 (563.9 to 1089.3) | 6862.3 (5154.9 to 9163.6) | 822.6 (516.6 to 1213.3) |
|  | Anxiety disorders | Females | 1292.5 (933.6 to 1767.9) | 11793.5 (8968.3 to 15576) | 1395.2 (880.1 to 2077.8) |
|  | Anxiety disorders | Both sexes | 1047.1 (753.1 to 1430.2) | 9388.1 (7165 to 12442.5) | 1115.5 (705.4 to 1660.5) |
| Armenia | Anxiety disorders | Males | 624 (456.9 to 868.5) | 5866.9 (4250.1 to 7841.6) | 703 (430.2 to 1072.9) |
|  | Anxiety disorders | Females | 839.1 (615.6 to 1163.2) | 8429.4 (6214.3 to 10991.6) | 1003.1 (634 to 1506.1) |
|  | Anxiety disorders | Both sexes | 733.3 (539.1 to 1013.5) | 7187.7 (5274 to 9528.4) | 857 (537.1 to 1292.6) |
| Australia | Anxiety disorders | Males | 1393.6 (1063.4 to 1757.4) | 9112.1 (7723 to 11049) | 1091.4 (730.3 to 1547.7) |
|  | Anxiety disorders | Females | 2051.9 (1642.4 to 2545.7) | 13785.8 (11817.8 to 15733.5) | 1618 (1106.7 to 2187.4) |
|  | Anxiety disorders | Both sexes | 1720.7 (1350.2 to 2139.2) | 11462.8 (9790.5 to 13496.4) | 1356.2 (913.4 to 1848) |
| Austria | Anxiety disorders | Males | 732.8 (500.3 to 1054.3) | 5899.2 (4269.8 to 8129.6) | 710.9 (441.7 to 1052.9) |
|  | Anxiety disorders | Females | 1247.1 (762.9 to 1960.7) | 10131.7 (7180 to 13314) | 1210.8 (758.8 to 1819) |
|  | Anxiety disorders | Both sexes | 983.5 (628.4 to 1482.8) | 7976.1 (5763.6 to 10710.6) | 956 (594.8 to 1448.3) |
| Azerbaijan | Anxiety disorders | Males | 360.2 (263.7 to 501.3) | 3499.7 (2526.7 to 4698.6) | 420.4 (260.7 to 641.4) |
|  | Anxiety disorders | Females | 381.9 (280.2 to 529.5) | 4378.4 (3201 to 5794.9) | 520.2 (324.1 to 798.9) |
|  | Anxiety disorders | Both sexes | 371 (272.1 to 513.4) | 3943.2 (2869.9 to 5291.8) | 470.5 (291.7 to 713.4) |
| Bahamas | Anxiety disorders | Males | 594.8 (439.1 to 807.4) | 5633.9 (4239.9 to 7243) | 668 (431.5 to 1000.4) |
|  | Anxiety disorders | Females | 887.6 (639 to 1215) | 8962.3 (6897.9 to 11142.9) | 1049.8 (683.2 to 1568.6) |
|  | Anxiety disorders | Both sexes | 745.8 (542.8 to 1016) | 7365.5 (5623.4 to 9228.4) | 866.4 (564.3 to 1282.6) |
| Bahrain | Anxiety disorders | Males | 484.3 (365.1 to 644.1) | 4792.5 (3783.1 to 6113.8) | 573.2 (372.8 to 847.9) |
|  | Anxiety disorders | Females | 623.7 (468.2 to 817.4) | 6574.1 (5202.9 to 8148.1) | 769.7 (518.9 to 1113.2) |
|  | Anxiety disorders | Both sexes | 539.9 (406.6 to 717.7) | 5453.8 (4336.2 to 6886.9) | 646.2 (427.5 to 942) |
| Bangladesh | Anxiety disorders | Males | 479.1 (340.2 to 676) | 4286.4 (3129.4 to 5697.3) | 512.5 (320 to 780.8) |
|  | Anxiety disorders | Females | 588.7 (416.9 to 821.1) | 5536.8 (4087.6 to 7234.4) | 648.5 (418.7 to 979.4) |
|  | Anxiety disorders | Both sexes | 534.4 (378.7 to 746.3) | 4916.2 (3614.5 to 6458.9) | 581.1 (368.7 to 880.9) |
| Barbados | Anxiety disorders | Males | 594.8 (439.1 to 807.4) | 5333.3 (4018.2 to 6847.8) | 634.6 (410.8 to 941.7) |
|  | Anxiety disorders | Females | 887.6 (639 to 1215) | 8519.4 (6524.2 to 10590.8) | 999.4 (652.9 to 1491.2) |
|  | Anxiety disorders | Both sexes | 743.7 (541.4 to 1012.9) | 6973.1 (5308.6 to 8736.7) | 822.1 (535.9 to 1220.1) |
| Belarus | Anxiety disorders | Males | 316.5 (225.9 to 472.8) | 2916.3 (2096.1 to 3977.1) | 350.1 (212.3 to 536.3) |
|  | Anxiety disorders | Females | 524.9 (376.5 to 763.3) | 5068.9 (3727.8 to 6824.6) | 603.9 (372.2 to 937.8) |
|  | Anxiety disorders | Both sexes | 423.2 (304.8 to 616.5) | 4029.1 (2937.6 to 5494.9) | 480.8 (295.2 to 737.8) |
| Belgium | Anxiety disorders | Males | 792.2 (540.3 to 1095.7) | 6559.6 (4725.1 to 8736.3) | 788.6 (486.3 to 1201.7) |
|  | Anxiety disorders | Females | 1167.3 (750.7 to 1737.1) | 10072.7 (7191.2 to 13644.3) | 1193.6 (736.2 to 1790.5) |
|  | Anxiety disorders | Both sexes | 976.8 (637.3 to 1426.7) | 8302.7 (5950.5 to 11188.7) | 989.3 (605.9 to 1491.9) |
| Belize | Anxiety disorders | Males | 607 (448.1 to 823.9) | 5769.7 (4334.5 to 7432.3) | 685.2 (436.9 to 1017.6) |
|  | Anxiety disorders | Females | 856.5 (616.6 to 1172.5) | 8464 (6455.3 to 10614.7) | 990.5 (652.5 to 1479.9) |
|  | Anxiety disorders | Both sexes | 734.8 (534.4 to 1001.9) | 7160 (5432.8 to 8927.6) | 842.7 (550.7 to 1253.3) |
| Benin | Anxiety disorders | Males | 819.1 (602.6 to 1155.5) | 6478.1 (5005.6 to 8540) | 775 (501.8 to 1135.2) |
|  | Anxiety disorders | Females | 1290.9 (907 to 1905.3) | 10420.8 (8134.1 to 13699.6) | 1232.3 (800.6 to 1875.4) |
|  | Anxiety disorders | Both sexes | 1057.2 (760.8 to 1534) | 8495 (6734.6 to 10996.7) | 1008.7 (650.9 to 1498.8) |
| Bermuda | Anxiety disorders | Males | 594.8 (439.1 to 807.4) | 5307.3 (3999.1 to 6814.7) | 632.5 (408.4 to 953) |
|  | Anxiety disorders | Females | 887.6 (639 to 1215) | 8481 (6492.8 to 10529.8) | 998.6 (653.9 to 1485.7) |
|  | Anxiety disorders | Both sexes | 742.4 (540.5 to 1011.2) | 6917.1 (5264.1 to 8666) | 818.1 (532.9 to 1209.6) |
| Bhutan | Anxiety disorders | Males | 478.9 (355.9 to 649.5) | 3719.7 (2910.6 to 4765.8) | 447.4 (294.2 to 689.8) |
|  | Anxiety disorders | Females | 587.2 (438.2 to 802) | 4927.1 (3878.9 to 6310.3) | 577.9 (391.7 to 871.9) |
|  | Anxiety disorders | Both sexes | 531.1 (395.5 to 723.5) | 4300 (3375.9 to 5504.8) | 510.1 (345.7 to 775.5) |
| Bolivia (Plurinational State of) | Anxiety disorders | Males | 825.2 (603.8 to 1110.8) | 7995.8 (5907.8 to 10216.1) | 950.4 (604.9 to 1419.3) |
|  | Anxiety disorders | Females | 1201.8 (855.9 to 1640.3) | 12201.6 (9089.3 to 14997) | 1430.5 (918.7 to 2095.2) |
|  | Anxiety disorders | Both sexes | 1014.5 (731.1 to 1370.3) | 10127.9 (7523.7 to 12624.4) | 1193.5 (764.6 to 1752.4) |
| Bosnia and Herzegovina | Anxiety disorders | Males | 461.1 (320.5 to 679.7) | 4478.2 (3274.4 to 6094.2) | 534.7 (328.4 to 801) |
|  | Anxiety disorders | Females | 820 (572.7 to 1203) | 8445.1 (6396.1 to 11307.7) | 1002.1 (629.9 to 1491.1) |
|  | Anxiety disorders | Both sexes | 640.4 (448.2 to 941.7) | 6472.2 (4840.5 to 8814.1) | 769.2 (480.7 to 1146.7) |
| Botswana | Anxiety disorders | Males | 510.8 (361.2 to 708) | 4824.7 (3526.2 to 6324.9) | 572.8 (360.9 to 851) |
|  | Anxiety disorders | Females | 657.7 (473 to 914.8) | 6317.8 (4727.6 to 8233.3) | 740.1 (471.3 to 1108.4) |
|  | Anxiety disorders | Both sexes | 585.9 (415.1 to 812) | 5593.4 (4142.3 to 7252.9) | 658.8 (418.8 to 980.5) |
| Brazil | Anxiety disorders | Males | 906.1 (689.1 to 1214.5) | 8084.3 (6235.8 to 9897.9) | 953.4 (630 to 1398.2) |
|  | Anxiety disorders | Females | 1644.8 (1224 to 2262.1) | 14885.3 (11687.1 to 18125.6) | 1729.2 (1175.9 to 2528.4) |
|  | Anxiety disorders | Both sexes | 1282 (962.5 to 1743.3) | 11577 (9003.3 to 14103.2) | 1351.3 (907.6 to 1980.7) |
| Brunei Darussalam | Anxiety disorders | Males | 445.4 (311.7 to 670.3) | 3385.2 (2419.1 to 4986.7) | 409.4 (238.8 to 666.8) |
|  | Anxiety disorders | Females | 619.5 (421.5 to 885.6) | 5124.8 (3689.8 to 7126.9) | 613.6 (373.9 to 944.7) |
|  | Anxiety disorders | Both sexes | 527.4 (363.7 to 766.5) | 4203.8 (3012.5 to 5964.5) | 505.4 (303.4 to 798.7) |
| Bulgaria | Anxiety disorders | Males | 306.2 (211.7 to 463.1) | 3007.7 (2214.5 to 4274.3) | 360.3 (221 to 550) |
|  | Anxiety disorders | Females | 631.7 (438.6 to 946.2) | 6532.1 (4926.4 to 8923.9) | 777.4 (488.4 to 1154.2) |
|  | Anxiety disorders | Both sexes | 467.2 (325.6 to 694) | 4761 (3559.6 to 6585.1) | 567.3 (355.6 to 845.2) |
| Cabo Verde | Anxiety disorders | Males | 721.8 (531 to 1018.1) | 6536.5 (5006.8 to 8535.6) | 786.2 (505.2 to 1163.6) |
|  | Anxiety disorders | Females | 1057.8 (743.2 to 1561.2) | 9781.2 (7572.3 to 12973.2) | 1165.4 (753.6 to 1777.9) |
|  | Anxiety disorders | Both sexes | 887.2 (638.4 to 1287.6) | 8132 (6382.7 to 10613.9) | 972.5 (624.4 to 1461.8) |
| Cambodia | Anxiety disorders | Males | 368.3 (287.5 to 478.8) | 2916.8 (2326.2 to 3539.4) | 349.6 (228.8 to 500.6) |
|  | Anxiety disorders | Females | 564.6 (435.7 to 744.5) | 4735.4 (3721.2 to 5748.5) | 558.8 (370.5 to 801.4) |
|  | Anxiety disorders | Both sexes | 469.9 (364 to 614.5) | 3867.2 (3062.7 to 4634.6) | 458.7 (302.4 to 651.2) |
| Cameroon | Anxiety disorders | Males | 738.7 (543.4 to 1042) | 6657.2 (5133.4 to 8699.3) | 795.5 (512.3 to 1176.9) |
|  | Anxiety disorders | Females | 1020.4 (716.9 to 1506.1) | 9496.3 (7354.9 to 12590.1) | 1120.4 (723.3 to 1693.8) |
|  | Anxiety disorders | Both sexes | 878.5 (632.4 to 1274.5) | 8080.7 (6387.3 to 10505.9) | 958.3 (610.7 to 1433.2) |
| Canada | Anxiety disorders | Males | 723.5 (532.1 to 1031.4) | 5573.1 (4284.6 to 7660.3) | 665.7 (415.3 to 1017.9) |
|  | Anxiety disorders | Females | 1132.6 (823.3 to 1547.6) | 9218.6 (7018.1 to 12192.3) | 1080.9 (690.9 to 1630) |
|  | Anxiety disorders | Both sexes | 926.7 (679.1 to 1285.6) | 7394.7 (5664.1 to 9916) | 872.9 (554.8 to 1316.6) |
| Chile | Anxiety disorders | Males | 797.3 (584.8 to 1083.5) | 7005.6 (5183 to 9117.9) | 840.6 (535.8 to 1266) |
|  | Anxiety disorders | Females | 1378.4 (999.5 to 1924.3) | 12621 (9577.7 to 16575.3) | 1493.9 (975.4 to 2236) |
|  | Anxiety disorders | Both sexes | 1085.9 (793 to 1523.7) | 9812.2 (7396.8 to 12878.2) | 1166.7 (757.1 to 1740.4) |
| China | Anxiety disorders | Males | 558.9 (378.2 to 916.3) | 3320.1 (2451.7 to 4879.1) | 400.8 (246.2 to 645.9) |
|  | Anxiety disorders | Females | 787.8 (540.5 to 1265) | 4812 (3588.4 to 6802.1) | 576.7 (365.4 to 918.5) |
|  | Anxiety disorders | Both sexes | 668.9 (454.3 to 1070.6) | 4041.3 (3006.4 to 5801.6) | 485.7 (303.9 to 769.6) |
| Colombia | Anxiety disorders | Males | 468.5 (352.3 to 629.7) | 4294.7 (3289.4 to 5358.4) | 509.5 (335.1 to 764.3) |
|  | Anxiety disorders | Females | 840.5 (624.2 to 1150.9) | 8082.2 (6358.9 to 10045.3) | 946.1 (631 to 1394) |
|  | Anxiety disorders | Both sexes | 655.6 (489.8 to 888.6) | 6217.5 (4846.3 to 7734) | 730.8 (488.5 to 1081.3) |
| Comoros | Anxiety disorders | Males | 617.8 (469.9 to 787.4) | 5380.1 (4082.4 to 6464.3) | 645.3 (427.1 to 940) |
|  | Anxiety disorders | Females | 687.1 (523 to 895.6) | 6295.3 (4905.4 to 7598.8) | 741.9 (496.7 to 1092.1) |
|  | Anxiety disorders | Both sexes | 652.4 (496.4 to 841.2) | 5837.3 (4488.4 to 7007.2) | 693.5 (461.2 to 1012.9) |
| Congo | Anxiety disorders | Males | 681.3 (524.9 to 880.1) | 6038.3 (4862.2 to 7366.5) | 716.4 (466.3 to 1039.2) |
|  | Anxiety disorders | Females | 796.2 (609 to 1048.3) | 7531 (5993.5 to 9040.3) | 877.4 (582.7 to 1265) |
|  | Anxiety disorders | Both sexes | 739.3 (567.1 to 963.2) | 6794.6 (5442.2 to 8211) | 798 (524.6 to 1164.6) |
| Costa Rica | Anxiety disorders | Males | 527.9 (397.5 to 713.7) | 4600.4 (3446.3 to 5743.9) | 543.9 (355.7 to 811.9) |
|  | Anxiety disorders | Females | 833.5 (616.5 to 1157.2) | 7780.9 (5951.3 to 9713.9) | 907.8 (600.4 to 1351.6) |
|  | Anxiety disorders | Both sexes | 679.6 (505.8 to 931) | 6183.6 (4684.9 to 7707.4) | 724.9 (476.4 to 1083.4) |
| Côte d'Ivoire | Anxiety disorders | Males | 767.1 (564.3 to 1082) | 6411 (4939.9 to 8350.9) | 767.3 (489.5 to 1124) |
|  | Anxiety disorders | Females | 1150.9 (808.6 to 1698.7) | 9857.8 (7635.8 to 13087) | 1160.8 (752.1 to 1753.9) |
|  | Anxiety disorders | Both sexes | 949.8 (683.5 to 1379.3) | 8050.3 (6354.2 to 10471.6) | 954.6 (614.8 to 1434.2) |
| Croatia | Anxiety disorders | Males | 623.1 (433 to 918.4) | 5483.3 (3992.7 to 7426.8) | 655.9 (398.8 to 989.8) |
|  | Anxiety disorders | Females | 1053.2 (735.6 to 1545.2) | 9839.8 (7463.8 to 13067) | 1170 (732.6 to 1748.6) |
|  | Anxiety disorders | Both sexes | 838.2 (586.6 to 1232.6) | 7675.7 (5735 to 10382.2) | 914 (567.2 to 1370.5) |
| Cuba | Anxiety disorders | Males | 600.2 (443.1 to 814.7) | 5667.4 (4319.5 to 7251.7) | 675.2 (433.5 to 998.5) |
|  | Anxiety disorders | Females | 899.2 (647.4 to 1231) | 9186.9 (7097.6 to 11340.2) | 1079.6 (699.7 to 1596.6) |
|  | Anxiety disorders | Both sexes | 747.2 (544.1 to 1017) | 7411.7 (5694.5 to 9263.6) | 875.4 (566.5 to 1286.5) |
| Cyprus | Anxiety disorders | Males | 974.1 (764.3 to 1260.7) | 8013.1 (6897.3 to 9674.9) | 968.8 (658.8 to 1402.8) |
|  | Anxiety disorders | Females | 1977.7 (1595.4 to 2498.2) | 15038.3 (13215.5 to 17024.2) | 1797.7 (1287.2 to 2500.4) |
|  | Anxiety disorders | Both sexes | 1460.8 (1174.3 to 1839.7) | 11466.3 (9969.5 to 13174.2) | 1375.8 (967.7 to 1934.3) |
| Czechia | Anxiety disorders | Males | 357.2 (246.2 to 540.1) | 3299.2 (2419.7 to 4669.6) | 394.6 (240 to 607) |
|  | Anxiety disorders | Females | 757.8 (520.1 to 1123.4) | 7740 (5901.1 to 10386.8) | 915.5 (565.4 to 1353.2) |
|  | Anxiety disorders | Both sexes | 555.5 (381.6 to 820.9) | 5507.6 (4147.2 to 7507.9) | 653.1 (402.3 to 965.2) |
| Denmark | Anxiety disorders | Males | 761.5 (597.5 to 985.6) | 5555 (4774.7 to 6731.5) | 669.7 (450.2 to 979.5) |
|  | Anxiety disorders | Females | 1452.1 (1171.7 to 1828.6) | 10318.5 (8957.8 to 11757.9) | 1233.2 (887.9 to 1746.1) |
|  | Anxiety disorders | Both sexes | 1099.2 (883.6 to 1380.5) | 7899.8 (6819.7 to 9153.2) | 946.9 (661.4 to 1331) |
| Djibouti | Anxiety disorders | Males | 541.5 (411.9 to 690.2) | 4626.2 (3510 to 5573.3) | 555.1 (364 to 805.8) |
|  | Anxiety disorders | Females | 598.4 (455.4 to 779.9) | 5233.3 (4052 to 6399.5) | 617.5 (416.4 to 931.7) |
|  | Anxiety disorders | Both sexes | 567.9 (432 to 731.7) | 4907.4 (3755.3 to 5923.7) | 584 (389.5 to 862.5) |
| Dominica | Anxiety disorders | Males | 594.8 (439.1 to 807.4) | 5383.7 (4054.5 to 6942.2) | 639.9 (412.5 to 969.3) |
|  | Anxiety disorders | Females | 887.6 (639 to 1215) | 8593.7 (6572.5 to 10653.1) | 1005.1 (657.3 to 1506.7) |
|  | Anxiety disorders | Both sexes | 737.1 (536.5 to 1004) | 6947.1 (5284 to 8729) | 817.7 (533.8 to 1236.4) |
| Dominican Republic | Anxiety disorders | Males | 654.6 (483.2 to 888.5) | 5888.9 (4449.9 to 7552) | 698.2 (449.7 to 1053.3) |
|  | Anxiety disorders | Females | 1076 (774.7 to 1473) | 10147.9 (7800.4 to 12544.2) | 1185.6 (769.3 to 1759.1) |
|  | Anxiety disorders | Both sexes | 867.9 (631.3 to 1182.2) | 8047.9 (6148.2 to 10052) | 945.2 (611 to 1387) |
| Ecuador | Anxiety disorders | Males | 735.6 (538.2 to 990) | 6972.9 (5142.1 to 8954.3) | 832.1 (529 to 1261.3) |
|  | Anxiety disorders | Females | 1061.1 (755.7 to 1448.2) | 10559.1 (7833.4 to 13017.9) | 1244 (791.5 to 1832.8) |
|  | Anxiety disorders | Both sexes | 902.6 (650.1 to 1220.4) | 8833.4 (6541.8 to 11017) | 1045.6 (665 to 1551.4) |
| Egypt | Anxiety disorders | Males | 544.6 (377.4 to 814.1) | 5232.3 (3774.2 to 7134.6) | 624 (375.2 to 940.1) |
|  | Anxiety disorders | Females | 683.9 (472.3 to 986.2) | 7050.7 (5212.2 to 9359) | 826.1 (523.8 to 1234.5) |
|  | Anxiety disorders | Both sexes | 611.7 (418.4 to 903.1) | 6109.9 (4469.6 to 8195.4) | 721.5 (446.9 to 1068.2) |
| El Salvador | Anxiety disorders | Males | 466 (350.9 to 630) | 4430.9 (3341.1 to 5515.6) | 521.6 (338.8 to 773.5) |
|  | Anxiety disorders | Females | 759.2 (561.6 to 1054) | 7652.9 (5906.7 to 9524.8) | 896.9 (598.9 to 1326.7) |
|  | Anxiety disorders | Both sexes | 621.4 (462.8 to 852.8) | 6179.8 (4736.5 to 7673.6) | 724.9 (480.5 to 1078.8) |
| Equatorial Guinea | Anxiety disorders | Males | 695.4 (535.7 to 898.3) | 6294.6 (5068.9 to 7670.1) | 746.5 (497 to 1086.9) |
|  | Anxiety disorders | Females | 791 (605.1 to 1041.5) | 7573.2 (6027.9 to 9082.5) | 886.4 (586.6 to 1265.9) |
|  | Anxiety disorders | Both sexes | 741.7 (569.6 to 964.2) | 6917.3 (5546.9 to 8345) | 814.2 (539.9 to 1177.6) |
| Estonia | Anxiety disorders | Males | 360.9 (257.6 to 539.4) | 3274.5 (2352.8 to 4465.7) | 393.2 (239.8 to 602.9) |
|  | Anxiety disorders | Females | 542.8 (389.3 to 789.2) | 5324.2 (3929.6 to 7142.4) | 635.2 (389.5 to 963.9) |
|  | Anxiety disorders | Both sexes | 451.9 (324.9 to 660.4) | 4306.3 (3146.6 to 5884.5) | 514.6 (316.1 to 782.6) |
| Eswatini | Anxiety disorders | Males | 464.9 (328.7 to 644.4) | 4782.8 (3492.1 to 6223) | 566.6 (354.8 to 836.2) |
|  | Anxiety disorders | Females | 671 (482.5 to 933.3) | 6929.9 (5209.5 to 9021.3) | 808 (515.9 to 1227.4) |
|  | Anxiety disorders | Both sexes | 570.1 (404.6 to 790) | 5885.8 (4368.1 to 7651) | 690.4 (436.9 to 1024.5) |
| Fiji | Anxiety disorders | Males | 475.5 (318.3 to 762.2) | 4078.1 (2928.1 to 6082.9) | 485.8 (291.5 to 793.2) |
|  | Anxiety disorders | Females | 635.4 (431.5 to 959.2) | 5683 (4114.5 to 7939.7) | 669.8 (412.2 to 1046.4) |
|  | Anxiety disorders | Both sexes | 554.7 (374.6 to 865.5) | 4876.8 (3517 to 7021.1) | 577.2 (352.1 to 926.5) |
| Finland | Anxiety disorders | Males | 640.2 (439.4 to 886.3) | 4771.5 (3485.8 to 6475.3) | 572 (346.9 to 861.4) |
|  | Anxiety disorders | Females | 732.2 (464.5 to 1129.4) | 5906.2 (4168.4 to 8275.1) | 703.5 (421.7 to 1070.6) |
|  | Anxiety disorders | Both sexes | 684.8 (450.4 to 1013.9) | 5325.8 (3819.9 to 7257.6) | 636.2 (381.5 to 960.7) |
| France | Anxiety disorders | Males | 852.1 (586.5 to 1165.6) | 6782.3 (4915.8 to 9065.3) | 813 (512.9 to 1223.9) |
|  | Anxiety disorders | Females | 1434 (937.8 to 2107.9) | 12174.8 (8887.9 to 16195.8) | 1444.2 (917.8 to 2124.7) |
|  | Anxiety disorders | Both sexes | 1140.7 (753.3 to 1627.1) | 9491.1 (6917.3 to 12628) | 1129.7 (716.5 to 1674.6) |
| Gabon | Anxiety disorders | Males | 708.6 (545.9 to 915.4) | 6327.1 (5094.8 to 7733.2) | 752.8 (491.7 to 1082.9) |
|  | Anxiety disorders | Females | 799.2 (611.3 to 1052.3) | 7581.3 (6056.4 to 9080.2) | 882 (593 to 1265.9) |
|  | Anxiety disorders | Both sexes | 755.6 (579.7 to 985.3) | 6980.3 (5597.1 to 8427.2) | 820.1 (545 to 1189) |
| Georgia | Anxiety disorders | Males | 324.5 (237.6 to 451.6) | 3078.5 (2209.3 to 4145.4) | 368.5 (226.9 to 565.5) |
|  | Anxiety disorders | Females | 510.6 (374.6 to 707.8) | 5170.7 (3781.7 to 6860.2) | 613.1 (389.1 to 938.3) |
|  | Anxiety disorders | Both sexes | 417.9 (307.8 to 577.7) | 4136.2 (3020.6 to 5532.7) | 491.6 (309.5 to 751.2) |
| Germany | Anxiety disorders | Males | 614.3 (469.9 to 797.9) | 4784.8 (3940.6 to 5860.5) | 576.8 (388.8 to 855.1) |
|  | Anxiety disorders | Females | 1055.6 (818.8 to 1378.3) | 8027.5 (6829.1 to 9639.1) | 958.8 (655.2 to 1401.3) |
|  | Anxiety disorders | Both sexes | 829.1 (641.3 to 1073.1) | 6369.6 (5353.7 to 7701.1) | 763.2 (510.2 to 1128.8) |
| Ghana | Anxiety disorders | Males | 505.2 (371.6 to 712.6) | 4438.8 (3413.2 to 5842) | 531.1 (337.6 to 781.8) |
|  | Anxiety disorders | Females | 770.7 (541.5 to 1137.6) | 7160.9 (5501.3 to 9648.8) | 846.9 (535.6 to 1300.5) |
|  | Anxiety disorders | Both sexes | 638.4 (459.6 to 926.2) | 5818.3 (4560 to 7633.3) | 691 (440 to 1050.4) |
| Greece | Anxiety disorders | Males | 920.3 (632.2 to 1255.4) | 7671.7 (5660.5 to 10050.3) | 922.2 (582.5 to 1343.3) |
|  | Anxiety disorders | Females | 1441.7 (936 to 2134.5) | 12323.8 (9076.6 to 16307.8) | 1460.8 (912.9 to 2133.7) |
|  | Anxiety disorders | Both sexes | 1175.2 (780.2 to 1705.5) | 9972.7 (7362.3 to 13171.5) | 1188.3 (749 to 1747) |
| Greenland | Anxiety disorders | Males | 754.1 (544.7 to 1040) | 6120.9 (4507.4 to 8087.6) | 728.4 (460.2 to 1099.1) |
|  | Anxiety disorders | Females | 1335.4 (949.8 to 1811) | 10868.5 (8249.5 to 13640.2) | 1269.8 (839 to 1876.6) |
|  | Anxiety disorders | Both sexes | 1031.6 (743.9 to 1394.9) | 8377.1 (6287.7 to 10807.8) | 985.7 (638.3 to 1467.7) |
| Grenada | Anxiety disorders | Males | 594.8 (439.1 to 807.4) | 5565.1 (4186.6 to 7148.7) | 658.5 (419.8 to 993.8) |
|  | Anxiety disorders | Females | 887.6 (639 to 1215) | 8861 (6781.4 to 11010.2) | 1037.9 (673.4 to 1540.7) |
|  | Anxiety disorders | Both sexes | 735.7 (535.5 to 1001.7) | 7156.9 (5441.2 to 8955.4) | 841.6 (547.2 to 1250.8) |
| Guam | Anxiety disorders | Males | 475.5 (318.3 to 762.2) | 4035.3 (2901.6 to 6027.6) | 485 (285.7 to 779.2) |
|  | Anxiety disorders | Females | 635.4 (431.5 to 959.2) | 5624.9 (4080.8 to 7878.8) | 671.7 (413 to 1053.6) |
|  | Anxiety disorders | Both sexes | 553.9 (373.9 to 864.2) | 4817.6 (3480.4 to 6939.3) | 576.8 (348.7 to 925.5) |
| Guatemala | Anxiety disorders | Males | 489 (368.3 to 661.2) | 4690.1 (3505.4 to 5853.8) | 549.7 (354.1 to 821.3) |
|  | Anxiety disorders | Females | 828.2 (612.6 to 1149.9) | 8286.4 (6326.7 to 10390.4) | 964.1 (637.4 to 1425.9) |
|  | Anxiety disorders | Both sexes | 665.1 (494.8 to 913.4) | 6577.2 (4985.8 to 8239) | 766.9 (501 to 1137.6) |
| Guinea | Anxiety disorders | Males | 851.2 (626.2 to 1200.7) | 7181.5 (5547.8 to 9332.6) | 859 (553.7 to 1247.8) |
|  | Anxiety disorders | Females | 1247.6 (876.6 to 1841.4) | 10726.9 (8340.4 to 14109.3) | 1266.9 (821.8 to 1914) |
|  | Anxiety disorders | Both sexes | 1051.9 (757.1 to 1525.2) | 9014.5 (7131.3 to 11645.5) | 1069.6 (693.1 to 1572.2) |
| Guyana | Anxiety disorders | Males | 592.9 (437.7 to 804.7) | 5543.7 (4172.9 to 7126.8) | 655.9 (423.8 to 981.5) |
|  | Anxiety disorders | Females | 881.6 (634.7 to 1206.8) | 8683.9 (6658.2 to 10828) | 1011.1 (656.1 to 1510.5) |
|  | Anxiety disorders | Both sexes | 738.8 (537.6 to 1006.3) | 7140.1 (5436.8 to 8931.6) | 836.3 (543.2 to 1249.1) |
| Haiti | Anxiety disorders | Males | 665.4 (491.2 to 903.1) | 5446.7 (4086.1 to 7067.3) | 644.5 (416.2 to 966.5) |
|  | Anxiety disorders | Females | 868.7 (625.5 to 1189.1) | 7761.4 (5933.5 to 9670.4) | 896.4 (586.9 to 1345.5) |
|  | Anxiety disorders | Both sexes | 770.1 (560.6 to 1048.9) | 6653 (5050 to 8370.4) | 775.8 (506.7 to 1165.3) |
| Honduras | Anxiety disorders | Males | 448.2 (337.6 to 606) | 4419.4 (3298.5 to 5529.4) | 522.4 (341.3 to 783.4) |
|  | Anxiety disorders | Females | 759.1 (561.5 to 1054) | 7938.5 (6063.7 to 9990.5) | 927.5 (614 to 1390.6) |
|  | Anxiety disorders | Both sexes | 609.2 (453 to 836.8) | 6261.9 (4747.4 to 7866.9) | 734.3 (481.5 to 1103.5) |
| Hungary | Anxiety disorders | Males | 321.8 (223.6 to 474.3) | 3180.8 (2322.7 to 4362.1) | 380.6 (231.5 to 568.3) |
|  | Anxiety disorders | Females | 665.9 (465.1 to 977) | 6979.4 (5283.5 to 9405.4) | 828.6 (516.4 to 1250.1) |
|  | Anxiety disorders | Both sexes | 494.4 (347 to 727) | 5098 (3812.2 to 6896.2) | 606.1 (379.1 to 907.1) |
| Iceland | Anxiety disorders | Males | 686.6 (538.7 to 888.7) | 5356.8 (4587.3 to 6544) | 645.2 (443.3 to 941.4) |
|  | Anxiety disorders | Females | 1391.4 (1122.8 to 1752.3) | 10539.7 (9179.2 to 12000) | 1253 (907.4 to 1747.1) |
|  | Anxiety disorders | Both sexes | 1028.4 (826.7 to 1290.6) | 7858 (6786.4 to 9143.9) | 938.5 (662 to 1302.4) |
| India | Anxiety disorders | Males | 486 (359.4 to 665.2) | 4220.6 (3288.7 to 5454.9) | 501.6 (327.8 to 729.5) |
|  | Anxiety disorders | Females | 850.2 (635.9 to 1169.8) | 7416.9 (5883.8 to 9452.2) | 863.3 (576.4 to 1247) |
|  | Anxiety disorders | Both sexes | 664.5 (495.3 to 911.4) | 5792.8 (4563.2 to 7413) | 679.2 (450.2 to 982.3) |
| Indonesia | Anxiety disorders | Males | 305 (234.1 to 402.3) | 2611.7 (1983.6 to 3253) | 313.3 (204.7 to 454) |
|  | Anxiety disorders | Females | 431.6 (323.7 to 571.9) | 3909.2 (3000.2 to 4777.9) | 462.4 (306.6 to 681.5) |
|  | Anxiety disorders | Both sexes | 367.5 (278.6 to 484.8) | 3255 (2485.4 to 3949.8) | 387.1 (255 to 565) |
| Iran (Islamic Republic of) | Anxiety disorders | Males | 1088.1 (785.5 to 1536.9) | 10245.2 (7881.3 to 13316.5) | 1221 (803.1 to 1809.1) |
|  | Anxiety disorders | Females | 1354.7 (990.3 to 1892.6) | 13717.2 (10876.7 to 17443) | 1604.7 (1056.5 to 2354.8) |
|  | Anxiety disorders | Both sexes | 1218.6 (886.9 to 1731.7) | 11952.2 (9352.5 to 15422.5) | 1409.5 (928 to 2073.8) |
| Iraq | Anxiety disorders | Males | 596.8 (417.3 to 856.4) | 5645.6 (4113.3 to 7654.5) | 670 (421.7 to 1008.9) |
|  | Anxiety disorders | Females | 967.7 (673.4 to 1378.6) | 9562.5 (7200.1 to 12459.9) | 1119.5 (712.5 to 1648.7) |
|  | Anxiety disorders | Both sexes | 777.8 (545.2 to 1129.8) | 7552.5 (5616.2 to 10038.1) | 888.7 (560.8 to 1315.8) |
| Ireland | Anxiety disorders | Males | 1017.6 (678.7 to 1591.2) | 8091 (5888.9 to 11517.3) | 975.2 (628 to 1483) |
|  | Anxiety disorders | Females | 1775.1 (1091 to 2770.2) | 13686.3 (10381.6 to 18036.4) | 1632.2 (1028.8 to 2393.8) |
|  | Anxiety disorders | Both sexes | 1390.2 (904.9 to 2187) | 10873 (8106 to 14762.3) | 1301.7 (833.6 to 1930.5) |
| Israel | Anxiety disorders | Males | 631.4 (442.7 to 842.8) | 5222.5 (3817.7 to 6949.2) | 629.1 (393.7 to 952.2) |
|  | Anxiety disorders | Females | 801.6 (502.3 to 1238.6) | 7157.7 (5025.1 to 9753) | 852.5 (527.6 to 1303.3) |
|  | Anxiety disorders | Both sexes | 713.9 (475.8 to 1038.8) | 6172.2 (4451 to 8356.2) | 738.6 (461 to 1119.4) |
| Italy | Anxiety disorders | Males | 768.8 (529 to 1052.8) | 6335.9 (4572.4 to 8514.3) | 764.3 (475.5 to 1154.7) |
|  | Anxiety disorders | Females | 1315.6 (857.1 to 1957) | 11259.9 (8143.9 to 14985.9) | 1342.4 (838.9 to 1998.8) |
|  | Anxiety disorders | Both sexes | 1036.5 (682.1 to 1480.5) | 8762.2 (6334 to 11718.9) | 1048.8 (654.1 to 1575.7) |
| Jamaica | Anxiety disorders | Males | 479.9 (354.2 to 651.3) | 4479.6 (3363.1 to 5781.4) | 533.7 (346 to 795.6) |
|  | Anxiety disorders | Females | 686.2 (494 to 939.4) | 7253.9 (5545.3 to 9065.1) | 849.4 (552 to 1270.1) |
|  | Anxiety disorders | Both sexes | 583.7 (424.8 to 795.2) | 5886 (4474.4 to 7425.3) | 693.6 (448.3 to 1030.1) |
| Japan | Anxiety disorders | Males | 373.2 (264.3 to 577) | 2706.2 (1952.5 to 4123.8) | 327.3 (192.8 to 538.8) |
|  | Anxiety disorders | Females | 508.5 (359 to 733.4) | 3967.5 (2916.9 to 5817.7) | 473.9 (284.8 to 760.1) |
|  | Anxiety disorders | Both sexes | 440 (315.4 to 655.6) | 3331.8 (2440.1 to 4983.6) | 399.9 (238.2 to 642.2) |
| Jordan | Anxiety disorders | Males | 513 (386.7 to 682.3) | 5045 (3977 to 6451.9) | 605.8 (397.1 to 893.9) |
|  | Anxiety disorders | Females | 634.9 (476.6 to 832.1) | 6684.7 (5247.4 to 8375.4) | 783.7 (532.8 to 1152.7) |
|  | Anxiety disorders | Both sexes | 569.5 (429 to 752.8) | 5794.4 (4626.9 to 7331.5) | 687 (460.5 to 1004.7) |
| Kazakhstan | Anxiety disorders | Males | 246.1 (180.2 to 342.5) | 2424.9 (1737.8 to 3270.5) | 290.8 (180 to 448.2) |
|  | Anxiety disorders | Females | 360.1 (264.2 to 499.1) | 3905.7 (2838.2 to 5195.7) | 464.3 (289.6 to 711.8) |
|  | Anxiety disorders | Both sexes | 304.3 (224 to 420.5) | 3186.8 (2311.9 to 4283.1) | 379.7 (237.9 to 579.8) |
| Kenya | Anxiety disorders | Males | 402.1 (304.7 to 518.1) | 3919 (3035.6 to 4722.9) | 468.3 (311.9 to 678.6) |
|  | Anxiety disorders | Females | 495.2 (380.5 to 650.2) | 4791.3 (3772.8 to 5782) | 564 (380.8 to 820.6) |
|  | Anxiety disorders | Both sexes | 449.5 (343 to 585.1) | 4363.2 (3410.3 to 5242.5) | 517 (346.7 to 748.5) |
| Kiribati | Anxiety disorders | Males | 475.5 (318.3 to 762.2) | 4089.4 (2936.8 to 6098.4) | 488.6 (296.9 to 795.7) |
|  | Anxiety disorders | Females | 635.4 (431.5 to 959.2) | 5698.2 (4128.5 to 7963.4) | 673.1 (412.9 to 1066.8) |
|  | Anxiety disorders | Both sexes | 558.1 (377.4 to 870.5) | 4929.7 (3555.1 to 7081.5) | 584.6 (358.1 to 932.8) |
| Kuwait | Anxiety disorders | Males | 475.5 (358.4 to 632.3) | 4366.3 (3457.6 to 5590.1) | 520.9 (336.3 to 778.6) |
|  | Anxiety disorders | Females | 629.1 (472.2 to 824.5) | 6095.9 (4763.7 to 7690.5) | 714.6 (479 to 1046.2) |
|  | Anxiety disorders | Both sexes | 537.1 (404.6 to 714) | 5026.6 (4004.8 to 6403.4) | 594.8 (393.6 to 880.5) |
| Kyrgyzstan | Anxiety disorders | Males | 218 (159.7 to 303.4) | 2179.7 (1563 to 2941.6) | 262.2 (161.2 to 408.8) |
|  | Anxiety disorders | Females | 361.4 (265.1 to 500.9) | 3822 (2781.9 to 5160.5) | 453.6 (280.9 to 696.3) |
|  | Anxiety disorders | Both sexes | 290.8 (214.2 to 402) | 3019.5 (2195.9 to 4061.5) | 359.8 (223.2 to 553.7) |
| Lao People's Democratic Republic | Anxiety disorders | Males | 263.7 (188.7 to 382.4) | 2008 (1471.8 to 2743.1) | 242 (150.4 to 364.1) |
|  | Anxiety disorders | Females | 432.1 (303.6 to 600.6) | 3298.9 (2414.5 to 4331.9) | 391.4 (250.8 to 597.8) |
|  | Anxiety disorders | Both sexes | 347.5 (246.1 to 485.2) | 2651.4 (1941.1 to 3534.7) | 316.4 (201.6 to 476.4) |
| Latvia | Anxiety disorders | Males | 414.4 (295.9 to 619.1) | 3777.6 (2703.7 to 5143.8) | 454.2 (281.4 to 699.5) |
|  | Anxiety disorders | Females | 641.6 (460.1 to 932.9) | 6293.4 (4641.4 to 8431.1) | 749 (461.3 to 1133.7) |
|  | Anxiety disorders | Both sexes | 528.8 (380.4 to 772.1) | 5055 (3698.9 to 6872.5) | 603.3 (375.7 to 915.5) |
| Lebanon | Anxiety disorders | Males | 739.1 (507.4 to 1108.8) | 6354.1 (4593.4 to 8640.1) | 757.4 (466 to 1146.4) |
|  | Anxiety disorders | Females | 1005.1 (685.6 to 1464.9) | 9505.9 (7144.6 to 12523.8) | 1115.6 (701.1 to 1661.3) |
|  | Anxiety disorders | Both sexes | 869.5 (597 to 1287.3) | 7920.3 (5868.5 to 10513.7) | 935 (582.9 to 1400.7) |
| Lesotho | Anxiety disorders | Males | 652.3 (461.3 to 904.2) | 5822.6 (4219.6 to 7576.8) | 688.8 (430.6 to 1023.7) |
|  | Anxiety disorders | Females | 741.1 (532.9 to 1030.8) | 6938.9 (5156.3 to 9052.8) | 809 (520 to 1232.9) |
|  | Anxiety disorders | Both sexes | 697.6 (493.6 to 967.3) | 6400.8 (4701.6 to 8344) | 751 (474.6 to 1128.3) |
| Libya | Anxiety disorders | Males | 511.3 (385.5 to 680) | 4972.5 (3923.1 to 6350.3) | 592.4 (383.2 to 878.5) |
|  | Anxiety disorders | Females | 714.2 (536.1 to 936) | 7267.4 (5725.8 to 9082.5) | 848.4 (574 to 1230.5) |
|  | Anxiety disorders | Both sexes | 610.2 (459.7 to 805) | 6088.4 (4868.8 to 7679.9) | 716.9 (475.3 to 1051.2) |
| Lithuania | Anxiety disorders | Males | 383.5 (272.9 to 576) | 3655.1 (2653.6 to 5016.7) | 438.9 (267.4 to 674.6) |
|  | Anxiety disorders | Females | 648.2 (459.6 to 962) | 6454.4 (4789.7 to 8683.8) | 770.5 (479.3 to 1194.8) |
|  | Anxiety disorders | Both sexes | 516.6 (368.8 to 764.2) | 5071.6 (3732.2 to 6928.9) | 606 (373.7 to 934.2) |
| Luxembourg | Anxiety disorders | Males | 767.1 (601.9 to 992.9) | 6196.9 (5295.2 to 7537) | 748.7 (514.1 to 1087.4) |
|  | Anxiety disorders | Females | 1252.6 (1010.7 to 1577.3) | 10107.8 (8781.4 to 11521.2) | 1207.5 (857.1 to 1724.1) |
|  | Anxiety disorders | Both sexes | 1003.3 (806 to 1260.2) | 8105.4 (6986.7 to 9391.7) | 972.5 (669.3 to 1378.7) |
| Malaysia | Anxiety disorders | Males | 213.3 (166.5 to 277.2) | 1795.9 (1426.7 to 2178.9) | 215.2 (141.5 to 311.6) |
|  | Anxiety disorders | Females | 323 (249.2 to 425.9) | 2875.8 (2252.6 to 3506.4) | 340 (229.3 to 499) |
|  | Anxiety disorders | Both sexes | 265.4 (205.8 to 346.7) | 2309.9 (1823.8 to 2765.2) | 274.6 (182.5 to 392.8) |
| Maldives | Anxiety disorders | Males | 255.8 (199.7 to 332.6) | 2077 (1648.6 to 2520.8) | 250.6 (167.4 to 372.6) |
|  | Anxiety disorders | Females | 390.1 (301 to 514.4) | 3337.6 (2612.2 to 4088.6) | 395.5 (261.6 to 589.3) |
|  | Anxiety disorders | Both sexes | 309.1 (240.4 to 401.9) | 2574.8 (2037.9 to 3074.9) | 307.7 (204.4 to 448.6) |
| Malta | Anxiety disorders | Males | 1375.3 (1079.1 to 1780) | 10521 (9085.5 to 12668.2) | 1271.3 (861.4 to 1814.2) |
|  | Anxiety disorders | Females | 2491 (2009.4 to 3146.5) | 17520.1 (15475.6 to 19822) | 2093.9 (1514.3 to 2917.8) |
|  | Anxiety disorders | Both sexes | 1905.7 (1531.3 to 2395.3) | 13811.2 (12021.1 to 15889.1) | 1658 (1164.8 to 2319.9) |
| Marshall Islands | Anxiety disorders | Males | 475.5 (318.3 to 762.2) | 4087.6 (2935.8 to 6095.6) | 487.8 (288.3 to 786.8) |
|  | Anxiety disorders | Females | 635.4 (431.5 to 959.2) | 5695.8 (4124.3 to 7959.9) | 672.2 (417.4 to 1052.6) |
|  | Anxiety disorders | Both sexes | 554 (373.8 to 863.9) | 4879.1 (3521.8 to 7018.8) | 578.5 (348.4 to 927.3) |
| Mauritania | Anxiety disorders | Males | 555.4 (408.6 to 783.4) | 4992.9 (3802.7 to 6519) | 599 (382.5 to 881.9) |
|  | Anxiety disorders | Females | 862.5 (606 to 1273.1) | 7808.9 (5982 to 10560.6) | 925.2 (601.8 to 1409.1) |
|  | Anxiety disorders | Both sexes | 710.3 (510.8 to 1030.8) | 6430.1 (5005.1 to 8527) | 765.3 (494.6 to 1150.3) |
| Mauritius | Anxiety disorders | Males | 177.7 (138.8 to 231.1) | 1570.7 (1248.9 to 1910) | 187 (121.8 to 273) |
|  | Anxiety disorders | Females | 267.2 (206.1 to 352.3) | 2596.5 (2036.2 to 3160.8) | 304.6 (205.5 to 452.8) |
|  | Anxiety disorders | Both sexes | 222.6 (172.6 to 290.9) | 2087.5 (1650.6 to 2501.2) | 246.2 (164.5 to 356.7) |
| Mexico | Anxiety disorders | Males | 487.8 (354.6 to 660.1) | 4818 (3564.9 to 6084.9) | 567.9 (359.8 to 859.7) |
|  | Anxiety disorders | Females | 884.7 (643.3 to 1237.3) | 9097.4 (6886.7 to 11567.4) | 1059 (694.3 to 1582.5) |
|  | Anxiety disorders | Both sexes | 689.7 (500.8 to 957.6) | 7014.9 (5271.3 to 8850.2) | 819.7 (530.8 to 1239.2) |
| Micronesia (Federated States of) | Anxiety disorders | Males | 475.5 (318.3 to 762.2) | 4093 (2939.1 to 6103.5) | 486.2 (289.8 to 801.4) |
|  | Anxiety disorders | Females | 635.4 (431.5 to 959.2) | 5703.1 (4131.8 to 7970.5) | 674.2 (412.2 to 1060.3) |
|  | Anxiety disorders | Both sexes | 554.5 (374.6 to 865.4) | 4893.1 (3530.1 to 7040.2) | 579.4 (348.4 to 925.5) |
| Monaco | Anxiety disorders | Males | 907.3 (711.9 to 1174.3) | 7443.6 (6383 to 9007.6) | 898.6 (617.9 to 1280.6) |
|  | Anxiety disorders | Females | 1664.2 (1342.8 to 2095.7) | 13003.4 (11357.1 to 14778.4) | 1551.4 (1123.9 to 2169.3) |
|  | Anxiety disorders | Both sexes | 1283.7 (1031.5 to 1612.2) | 10211.3 (8859.6 to 11783.9) | 1223.3 (862.2 to 1707.1) |
| Mongolia | Anxiety disorders | Males | 390 (285.6 to 542.8) | 3469.4 (2491.2 to 4666) | 416.6 (254 to 649) |
|  | Anxiety disorders | Females | 523 (383.7 to 724.9) | 5038.3 (3678.3 to 6686.4) | 601 (382.2 to 926) |
|  | Anxiety disorders | Both sexes | 457.9 (336.6 to 632.5) | 4278 (3107.4 to 5745.4) | 511.3 (321.2 to 786.6) |
| Montenegro | Anxiety disorders | Males | 587 (407.9 to 865.2) | 5539.2 (4013.2 to 7556.4) | 663.9 (405.8 to 991.9) |
|  | Anxiety disorders | Females | 1033.2 (721.6 to 1515.8) | 10306 (7785.8 to 13683.4) | 1225.3 (765.9 to 1804.5) |
|  | Anxiety disorders | Both sexes | 806.9 (564.4 to 1185.8) | 7901.6 (5879.3 to 10576.8) | 941.6 (580.7 to 1390.2) |
| Morocco | Anxiety disorders | Males | 529.1 (398.9 to 703.7) | 4611.2 (3634.4 to 5909.5) | 553.4 (358.4 to 811.9) |
|  | Anxiety disorders | Females | 681 (511.2 to 892.4) | 6278.7 (4925.7 to 7899) | 738.1 (497.5 to 1067.3) |
|  | Anxiety disorders | Both sexes | 604 (455.1 to 796.6) | 5436.9 (4336.2 to 6893.8) | 644.8 (426.5 to 937.7) |
| Myanmar | Anxiety disorders | Males | 286 (223.3 to 371.9) | 2348.5 (1867.6 to 2854.9) | 279.7 (182.6 to 416.3) |
|  | Anxiety disorders | Females | 432.3 (333.5 to 569.9) | 3745.5 (2963.7 to 4615.4) | 442.1 (299.5 to 651.7) |
|  | Anxiety disorders | Both sexes | 362.5 (280.9 to 474.1) | 3086 (2445.5 to 3758.5) | 365.2 (244.3 to 534.8) |
| Namibia | Anxiety disorders | Males | 490.3 (346.7 to 679.7) | 4930.6 (3586.6 to 6472.8) | 585.9 (376.4 to 866.7) |
|  | Anxiety disorders | Females | 679.6 (488.7 to 945.4) | 6848.6 (5118.4 to 8935.8) | 806 (509.6 to 1217.9) |
|  | Anxiety disorders | Both sexes | 588 (417.1 to 814.8) | 5924.9 (4376.7 to 7651.8) | 699.8 (446.4 to 1045.6) |
| Nauru | Anxiety disorders | Males | 475.5 (318.3 to 762.2) | 4150.5 (2985.2 to 6179.4) | 495.5 (294.9 to 821.3) |
|  | Anxiety disorders | Females | 635.4 (431.5 to 959.2) | 5781 (4200.2 to 8091.5) | 684.4 (420.9 to 1075.4) |
|  | Anxiety disorders | Both sexes | 556.2 (376.2 to 868.6) | 4980.8 (3598.8 to 7160.6) | 591.4 (358.6 to 950.3) |
| Nepal | Anxiety disorders | Males | 493.4 (366.6 to 669.1) | 4391.6 (3403 to 5779) | 525.2 (346.6 to 800.3) |
|  | Anxiety disorders | Females | 606.5 (452.7 to 828.4) | 5684.6 (4404.7 to 7420.3) | 670.8 (454.3 to 1029.5) |
|  | Anxiety disorders | Both sexes | 551.4 (410.3 to 750.9) | 5059.3 (3925.3 to 6622.4) | 600.3 (402.5 to 917.7) |
| Netherlands | Anxiety disorders | Males | 1160.3 (911.8 to 1499.7) | 9242.3 (7458.8 to 11298.9) | 1109.1 (756 to 1579) |
|  | Anxiety disorders | Females | 1959.7 (1533.9 to 2495.7) | 15636.2 (13186.5 to 18731.4) | 1839.8 (1292.9 to 2545.6) |
|  | Anxiety disorders | Both sexes | 1554.1 (1223.4 to 1979.2) | 12411.1 (10285 to 14950.9) | 1471 (1023.5 to 2055.2) |
| New Zealand | Anxiety disorders | Males | 961.5 (656.9 to 1304.4) | 6790.1 (5027 to 8726.6) | 809.6 (504.9 to 1214.4) |
|  | Anxiety disorders | Females | 1853.2 (1271.2 to 2544.2) | 13359.4 (10066.1 to 16999.8) | 1564.2 (1005.6 to 2302.9) |
|  | Anxiety disorders | Both sexes | 1401.1 (961 to 1923.9) | 10046.2 (7528.1 to 12758.4) | 1183.3 (755 to 1749.8) |
| Nicaragua | Anxiety disorders | Males | 534.4 (402.5 to 722.5) | 4963.9 (3716.1 to 6181.6) | 585.7 (378.4 to 892.7) |
|  | Anxiety disorders | Females | 845.3 (625.3 to 1173.6) | 8490.7 (6545.5 to 10560.4) | 991.7 (655.7 to 1469) |
|  | Anxiety disorders | Both sexes | 691.4 (515 to 947.3) | 6769.2 (5158.2 to 8400.6) | 793.2 (519.6 to 1184.6) |
| Nigeria | Anxiety disorders | Males | 627.7 (418.7 to 981.8) | 5280.8 (3877.1 to 7608.1) | 631.6 (385.4 to 1002.5) |
|  | Anxiety disorders | Females | 1068.4 (585.1 to 2018.3) | 8524.9 (5628.8 to 13020.1) | 1008.4 (576.8 to 1703.7) |
|  | Anxiety disorders | Both sexes | 852.7 (507.8 to 1518.9) | 6976.6 (4838.9 to 10455.3) | 828.3 (488.1 to 1365.5) |
| North Macedonia | Anxiety disorders | Males | 495.6 (344.4 to 730.5) | 4706.6 (3406.8 to 6426.5) | 565 (348.8 to 853.6) |
|  | Anxiety disorders | Females | 856 (597.9 to 1255.9) | 8673.5 (6522.3 to 11617.5) | 1031.8 (640 to 1540.6) |
|  | Anxiety disorders | Both sexes | 673.9 (471.1 to 990.6) | 6678.3 (4953.4 to 9116.9) | 796.7 (491.4 to 1190) |
| Northern Mariana Islands | Anxiety disorders | Males | 475.5 (318.3 to 762.2) | 4068.2 (2922.1 to 6071) | 488.1 (288 to 791.6) |
|  | Anxiety disorders | Females | 635.4 (431.5 to 959.2) | 5669.5 (4109.4 to 7924.4) | 674.2 (418.2 to 1052.1) |
|  | Anxiety disorders | Both sexes | 551.3 (371.9 to 860.8) | 4827.2 (3482.3 to 6960.7) | 576.3 (347.1 to 927.7) |
| Norway | Anxiety disorders | Males | 806 (580.9 to 1094.9) | 6228.4 (4668 to 8266.6) | 749.4 (475.6 to 1102.2) |
|  | Anxiety disorders | Females | 1539.1 (1047.3 to 2331.9) | 11898.6 (8841 to 15843) | 1414.1 (903.4 to 2055.2) |
|  | Anxiety disorders | Both sexes | 1164 (816.1 to 1708.6) | 9005.1 (6717.2 to 11998.1) | 1074.6 (678.2 to 1563.7) |
| Oman | Anxiety disorders | Males | 544 (410.1 to 723.6) | 5123.2 (4052.2 to 6562.8) | 612.4 (399.2 to 906.9) |
|  | Anxiety disorders | Females | 643.6 (483.1 to 843.4) | 6680.6 (5255.4 to 8367) | 784.8 (525.2 to 1140) |
|  | Anxiety disorders | Both sexes | 584 (439.8 to 775) | 5708.8 (4560.8 to 7250.9) | 677.3 (450.9 to 991.3) |
| Pakistan | Anxiety disorders | Males | 466.6 (346.7 to 632.7) | 4250.8 (3293.4 to 5562.7) | 505.4 (333.4 to 773.5) |
|  | Anxiety disorders | Females | 538.9 (402.2 to 736) | 5398.4 (4218.3 to 6971.9) | 629.5 (427.7 to 934.4) |
|  | Anxiety disorders | Both sexes | 501.6 (373.4 to 683.5) | 4804.4 (3738.5 to 6243.1) | 565.4 (378.8 to 854.6) |
| Palau | Anxiety disorders | Males | 475.5 (318.3 to 762.2) | 4075.6 (2926.6 to 6082.1) | 486.4 (291.4 to 785.6) |
|  | Anxiety disorders | Females | 635.4 (431.5 to 959.2) | 5679.6 (4116.3 to 7937.4) | 672.8 (416.8 to 1057.9) |
|  | Anxiety disorders | Both sexes | 548.2 (369.8 to 857.4) | 4804.6 (3464.4 to 6940.9) | 571.1 (350.4 to 912.1) |
| Palestine | Anxiety disorders | Males | 590.8 (445.4 to 785.8) | 5670.4 (4483.1 to 7230.5) | 675.5 (443.6 to 1002.1) |
|  | Anxiety disorders | Females | 687.8 (516.3 to 901.4) | 7181.6 (5665 to 8937.9) | 839.5 (567.4 to 1213.9) |
|  | Anxiety disorders | Both sexes | 638.7 (481.2 to 843.2) | 6418.2 (5151.8 to 8086.9) | 756.6 (504.8 to 1102.2) |
| Panama | Anxiety disorders | Males | 439.3 (330.8 to 594) | 3903.4 (2923.6 to 4900.7) | 462.1 (300.7 to 702) |
|  | Anxiety disorders | Females | 665.5 (492.2 to 923.9) | 6499.3 (4974 to 8147.4) | 762 (508.2 to 1134.1) |
|  | Anxiety disorders | Both sexes | 552.6 (411.3 to 757.3) | 5213.9 (3951.7 to 6535.9) | 613.4 (403.9 to 922) |
| Papua New Guinea | Anxiety disorders | Males | 475.5 (318.3 to 762.2) | 3848.2 (2772.2 to 5764.8) | 460.4 (271.7 to 717.9) |
|  | Anxiety disorders | Females | 635.4 (431.5 to 959.2) | 5371.5 (3899.4 to 7527.6) | 635.8 (394.3 to 996.7) |
|  | Anxiety disorders | Both sexes | 552.4 (372.2 to 861.2) | 4580.4 (3316.7 to 6615) | 544.7 (331 to 861.3) |
| Paraguay | Anxiety disorders | Males | 724.7 (555.7 to 971.8) | 6796.2 (5300.1 to 8301.4) | 802.4 (529.3 to 1180.8) |
|  | Anxiety disorders | Females | 1181.8 (886.7 to 1634.2) | 11562.4 (8987.3 to 14185.6) | 1350.5 (913.5 to 2008.2) |
|  | Anxiety disorders | Both sexes | 954.7 (723.3 to 1305.3) | 9196.7 (7177.4 to 11254.7) | 1078.2 (722.1 to 1594.7) |
| Peru | Anxiety disorders | Males | 763.8 (554.1 to 1037.8) | 7591.7 (5643 to 9751.1) | 904.5 (587 to 1351.1) |
|  | Anxiety disorders | Females | 1264 (901.6 to 1710) | 12692.4 (9631 to 15566.5) | 1492.8 (970 to 2174.7) |
|  | Anxiety disorders | Both sexes | 1012.4 (726.2 to 1368.9) | 10135.4 (7631.8 to 12535.9) | 1197.6 (774.8 to 1739.2) |
| Philippines | Anxiety disorders | Males | 285.1 (222.6 to 370.6) | 2490.1 (1981.3 to 3019.1) | 298.8 (195.9 to 427.3) |
|  | Anxiety disorders | Females | 419.6 (323.5 to 553.2) | 3862.8 (3046.6 to 4735.3) | 457.6 (304.7 to 670) |
|  | Anxiety disorders | Both sexes | 351.9 (272.8 to 460.1) | 3177 (2516.6 to 3838.6) | 378.1 (249.7 to 545.7) |
| Poland | Anxiety disorders | Males | 302.6 (210.3 to 446) | 2956.3 (2151.5 to 4070.5) | 353.8 (213.2 to 531.2) |
|  | Anxiety disorders | Females | 554.1 (387 to 813) | 5888.9 (4437.2 to 7995.7) | 699.2 (437.7 to 1041.3) |
|  | Anxiety disorders | Both sexes | 428.6 (300.2 to 630.1) | 4434.4 (3300.6 to 6039) | 527.4 (327.3 to 787.4) |
| Portugal | Anxiety disorders | Males | 1331.8 (907.5 to 1825.3) | 10577.7 (7811.2 to 13618.8) | 1270.8 (798.4 to 1887.1) |
|  | Anxiety disorders | Females | 2121.5 (1352.7 to 3205.6) | 16808.3 (12497.3 to 21456.8) | 1996.9 (1267.9 to 2915.7) |
|  | Anxiety disorders | Both sexes | 1722.9 (1132.2 to 2520.8) | 13711.4 (10176.1 to 17733.1) | 1635.6 (1042.2 to 2400.1) |
| Puerto Rico | Anxiety disorders | Males | 676.5 (494.8 to 925.4) | 5861.8 (4468.2 to 7538.8) | 695.2 (449.8 to 1039.4) |
|  | Anxiety disorders | Females | 1105.1 (801.6 to 1489.3) | 10000.1 (7830.8 to 12275.1) | 1171.7 (772.1 to 1700.1) |
|  | Anxiety disorders | Both sexes | 897.1 (650.5 to 1218.8) | 8024.7 (6240.8 to 10069.1) | 944 (619.1 to 1387.3) |
| Qatar | Anxiety disorders | Males | 419.7 (301.5 to 641.5) | 3572.8 (2671.1 to 5067.1) | 428.1 (265.1 to 648.8) |
|  | Anxiety disorders | Females | 516 (369.3 to 746.3) | 4708.3 (3603.9 to 6379.8) | 554 (354.1 to 822.5) |
|  | Anxiety disorders | Both sexes | 456 (324.6 to 687.1) | 3963.6 (2983.1 to 5544.7) | 471.7 (299.2 to 705.4) |
| Republic of Korea | Anxiety disorders | Males | 494.2 (340.3 to 814.7) | 3786.8 (2725.5 to 6000.9) | 460.2 (266.9 to 757) |
|  | Anxiety disorders | Females | 807.4 (561.2 to 1215.6) | 6684.3 (4984.2 to 9512) | 803.9 (488.7 to 1262.4) |
|  | Anxiety disorders | Both sexes | 647 (450.6 to 1005.2) | 5201.9 (3838.6 to 7711.1) | 627.8 (376.9 to 1006.3) |
| Republic of Moldova | Anxiety disorders | Males | 465.6 (332.4 to 695.5) | 4571.6 (3275.7 to 6231.4) | 550 (339.9 to 853.1) |
|  | Anxiety disorders | Females | 694.4 (498 to 1009.6) | 7109.1 (5256.6 to 9487.4) | 847.9 (527.3 to 1326.8) |
|  | Anxiety disorders | Both sexes | 580.7 (417.4 to 848.4) | 5858.6 (4274.8 to 7949) | 700.5 (437.3 to 1089.7) |
| Romania | Anxiety disorders | Males | 407.4 (281.6 to 614.4) | 3972.5 (2926.2 to 5613) | 475.3 (283.7 to 723.2) |
|  | Anxiety disorders | Females | 729.3 (502.5 to 1102.5) | 7575.6 (5701.5 to 10385.2) | 902.6 (566.4 to 1353.5) |
|  | Anxiety disorders | Both sexes | 567.8 (393.1 to 847) | 5776.6 (4312 to 7992.5) | 688.8 (425.3 to 1033.6) |
| Russian Federation | Anxiety disorders | Males | 300.3 (214.4 to 448.9) | 2822 (2021 to 3898.5) | 337.9 (208.9 to 524) |
|  | Anxiety disorders | Females | 528 (378.6 to 767.6) | 5309.8 (3892.1 to 7139.1) | 631.2 (393.2 to 976.9) |
|  | Anxiety disorders | Both sexes | 416.6 (300 to 606.2) | 4102.6 (2986.4 to 5535.1) | 488.2 (303.7 to 754.6) |
| Saint Kitts and Nevis | Anxiety disorders | Males | 594.8 (439.1 to 807.4) | 5415.3 (4078.5 to 7000.3) | 640.9 (412.6 to 966.5) |
|  | Anxiety disorders | Females | 887.6 (639 to 1215) | 8640.2 (6610.1 to 10708.2) | 1012.9 (665.5 to 1507.4) |
|  | Anxiety disorders | Both sexes | 746.9 (543.2 to 1018.1) | 7099.3 (5402.7 to 8906.4) | 835.1 (545.2 to 1243.2) |
| Saint Lucia | Anxiety disorders | Males | 594.8 (439.1 to 807.4) | 5528.6 (4160.3 to 7183.5) | 653.9 (418.5 to 977.8) |
|  | Anxiety disorders | Females | 887.6 (639 to 1215) | 8807.2 (6756.9 to 10935.2) | 1029.3 (671.9 to 1522.7) |
|  | Anxiety disorders | Both sexes | 740.5 (538.8 to 1008.5) | 7167.4 (5459.3 to 9016.9) | 841.5 (550.5 to 1259.2) |
| Saint Vincent and the Grenadines | Anxiety disorders | Males | 594.8 (439.1 to 807.4) | 5178.6 (3903 to 6669.8) | 613.2 (397 to 920.2) |
|  | Anxiety disorders | Females | 887.6 (639 to 1215) | 8291 (6356.6 to 10316.1) | 968.7 (631.9 to 1441.1) |
|  | Anxiety disorders | Both sexes | 738.2 (536.9 to 1005.8) | 6703.2 (5110 to 8424.3) | 787.4 (507.4 to 1170.5) |
| Samoa | Anxiety disorders | Males | 475.5 (318.3 to 762.2) | 4097.8 (2941.8 to 6110.7) | 492.2 (290.9 to 788.1) |
|  | Anxiety disorders | Females | 635.4 (431.5 to 959.2) | 5709.7 (4150.3 to 7980.3) | 678.6 (419 to 1070.7) |
|  | Anxiety disorders | Both sexes | 555 (374.4 to 864.9) | 4901.8 (3546.6 to 7045.9) | 585.1 (353.6 to 930.1) |
| San Marino | Anxiety disorders | Males | 907.3 (711.9 to 1174.3) | 7770.2 (6665 to 9385.3) | 936.9 (639.4 to 1344.2) |
|  | Anxiety disorders | Females | 1664.2 (1342.8 to 2095.7) | 13513.1 (11825.8 to 15327.9) | 1610.1 (1152.2 to 2235.1) |
|  | Anxiety disorders | Both sexes | 1274.3 (1023.8 to 1601.2) | 10564.1 (9145.9 to 12201.4) | 1264 (878.7 to 1765.9) |
| Sao Tome and Principe | Anxiety disorders | Males | 721.8 (531 to 1018.1) | 6552.4 (5018.5 to 8542.3) | 786.8 (500.8 to 1144.7) |
|  | Anxiety disorders | Females | 1057.8 (743.2 to 1561.2) | 9802.8 (7587.8 to 12993.8) | 1167.7 (759.5 to 1778.4) |
|  | Anxiety disorders | Both sexes | 889 (639.5 to 1290.9) | 8176.7 (6424.6 to 10664.7) | 977.1 (632 to 1464.5) |
| Saudi Arabia | Anxiety disorders | Males | 599.3 (409.3 to 911.2) | 5537.2 (4058.9 to 7547.8) | 659.6 (405.1 to 1005.8) |
|  | Anxiety disorders | Females | 942.9 (590 to 1480.4) | 9108 (6501.3 to 12464.9) | 1071.5 (646.8 to 1686.5) |
|  | Anxiety disorders | Both sexes | 753.8 (491.3 to 1171.1) | 7011.4 (5038.1 to 9655.7) | 830.8 (515 to 1267.7) |
| Senegal | Anxiety disorders | Males | 612.4 (450.5 to 863.8) | 5640.1 (4331.2 to 7354.1) | 675.2 (431.2 to 990.2) |
|  | Anxiety disorders | Females | 946.5 (665 to 1397) | 8678.9 (6685.3 to 11624) | 1023.9 (647.8 to 1560.2) |
|  | Anxiety disorders | Both sexes | 774.3 (558 to 1121.1) | 7132.5 (5596.1 to 9324) | 846 (539.3 to 1272) |
| Serbia | Anxiety disorders | Males | 492.4 (342.1 to 725.7) | 4880.4 (3591.9 to 6627.2) | 585.1 (352.3 to 869.8) |
|  | Anxiety disorders | Females | 911.6 (636.7 to 1337.5) | 9362.7 (7114.4 to 12451.2) | 1116.7 (702 to 1646.8) |
|  | Anxiety disorders | Both sexes | 695.6 (487.2 to 1022.4) | 7063.3 (5299.4 to 9567.1) | 843.4 (523.5 to 1248.6) |
| Seychelles | Anxiety disorders | Males | 255.8 (199.7 to 332.6) | 2219.8 (1768.4 to 2697.3) | 266.5 (174.3 to 387.5) |
|  | Anxiety disorders | Females | 390.1 (301 to 514.4) | 3559.5 (2813.8 to 4368.1) | 422 (279.9 to 620.9) |
|  | Anxiety disorders | Both sexes | 315.5 (244.9 to 411.5) | 2816.1 (2238.5 to 3405.9) | 335.5 (222.1 to 489.4) |
| Singapore | Anxiety disorders | Males | 339.4 (237.5 to 510.7) | 2620.9 (1872.4 to 3885.8) | 318.1 (184.4 to 515.6) |
|  | Anxiety disorders | Females | 437.3 (297.5 to 625.2) | 3841.5 (2754.4 to 5334.3) | 462.8 (278.2 to 721.8) |
|  | Anxiety disorders | Both sexes | 387.6 (267.5 to 562.8) | 3222.9 (2304.1 to 4574.5) | 389.4 (228.8 to 615.9) |
| Slovakia | Anxiety disorders | Males | 407.6 (283.2 to 600.8) | 3821.8 (2786.8 to 5237.7) | 457.5 (280.9 to 686.2) |
|  | Anxiety disorders | Females | 736.1 (514.1 to 1079.9) | 7364.4 (5549.9 to 9920.7) | 875.1 (542.2 to 1288.3) |
|  | Anxiety disorders | Both sexes | 571.5 (400.4 to 840.9) | 5598.8 (4169.7 to 7673.4) | 666.5 (411.1 to 986) |
| Slovenia | Anxiety disorders | Males | 542.7 (377.1 to 799.9) | 4868.8 (3573 to 6609.7) | 582.7 (356.7 to 878.9) |
|  | Anxiety disorders | Females | 1078.9 (753.6 to 1582.9) | 9918 (7514.3 to 13158.9) | 1178.8 (737.2 to 1751.5) |
|  | Anxiety disorders | Both sexes | 803.1 (562.2 to 1183.8) | 7322.5 (5483.7 to 9917) | 871.8 (543.3 to 1301.7) |
| Solomon Islands | Anxiety disorders | Males | 475.5 (318.3 to 762.2) | 3941.9 (2837 to 5878) | 472.7 (283.9 to 756.6) |
|  | Anxiety disorders | Females | 635.4 (431.5 to 959.2) | 5498.4 (3982.7 to 7706.4) | 653.1 (406 to 1027) |
|  | Anxiety disorders | Both sexes | 553.6 (373.4 to 863.3) | 4703.6 (3397.5 to 6773.1) | 560.9 (346.4 to 888.6) |
| South Africa | Anxiety disorders | Males | 563.3 (391.4 to 810.3) | 5450.9 (3989.2 to 7325.8) | 645.4 (401.9 to 984.3) |
|  | Anxiety disorders | Females | 781.1 (553.1 to 1145.7) | 7699.7 (5758 to 10461.5) | 898.1 (577.4 to 1358.8) |
|  | Anxiety disorders | Both sexes | 674.9 (474.7 to 978.6) | 6613.1 (4901.4 to 8931.2) | 775.7 (492.7 to 1173.6) |
| Spain | Anxiety disorders | Males | 834.5 (579 to 1139) | 6860.6 (5026.5 to 9137.6) | 824.4 (513.7 to 1225.8) |
|  | Anxiety disorders | Females | 1195 (786.9 to 1751.3) | 10473.1 (7633 to 13990.9) | 1243.1 (785.5 to 1847.8) |
|  | Anxiety disorders | Both sexes | 1010.9 (673.9 to 1436.9) | 8647.8 (6322.3 to 11583.8) | 1031.3 (650.2 to 1530.5) |
| Sri Lanka | Anxiety disorders | Males | 230.3 (179.8 to 299.4) | 1737.7 (1387 to 2115.7) | 207.6 (135.1 to 301.5) |
|  | Anxiety disorders | Females | 338.9 (261.3 to 446.8) | 2737.5 (2163.3 to 3381.3) | 324.4 (216.1 to 478.9) |
|  | Anxiety disorders | Both sexes | 286.4 (221.9 to 374.6) | 2257.4 (1788.3 to 2763.1) | 268.2 (178.3 to 395.1) |
| Suriname | Anxiety disorders | Males | 570.2 (421 to 774) | 5368.4 (4031.4 to 6914.7) | 635.7 (404.3 to 957) |
|  | Anxiety disorders | Females | 831.8 (599 to 1138.6) | 8287.3 (6324 to 10326.6) | 965.1 (632.7 to 1442.7) |
|  | Anxiety disorders | Both sexes | 702.2 (511.3 to 956.8) | 6858.6 (5205 to 8532.1) | 803.7 (520.2 to 1205.7) |
| Sweden | Anxiety disorders | Males | 662 (454.7 to 913.5) | 5223.9 (3732.4 to 7035.8) | 626.3 (387.7 to 956.6) |
|  | Anxiety disorders | Females | 1308.4 (1055.8 to 1647.8) | 10003 (8672.8 to 11445.8) | 1194.3 (856.3 to 1674) |
|  | Anxiety disorders | Both sexes | 975.4 (760.8 to 1238) | 7543.3 (6188.4 to 9085.9) | 901.9 (623.1 to 1278.6) |
| Switzerland | Anxiety disorders | Males | 711.9 (496.4 to 999.9) | 6192.8 (4668 to 8199.6) | 742.8 (473.5 to 1085.6) |
|  | Anxiety disorders | Females | 1212.2 (793.7 to 1893.5) | 10765.9 (7957.6 to 14147.5) | 1278.6 (790.5 to 1904.4) |
|  | Anxiety disorders | Both sexes | 955.8 (643.1 to 1418) | 8434.2 (6196.5 to 11132.6) | 1005.2 (631.7 to 1489.5) |
| Taiwan | Anxiety disorders | Males | 443.4 (298 to 742.6) | 3251.4 (2426.7 to 4785.9) | 392.4 (245.2 to 625) |
|  | Anxiety disorders | Females | 747.6 (488.1 to 1244.1) | 5636.1 (4214.6 to 7843.2) | 678.8 (427 to 1067.8) |
|  | Anxiety disorders | Both sexes | 591.6 (389.8 to 969.6) | 4417.7 (3264.3 to 6257.8) | 532.3 (334.1 to 839.5) |
| Tajikistan | Anxiety disorders | Males | 303 (221.9 to 421.7) | 2860.1 (2056.5 to 3847.1) | 343.8 (210.9 to 531.4) |
|  | Anxiety disorders | Females | 484.2 (355.2 to 671.2) | 4768.2 (3474.1 to 6353.8) | 567.8 (355.2 to 872.5) |
|  | Anxiety disorders | Both sexes | 392.3 (288 to 542) | 3803.6 (2755.3 to 5110) | 454.5 (284.1 to 701.6) |
| Thailand | Anxiety disorders | Males | 240.3 (172.3 to 347.9) | 1792.6 (1315.7 to 2450.8) | 214.8 (134.5 to 332.1) |
|  | Anxiety disorders | Females | 382.5 (274.2 to 528.2) | 2924.3 (2173.5 to 3794.7) | 347.1 (223.4 to 533.5) |
|  | Anxiety disorders | Both sexes | 313.4 (224.8 to 439) | 2378.4 (1759.7 to 3131.5) | 283.2 (180.9 to 435.1) |
| Timor-Leste | Anxiety disorders | Males | 255.8 (199.7 to 332.6) | 2003.6 (1593.1 to 2433) | 240.1 (157.1 to 350.8) |
|  | Anxiety disorders | Females | 390.1 (301 to 514.4) | 3223.2 (2527.9 to 3932.9) | 382.1 (252.9 to 561.6) |
|  | Anxiety disorders | Both sexes | 321.6 (249.2 to 420.8) | 2601.9 (2052.8 to 3130.1) | 309.8 (204.1 to 453.5) |
| Tonga | Anxiety disorders | Males | 601.3 (366.1 to 1212.8) | 5093.4 (3432.1 to 8457.4) | 613.3 (353.1 to 1141.4) |
|  | Anxiety disorders | Females | 882.7 (531.1 to 1785) | 7616.2 (5114.5 to 12283.5) | 908.3 (528.4 to 1598.8) |
|  | Anxiety disorders | Both sexes | 745.5 (454.7 to 1477.6) | 6415.4 (4367.1 to 10649.7) | 767.5 (448.9 to 1373.2) |
| Trinidad and Tobago | Anxiety disorders | Males | 539.1 (398 to 731.7) | 4889.2 (3642.6 to 6311) | 578 (370 to 876.3) |
|  | Anxiety disorders | Females | 778.5 (560.5 to 1065.8) | 7567.4 (5769.6 to 9492.3) | 882.6 (573.7 to 1319.8) |
|  | Anxiety disorders | Both sexes | 657.9 (478.9 to 895.7) | 6223 (4701.2 to 7764.2) | 729.6 (471.1 to 1099.5) |
| Tunisia | Anxiety disorders | Males | 677.1 (510.5 to 900.6) | 6304.6 (5008.7 to 8068.3) | 753.5 (496.2 to 1114.6) |
|  | Anxiety disorders | Females | 737.6 (553.6 to 966.6) | 7589.8 (5959.9 to 9510.8) | 892.5 (602.7 to 1297.6) |
|  | Anxiety disorders | Both sexes | 707.7 (533.2 to 934.1) | 6960.2 (5567.9 to 8785.6) | 824.4 (546.4 to 1200.2) |
| Türkiye | Anxiety disorders | Males | 471.5 (342.4 to 672.4) | 4364.4 (3271.3 to 5787.2) | 524.7 (332.9 to 775.9) |
|  | Anxiety disorders | Females | 557.1 (394 to 744.4) | 5583.8 (4102.4 to 7039.8) | 655 (420 to 972.9) |
|  | Anxiety disorders | Both sexes | 514.2 (369.2 to 709.1) | 4975.7 (3660.2 to 6410.8) | 590 (377.5 to 876.3) |
| Turkmenistan | Anxiety disorders | Males | 352.1 (257.8 to 490) | 3183.6 (2300.9 to 4283.8) | 382.1 (234.2 to 591.4) |
|  | Anxiety disorders | Females | 489.8 (359.3 to 679) | 4604.2 (3359.4 to 6123.9) | 548.4 (351.3 to 843.2) |
|  | Anxiety disorders | Both sexes | 419.7 (308.5 to 579.7) | 3883.9 (2828.8 to 5216.4) | 463.8 (296.1 to 711.1) |
| Tuvalu | Anxiety disorders | Males | 475.5 (318.3 to 762.2) | 4039.2 (2904.1 to 6034.2) | 482.9 (294.7 to 785.1) |
|  | Anxiety disorders | Females | 635.4 (431.5 to 959.2) | 5630.3 (4084.3 to 7863.3) | 667.7 (409.4 to 1049.2) |
|  | Anxiety disorders | Both sexes | 553.1 (373.7 to 863.8) | 4817.1 (3478.5 to 6944.1) | 573 (352.9 to 919.1) |
| Ukraine | Anxiety disorders | Males | 336.6 (231.4 to 536.6) | 3309.9 (2427.1 to 4780.7) | 397 (240.2 to 613.6) |
|  | Anxiety disorders | Females | 604 (422.6 to 950.3) | 6042.3 (4508.2 to 8613.6) | 717.8 (438 to 1103.9) |
|  | Anxiety disorders | Both sexes | 473.4 (331 to 749) | 4719.9 (3496.7 to 6793.9) | 561.9 (340.6 to 862.1) |
| United Arab Emirates | Anxiety disorders | Males | 423.8 (319.4 to 563.6) | 3780.6 (2965.3 to 4827.4) | 452.5 (298.6 to 665.4) |
|  | Anxiety disorders | Females | 644.3 (430.3 to 953.9) | 6064.7 (4425 to 8140.7) | 712.8 (445.8 to 1074.6) |
|  | Anxiety disorders | Both sexes | 510.3 (359.2 to 749.5) | 4599.7 (3395.8 to 6219.6) | 547.3 (352.1 to 801) |
| United Kingdom | Anxiety disorders | Males | 983.3 (734.5 to 1270.9) | 7899.4 (6069.9 to 9794.7) | 942.8 (622.5 to 1358.9) |
|  | Anxiety disorders | Females | 2005.6 (1528.3 to 2459.1) | 14642.3 (11873.8 to 16907.8) | 1719.9 (1188.2 to 2385.1) |
|  | Anxiety disorders | Both sexes | 1485.9 (1113.5 to 1848.7) | 11267.8 (8968.1 to 13359.5) | 1330.7 (910.5 to 1872.5) |
| United Republic of Tanzania | Anxiety disorders | Males | 367.5 (279.5 to 468.4) | 3493.9 (2668.1 to 4207.3) | 415.9 (271.2 to 600.1) |
|  | Anxiety disorders | Females | 472.6 (359.7 to 616) | 4579.7 (3556.4 to 5517.1) | 536.3 (366.2 to 778) |
|  | Anxiety disorders | Both sexes | 421.7 (320.9 to 544.5) | 4054.8 (3125.7 to 4876) | 478 (318.5 to 685.6) |
| United States of America | Anxiety disorders | Males | 623.2 (451.1 to 826.7) | 5097.6 (3835.2 to 6376.1) | 598.8 (392.2 to 886.6) |
|  | Anxiety disorders | Females | 1131.6 (831.8 to 1516.9) | 9026.6 (6868.7 to 11024.7) | 1041.5 (685.1 to 1520.6) |
|  | Anxiety disorders | Both sexes | 876.4 (641.5 to 1168.4) | 7063.7 (5317.1 to 8636.1) | 820 (542 to 1194.3) |
| United States Virgin Islands | Anxiety disorders | Males | 594.8 (439.1 to 807.4) | 5291.5 (3987.5 to 6794.5) | 628.1 (409.9 to 942.6) |
|  | Anxiety disorders | Females | 887.6 (639 to 1215) | 8457.6 (6469.8 to 10500.3) | 994.4 (644.9 to 1488.7) |
|  | Anxiety disorders | Both sexes | 743.3 (541.1 to 1012.6) | 6908.9 (5256.4 to 8655.5) | 815.1 (528.5 to 1200.9) |
| Uruguay | Anxiety disorders | Males | 752.2 (535.8 to 1050) | 6421.6 (4740.2 to 8596.5) | 771.2 (484.9 to 1166.1) |
|  | Anxiety disorders | Females | 1256.1 (876.3 to 1755.3) | 11413.8 (8475.1 to 14820.5) | 1351.6 (861.8 to 1998.5) |
|  | Anxiety disorders | Both sexes | 1006.8 (711.2 to 1402.4) | 8972.9 (6670.7 to 11870.1) | 1067.3 (682.8 to 1592.7) |
| Uzbekistan | Anxiety disorders | Males | 218.8 (160.2 to 304.6) | 1865.6 (1341.5 to 2513.7) | 224.6 (139.3 to 346) |
|  | Anxiety disorders | Females | 404.9 (297.1 to 561.3) | 3606.7 (2615.7 to 4839.8) | 430 (263 to 662.1) |
|  | Anxiety disorders | Both sexes | 312 (229.5 to 431.4) | 2742 (1988.7 to 3688.3) | 327.7 (203.7 to 499.9) |
| Vanuatu | Anxiety disorders | Males | 475.5 (318.3 to 762.2) | 3961.2 (2850.1 to 5904) | 475.5 (282.8 to 769.8) |
|  | Anxiety disorders | Females | 635.4 (431.5 to 959.2) | 5524.6 (4000.3 to 7714.1) | 658 (409.1 to 1027.7) |
|  | Anxiety disorders | Both sexes | 555.7 (374.9 to 865.9) | 4747.8 (3428.4 to 6813.2) | 567.3 (347.7 to 913.4) |
| Viet Nam | Anxiety disorders | Males | 194.2 (145.3 to 256.5) | 1581.8 (1202.6 to 2035.3) | 189.6 (124.1 to 278.8) |
|  | Anxiety disorders | Females | 318.4 (239 to 425.6) | 2757.5 (2142.4 to 3339.8) | 328.3 (219.2 to 475.8) |
|  | Anxiety disorders | Both sexes | 256.4 (192.8 to 340.3) | 2177.5 (1676.3 to 2675.9) | 259.7 (173 to 377.4) |
| Zambia | Anxiety disorders | Males | 687.1 (522.6 to 875.7) | 6247.5 (4759.3 to 7493) | 739.5 (493.3 to 1081.2) |
|  | Anxiety disorders | Females | 713.4 (543 to 929.8) | 6776.4 (5282.8 to 8130.1) | 791.1 (526.5 to 1150.6) |
|  | Anxiety disorders | Both sexes | 701.2 (533.4 to 904.1) | 6525.9 (5034.1 to 7818.6) | 766.6 (511 to 1110.2) |
| Zimbabwe | Anxiety disorders | Males | 463.7 (327.9 to 642.7) | 4734.4 (3468.2 to 6136.6) | 563.6 (353.4 to 835.8) |
|  | Anxiety disorders | Females | 657.3 (448.4 to 976.2) | 6686.5 (4925 to 9228.5) | 785.6 (485.7 to 1216.4) |
|  | Anxiety disorders | Both sexes | 564.7 (388.7 to 816.7) | 5764.8 (4238.9 to 7734.5) | 680.6 (425 to 1024.9) |

**Table S6. Average annual percent change of age-standardized incidence, prevalence and DALYs rate from 1990 to 202**1

| Locations | Sex | ASIR AAPC and 95%CI | ASPR AAPC and 95%CI | ASDR AAPC and 95%CI |
| --- | --- | --- | --- | --- |
| World Bank High Income | Both sexes | 1.1134(0.4351 to 1.5103) | 1.8352（1.7223 to 1.9339） | 1.8429(1.7420 to 1.9426) |
|  | Females | 1.0817(0.4065 to 1.4967) | 1.7209(1.6031 to 1.8201) | 1.7250(1.6223 to 1.8250) |
|  | Males | 1.1929(0.5017 to 1.5945) | 2.0878(1.9733 to 2.1914) | 2.0961(1.9931 to 2.1997) |
| World Bank Upper Middle Income | Both sexes | 1.4590(0.8033 to 2.0164) | 2.16（2.0531 to 2.2656） | 2.1564(2.0457 to 2.2597) |
|  | Females | 1.4720(0.7786 to 2.0578) | 2.2055(2.0876 to 2.3145) | 2.1972(2.0796 to 2.3076) |
|  | Males | 1.4620(0.8247 to 1.9881) | 2.1141(2.0151 to 2.2157) | 2.1165(2.0089 to 2.2272) |
| World Bank Lower Middle Income | Both sexes | 3.1564(2.2475 to 4.0920) | 4.0416(3.8505 to 4.2195) | 4.0530(3.8708 to 4.2321） |
|  | Females | 3.3297(2.4352 to 4.1281) | 4.1715(3.9800 to 4.3493) | 4.1819(4.0051 to 4.3534) |
|  | Males | 3.0884(2.3150 to 3.5959) | 3.8398(3.6476 to 4.0198) | 3.8566(3.6627 to 4.0413) |
| Albania | Both sexes | 1.7 (1.0 to 2.3) | 1.1 (0.9 to 1.4) | 1.1 (0.9 to 1.4) |
|  | Females | 2.0 (1.3 to 2.6) | 1.4 (1.1 to 1.6) | 1.4 (1.1 to 1.6) |
|  | Males | 1.1 (0.4 to 1.8) | 0.6 (0.4 to 0.9) | 0.6 (0.4 to 0.9) |
| Algeria | Both sexes | -0.1 (-0.7 to 0.4) | -0.3 (-0.5 to -0.2) | -0.4 (-0.5 to -0.2) |
|  | Females | -0.8 (-1.4 to -0.2) | -0.9 (-1.0 to -0.7) | -0.9 (-1.0 to -0.7) |
|  | Males | 0.6 (0.1 to 1.2) | 0.3 (0.2 to 0.5) | 0.3 (0.2 to 0.5) |
| American Samoa | Both sexes | 1.8 (1.1 to 2.5) | 1.0 (0.7 to 1.3) | 1.0 (0.6 to 1.3) |
|  | Females | 1.9 (1.2 to 2.6) | 1.1 (0.7 to 1.4) | 1.0 (0.7 to 1.4) |
|  | Males | 1.7 (0.9 to 2.4) | 0.9 (0.5 to 1.2) | 0.8 (0.5 to 1.2) |
| Andorra | Both sexes | 3.8 (3.1 to 4.5) | 2.1 (1.6 to 2.6) | 2.1 (1.6 to 2.6) |
|  | Females | 4.1 (3.4 to 4.8) | 1.9 (1.4 to 2.4) | 1.9 (1.4 to 2.4) |
|  | Males | 3.3 (2.6 to 4.0) | 2.3 (1.9 to 2.7) | 2.3 (1.9 to 2.7) |
| Angola | Both sexes | 0.9 (0.2 to 1.6) | 0.5 (0.3 to 0.7) | 0.6 (0.4 to 0.8) |
|  | Females | 0.6 (-0.1 to 1.3) | 0.4 (0.2 to 0.6) | 0.4 (0.2 to 0.6) |
|  | Males | 1.2 (0.5 to 1.9) | 0.6 (0.4 to 0.9) | 0.7 (0.4 to 0.9) |
| Antigua and Barbuda | Both sexes | 1.3 (0.8 to 1.9) | 1.1 (0.9 to 1.2) | 1.1 (0.9 to 1.2) |
|  | Females | 1.3 (0.7 to 1.8) | 1.0 (0.9 to 1.2) | 1.0 (0.9 to 1.2) |
|  | Males | 1.5 (0.9 to 2.1) | 1.2 (1.1 to 1.4) | 1.2 (1.1 to 1.4) |
| Argentina | Both sexes | 1.6 (0.8 to 2.4) | 0.9 (0.6 to 1.2) | 0.9 (0.6 to 1.2) |
|  | Females | 1.7 (0.9 to 2.4) | 0.9 (0.6 to 1.2) | 0.9 (0.6 to 1.2) |
|  | Males | 1.5 (0.7 to 2.3) | 0.8 (0.4 to 1.1) | 0.8 (0.5 to 1.1) |
| Armenia | Both sexes | 1.1 (0.4 to 1.8) | 0.8 (0.6 to 1.0) | 0.8 (0.6 to 1.0) |
|  | Females | 1.2 (0.5 to 1.9) | 0.9 (0.7 to 1.1) | 0.9 (0.7 to 1.1) |
|  | Males | 1.0 (0.3 to 1.7) | 0.6 (0.3 to 0.8) | 0.6 (0.4 to 0.8) |
| Australia | Both sexes | 3.8 (3.4 to 4.2) | 3.2 (3.0 to 3.4) | 3.1 (2.9 to 3.4) |
|  | Females | 3.9 (3.6 to 4.3) | 3.3 (3.1 to 3.5) | 3.3 (3.1 to 3.5) |
|  | Males | 3.6 (3.1 to 4.0) | 2.9 (2.6 to 3.2) | 2.9 (2.6 to 3.2) |
| Austria | Both sexes | 2.8 (2.2 to 3.5) | 1.8 (1.5 to 2.1) | 1.8 (1.5 to 2.1) |
|  | Females | 3.2 (2.5 to 3.8) | 1.9 (1.6 to 2.2) | 1.9 (1.6 to 2.2) |
|  | Males | 2.4 (1.7 to 3.0) | 1.6 (1.3 to 1.9) | 1.6 (1.3 to 1.9) |
| Azerbaijan | Both sexes | 0.2 (-0.4 to 0.8) | 0.3 (0.2 to 0.4) | 0.3 (0.2 to 0.4) |
|  | Females | -0.0 (-0.7 to 0.6) | 0.3 (0.2 to 0.5) | 0.3 (0.2 to 0.5) |
|  | Males | 0.5 (-0.2 to 1.2) | 0.3 (0.1 to 0.5) | 0.3 (0.1 to 0.5) |
| Bahamas | Both sexes | 1.5 (0.8 to 2.1) | 1.2 (1.0 to 1.4) | 1.2 (1.0 to 1.4) |
|  | Females | 1.4 (0.8 to 2.0) | 1.1 (0.9 to 1.3) | 1.1 (0.9 to 1.3) |
|  | Males | 1.6 (0.9 to 2.2) | 1.3 (1.1 to 1.5) | 1.3 (1.1 to 1.5) |
| Bahrain | Both sexes | -0.5 (-1.2 to 0.1) | -0.5 (-0.6 to -0.3) | -0.5 (-0.7 to -0.4) |
|  | Females | -0.8 (-1.4 to -0.1) | -0.7 (-0.9 to -0.5) | -0.7 (-0.9 to -0.6) |
|  | Males | -0.1 (-0.7 to 0.6) | -0.0 (-0.2 to 0.1) | -0.0 (-0.2 to 0.1) |
| Bangladesh | Both sexes | 1.4 (0.5 to 2.3) | 0.6 (0.2 to 1.0) | 0.6 (0.3 to 1.0) |
|  | Females | 1.2 (0.3 to 2.2) | 0.5 (0.1 to 0.8) | 0.5 (0.1 to 0.9) |
|  | Males | 1.6 (0.7 to 2.4) | 0.7 (0.3 to 1.1) | 0.8 (0.4 to 1.1) |
| Barbados | Both sexes | 1.3 (0.8 to 1.9) | 1.1 (0.9 to 1.3) | 1.1 (0.9 to 1.2) |
|  | Females | 1.3 (0.7 to 1.8) | 1.0 (0.9 to 1.2) | 1.0 (0.9 to 1.2) |
|  | Males | 1.5 (0.9 to 2.0) | 1.2 (1.1 to 1.4) | 1.2 (1.1 to 1.4) |
| Belarus | Both sexes | 0.4 (-0.2 to 1.0) | 0.3 (0.1 to 0.4) | 0.3 (0.1 to 0.4) |
|  | Females | 0.5 (-0.1 to 1.1) | 0.4 (0.2 to 0.6) | 0.4 (0.2 to 0.6) |
|  | Males | 0.3 (-0.3 to 0.9) | 0.1 (-0.1 to 0.3) | 0.1 (-0.0 to 0.3) |
| Belgium | Both sexes | 2.2 (1.6 to 2.9) | 1.4 (1.0 to 1.7) | 1.4 (1.0 to 1.7) |
|  | Females | 2.4 (1.7 to 3.0) | 1.3 (1.0 to 1.6) | 1.3 (1.0 to 1.6) |
|  | Males | 2.1 (1.5 to 2.8) | 1.4 (1.1 to 1.7) | 1.4 (1.1 to 1.7) |
| Belize | Both sexes | 2.0 (1.3 to 2.7) | 1.6 (1.3 to 1.8) | 1.5 (1.3 to 1.8) |
|  | Females | 2.2 (1.5 to 2.9) | 1.6 (1.4 to 1.9) | 1.6 (1.4 to 1.8) |
|  | Males | 1.7 (1.0 to 2.4) | 1.4 (1.1 to 1.6) | 1.4 (1.1 to 1.6) |
| Benin | Both sexes | 1.3 (0.6 to 2.0) | 0.9 (0.7 to 1.1) | 0.9 (0.7 to 1.2) |
|  | Females | 1.1 (0.4 to 1.8) | 1.0 (0.8 to 1.2) | 1.0 (0.8 to 1.2) |
|  | Males | 1.4 (0.7 to 2.1) | 0.7 (0.4 to 1.0) | 0.8 (0.5 to 1.1) |
| Bermuda | Both sexes | 1.3 (0.8 to 1.9) | 1.1 (0.9 to 1.2) | 1.1 (0.9 to 1.2) |
|  | Females | 1.2 (0.7 to 1.8) | 1.0 (0.9 to 1.2) | 1.0 (0.9 to 1.2) |
|  | Males | 1.5 (0.9 to 2.0) | 1.2 (1.0 to 1.4) | 1.2 (1.1 to 1.4) |
| Bhutan | Both sexes | 1.6 (1.1 to 2.0) | 1.0 (0.8 to 1.2) | 1.0 (0.8 to 1.2) |
|  | Females | 1.5 (1.0 to 1.9) | 0.9 (0.8 to 1.1) | 0.9 (0.8 to 1.1) |
|  | Males | 1.6 (1.2 to 2.1) | 1.0 (0.8 to 1.2) | 1.0 (0.8 to 1.2) |
| Bolivia (Plurinational State of) | Both sexes | 1.1 (0.3 to 1.9) | 0.7 (0.4 to 1.0) | 0.7 (0.4 to 1.0) |
|  | Females | 1.0 (0.2 to 1.8) | 0.6 (0.4 to 0.9) | 0.6 (0.4 to 0.9) |
|  | Males | 1.3 (0.5 to 2.2) | 0.8 (0.5 to 1.1) | 0.8 (0.5 to 1.1) |
| Bosnia and Herzegovina | Both sexes | 1.0 (0.3 to 1.6) | 0.7 (0.6 to 0.9) | 0.7 (0.6 to 0.9) |
|  | Females | 1.2 (0.5 to 1.8) | 0.9 (0.8 to 1.1) | 0.9 (0.8 to 1.1) |
|  | Males | 0.6 (-0.0 to 1.3) | 0.4 (0.2 to 0.6) | 0.4 (0.2 to 0.6) |
| Botswana | Both sexes | 1.4 (0.6 to 2.2) | 0.6 (0.3 to 1.0) | 0.7 (0.3 to 1.0) |
|  | Females | 1.6 (0.8 to 2.4) | 0.8 (0.5 to 1.1) | 0.8 (0.5 to 1.1) |
|  | Males | 1.2 (0.4 to 2.0) | 0.5 (0.1 to 0.8) | 0.5 (0.1 to 0.8) |
| Brazil | Both sexes | 1.6 (0.9 to 2.4) | 1.3 (1.0 to 1.5) | 1.3 (1.0 to 1.5) |
|  | Females | 1.6 (0.9 to 2.3) | 1.3 (1.1 to 1.6) | 1.4 (1.1 to 1.6) |
|  | Males | 1.7 (0.9 to 2.5) | 1.1 (0.8 to 1.4) | 1.1 (0.8 to 1.4) |
| Brunei Darussalam | Both sexes | 1.3 (0.8 to 1.8) | 0.9 (0.7 to 1.1) | 0.9 (0.8 to 1.1) |
|  | Females | 1.2 (0.7 to 1.7) | 0.7 (0.5 to 0.9) | 0.7 (0.5 to 0.9) |
|  | Males | 1.5 (1.0 to 2.0) | 1.2 (1.1 to 1.4) | 1.2 (1.1 to 1.4) |
| Bulgaria | Both sexes | 0.6 (-0.0 to 1.3) | 0.4 (0.3 to 0.6) | 0.4 (0.3 to 0.6) |
|  | Females | 0.7 (-0.0 to 1.3) | 0.5 (0.3 to 0.7) | 0.5 (0.3 to 0.7) |
|  | Males | 0.6 (-0.0 to 1.3) | 0.4 (0.2 to 0.6) | 0.4 (0.2 to 0.6) |
| Cabo Verde | Both sexes | 2.0 (1.1 to 2.8) | 1.4 (1.0 to 1.7) | 1.4 (1.0 to 1.7) |
|  | Females | 1.9 (1.0 to 2.7) | 1.5 (1.2 to 1.8) | 1.5 (1.2 to 1.8) |
|  | Males | 2.2 (1.4 to 3.1) | 1.3 (0.9 to 1.7) | 1.3 (0.9 to 1.7) |
| Cambodia | Both sexes | 1.4 (0.7 to 2.0) | 0.5 (0.2 to 0.8) | 0.5 (0.2 to 0.8) |
|  | Females | 1.4 (0.8 to 2.0) | 0.6 (0.3 to 0.9) | 0.6 (0.3 to 0.9) |
|  | Males | 1.4 (0.7 to 2.0) | 0.4 (0.1 to 0.8) | 0.5 (0.1 to 0.8) |
| Cameroon | Both sexes | 1.4 (0.7 to 2.2) | 1.1 (0.8 to 1.3) | 1.1 (0.9 to 1.4) |
|  | Females | 1.3 (0.6 to 2.0) | 1.2 (1.0 to 1.4) | 1.2 (1.0 to 1.4) |
|  | Males | 1.6 (0.9 to 2.4) | 0.9 (0.6 to 1.3) | 1.0 (0.7 to 1.3) |
| Canada | Both sexes | 1.6 (1.0 to 2.1) | 0.8 (0.6 to 1.1) | 0.8 (0.6 to 1.1) |
|  | Females | 1.5 (0.9 to 2.1) | 0.8 (0.5 to 1.0) | 0.8 (0.6 to 1.0) |
|  | Males | 1.7 (1.0 to 2.3) | 1.0 (0.7 to 1.3) | 1.0 (0.7 to 1.3) |
| Chile | Both sexes | 1.3 (0.6 to 2.0) | 0.8 (0.6 to 1.1) | 0.8 (0.6 to 1.1) |
|  | Females | 1.4 (0.7 to 2.1) | 0.9 (0.7 to 1.1) | 0.9 (0.7 to 1.1) |
|  | Males | 1.2 (0.5 to 2.0) | 0.8 (0.5 to 1.0) | 0.8 (0.6 to 1.1) |
| China | Both sexes | 1.2 (0.8 to 1.6) | 0.5 (0.2 to 0.7) | 0.5 (0.2 to 0.7) |
|  | Females | 1.2 (0.8 to 1.7) | 0.5 (0.2 to 0.8) | 0.5 (0.2 to 0.8) |
|  | Males | 1.2 (0.8 to 1.6) | 0.5 (0.3 to 0.7) | 0.5 (0.3 to 0.7) |
| Colombia | Both sexes | 0.9 (0.1 to 1.8) | 0.4 (0.1 to 0.7) | 0.4 (0.1 to 0.7) |
|  | Females | 1.0 (0.2 to 1.8) | 0.4 (0.2 to 0.7) | 0.5 (0.2 to 0.7) |
|  | Males | 0.9 (0.1 to 1.7) | 0.3 (-0.0 to 0.6) | 0.3 (-0.0 to 0.6) |
| Comoros | Both sexes | 2.6 (1.6 to 3.6) | 1.2 (0.7 to 1.8) | 1.2 (0.7 to 1.8) |
|  | Females | 2.5 (1.5 to 3.4) | 1.1 (0.6 to 1.6) | 1.1 (0.6 to 1.6) |
|  | Males | 2.8 (1.8 to 3.8) | 1.4 (0.8 to 2.0) | 1.4 (0.8 to 2.0) |
| Congo | Both sexes | 1.5 (0.7 to 2.2) | 0.8 (0.5 to 1.1) | 0.8 (0.6 to 1.1) |
|  | Females | 1.2 (0.4 to 1.9) | 0.7 (0.5 to 1.0) | 0.7 (0.5 to 1.0) |
|  | Males | 1.9 (1.1 to 2.6) | 1.0 (0.6 to 1.3) | 1.0 (0.6 to 1.3) |
| Costa Rica | Both sexes | 1.9 (1.2 to 2.5) | 1.3 (1.1 to 1.6) | 1.3 (1.1 to 1.6) |
|  | Females | 1.7 (1.0 to 2.4) | 1.3 (1.1 to 1.5) | 1.3 (1.0 to 1.5) |
|  | Males | 2.1 (1.4 to 2.8) | 1.4 (1.0 to 1.7) | 1.4 (1.0 to 1.7) |
| Croatia | Both sexes | 1.2 (0.6 to 1.9) | 0.8 (0.6 to 1.0) | 0.8 (0.6 to 1.0) |
|  | Females | 1.2 (0.6 to 1.9) | 0.9 (0.7 to 1.1) | 0.9 (0.7 to 1.1) |
|  | Males | 1.3 (0.6 to 2.0) | 0.7 (0.4 to 0.9) | 0.7 (0.4 to 0.9) |
| Cuba | Both sexes | 0.1 (-0.5 to 0.7) | 0.3 (0.2 to 0.5) | 0.3 (0.2 to 0.5) |
|  | Females | -0.1 (-0.7 to 0.5) | 0.3 (0.1 to 0.4) | 0.3 (0.1 to 0.4) |
|  | Males | 0.3 (-0.3 to 1.0) | 0.5 (0.3 to 0.7) | 0.5 (0.3 to 0.7) |
| Cyprus | Both sexes | 3.9 (3.3 to 4.5) | 2.2 (1.7 to 2.6) | 2.2 (1.7 to 2.7) |
|  | Females | 4.2 (3.5 to 4.8) | 2.0 (1.5 to 2.5) | 2.0 (1.5 to 2.5) |
|  | Males | 3.5 (2.9 to 4.1) | 2.5 (2.1 to 2.9) | 2.5 (2.1 to 3.0) |
| Czechia | Both sexes | 0.7 (0.1 to 1.3) | 0.5 (0.4 to 0.7) | 0.5 (0.4 to 0.7) |
|  | Females | 0.6 (0.0 to 1.2) | 0.6 (0.5 to 0.8) | 0.6 (0.5 to 0.7) |
|  | Males | 0.9 (0.3 to 1.5) | 0.5 (0.3 to 0.7) | 0.5 (0.3 to 0.7) |
| C么te d'Ivoire | Both sexes | 2.1 (1.4 to 2.8) | 1.5 (1.2 to 1.8) | 1.5 (1.3 to 1.8) |
|  | Females | 1.9 (1.2 to 2.6) | 1.6 (1.3 to 1.8) | 1.6 (1.4 to 1.8) |
|  | Males | 2.4 (1.6 to 3.1) | 1.4 (1.1 to 1.8) | 1.5 (1.1 to 1.8) |
| Denmark | Both sexes | 4.1 (3.5 to 4.6) | 2.3 (1.8 to 2.8) | 2.4 (1.9 to 2.9) |
|  | Females | 4.6 (4.0 to 5.2) | 2.4 (1.8 to 3.0) | 2.4 (1.8 to 3.0) |
|  | Males | 3.2 (2.7 to 3.7) | 2.3 (1.9 to 2.7) | 2.3 (1.9 to 2.7) |
| Djibouti | Both sexes | 3.4 (2.4 to 4.4) | 1.8 (1.2 to 2.4) | 1.8 (1.2 to 2.4) |
|  | Females | 3.6 (2.6 to 4.6) | 1.9 (1.2 to 2.5) | 1.9 (1.2 to 2.5) |
|  | Males | 3.2 (2.3 to 4.2) | 1.8 (1.2 to 2.3) | 1.8 (1.2 to 2.4) |
| Dominica | Both sexes | 1.4 (0.8 to 1.9) | 1.1 (0.9 to 1.3) | 1.1 (0.9 to 1.2) |
|  | Females | 1.3 (0.7 to 1.8) | 1.0 (0.9 to 1.2) | 1.0 (0.9 to 1.2) |
|  | Males | 1.5 (0.9 to 2.1) | 1.2 (1.1 to 1.4) | 1.2 (1.1 to 1.4) |
| Dominican Republic | Both sexes | 1.1 (0.5 to 1.6) | 1.0 (0.8 to 1.1) | 1.0 (0.8 to 1.1) |
|  | Females | 1.0 (0.5 to 1.6) | 0.9 (0.8 to 1.1) | 0.9 (0.8 to 1.0) |
|  | Males | 1.2 (0.7 to 1.8) | 1.1 (1.0 to 1.3) | 1.1 (1.0 to 1.3) |
| Ecuador | Both sexes | 1.4 (0.6 to 2.2) | 0.8 (0.6 to 1.1) | 0.9 (0.6 to 1.1) |
|  | Females | 1.3 (0.5 to 2.1) | 0.8 (0.5 to 1.0) | 0.8 (0.5 to 1.0) |
|  | Males | 1.6 (0.8 to 2.4) | 1.0 (0.6 to 1.3) | 1.0 (0.7 to 1.3) |
| Egypt | Both sexes | 0.8 (0.0 to 1.5) | 0.2 (-0.1 to 0.4) | 0.2 (-0.0 to 0.4) |
|  | Females | 0.4 (-0.3 to 1.1) | -0.1 (-0.3 to 0.1) | -0.1 (-0.3 to 0.1) |
|  | Males | 1.2 (0.5 to 1.9) | 0.6 (0.4 to 0.9) | 0.6 (0.4 to 0.9) |
| El Salvador | Both sexes | 1.8 (1.2 to 2.4) | 1.5 (1.3 to 1.6) | 1.5 (1.3 to 1.6) |
|  | Females | 1.8 (1.2 to 2.4) | 1.5 (1.3 to 1.6) | 1.5 (1.3 to 1.6) |
|  | Males | 1.8 (1.2 to 2.5) | 1.4 (1.1 to 1.6) | 1.4 (1.1 to 1.6) |
| Equatorial Guinea | Both sexes | 1.4 (0.7 to 2.1) | 0.8 (0.5 to 1.0) | 0.8 (0.5 to 1.1) |
|  | Females | 1.2 (0.5 to 1.9) | 0.7 (0.5 to 1.0) | 0.8 (0.5 to 1.0) |
|  | Males | 1.7 (0.9 to 2.4) | 0.9 (0.6 to 1.2) | 0.9 (0.6 to 1.2) |
| Estonia | Both sexes | 0.9 (0.4 to 1.5) | 0.7 (0.5 to 0.8) | 0.7 (0.5 to 0.8) |
|  | Females | 0.9 (0.4 to 1.5) | 0.8 (0.6 to 0.9) | 0.8 (0.6 to 0.9) |
|  | Males | 1.0 (0.4 to 1.5) | 0.6 (0.4 to 0.8) | 0.6 (0.4 to 0.8) |
| Eswatini | Both sexes | 1.3 (0.4 to 2.1) | 0.6 (0.3 to 0.9) | 0.6 (0.2 to 0.9) |
|  | Females | 1.4 (0.5 to 2.2) | 0.7 (0.4 to 1.0) | 0.7 (0.3 to 1.0) |
|  | Males | 1.1 (0.3 to 2.0) | 0.5 (0.2 to 0.8) | 0.5 (0.1 to 0.8) |
| Fiji | Both sexes | 1.8 (1.1 to 2.4) | 1.0 (0.6 to 1.3) | 0.9 (0.6 to 1.3) |
|  | Females | 1.9 (1.2 to 2.5) | 1.1 (0.7 to 1.4) | 1.0 (0.7 to 1.4) |
|  | Males | 1.6 (0.9 to 2.3) | 0.8 (0.5 to 1.2) | 0.8 (0.5 to 1.2) |
| Finland | Both sexes | 2.3 (1.8 to 2.8) | 1.4 (1.1 to 1.7) | 1.4 (1.1 to 1.7) |
|  | Females | 2.4 (1.8 to 2.9) | 1.2 (0.9 to 1.6) | 1.2 (0.9 to 1.5) |
|  | Males | 2.3 (1.8 to 2.8) | 1.6 (1.3 to 1.9) | 1.6 (1.3 to 1.9) |
| France | Both sexes | 2.0 (1.4 to 2.7) | 1.1 (0.8 to 1.4) | 1.1 (0.8 to 1.4) |
|  | Females | 1.9 (1.3 to 2.5) | 0.9 (0.7 to 1.2) | 0.9 (0.7 to 1.2) |
|  | Males | 2.3 (1.6 to 3.0) | 1.4 (1.1 to 1.8) | 1.4 (1.1 to 1.8) |
| Gabon | Both sexes | 1.2 (0.5 to 1.9) | 0.7 (0.4 to 0.9) | 0.7 (0.4 to 0.9) |
|  | Females | 0.9 (0.2 to 1.6) | 0.6 (0.3 to 0.8) | 0.6 (0.3 to 0.8) |
|  | Males | 1.5 (0.8 to 2.2) | 0.8 (0.5 to 1.1) | 0.8 (0.5 to 1.1) |
| Georgia | Both sexes | 1.2 (0.5 to 1.9) | 0.8 (0.6 to 1.0) | 0.8 (0.6 to 1.0) |
|  | Females | 1.4 (0.7 to 2.1) | 1.0 (0.8 to 1.2) | 1.0 (0.8 to 1.2) |
|  | Males | 1.0 (0.3 to 1.7) | 0.5 (0.2 to 0.8) | 0.5 (0.2 to 0.8) |
| Germany | Both sexes | 1.2 (0.5 to 1.9) | -0.1 (-0.4 to 0.2) | -0.1 (-0.4 to 0.2) |
|  | Females | 1.0 (0.2 to 1.7) | -0.6 (-0.9 to -0.2) | -0.5 (-0.9 to -0.2) |
|  | Males | 1.6 (1.0 to 2.2) | 0.8 (0.6 to 1.1) | 0.9 (0.6 to 1.1) |
| Ghana | Both sexes | 1.5 (0.8 to 2.2) | 1.1 (0.9 to 1.4) | 1.2 (0.9 to 1.4) |
|  | Females | 1.2 (0.6 to 1.9) | 1.1 (0.9 to 1.4) | 1.2 (0.9 to 1.4) |
|  | Males | 1.9 (1.2 to 2.6) | 1.1 (0.8 to 1.4) | 1.1 (0.8 to 1.5) |
| Greece | Both sexes | 2.7 (2.1 to 3.3) | 1.8 (1.5 to 2.0) | 1.8 (1.5 to 2.1) |
|  | Females | 2.6 (2.0 to 3.2) | 1.6 (1.3 to 1.8) | 1.6 (1.3 to 1.8) |
|  | Males | 2.9 (2.3 to 3.5) | 2.1 (1.8 to 2.4) | 2.1 (1.8 to 2.4) |
| Greenland | Both sexes | 1.0 (0.3 to 1.8) | 0.5 (0.2 to 0.8) | 0.5 (0.2 to 0.8) |
|  | Females | 0.9 (0.1 to 1.7) | 0.3 (-0.0 to 0.6) | 0.3 (0.0 to 0.7) |
|  | Males | 1.1 (0.4 to 1.9) | 0.6 (0.3 to 0.9) | 0.7 (0.3 to 1.0) |
| Grenada | Both sexes | 1.4 (0.8 to 2.0) | 1.1 (0.9 to 1.3) | 1.1 (0.9 to 1.3) |
|  | Females | 1.4 (0.7 to 2.0) | 1.1 (0.9 to 1.3) | 1.1 (0.9 to 1.2) |
|  | Males | 1.6 (0.9 to 2.2) | 1.3 (1.1 to 1.5) | 1.3 (1.1 to 1.5) |
| Guam | Both sexes | 1.7 (1.1 to 2.4) | 1.0 (0.6 to 1.3) | 0.9 (0.6 to 1.2) |
|  | Females | 1.8 (1.2 to 2.5) | 1.0 (0.7 to 1.4) | 1.0 (0.7 to 1.3) |
|  | Males | 1.6 (0.9 to 2.3) | 0.8 (0.5 to 1.2) | 0.8 (0.5 to 1.1) |
| Guatemala | Both sexes | 2.2 (1.4 to 2.9) | 1.6 (1.3 to 1.9) | 1.6 (1.4 to 1.9) |
|  | Females | 2.1 (1.4 to 2.8) | 1.6 (1.4 to 1.8) | 1.6 (1.4 to 1.8) |
|  | Males | 2.3 (1.5 to 3.0) | 1.6 (1.2 to 1.9) | 1.6 (1.3 to 1.9) |
| Guinea | Both sexes | 1.9 (1.2 to 2.6) | 1.4 (1.2 to 1.7) | 1.4 (1.2 to 1.7) |
|  | Females | 1.8 (1.1 to 2.4) | 1.5 (1.3 to 1.7) | 1.5 (1.3 to 1.7) |
|  | Males | 2.1 (1.4 to 2.8) | 1.3 (1.0 to 1.6) | 1.3 (1.0 to 1.6) |
| Guyana | Both sexes | 1.7 (1.0 to 2.3) | 1.3 (1.1 to 1.5) | 1.3 (1.1 to 1.5) |
|  | Females | 1.7 (1.1 to 2.4) | 1.3 (1.1 to 1.5) | 1.3 (1.1 to 1.5) |
|  | Males | 1.6 (0.9 to 2.2) | 1.3 (1.1 to 1.5) | 1.3 (1.1 to 1.5) |
| Haiti | Both sexes | 1.8 (1.2 to 2.3) | 1.3 (1.1 to 1.5) | 1.3 (1.1 to 1.5) |
|  | Females | 1.5 (1.0 to 2.1) | 1.2 (1.0 to 1.3) | 1.2 (1.0 to 1.3) |
|  | Males | 2.1 (1.5 to 2.7) | 1.6 (1.3 to 1.8) | 1.6 (1.4 to 1.8) |
| Honduras | Both sexes | 2.4 (1.7 to 3.2) | 1.9 (1.6 to 2.1) | 1.9 (1.6 to 2.1) |
|  | Females | 2.4 (1.6 to 3.1) | 1.9 (1.6 to 2.1) | 1.9 (1.6 to 2.1) |
|  | Males | 2.5 (1.8 to 3.3) | 1.8 (1.5 to 2.1) | 1.8 (1.5 to 2.1) |
| Hungary | Both sexes | 0.6 (-0.0 to 1.2) | 0.5 (0.4 to 0.6) | 0.5 (0.4 to 0.6) |
|  | Females | 0.6 (0.0 to 1.2) | 0.6 (0.5 to 0.8) | 0.6 (0.5 to 0.8) |
|  | Males | 0.5 (-0.0 to 1.1) | 0.3 (0.1 to 0.5) | 0.3 (0.2 to 0.5) |
| Iceland | Both sexes | 3.2 (2.6 to 3.9) | 1.6 (1.1 to 2.0) | 1.6 (1.1 to 2.0) |
|  | Females | 3.6 (2.9 to 4.2) | 1.5 (1.0 to 2.0) | 1.5 (1.0 to 2.0) |
|  | Males | 2.7 (2.1 to 3.3) | 1.8 (1.4 to 2.2) | 1.8 (1.4 to 2.2) |
| India | Both sexes | 2.9 (2.0 to 3.7) | 1.7 (1.2 to 2.1) | 1.7 (1.2 to 2.1) |
|  | Females | 3.2 (2.3 to 4.0) | 1.8 (1.4 to 2.3) | 1.9 (1.4 to 2.3) |
|  | Males | 2.4 (1.6 to 3.2) | 1.4 (1.0 to 1.8) | 1.4 (1.0 to 1.8) |
| Indonesia | Both sexes | 1.9 (1.1 to 2.7) | 0.9 (0.5 to 1.3) | 0.9 (0.5 to 1.3) |
|  | Females | 1.9 (1.1 to 2.7) | 1.0 (0.6 to 1.3) | 1.0 (0.6 to 1.4) |
|  | Males | 1.9 (1.0 to 2.7) | 0.9 (0.4 to 1.3) | 0.9 (0.4 to 1.3) |
| Iran (Islamic Republic of) | Both sexes | 1.0 (0.2 to 1.8) | 0.4 (0.2 to 0.7) | 0.4 (0.2 to 0.7) |
|  | Females | 0.7 (-0.0 to 1.5) | 0.2 (-0.0 to 0.4) | 0.2 (-0.0 to 0.4) |
|  | Males | 1.5 (0.6 to 2.3) | 0.8 (0.5 to 1.1) | 0.8 (0.5 to 1.1) |
| Iraq | Both sexes | 1.0 (0.3 to 1.8) | 0.4 (0.1 to 0.6) | 0.4 (0.1 to 0.6) |
|  | Females | 0.8 (0.0 to 1.6) | 0.1 (-0.1 to 0.4) | 0.1 (-0.1 to 0.4) |
|  | Males | 1.4 (0.7 to 2.1) | 0.8 (0.5 to 1.0) | 0.8 (0.5 to 1.0) |
| Ireland | Both sexes | 3.8 (3.2 to 4.4) | 2.6 (2.3 to 3.0) | 2.7 (2.3 to 3.0) |
|  | Females | 4.0 (3.4 to 4.6) | 2.7 (2.3 to 3.0) | 2.7 (2.4 to 3.0) |
|  | Males | 3.5 (2.9 to 4.1) | 2.6 (2.3 to 3.0) | 2.6 (2.3 to 3.0) |
| Israel | Both sexes | 2.2 (1.7 to 2.8) | 1.6 (1.4 to 1.9) | 1.6 (1.4 to 1.9) |
|  | Females | 2.4 (1.9 to 2.9) | 1.8 (1.5 to 2.0) | 1.8 (1.6 to 2.0) |
|  | Males | 2.1 (1.5 to 2.7) | 1.5 (1.3 to 1.8) | 1.5 (1.3 to 1.8) |
| Italy | Both sexes | 2.7 (2.1 to 3.3) | 1.6 (1.3 to 1.9) | 1.6 (1.3 to 2.0) |
|  | Females | 2.7 (2.1 to 3.3) | 1.5 (1.2 to 1.9) | 1.6 (1.2 to 1.9) |
|  | Males | 2.7 (2.1 to 3.3) | 1.8 (1.5 to 2.2) | 1.8 (1.5 to 2.2) |
| Jamaica | Both sexes | 2.3 (1.7 to 2.8) | 1.9 (1.8 to 2.1) | 1.9 (1.7 to 2.1) |
|  | Females | 2.4 (1.8 to 2.9) | 2.0 (1.8 to 2.2) | 2.0 (1.8 to 2.2) |
|  | Males | 2.1 (1.6 to 2.7) | 1.8 (1.6 to 2.0) | 1.8 (1.6 to 2.0) |
| Japan | Both sexes | 1.2 (0.8 to 1.7) | 0.6 (0.4 to 0.8) | 0.6 (0.4 to 0.8) |
|  | Females | 1.2 (0.8 to 1.7) | 0.5 (0.3 to 0.7) | 0.6 (0.3 to 0.7) |
|  | Males | 1.3 (0.9 to 1.7) | 0.8 (0.6 to 0.9) | 0.8 (0.6 to 0.9) |
| Jordan | Both sexes | 0.8 (0.1 to 1.6) | 0.3 (0.0 to 0.5) | 0.2 (0.0 to 0.5) |
|  | Females | 0.7 (-0.1 to 1.4) | 0.1 (-0.1 to 0.3) | 0.1 (-0.1 to 0.3) |
|  | Males | 1.0 (0.3 to 1.7) | 0.5 (0.3 to 0.8) | 0.5 (0.3 to 0.8) |
| Kazakhstan | Both sexes | 1.4 (0.7 to 2.0) | 0.9 (0.7 to 1.2) | 1.0 (0.8 to 1.2) |
|  | Females | 1.3 (0.6 to 1.9) | 1.0 (0.8 to 1.2) | 1.0 (0.8 to 1.2) |
|  | Males | 1.5 (0.8 to 2.2) | 0.9 (0.6 to 1.2) | 1.0 (0.7 to 1.2) |
| Kenya | Both sexes | 1.6 (0.7 to 2.4) | 0.7 (0.3 to 1.0) | 0.7 (0.3 to 1.0) |
|  | Females | 1.7 (0.8 to 2.6) | 0.7 (0.3 to 1.1) | 0.7 (0.3 to 1.1) |
|  | Males | 1.4 (0.6 to 2.1) | 0.6 (0.3 to 1.0) | 0.6 (0.3 to 1.0) |
| Kiribati | Both sexes | 1.8 (1.1 to 2.5) | 1.0 (0.7 to 1.3) | 1.0 (0.7 to 1.3) |
|  | Females | 1.8 (1.2 to 2.5) | 1.1 (0.7 to 1.4) | 1.0 (0.7 to 1.4) |
|  | Males | 1.6 (0.9 to 2.3) | 0.9 (0.5 to 1.2) | 0.9 (0.5 to 1.2) |
| Kuwait | Both sexes | 1.3 (0.6 to 2.1) | 0.5 (0.2 to 0.8) | 0.5 (0.2 to 0.7) |
|  | Females | 1.1 (0.4 to 1.9) | 0.3 (-0.0 to 0.5) | 0.2 (-0.0 to 0.5) |
|  | Males | 1.6 (0.9 to 2.4) | 0.9 (0.6 to 1.2) | 0.9 (0.6 to 1.1) |
| Kyrgyzstan | Both sexes | 1.0 (0.2 to 1.8) | 0.6 (0.4 to 0.9) | 0.7 (0.4 to 0.9) |
|  | Females | 1.2 (0.4 to 2.0) | 0.9 (0.6 to 1.1) | 0.9 (0.6 to 1.1) |
|  | Males | 0.7 (-0.1 to 1.5) | 0.3 (0.0 to 0.6) | 0.3 (0.0 to 0.6) |
| Lao People's Democratic Republic | Both sexes | 2.9 (2.2 to 3.5) | 1.7 (1.3 to 2.1) | 1.7 (1.3 to 2.1) |
|  | Females | 3.1 (2.4 to 3.8) | 1.9 (1.4 to 2.3) | 1.9 (1.4 to 2.3) |
|  | Males | 2.6 (2.0 to 3.2) | 1.5 (1.1 to 1.9) | 1.6 (1.2 to 1.9) |
| Latvia | Both sexes | 0.8 (0.2 to 1.4) | 0.6 (0.4 to 0.7) | 0.6 (0.4 to 0.8) |
|  | Females | 0.8 (0.2 to 1.4) | 0.6 (0.4 to 0.8) | 0.6 (0.5 to 0.8) |
|  | Males | 1.0 (0.3 to 1.6) | 0.6 (0.4 to 0.8) | 0.6 (0.4 to 0.8) |
| Lebanon | Both sexes | 0.9 (0.1 to 1.8) | 0.2 (-0.1 to 0.4) | 0.2 (-0.1 to 0.4) |
|  | Females | 0.4 (-0.4 to 1.2) | -0.2 (-0.5 to 0.0) | -0.2 (-0.5 to 0.0) |
|  | Males | 1.8 (1.0 to 2.7) | 0.9 (0.6 to 1.3) | 0.9 (0.6 to 1.3) |
| Lesotho | Both sexes | 2.4 (1.5 to 3.4) | 1.2 (0.8 to 1.7) | 1.2 (0.7 to 1.7) |
|  | Females | 2.4 (1.4 to 3.4) | 1.2 (0.8 to 1.7) | 1.2 (0.7 to 1.7) |
|  | Males | 2.5 (1.6 to 3.5) | 1.3 (0.8 to 1.8) | 1.2 (0.7 to 1.8) |
| Libya | Both sexes | 0.5 (-0.2 to 1.2) | 0.0 (-0.2 to 0.2) | -0.0 (-0.2 to 0.2) |
|  | Females | 0.2 (-0.5 to 1.0) | -0.2 (-0.4 to -0.0) | -0.3 (-0.5 to -0.1) |
|  | Males | 0.9 (0.2 to 1.5) | 0.4 (0.2 to 0.6) | 0.4 (0.2 to 0.6) |
| Lithuania | Both sexes | 1.3 (0.6 to 1.9) | 0.9 (0.7 to 1.0) | 0.9 (0.7 to 1.1) |
|  | Females | 1.3 (0.7 to 1.9) | 0.9 (0.7 to 1.1) | 0.9 (0.8 to 1.1) |
|  | Males | 1.2 (0.6 to 1.8) | 0.8 (0.6 to 1.0) | 0.8 (0.6 to 1.0) |
| Luxembourg | Both sexes | 3.2 (2.5 to 4.0) | 1.6 (1.1 to 2.1) | 1.6 (1.1 to 2.1) |
|  | Females | 3.5 (2.8 to 4.2) | 1.4 (0.9 to 1.9) | 1.4 (0.9 to 2.0) |
|  | Males | 3.0 (2.3 to 3.6) | 1.9 (1.5 to 2.4) | 2.0 (1.6 to 2.4) |
| Malaysia | Both sexes | 1.4 (0.7 to 2.0) | 0.5 (0.2 to 0.8) | 0.5 (0.2 to 0.8) |
|  | Females | 1.5 (0.9 to 2.2) | 0.7 (0.3 to 1.0) | 0.6 (0.3 to 1.0) |
|  | Males | 1.2 (0.6 to 1.9) | 0.4 (0.0 to 0.7) | 0.4 (0.1 to 0.7) |
| Maldives | Both sexes | 1.4 (0.8 to 2.1) | 0.4 (0.1 to 0.8) | 0.5 (0.1 to 0.8) |
|  | Females | 1.7 (1.0 to 2.3) | 0.7 (0.4 to 1.0) | 0.7 (0.4 to 1.1) |
|  | Males | 1.4 (0.7 to 2.1) | 0.4 (0.0 to 0.8) | 0.4 (0.1 to 0.8) |
| Malta | Both sexes | 4.1 (3.4 to 4.7) | 2.2 (1.8 to 2.7) | 2.3 (1.8 to 2.7) |
|  | Females | 4.4 (3.7 to 5.1) | 2.1 (1.6 to 2.7) | 2.2 (1.6 to 2.7) |
|  | Males | 3.6 (3.0 to 4.2) | 2.5 (2.1 to 3.0) | 2.6 (2.2 to 3.0) |
| Marshall Islands | Both sexes | 1.8 (1.1 to 2.4) | 1.0 (0.6 to 1.3) | 1.0 (0.6 to 1.3) |
|  | Females | 1.8 (1.2 to 2.5) | 1.1 (0.7 to 1.4) | 1.0 (0.7 to 1.4) |
|  | Males | 1.6 (0.9 to 2.3) | 0.8 (0.5 to 1.2) | 0.8 (0.5 to 1.2) |
| Mauritania | Both sexes | 2.0 (1.1 to 3.0) | 1.3 (0.9 to 1.7) | 1.3 (0.9 to 1.7) |
|  | Females | 2.0 (1.1 to 3.0) | 1.5 (1.1 to 1.9) | 1.5 (1.1 to 1.9) |
|  | Males | 2.0 (1.0 to 2.9) | 1.1 (0.6 to 1.5) | 1.1 (0.6 to 1.5) |
| Mauritius | Both sexes | 0.3 (-0.2 to 0.9) | -0.1 (-0.2 to 0.1) | -0.1 (-0.3 to 0.1) |
|  | Females | 0.4 (-0.2 to 0.9) | 0.1 (-0.1 to 0.2) | 0.0 (-0.1 to 0.2) |
|  | Males | 0.2 (-0.3 to 0.8) | -0.2 (-0.5 to -0.0) | -0.3 (-0.5 to -0.0) |
| Mexico | Both sexes | 2.5 (1.8 to 3.2) | 2.1 (1.9 to 2.3) | 2.1 (1.9 to 2.4) |
|  | Females | 2.6 (1.9 to 3.3) | 2.2 (2.0 to 2.4) | 2.2 (2.0 to 2.4) |
|  | Males | 2.4 (1.7 to 3.1) | 1.9 (1.6 to 2.2) | 1.9 (1.7 to 2.2) |
| Micronesia (Federated States of) | Both sexes | 1.8 (1.1 to 2.4) | 1.0 (0.6 to 1.3) | 0.9 (0.6 to 1.3) |
|  | Females | 1.9 (1.2 to 2.5) | 1.1 (0.7 to 1.4) | 1.0 (0.7 to 1.4) |
|  | Males | 1.6 (0.9 to 2.3) | 0.9 (0.5 to 1.2) | 0.8 (0.5 to 1.1) |
| Monaco | Both sexes | 3.8 (3.1 to 4.5) | 2.0 (1.5 to 2.5) | 2.0 (1.6 to 2.5) |
|  | Females | 4.1 (3.4 to 4.8) | 1.9 (1.4 to 2.4) | 1.9 (1.4 to 2.4) |
|  | Males | 3.3 (2.7 to 4.0) | 2.3 (1.9 to 2.7) | 2.3 (1.9 to 2.7) |
| Mongolia | Both sexes | 1.4 (0.8 to 2.0) | 0.9 (0.7 to 1.1) | 1.0 (0.8 to 1.2) |
|  | Females | 1.3 (0.7 to 1.9) | 0.9 (0.8 to 1.1) | 1.0 (0.8 to 1.1) |
|  | Males | 1.6 (1.0 to 2.2) | 0.9 (0.7 to 1.2) | 0.9 (0.7 to 1.2) |
| Montenegro | Both sexes | 1.4 (0.6 to 2.1) | 0.9 (0.7 to 1.1) | 0.9 (0.6 to 1.1) |
|  | Females | 1.6 (0.8 to 2.3) | 1.1 (0.9 to 1.3) | 1.1 (0.9 to 1.3) |
|  | Males | 1.1 (0.3 to 1.8) | 0.6 (0.3 to 0.8) | 0.6 (0.3 to 0.8) |
| Morocco | Both sexes | 0.4 (-0.3 to 1.2) | -0.2 (-0.4 to 0.1) | -0.2 (-0.4 to 0.1) |
|  | Females | 0.1 (-0.7 to 0.9) | -0.5 (-0.7 to -0.2) | -0.5 (-0.7 to -0.2) |
|  | Males | 0.9 (0.2 to 1.6) | 0.3 (0.1 to 0.6) | 0.3 (0.1 to 0.6) |
| Myanmar | Both sexes | 1.9 (1.1 to 2.6) | 0.8 (0.4 to 1.2) | 0.8 (0.4 to 1.2) |
|  | Females | 1.9 (1.1 to 2.7) | 0.8 (0.4 to 1.2) | 0.8 (0.4 to 1.2) |
|  | Males | 1.7 (0.9 to 2.5) | 0.6 (0.2 to 1.1) | 0.6 (0.2 to 1.1) |
| Namibia | Both sexes | 2.4 (1.6 to 3.2) | 1.4 (1.0 to 1.8) | 1.4 (1.0 to 1.8) |
|  | Females | 2.6 (1.8 to 3.5) | 1.6 (1.2 to 2.0) | 1.6 (1.2 to 2.0) |
|  | Males | 2.1 (1.3 to 2.9) | 1.2 (0.8 to 1.6) | 1.2 (0.8 to 1.6) |
| Nauru | Both sexes | 1.8 (1.1 to 2.5) | 1.0 (0.7 to 1.4) | 1.0 (0.7 to 1.3) |
|  | Females | 1.9 (1.2 to 2.6) | 1.1 (0.7 to 1.4) | 1.0 (0.7 to 1.4) |
|  | Males | 1.7 (0.9 to 2.4) | 0.9 (0.5 to 1.2) | 0.9 (0.5 to 1.2) |
| Nepal | Both sexes | 3.1 (2.2 to 3.9) | 1.8 (1.4 to 2.3) | 1.9 (1.4 to 2.3) |
|  | Females | 3.1 (2.3 to 4.0) | 1.8 (1.4 to 2.3) | 1.9 (1.5 to 2.4) |
|  | Males | 3.0 (2.1 to 3.8) | 1.7 (1.2 to 2.2) | 1.8 (1.3 to 2.2) |
| Netherlands | Both sexes | 2.7 (1.9 to 3.5) | 1.5 (1.1 to 1.9) | 1.5 (1.1 to 1.9) |
|  | Females | 2.8 (2.0 to 3.6) | 1.4 (1.0 to 1.8) | 1.4 (1.0 to 1.8) |
|  | Males | 2.5 (1.7 to 3.2) | 1.6 (1.2 to 2.0) | 1.6 (1.2 to 2.0) |
| New Zealand | Both sexes | 0.6 (0.2 to 1.0) | 0.6 (0.5 to 0.7) | 0.6 (0.5 to 0.7) |
|  | Females | 0.7 (0.3 to 1.1) | 0.7 (0.6 to 0.8) | 0.7 (0.6 to 0.8) |
|  | Males | 0.4 (0.0 to 0.8) | 0.6 (0.4 to 0.7) | 0.6 (0.4 to 0.7) |
| Nicaragua | Both sexes | 1.4 (0.7 to 2.2) | 1.1 (0.9 to 1.3) | 1.1 (0.9 to 1.3) |
|  | Females | 1.4 (0.6 to 2.0) | 1.1 (0.9 to 1.3) | 1.1 (0.9 to 1.3) |
|  | Males | 1.7 (0.9 to 2.4) | 1.1 (0.8 to 1.4) | 1.1 (0.8 to 1.4) |
| Nigeria | Both sexes | 3.0 (2.3 to 3.8) | 2.2 (1.8 to 2.5) | 2.2 (1.9 to 2.5) |
|  | Females | 3.3 (2.6 to 4.0) | 2.4 (2.1 to 2.7) | 2.4 (2.1 to 2.7) |
|  | Males | 2.6 (1.9 to 3.4) | 1.7 (1.4 to 2.1) | 1.8 (1.4 to 2.1) |
| North Macedonia | Both sexes | 1.6 (0.9 to 2.3) | 1.1 (0.8 to 1.3) | 1.1 (0.8 to 1.3) |
|  | Females | 1.7 (1.0 to 2.4) | 1.2 (1.0 to 1.5) | 1.2 (1.0 to 1.4) |
|  | Males | 1.4 (0.6 to 2.1) | 0.8 (0.5 to 1.1) | 0.8 (0.5 to 1.1) |
| Northern Mariana Islands | Both sexes | 1.7 (1.1 to 2.4) | 0.9 (0.6 to 1.3) | 0.9 (0.6 to 1.2) |
|  | Females | 1.8 (1.2 to 2.5) | 1.0 (0.7 to 1.4) | 1.0 (0.7 to 1.4) |
|  | Males | 1.6 (0.9 to 2.3) | 0.8 (0.5 to 1.2) | 0.8 (0.5 to 1.1) |
| Norway | Both sexes | 2.2 (1.6 to 2.8) | 1.0 (0.7 to 1.3) | 1.0 (0.7 to 1.3) |
|  | Females | 2.2 (1.6 to 2.8) | 0.8 (0.5 to 1.1) | 0.8 (0.5 to 1.1) |
|  | Males | 2.2 (1.7 to 2.8) | 1.4 (1.1 to 1.7) | 1.4 (1.1 to 1.7) |
| Oman | Both sexes | 0.5 (-0.3 to 1.3) | -0.0 (-0.3 to 0.2) | -0.0 (-0.3 to 0.2) |
|  | Females | -0.0 (-0.8 to 0.7) | -0.4 (-0.6 to -0.2) | -0.4 (-0.7 to -0.2) |
|  | Males | 1.2 (0.4 to 1.9) | 0.5 (0.2 to 0.8) | 0.5 (0.2 to 0.8) |
| Pakistan | Both sexes | 1.9 (1.1 to 2.6) | 1.1 (0.8 to 1.4) | 1.1 (0.8 to 1.4) |
|  | Females | 1.4 (0.7 to 2.2) | 0.8 (0.6 to 1.1) | 0.8 (0.6 to 1.1) |
|  | Males | 2.4 (1.6 to 3.2) | 1.5 (1.1 to 1.8) | 1.5 (1.1 to 1.8) |
| Palau | Both sexes | 1.7 (1.0 to 2.4) | 0.9 (0.6 to 1.3) | 0.9 (0.6 to 1.2) |
|  | Females | 1.8 (1.2 to 2.5) | 1.1 (0.7 to 1.4) | 1.0 (0.7 to 1.3) |
|  | Males | 1.6 (0.9 to 2.3) | 0.8 (0.5 to 1.2) | 0.8 (0.5 to 1.1) |
| Palestine | Both sexes | -0.1 (-0.8 to 0.6) | -0.3 (-0.4 to -0.1) | -0.3 (-0.5 to -0.1) |
|  | Females | -0.5 (-1.1 to 0.2) | -0.6 (-0.8 to -0.4) | -0.6 (-0.8 to -0.4) |
|  | Males | 0.3 (-0.3 to 1.0) | 0.1 (-0.0 to 0.3) | 0.1 (-0.1 to 0.3) |
| Panama | Both sexes | 2.0 (1.3 to 2.8) | 1.4 (1.2 to 1.7) | 1.4 (1.1 to 1.7) |
|  | Females | 1.8 (1.1 to 2.5) | 1.4 (1.1 to 1.6) | 1.4 (1.1 to 1.6) |
|  | Males | 2.4 (1.6 to 3.2) | 1.5 (1.1 to 1.9) | 1.5 (1.1 to 1.9) |
| Papua New Guinea | Both sexes | 1.6 (1.0 to 2.2) | 0.9 (0.6 to 1.2) | 0.9 (0.6 to 1.2) |
|  | Females | 1.7 (1.1 to 2.3) | 1.0 (0.7 to 1.3) | 1.0 (0.7 to 1.3) |
|  | Males | 1.5 (0.9 to 2.1) | 0.8 (0.5 to 1.1) | 0.8 (0.5 to 1.1) |
| Paraguay | Both sexes | 0.6 (-0.1 to 1.4) | 0.6 (0.4 to 0.8) | 0.6 (0.3 to 0.8) |
|  | Females | 0.6 (-0.2 to 1.4) | 0.7 (0.4 to 0.9) | 0.7 (0.4 to 0.9) |
|  | Males | 0.7 (-0.1 to 1.5) | 0.4 (0.2 to 0.7) | 0.4 (0.1 to 0.7) |
| Peru | Both sexes | 0.9 (0.0 to 1.7) | 0.4 (0.1 to 0.7) | 0.4 (0.2 to 0.7) |
|  | Females | 0.9 (0.1 to 1.7) | 0.4 (0.2 to 0.7) | 0.4 (0.2 to 0.7) |
|  | Males | 0.9 (0.1 to 1.6) | 0.5 (0.2 to 0.7) | 0.5 (0.2 to 0.7) |
| Philippines | Both sexes | 1.2 (0.4 to 1.9) | 0.4 (0.0 to 0.7) | 0.4 (0.0 to 0.7) |
|  | Females | 1.4 (0.6 to 2.1) | 0.5 (0.2 to 0.9) | 0.5 (0.2 to 0.8) |
|  | Males | 0.9 (0.2 to 1.7) | 0.1 (-0.2 to 0.5) | 0.1 (-0.2 to 0.5) |
| Poland | Both sexes | 0.5 (-0.2 to 1.1) | 0.4 (0.2 to 0.5) | 0.4 (0.2 to 0.5) |
|  | Females | 0.5 (-0.1 to 1.2) | 0.5 (0.3 to 0.6) | 0.5 (0.3 to 0.6) |
|  | Males | 0.4 (-0.2 to 1.1) | 0.2 (-0.0 to 0.4) | 0.2 (-0.0 to 0.4) |
| Portugal | Both sexes | 2.4 (1.7 to 3.0) | 1.5 (1.2 to 1.7) | 1.5 (1.2 to 1.7) |
|  | Females | 2.4 (1.8 to 3.1) | 1.4 (1.2 to 1.7) | 1.4 (1.2 to 1.7) |
|  | Males | 2.3 (1.6 to 2.9) | 1.6 (1.3 to 1.8) | 1.6 (1.3 to 1.9) |
| Puerto Rico | Both sexes | 1.3 (0.8 to 1.8) | 1.1 (1.0 to 1.3) | 1.1 (1.0 to 1.3) |
|  | Females | 1.4 (0.9 to 1.9) | 1.1 (1.0 to 1.3) | 1.1 (1.0 to 1.3) |
|  | Males | 1.1 (0.6 to 1.7) | 1.1 (0.9 to 1.2) | 1.1 (0.9 to 1.2) |
| Qatar | Both sexes | 0.9 (0.2 to 1.7) | -0.0 (-0.4 to 0.3) | -0.0 (-0.4 to 0.3) |
|  | Females | 0.8 (0.1 to 1.6) | -0.2 (-0.5 to 0.1) | -0.2 (-0.5 to 0.1) |
|  | Males | 1.1 (0.3 to 1.8) | 0.2 (-0.1 to 0.5) | 0.2 (-0.1 to 0.5) |
| Republic of Korea | Both sexes | 0.7 (0.2 to 1.2) | 0.5 (0.3 to 0.6) | 0.5 (0.3 to 0.6) |
|  | Females | 0.7 (0.2 to 1.2) | 0.4 (0.3 to 0.6) | 0.4 (0.3 to 0.6) |
|  | Males | 0.8 (0.3 to 1.2) | 0.6 (0.5 to 0.8) | 0.7 (0.5 to 0.8) |
| Republic of Moldova | Both sexes | 1.3 (0.6 to 2.1) | 0.8 (0.6 to 1.1) | 0.9 (0.6 to 1.1) |
|  | Females | 1.4 (0.7 to 2.2) | 1.0 (0.7 to 1.2) | 1.0 (0.7 to 1.2) |
|  | Males | 1.2 (0.4 to 2.0) | 0.7 (0.4 to 1.0) | 0.7 (0.4 to 1.0) |
| Romania | Both sexes | 0.8 (0.1 to 1.5) | 0.5 (0.3 to 0.7) | 0.5 (0.3 to 0.7) |
|  | Females | 0.9 (0.2 to 1.6) | 0.6 (0.4 to 0.8) | 0.6 (0.4 to 0.8) |
|  | Males | 0.6 (-0.1 to 1.3) | 0.2 (0.0 to 0.5) | 0.2 (0.0 to 0.5) |
| Russian Federation | Both sexes | 0.8 (0.0 to 1.6) | 0.4 (0.2 to 0.7) | 0.5 (0.2 to 0.7) |
|  | Females | 0.9 (0.1 to 1.7) | 0.6 (0.3 to 0.8) | 0.6 (0.3 to 0.8) |
|  | Males | 0.7 (-0.2 to 1.5) | 0.2 (-0.0 to 0.5) | 0.3 (0.0 to 0.6) |
| Saint Kitts and Nevis | Both sexes | 1.4 (0.8 to 2.0) | 1.1 (1.0 to 1.3) | 1.2 (1.0 to 1.3) |
|  | Females | 1.3 (0.7 to 1.9) | 1.1 (0.9 to 1.2) | 1.1 (0.9 to 1.2) |
|  | Males | 1.5 (0.9 to 2.1) | 1.3 (1.1 to 1.4) | 1.3 (1.1 to 1.4) |
| Saint Lucia | Both sexes | 1.4 (0.8 to 2.0) | 1.1 (0.9 to 1.3) | 1.1 (0.9 to 1.3) |
|  | Females | 1.3 (0.7 to 1.9) | 1.1 (0.9 to 1.2) | 1.1 (0.9 to 1.2) |
|  | Males | 1.6 (0.9 to 2.2) | 1.3 (1.1 to 1.5) | 1.3 (1.1 to 1.5) |
| Saint Vincent and the Grenadines | Both sexes | 1.3 (0.8 to 1.8) | 1.0 (0.9 to 1.2) | 1.1 (0.9 to 1.2) |
|  | Females | 1.2 (0.7 to 1.7) | 1.0 (0.9 to 1.1) | 1.0 (0.9 to 1.1) |
|  | Males | 1.4 (0.9 to 1.9) | 1.2 (1.0 to 1.4) | 1.2 (1.0 to 1.4) |
| Samoa | Both sexes | 1.8 (1.1 to 2.5) | 1.0 (0.7 to 1.3) | 1.0 (0.6 to 1.3) |
|  | Females | 1.9 (1.2 to 2.5) | 1.1 (0.7 to 1.4) | 1.0 (0.7 to 1.4) |
|  | Males | 1.6 (0.9 to 2.3) | 0.9 (0.5 to 1.2) | 0.8 (0.5 to 1.2) |
| San Marino | Both sexes | 3.8 (3.1 to 4.6) | 2.1 (1.6 to 2.6) | 2.1 (1.6 to 2.6) |
|  | Females | 4.2 (3.4 to 5.0) | 2.0 (1.4 to 2.5) | 2.0 (1.4 to 2.5) |
|  | Males | 3.4 (2.7 to 4.1) | 2.4 (1.9 to 2.8) | 2.4 (1.9 to 2.8) |
| Sao Tome and Principe | Both sexes | 2.0 (1.2 to 2.8) | 1.4 (1.1 to 1.8) | 1.4 (1.1 to 1.8) |
|  | Females | 1.9 (1.0 to 2.7) | 1.5 (1.2 to 1.8) | 1.5 (1.2 to 1.8) |
|  | Males | 2.2 (1.4 to 3.1) | 1.3 (0.9 to 1.7) | 1.3 (0.9 to 1.7) |
| Saudi Arabia | Both sexes | 1.4 (0.8 to 2.1) | 0.8 (0.6 to 1.1) | 0.8 (0.6 to 1.0) |
|  | Females | 1.2 (0.6 to 1.9) | 0.7 (0.5 to 0.9) | 0.7 (0.5 to 0.9) |
|  | Males | 1.9 (1.2 to 2.5) | 1.2 (1.0 to 1.5) | 1.2 (0.9 to 1.5) |
| Senegal | Both sexes | 1.7 (0.9 to 2.5) | 1.2 (0.9 to 1.5) | 1.2 (0.9 to 1.6) |
|  | Females | 1.9 (1.0 to 2.7) | 1.5 (1.2 to 1.8) | 1.5 (1.2 to 1.8) |
|  | Males | 1.6 (0.8 to 2.4) | 0.9 (0.6 to 1.2) | 0.9 (0.6 to 1.2) |
| Serbia | Both sexes | 0.9 (0.2 to 1.5) | 0.7 (0.5 to 0.9) | 0.7 (0.5 to 0.8) |
|  | Females | 1.1 (0.4 to 1.7) | 0.9 (0.7 to 1.0) | 0.9 (0.7 to 1.0) |
|  | Males | 0.7 (0.0 to 1.3) | 0.5 (0.3 to 0.6) | 0.5 (0.3 to 0.6) |
| Seychelles | Both sexes | 1.6 (0.9 to 2.4) | 0.6 (0.2 to 1.0) | 0.6 (0.2 to 1.0) |
|  | Females | 1.8 (1.0 to 2.6) | 0.8 (0.4 to 1.2) | 0.8 (0.4 to 1.1) |
|  | Males | 1.5 (0.7 to 2.3) | 0.5 (0.1 to 0.9) | 0.5 (0.1 to 0.9) |
| Singapore | Both sexes | 1.0 (0.6 to 1.4) | 0.8 (0.7 to 0.9) | 0.8 (0.7 to 0.9) |
|  | Females | 0.7 (0.3 to 1.1) | 0.5 (0.4 to 0.6) | 0.5 (0.4 to 0.6) |
|  | Males | 1.4 (1.0 to 1.8) | 1.2 (1.1 to 1.4) | 1.2 (1.1 to 1.4) |
| Slovakia | Both sexes | 0.9 (0.2 to 1.6) | 0.6 (0.4 to 0.8) | 0.6 (0.4 to 0.8) |
|  | Females | 1.1 (0.5 to 1.8) | 0.9 (0.7 to 1.0) | 0.9 (0.7 to 1.0) |
|  | Males | 0.5 (-0.2 to 1.2) | 0.2 (-0.0 to 0.4) | 0.2 (-0.0 to 0.4) |
| Slovenia | Both sexes | 1.0 (0.3 to 1.6) | 0.6 (0.4 to 0.8) | 0.6 (0.4 to 0.8) |
|  | Females | 1.3 (0.6 to 1.9) | 0.9 (0.7 to 1.1) | 0.9 (0.7 to 1.1) |
|  | Males | 0.5 (-0.1 to 1.2) | 0.2 (0.0 to 0.4) | 0.2 (0.0 to 0.4) |
| Solomon Islands | Both sexes | 1.7 (1.0 to 2.3) | 0.9 (0.6 to 1.2) | 0.9 (0.6 to 1.2) |
|  | Females | 1.8 (1.1 to 2.4) | 1.0 (0.7 to 1.3) | 1.0 (0.7 to 1.3) |
|  | Males | 1.6 (0.9 to 2.2) | 0.8 (0.5 to 1.1) | 0.8 (0.5 to 1.1) |
| South Africa | Both sexes | 1.8 (0.9 to 2.7) | 0.9 (0.5 to 1.3) | 0.9 (0.5 to 1.3) |
|  | Females | 1.7 (0.8 to 2.6) | 0.8 (0.4 to 1.2) | 0.8 (0.4 to 1.2) |
|  | Males | 2.1 (1.2 to 3.0) | 1.1 (0.6 to 1.5) | 1.0 (0.6 to 1.5) |
| Spain | Both sexes | 3.2 (2.6 to 3.8) | 2.3 (1.9 to 2.6) | 2.3 (2.0 to 2.6) |
|  | Females | 3.2 (2.6 to 3.8) | 2.2 (1.9 to 2.4) | 2.2 (1.9 to 2.4) |
|  | Males | 3.3 (2.7 to 3.9) | 2.5 (2.1 to 2.8) | 2.5 (2.1 to 2.8) |
| Sri Lanka | Both sexes | 2.2 (1.3 to 3.0) | 0.9 (0.4 to 1.4) | 0.9 (0.4 to 1.4) |
|  | Females | 2.3 (1.5 to 3.2) | 1.0 (0.5 to 1.5) | 1.0 (0.5 to 1.5) |
|  | Males | 1.8 (0.9 to 2.8) | 0.5 (0.0 to 1.1) | 0.6 (0.0 to 1.1) |
| Suriname | Both sexes | 1.2 (0.5 to 1.9) | 1.0 (0.8 to 1.2) | 1.0 (0.8 to 1.2) |
|  | Females | 1.1 (0.4 to 1.8) | 0.9 (0.7 to 1.1) | 0.9 (0.6 to 1.1) |
|  | Males | 1.4 (0.7 to 2.1) | 1.1 (0.9 to 1.4) | 1.1 (0.9 to 1.4) |
| Sweden | Both sexes | 3.8 (3.1 to 4.6) | 2.1 (1.6 to 2.6) | 2.1 (1.6 to 2.6) |
|  | Females | 4.6 (3.9 to 5.4) | 2.2 (1.6 to 2.9) | 2.3 (1.6 to 2.9) |
|  | Males | 2.7 (2.1 to 3.4) | 1.8 (1.5 to 2.2) | 1.8 (1.4 to 2.2) |
| Switzerland | Both sexes | 1.6 (1.0 to 2.1) | 1.2 (1.0 to 1.3) | 1.2 (1.0 to 1.4) |
|  | Females | 1.9 (1.4 to 2.5) | 1.4 (1.2 to 1.5) | 1.4 (1.2 to 1.5) |
|  | Males | 1.1 (0.5 to 1.6) | 0.9 (0.8 to 1.1) | 0.9 (0.8 to 1.1) |
| Taiwan (Province of China) | Both sexes | 2.2 (1.8 to 2.6) | 1.7 (1.5 to 1.9) | 1.7 (1.5 to 1.9) |
|  | Females | 2.0 (1.6 to 2.4) | 1.7 (1.6 to 1.8) | 1.7 (1.6 to 1.8) |
|  | Males | 2.4 (1.9 to 2.9) | 1.7 (1.5 to 2.0) | 1.7 (1.5 to 2.0) |
| Tajikistan | Both sexes | 1.9 (1.3 to 2.6) | 1.3 (1.0 to 1.6) | 1.3 (1.0 to 1.6) |
|  | Females | 2.1 (1.4 to 2.8) | 1.5 (1.2 to 1.7) | 1.5 (1.2 to 1.7) |
|  | Males | 1.7 (1.1 to 2.4) | 1.1 (0.8 to 1.3) | 1.1 (0.8 to 1.4) |
| Thailand | Both sexes | 1.6 (0.9 to 2.3) | 0.6 (0.3 to 1.0) | 0.6 (0.3 to 1.0) |
|  | Females | 1.7 (1.0 to 2.4) | 0.8 (0.4 to 1.1) | 0.8 (0.4 to 1.1) |
|  | Males | 1.3 (0.6 to 2.0) | 0.4 (0.0 to 0.7) | 0.4 (0.1 to 0.8) |
| Timor-Leste | Both sexes | 1.5 (0.9 to 2.1) | 0.6 (0.2 to 0.9) | 0.6 (0.2 to 0.9) |
|  | Females | 1.6 (1.0 to 2.2) | 0.7 (0.3 to 1.0) | 0.7 (0.3 to 1.0) |
|  | Males | 1.3 (0.7 to 1.9) | 0.4 (0.0 to 0.7) | 0.4 (0.0 to 0.8) |
| Tonga | Both sexes | 2.1 (1.4 to 2.8) | 1.4 (1.1 to 1.7) | 1.4 (1.1 to 1.7) |
|  | Females | 2.2 (1.6 to 2.9) | 1.5 (1.2 to 1.8) | 1.5 (1.2 to 1.8) |
|  | Males | 1.9 (1.2 to 2.6) | 1.2 (0.9 to 1.5) | 1.2 (0.9 to 1.5) |
| Trinidad and Tobago | Both sexes | 1.7 (1.0 to 2.5) | 1.2 (1.0 to 1.5) | 1.2 (1.0 to 1.5) |
|  | Females | 1.4 (0.7 to 2.2) | 1.0 (0.8 to 1.3) | 1.0 (0.8 to 1.3) |
|  | Males | 2.2 (1.4 to 2.9) | 1.6 (1.3 to 1.9) | 1.6 (1.3 to 1.9) |
| Tunisia | Both sexes | 0.9 (0.1 to 1.7) | 0.2 (-0.0 to 0.5) | 0.2 (-0.0 to 0.5) |
|  | Females | 0.3 (-0.5 to 1.1) | -0.2 (-0.4 to 0.0) | -0.2 (-0.5 to -0.0) |
|  | Males | 1.7 (0.9 to 2.5) | 0.9 (0.6 to 1.2) | 0.9 (0.6 to 1.2) |
| Turkmenistan | Both sexes | 3.0 (2.2 to 3.9) | 1.9 (1.4 to 2.4) | 1.9 (1.4 to 2.4) |
|  | Females | 2.9 (2.0 to 3.8) | 1.8 (1.4 to 2.3) | 1.9 (1.4 to 2.3) |
|  | Males | 3.3 (2.4 to 4.2) | 2.0 (1.5 to 2.6) | 2.0 (1.5 to 2.6) |
| Tuvalu | Both sexes | 1.7 (1.0 to 2.4) | 0.9 (0.6 to 1.2) | 0.9 (0.6 to 1.2) |
|  | Females | 1.8 (1.2 to 2.5) | 1.0 (0.7 to 1.4) | 1.0 (0.7 to 1.4) |
|  | Males | 1.6 (0.9 to 2.3) | 0.8 (0.5 to 1.2) | 0.8 (0.5 to 1.2) |
| T眉rkiye | Both sexes | 0.5 (-0.2 to 1.2) | -0.1 (-0.3 to 0.1) | -0.1 (-0.3 to 0.1) |
|  | Females | 0.1 (-0.6 to 0.8) | -0.5 (-0.7 to -0.2) | -0.5 (-0.7 to -0.3) |
|  | Males | 1.0 (0.3 to 1.7) | 0.5 (0.2 to 0.7) | 0.5 (0.2 to 0.7) |
| Ukraine | Both sexes | 1.4 (0.7 to 2.1) | 0.8 (0.6 to 1.0) | 0.8 (0.6 to 1.1) |
|  | Females | 1.7 (1.0 to 2.4) | 1.0 (0.7 to 1.3) | 1.0 (0.8 to 1.3) |
|  | Males | 0.9 (0.2 to 1.6) | 0.5 (0.3 to 0.7) | 0.5 (0.3 to 0.7) |
| United Arab Emirates | Both sexes | 0.6 (0.0 to 1.2) | 0.1 (-0.1 to 0.3) | 0.1 (-0.1 to 0.3) |
|  | Females | 0.3 (-0.3 to 1.0) | -0.2 (-0.4 to -0.0) | -0.3 (-0.5 to -0.1) |
|  | Males | 1.0 (0.4 to 1.6) | 0.6 (0.4 to 0.7) | 0.6 (0.4 to 0.7) |
| United Kingdom | Both sexes | 3.1 (2.4 to 3.8) | 1.9 (1.5 to 2.2) | 1.9 (1.5 to 2.2) |
|  | Females | 3.5 (2.8 to 4.3) | 2.1 (1.7 to 2.4) | 2.1 (1.7 to 2.4) |
|  | Males | 2.4 (1.7 to 3.2) | 1.5 (1.1 to 1.9) | 1.5 (1.1 to 1.9) |
| United Republic of Tanzania | Both sexes | 1.9 (1.1 to 2.8) | 0.9 (0.4 to 1.3) | 0.9 (0.5 to 1.3) |
|  | Females | 2.4 (1.4 to 3.2) | 1.1 (0.6 to 1.6) | 1.1 (0.6 to 1.6) |
|  | Males | 1.4 (0.6 to 2.3) | 0.6 (0.2 to 1.0) | 0.6 (0.2 to 1.0) |
| United States Virgin Islands | Both sexes | 1.3 (0.8 to 1.8) | 1.1 (0.9 to 1.2) | 1.1 (0.9 to 1.2) |
|  | Females | 1.2 (0.7 to 1.8) | 1.0 (0.9 to 1.2) | 1.0 (0.9 to 1.2) |
|  | Males | 1.5 (0.9 to 2.0) | 1.2 (1.0 to 1.4) | 1.2 (1.0 to 1.4) |
| United States of America | Both sexes | 0.2 (-0.6 to 1.0) | -0.4 (-0.7 to -0.0) | -0.4 (-0.7 to -0.0) |
|  | Females | 0.1 (-0.8 to 0.9) | -0.5 (-0.9 to -0.2) | -0.5 (-0.9 to -0.2) |
|  | Males | 0.4 (-0.3 to 1.1) | -0.1 (-0.4 to 0.2) | -0.1 (-0.4 to 0.2) |
| Uruguay | Both sexes | 1.3 (0.7 to 1.9) | 0.9 (0.7 to 1.1) | 0.9 (0.7 to 1.1) |
|  | Females | 1.3 (0.7 to 1.9) | 0.9 (0.7 to 1.1) | 0.9 (0.7 to 1.1) |
|  | Males | 1.3 (0.6 to 1.9) | 0.8 (0.6 to 1.1) | 0.9 (0.6 to 1.1) |
| Uzbekistan | Both sexes | 0.4 (-0.2 to 1.1) | 0.2 (-0.0 to 0.4) | 0.2 (-0.0 to 0.4) |
|  | Females | 1.0 (0.3 to 1.6) | 0.6 (0.4 to 0.8) | 0.6 (0.4 to 0.8) |
|  | Males | -0.4 (-1.1 to 0.4) | -0.5 (-0.7 to -0.2) | -0.5 (-0.7 to -0.2) |
| Vanuatu | Both sexes | 1.7 (1.1 to 2.4) | 0.9 (0.6 to 1.3) | 0.9 (0.6 to 1.2) |
|  | Females | 1.8 (1.1 to 2.4) | 1.0 (0.7 to 1.3) | 1.0 (0.7 to 1.3) |
|  | Males | 1.6 (0.9 to 2.2) | 0.8 (0.5 to 1.1) | 0.8 (0.5 to 1.1) |
| Venezuela (Bolivarian Republic of) | Both sexes | 2.6 (1.9 to 3.4) | 1.7 (1.3 to 2.1) | 1.7 (1.3 to 2.1) |
|  | Females | 2.4 (1.6 to 3.2) | 1.6 (1.3 to 1.9) | 1.6 (1.3 to 1.9) |
|  | Males | 3.1 (2.3 to 3.9) | 1.9 (1.5 to 2.4) | 1.9 (1.5 to 2.4) |
| Viet Nam | Both sexes | 0.9 (0.3 to 1.4) | 0.3 (0.1 to 0.5) | 0.3 (0.1 to 0.5) |
|  | Females | 0.9 (0.4 to 1.4) | 0.4 (0.2 to 0.6) | 0.4 (0.2 to 0.6) |
|  | Males | 1.0 (0.4 to 1.5) | 0.3 (0.1 to 0.6) | 0.3 (0.1 to 0.6) |
| Zambia | Both sexes | 2.7 (1.8 to 3.6) | 1.4 (0.9 to 1.9) | 1.4 (0.9 to 1.9) |
|  | Females | 2.6 (1.6 to 3.5) | 1.2 (0.8 to 1.7) | 1.2 (0.8 to 1.7) |
|  | Males | 2.8 (1.9 to 3.7) | 1.6 (1.0 to 2.0) | 1.6 (1.1 to 2.1) |
| Zimbabwe | Both sexes | 0.8 (-0.1 to 1.6) | 0.3 (-0.0 to 0.6) | 0.3 (-0.0 to 0.6) |
|  | Females | 0.8 (-0.0 to 1.7) | 0.3 (0.0 to 0.7) | 0.3 (0.0 to 0.7) |
|  | Males | 0.6 (-0.2 to 1.4) | 0.2 (-0.1 to 0.5) | 0.2 (-0.1 to 0.5) |

**Table S7 Age-standardized DALYs rates for anxiety disorders attributable to risk factors in 2023.**

| location_name | age_name | sex_name | Intimate partner violence | Sexual violence against children | Bullying victimization |
| --- | --- | --- | --- | --- | --- |
| Afghanistan | <5 years | Both sexes | NA | NA | 0 (0 to 0) |
| Afghanistan | <5 years | Males | NA | NA | 0 (0 to 0) |
| Afghanistan | <5 years | Females | NA | NA | 0 (0 to 0) |
| Afghanistan | 5-9 years | Both sexes | NA | NA | 5.75 (1.52 to 15.91) |
| Afghanistan | 5-9 years | Males | NA | NA | 8.81 (2.38 to 23.89) |
| Afghanistan | 5-9 years | Females | NA | NA | 2.47 (0.54 to 7.55) |
| Afghanistan | 10-14 years | Both sexes | NA | NA | 96.2 (39.54 to 206.84) |
| Afghanistan | 10-14 years | Males | NA | NA | 141 (57.19 to 289.58) |
| Afghanistan | 10-14 years | Females | NA | NA | 48.21 (15.96 to 116.02) |
| Afghanistan | 15-19 years | Both sexes | 75.69 (-8.28 to 282.37) | 73.82 (-26.12 to 309.78) | 89.64 (35.25 to 189.38) |
| Afghanistan | 15-19 years | Males | NA | 67.84 (-24.26 to 266.54) | 128.39 (51.87 to 249.96) |
| Afghanistan | 15-19 years | Females | 154.42 (-16.89 to 576.09) | 80.05 (-27.83 to 365.9) | 49.33 (15.33 to 116.89) |
| Afghanistan | 20-24 years | Both sexes | 114.25 (-21.92 to 314.92) | 62.98 (-19.02 to 238.62) | 61.12 (21.13 to 129.14) |
| Afghanistan | 20-24 years | Males | NA | 54.58 (-17.01 to 203.87) | 87.92 (34.14 to 179.26) |
| Afghanistan | 20-24 years | Females | 229.69 (-44.06 to 633.15) | 71.47 (-21.12 to 290.23) | 34.03 (8.83 to 87.62) |
| Afghanistan | 25-29 years | Both sexes | 141.19 (-27.87 to 333.62) | 55.91 (-19.55 to 221.47) | 43.01 (11.94 to 93.34) |
| Afghanistan | 25-29 years | Males | NA | 47.19 (-15 to 176.78) | 64.04 (20.43 to 134.87) |
| Afghanistan | 25-29 years | Females | 282.35 (-55.74 to 667.17) | 64.62 (-23.15 to 265.08) | 21.99 (2.86 to 53.77) |
| Afghanistan | 30-34 years | Both sexes | 140.54 (-34.14 to 322.13) | 55.66 (-19.81 to 211.55) | 29.4 (5.91 to 64.43) |
| Afghanistan | 30-34 years | Males | NA | 47.66 (-13.76 to 187.18) | 46.17 (10.01 to 98.25) |
| Afghanistan | 30-34 years | Females | 283.01 (-68.76 to 648.71) | 63.77 (-25.4 to 256.3) | 12.4 (0 to 36.42) |
| Afghanistan | 35-39 years | Both sexes | 136.54 (-34.35 to 296.16) | 53.12 (-19.36 to 209.35) | 14.82 (0.13 to 40.37) |
| Afghanistan | 35-39 years | Males | NA | 46.98 (-16.91 to 173.86) | 26.86 (0.25 to 70.78) |
| Afghanistan | 35-39 years | Females | 281.52 (-70.82 to 610.61) | 59.63 (-21.96 to 236.51) | 2.03 (0 to 8.07) |
| Afghanistan | 40-44 years | Both sexes | 133 (-30.22 to 290.1) | 53.78 (-19.55 to 195.94) | 6.29 (0 to 22.03) |
| Afghanistan | 40-44 years | Males | NA | 49.07 (-17.73 to 175.83) | 12.35 (0 to 43.3) |
| Afghanistan | 40-44 years | Females | 270.82 (-61.54 to 590.7) | 58.65 (-21.43 to 236.81) | 0 (0 to 0) |
| Afghanistan | 45-49 years | Both sexes | 140.21 (-31.92 to 288.6) | 53.7 (-20.17 to 203.49) | 0.6 (0 to 4.03) |
| Afghanistan | 45-49 years | Males | NA | 50.91 (-19.55 to 182.14) | 1.11 (0 to 7.47) |
| Afghanistan | 45-49 years | Females | 304.26 (-69.28 to 626.29) | 56.96 (-21.2 to 221.88) | 0 (0 to 0) |
| Afghanistan | 50-54 years | Both sexes | 129.34 (-26.46 to 334.86) | 55.03 (-20.98 to 238.98) | 0 (0 to 0) |
| Afghanistan | 50-54 years | Males | NA | 53.2 (-19.68 to 214.29) | 0 (0 to 0) |
| Afghanistan | 50-54 years | Females | 254.77 (-52.11 to 659.6) | 56.81 (-22.13 to 257.7) | 0 (0 to 0) |
| Afghanistan | 55-59 years | Both sexes | 137.06 (-27.63 to 362.7) | 53.48 (-20.72 to 225.93) | 0 (0 to 0) |
| Afghanistan | 55-59 years | Males | NA | 52.91 (-20.49 to 202.43) | 0 (0 to 0) |
| Afghanistan | 55-59 years | Females | 246.26 (-49.65 to 651.71) | 53.93 (-20.97 to 226.84) | 0 (0 to 0) |
| Afghanistan | 60-64 years | Both sexes | 128.89 (-24.12 to 356.54) | 52.16 (-18.46 to 229.78) | 0 (0 to 0) |
| Afghanistan | 60-64 years | Males | NA | 52.54 (-19.39 to 202.78) | 0 (0 to 0) |
| Afghanistan | 60-64 years | Females | 208.56 (-39.03 to 576.94) | 51.93 (-18.13 to 244.3) | 0 (0 to 0) |
| Afghanistan | 65-69 years | Both sexes | 103.35 (-15.8 to 323.55) | 49.03 (-16.68 to 225.18) | 0 (0 to 0) |
| Afghanistan | 65-69 years | Males | NA | 50.79 (-18.2 to 213.75) | 0 (0 to 0) |
| Afghanistan | 65-69 years | Females | 172.77 (-26.41 to 540.86) | 47.85 (-16.03 to 235.71) | 0 (0 to 0) |
| Afghanistan | 70-74 years | Both sexes | 90 (-11.09 to 304.96) | 40.78 (-12.1 to 189.42) | 0 (0 to 0) |
| Afghanistan | 70-74 years | Males | NA | 42.96 (-13.5 to 194.36) | 0 (0 to 0) |
| Afghanistan | 70-74 years | Females | 159.85 (-19.69 to 541.67) | 39.09 (-11.07 to 175.19) | 0 (0 to 0) |
| Afghanistan | 75-79 years | Both sexes | 65.92 (-7.82 to 216.14) | 35.97 (-10.83 to 154.31) | 0 (0 to 0) |
| Afghanistan | 75-79 years | Males | NA | 38.62 (-11.58 to 162.16) | 0 (0 to 0) |
| Afghanistan | 75-79 years | Females | 129.78 (-15.4 to 425.53) | 33.4 (-10.22 to 153.31) | 0 (0 to 0) |
| Afghanistan | 80-84 years | Both sexes | 46.62 (-4.83 to 166.13) | 25.75 (-7.45 to 116.31) | 0 (0 to 0) |
| Afghanistan | 80-84 years | Males | NA | 27.68 (-8.22 to 116.02) | 0 (0 to 0) |
| Afghanistan | 80-84 years | Females | 94.99 (-9.84 to 338.54) | 23.76 (-6.74 to 116.98) | 0 (0 to 0) |
| Afghanistan | 85-89 years | Both sexes | 42.54 (-4.62 to 154.58) | 23.08 (-7.29 to 105.3) | 0 (0 to 0) |
| Afghanistan | 85-89 years | Males | NA | 24.97 (-7.31 to 109) | 0 (0 to 0) |
| Afghanistan | 85-89 years | Females | 93.34 (-10.15 to 339.15) | 20.83 (-6.58 to 101.68) | 0 (0 to 0) |
| Afghanistan | 90-94 years | Both sexes | 38.52 (-4.4 to 144.75) | 21.41 (-6.79 to 103.22) | 0 (0 to 0) |
| Afghanistan | 90-94 years | Males | NA | 23.14 (-7.45 to 108.43) | 0 (0 to 0) |
| Afghanistan | 90-94 years | Females | 92.8 (-10.6 to 348.72) | 18.96 (-6.03 to 93) | 0 (0 to 0) |
| Afghanistan | 95+ years | Both sexes | 38.07 (-4.4 to 142.42) | 20.9 (-7.38 to 95.18) | 0 (0 to 0) |
| Afghanistan | 95+ years | Males | NA | 22.63 (-7.93 to 96.95) | 0 (0 to 0) |
| Afghanistan | 95+ years | Females | 94.23 (-10.9 to 352.52) | 18.36 (-6.6 to 91.7) | 0 (0 to 0) |
| Albania | <5 years | Both sexes | NA | NA | 0 (0 to 0) |
| Albania | <5 years | Males | NA | NA | 0 (0 to 0) |
| Albania | <5 years | Females | NA | NA | 0 (0 to 0) |
| Albania | 5-9 years | Both sexes | NA | NA | 5.28 (1.21 to 15.53) |
| Albania | 5-9 years | Males | NA | NA | 5.59 (1.37 to 16.36) |
| Albania | 5-9 years | Females | NA | NA | 4.93 (1.02 to 16.52) |
| Albania | 10-14 years | Both sexes | NA | NA | 72.3 (25.05 to 158.38) |
| Albania | 10-14 years | Males | NA | NA | 79.05 (26.42 to 174.08) |
| Albania | 10-14 years | Females | NA | NA | 65.01 (20.24 to 146.04) |
| Albania | 15-19 years | Both sexes | 43.52 (-4.26 to 170.56) | 68.44 (-24.89 to 238.12) | 60.21 (21.33 to 131.13) |
| Albania | 15-19 years | Males | NA | 59.55 (-21.78 to 198.04) | 67.99 (25.15 to 141.88) |
| Albania | 15-19 years | Females | 87.67 (-8.59 to 343.56) | 77.46 (-28.11 to 277.77) | 52.32 (15.87 to 124.67) |
| Albania | 20-24 years | Both sexes | 53.98 (-7.15 to 181.8) | 54.14 (-17.09 to 182.55) | 42.68 (15.6 to 100.01) |
| Albania | 20-24 years | Males | NA | 53.9 (-18.13 to 191.78) | 48.46 (17.36 to 103.45) |
| Albania | 20-24 years | Females | 105.01 (-13.9 to 353.65) | 54.37 (-13.92 to 186.16) | 37.22 (10.96 to 96.9) |
| Albania | 25-29 years | Both sexes | 93.01 (-16.13 to 269.99) | 52.33 (-16.24 to 171.16) | 30.34 (8.5 to 67.13) |
| Albania | 25-29 years | Males | NA | 47.98 (-15.3 to 177.49) | 34.96 (11.61 to 72.98) |
| Albania | 25-29 years | Females | 188.98 (-32.77 to 548.54) | 56.82 (-16.63 to 205.7) | 25.57 (3.64 to 63.54) |
| Albania | 30-34 years | Both sexes | 108.84 (-18.66 to 297.36) | 51.33 (-18.18 to 192.39) | 20.03 (3.63 to 45.81) |
| Albania | 30-34 years | Males | NA | 47.83 (-17.11 to 179.23) | 24.34 (4.8 to 51.94) |
| Albania | 30-34 years | Females | 226.04 (-38.75 to 617.58) | 55.1 (-15.39 to 192.33) | 15.4 (0 to 44.48) |
| Albania | 35-39 years | Both sexes | 110.14 (-22.19 to 295.88) | 54.16 (-18.44 to 180.77) | 9.11 (0.06 to 25.25) |
| Albania | 35-39 years | Males | NA | 45.27 (-17.21 to 169.95) | 14.88 (0.08 to 40.25) |
| Albania | 35-39 years | Females | 228.6 (-46.06 to 614.12) | 63.72 (-21.59 to 220.36) | 2.91 (0 to 11.81) |
| Albania | 40-44 years | Both sexes | 114.31 (-24.58 to 297.02) | 53.72 (-18.11 to 172.84) | 3.48 (0 to 12.32) |
| Albania | 40-44 years | Males | NA | 44.14 (-16.66 to 154.05) | 7.11 (0 to 25.14) |
| Albania | 40-44 years | Females | 224.09 (-48.19 to 582.27) | 62.92 (-21.77 to 207.24) | 0 (0 to 0) |
| Albania | 45-49 years | Both sexes | 111.84 (-21.63 to 304.16) | 53.12 (-18.95 to 179.78) | 0.19 (0 to 1.49) |
| Albania | 45-49 years | Males | NA | 43.67 (-17.28 to 149.09) | 0.41 (0 to 3.15) |
| Albania | 45-49 years | Females | 212.01 (-41.01 to 576.55) | 61.59 (-21.93 to 213.83) | 0 (0 to 0) |
| Albania | 50-54 years | Both sexes | 97.95 (-13.8 to 281.14) | 56.49 (-20.11 to 209.26) | 0 (0 to 0) |
| Albania | 50-54 years | Males | NA | 44.14 (-17.69 to 177.29) | 0 (0 to 0) |
| Albania | 50-54 years | Females | 185.68 (-26.16 to 532.95) | 67.56 (-24.34 to 236.52) | 0 (0 to 0) |
| Albania | 55-59 years | Both sexes | 87.07 (-11.44 to 257.37) | 50.99 (-19.59 to 187.97) | 0 (0 to 0) |
| Albania | 55-59 years | Males | NA | 44.23 (-18.62 to 177.9) | 0 (0 to 0) |
| Albania | 55-59 years | Females | 168.83 (-22.18 to 499.06) | 57.34 (-19.22 to 204.27) | 0 (0 to 0) |
| Albania | 60-64 years | Both sexes | 89.37 (-13.65 to 255.83) | 50.96 (-19.55 to 190.42) | 0 (0 to 0) |
| Albania | 60-64 years | Males | NA | 44.25 (-18.09 to 179.58) | 0 (0 to 0) |
| Albania | 60-64 years | Females | 175.99 (-26.89 to 503.8) | 57.46 (-19.66 to 212.91) | 0 (0 to 0) |
| Albania | 65-69 years | Both sexes | 64.51 (-9.16 to 202.31) | 46.73 (-17.54 to 172.4) | 0 (0 to 0) |
| Albania | 65-69 years | Males | NA | 43.53 (-17 to 172.34) | 0 (0 to 0) |
| Albania | 65-69 years | Females | 127.67 (-18.12 to 400.36) | 49.87 (-16.77 to 180.02) | 0 (0 to 0) |
| Albania | 70-74 years | Both sexes | 68.5 (-9.1 to 227.61) | 36.68 (-11.43 to 150.9) | 0 (0 to 0) |
| Albania | 70-74 years | Males | NA | 37.46 (-10.91 to 164.7) | 0 (0 to 0) |
| Albania | 70-74 years | Females | 126.56 (-16.81 to 420.52) | 36.01 (-11.09 to 139.32) | 0 (0 to 0) |
| Albania | 75-79 years | Both sexes | 41.86 (-3.82 to 156.25) | 38.64 (-11.22 to 161.93) | 0 (0 to 0) |
| Albania | 75-79 years | Males | NA | 33.87 (-10.03 to 128.11) | 0 (0 to 0) |
| Albania | 75-79 years | Females | 75.18 (-6.86 to 280.66) | 42.43 (-11.51 to 181.51) | 0 (0 to 0) |
| Albania | 80-84 years | Both sexes | 29.48 (-2.36 to 118.16) | 28.04 (-7.6 to 127.64) | 0 (0 to 0) |
| Albania | 80-84 years | Males | NA | 25.15 (-6.94 to 102.53) | 0 (0 to 0) |
| Albania | 80-84 years | Females | 51.14 (-4.1 to 205) | 30.16 (-8.1 to 135.02) | 0 (0 to 0) |
| Albania | 85-89 years | Both sexes | 28.19 (-2.51 to 113.58) | 25.06 (-7.61 to 111.73) | 0 (0 to 0) |
| Albania | 85-89 years | Males | NA | 22.85 (-7.01 to 93.98) | 0 (0 to 0) |
| Albania | 85-89 years | Females | 49.42 (-4.4 to 199.12) | 26.74 (-8.14 to 125.11) | 0 (0 to 0) |
| Albania | 90-94 years | Both sexes | 31.23 (-2.99 to 127.99) | 23.04 (-7.52 to 107.59) | 0 (0 to 0) |
| Albania | 90-94 years | Males | NA | 21.42 (-7.36 to 89.37) | 0 (0 to 0) |
| Albania | 90-94 years | Females | 48.83 (-4.68 to 200.12) | 23.95 (-7.94 to 115.72) | 0 (0 to 0) |
| Albania | 95+ years | Both sexes | 36.24 (-3.44 to 147.45) | 22.79 (-7.8 to 101.71) | 0 (0 to 0) |
| Albania | 95+ years | Males | NA | 21.17 (-7.36 to 81.83) | 0 (0 to 0) |
| Albania | 95+ years | Females | 49.7 (-4.72 to 202.23) | 23.39 (-8 to 108.57) | 0 (0 to 0) |
| Algeria | <5 years | Both sexes | NA | NA | 0 (0 to 0) |
| Algeria | <5 years | Males | NA | NA | 0 (0 to 0) |
| Algeria | <5 years | Females | NA | NA | 0 (0 to 0) |
| Algeria | 5-9 years | Both sexes | NA | NA | 6.68 (1.67 to 19.09) |
| Algeria | 5-9 years | Males | NA | NA | 9.16 (2.36 to 25.24) |
| Algeria | 5-9 years | Females | NA | NA | 4.12 (0.88 to 13.02) |
| Algeria | 10-14 years | Both sexes | NA | NA | 113.76 (46.19 to 245.26) |
| Algeria | 10-14 years | Males | NA | NA | 143.65 (57.72 to 287.13) |
| Algeria | 10-14 years | Females | NA | NA | 82.61 (28.19 to 184.96) |
| Algeria | 15-19 years | Both sexes | 32.84 (-2.75 to 131.23) | 69.51 (-25.23 to 279.46) | 143.3 (56.62 to 278.36) |
| Algeria | 15-19 years | Males | NA | 68.06 (-24.58 to 252.83) | 157.45 (68.84 to 282.6) |
| Algeria | 15-19 years | Females | 67.05 (-5.62 to 267.96) | 71.01 (-25.89 to 316.58) | 128.56 (44.11 to 271.55) |
| Algeria | 20-24 years | Both sexes | 59.35 (-8.38 to 191.7) | 62.81 (-20.35 to 243.35) | 102.57 (40.68 to 204.36) |
| Algeria | 20-24 years | Males | NA | 57.28 (-18.89 to 211.71) | 108.27 (45.75 to 208.84) |
| Algeria | 20-24 years | Females | 120.73 (-17.04 to 389.94) | 68.53 (-21.86 to 292.99) | 96.66 (29.13 to 220.88) |
| Algeria | 25-29 years | Both sexes | 76.09 (-13.47 to 222.35) | 56.22 (-20.15 to 208.24) | 72.26 (21.07 to 149.96) |
| Algeria | 25-29 years | Males | NA | 49.57 (-16.84 to 179.61) | 77.14 (26.97 to 158.94) |
| Algeria | 25-29 years | Females | 154.05 (-27.27 to 450.18) | 63.04 (-23.55 to 247.59) | 67.26 (11.05 to 160.83) |
| Algeria | 30-34 years | Both sexes | 78.98 (-13.77 to 226.98) | 55.25 (-21.58 to 211.36) | 46.84 (7.57 to 104.33) |
| Algeria | 30-34 years | Males | NA | 48.99 (-18.3 to 179.03) | 54.74 (12.64 to 117.71) |
| Algeria | 30-34 years | Females | 158.71 (-27.68 to 456.12) | 61.57 (-24.97 to 247.67) | 38.87 (0 to 106.59) |
| Algeria | 35-39 years | Both sexes | 78.15 (-15.18 to 210.5) | 53.23 (-18.31 to 212.82) | 21.94 (0.33 to 56.72) |
| Algeria | 35-39 years | Males | NA | 48.71 (-17.42 to 195.42) | 34.92 (0.17 to 82.72) |
| Algeria | 35-39 years | Females | 155.44 (-30.19 to 418.66) | 57.7 (-19.37 to 237.24) | 9.09 (0 to 33.87) |
| Algeria | 40-44 years | Both sexes | 77.46 (-14.95 to 201.98) | 53.96 (-19.56 to 198.98) | 7.9 (0 to 25.67) |
| Algeria | 40-44 years | Males | NA | 50.85 (-17.79 to 187.81) | 15.85 (0 to 51.56) |
| Algeria | 40-44 years | Females | 154.23 (-29.77 to 402.15) | 57.04 (-22.19 to 218.23) | 0.01 (0 to 0.04) |
| Algeria | 45-49 years | Both sexes | 78.69 (-14.81 to 211.7) | 53.73 (-19.35 to 197.81) | 0.55 (0 to 3.57) |
| Algeria | 45-49 years | Males | NA | 52.81 (-18.95 to 184.52) | 1.11 (0 to 7.15) |
| Algeria | 45-49 years | Females | 156.89 (-29.53 to 422.06) | 54.64 (-19.74 to 214.18) | 0 (0 to 0) |
| Algeria | 50-54 years | Both sexes | 72.68 (-11.32 to 210.79) | 54.79 (-21.2 to 229.18) | 0 (0 to 0) |
| Algeria | 50-54 years | Males | NA | 55.35 (-21.98 to 218.99) | 0 (0 to 0) |
| Algeria | 50-54 years | Females | 144.97 (-22.58 to 420.47) | 54.24 (-20.56 to 227.31) | 0 (0 to 0) |
| Algeria | 55-59 years | Both sexes | 67.86 (-10.05 to 203.37) | 53.87 (-23.16 to 218.6) | 0 (0 to 0) |
| Algeria | 55-59 years | Males | NA | 55.75 (-23.55 to 213.26) | 0 (0 to 0) |
| Algeria | 55-59 years | Females | 136.65 (-20.23 to 409.51) | 51.96 (-21.56 to 211.26) | 0 (0 to 0) |
| Algeria | 60-64 years | Both sexes | 53.76 (-7.86 to 169.16) | 52.69 (-20.56 to 213.41) | 0 (0 to 0) |
| Algeria | 60-64 years | Males | NA | 55.57 (-23.44 to 205.4) | 0 (0 to 0) |
| Algeria | 60-64 years | Females | 108.96 (-15.94 to 342.83) | 49.72 (-17.61 to 223.29) | 0 (0 to 0) |
| Algeria | 65-69 years | Both sexes | 41.54 (-4.81 to 139.8) | 49.45 (-18.23 to 214.9) | 0 (0 to 0) |
| Algeria | 65-69 years | Males | NA | 53.01 (-19.32 to 223.41) | 0 (0 to 0) |
| Algeria | 65-69 years | Females | 84.75 (-9.81 to 285.21) | 45.76 (-17.19 to 212.12) | 0 (0 to 0) |
| Algeria | 70-74 years | Both sexes | 36.85 (-3.77 to 129.46) | 41.2 (-11.98 to 188.37) | 0 (0 to 0) |
| Algeria | 70-74 years | Males | NA | 44.86 (-12.93 to 200.42) | 0 (0 to 0) |
| Algeria | 70-74 years | Females | 76.09 (-7.77 to 267.27) | 37.32 (-11.4 to 167.31) | 0 (0 to 0) |
| Algeria | 75-79 years | Both sexes | 30.12 (-3.18 to 109) | 35.82 (-10.63 to 147.66) | 0 (0 to 0) |
| Algeria | 75-79 years | Males | NA | 39.52 (-12.11 to 160.12) | 0 (0 to 0) |
| Algeria | 75-79 years | Females | 59.7 (-6.3 to 216.03) | 32.18 (-9.15 to 140.62) | 0 (0 to 0) |
| Algeria | 80-84 years | Both sexes | 20.7 (-2 to 84.07) | 25.61 (-7.44 to 118.31) | 0 (0 to 0) |
| Algeria | 80-84 years | Males | NA | 28.81 (-8.73 to 125.49) | 0 (0 to 0) |
| Algeria | 80-84 years | Females | 41.11 (-3.98 to 166.91) | 22.46 (-6.05 to 113.44) | 0 (0 to 0) |
| Algeria | 85-89 years | Both sexes | 20.92 (-1.99 to 80.31) | 22.65 (-6.9 to 98.64) | 0 (0 to 0) |
| Algeria | 85-89 years | Males | NA | 25.5 (-7.65 to 101.71) | 0 (0 to 0) |
| Algeria | 85-89 years | Females | 40.4 (-3.83 to 155.09) | 19.99 (-6.34 to 97.98) | 0 (0 to 0) |
| Algeria | 90-94 years | Both sexes | 22.03 (-2.23 to 86.63) | 20.7 (-6.95 to 96.49) | 0 (0 to 0) |
| Algeria | 90-94 years | Males | NA | 23.87 (-8.17 to 102.49) | 0 (0 to 0) |
| Algeria | 90-94 years | Females | 40.35 (-4.08 to 158.69) | 18.06 (-5.79 to 88.09) | 0 (0 to 0) |
| Algeria | 95+ years | Both sexes | 27.75 (-2.74 to 113.38) | 19.33 (-6.63 to 90.31) | 0 (0 to 0) |
| Algeria | 95+ years | Males | NA | 23.23 (-7.61 to 98.08) | 0 (0 to 0) |
| Algeria | 95+ years | Females | 40.79 (-4.02 to 166.65) | 17.5 (-6.09 to 86.66) | 0 (0 to 0) |
| American Samoa | <5 years | Both sexes | NA | NA | 0 (0 to 0) |
| American Samoa | <5 years | Males | NA | NA | 0 (0 to 0) |
| American Samoa | <5 years | Females | NA | NA | 0 (0 to 0) |
| American Samoa | 5-9 years | Both sexes | NA | NA | 7.38 (1.96 to 20.37) |
| American Samoa | 5-9 years | Males | NA | NA | 9.05 (2.53 to 24.62) |
| American Samoa | 5-9 years | Females | NA | NA | 5.63 (1.31 to 15.73) |
| American Samoa | 10-14 years | Both sexes | NA | NA | 126.23 (38.53 to 307.91) |
| American Samoa | 10-14 years | Males | NA | NA | 152.31 (45.13 to 379.65) |
| American Samoa | 10-14 years | Females | NA | NA | 98.4 (28.39 to 243.42) |
| American Samoa | 15-19 years | Both sexes | 44.86 (-4.24 to 180.21) | 43.54 (-16.59 to 167.24) | 93.88 (31.88 to 204.08) |
| American Samoa | 15-19 years | Males | NA | 45.6 (-18.38 to 170.64) | 110.86 (36 to 239.44) |
| American Samoa | 15-19 years | Females | 93.63 (-8.84 to 376.14) | 41.29 (-14.95 to 162.08) | 75.42 (22.91 to 180.33) |
| American Samoa | 20-24 years | Both sexes | 88.64 (-17.22 to 270.32) | 35.94 (-10.1 to 134.81) | 64.93 (23.16 to 145.32) |
| American Samoa | 20-24 years | Males | NA | 37.14 (-13.04 to 144.84) | 75.95 (27.88 to 166.61) |
| American Samoa | 20-24 years | Females | 179.7 (-34.91 to 548.02) | 34.71 (-10.25 to 121.59) | 53.6 (15.25 to 129.76) |
| American Samoa | 25-29 years | Both sexes | 110.42 (-25.62 to 298.38) | 34.43 (-10.19 to 125.95) | 46.1 (12.89 to 101.54) |
| American Samoa | 25-29 years | Males | NA | 33.34 (-10.22 to 135.18) | 54.82 (17.32 to 113) |
| American Samoa | 25-29 years | Females | 219.08 (-50.84 to 591.98) | 35.49 (-10.14 to 127.63) | 37.52 (5.3 to 98.94) |
| American Samoa | 30-34 years | Both sexes | 111.22 (-27.41 to 305.02) | 34.44 (-10.24 to 126.06) | 31.82 (5.64 to 74.08) |
| American Samoa | 30-34 years | Males | NA | 34 (-11.13 to 137.15) | 40.06 (9.16 to 86.85) |
| American Samoa | 30-34 years | Females | 225.06 (-55.47 to 617.24) | 34.9 (-10.14 to 130.05) | 23.39 (0 to 66.67) |
| American Samoa | 35-39 years | Both sexes | 108.88 (-30.55 to 269) | 35.9 (-11.96 to 128.39) | 15.68 (0.1 to 44.45) |
| American Samoa | 35-39 years | Males | NA | 33.09 (-10.53 to 129.23) | 25.72 (0.15 to 65.94) |
| American Samoa | 35-39 years | Females | 224.64 (-63.03 to 554.99) | 38.89 (-11.54 to 132.59) | 5 (0 to 21.06) |
| American Samoa | 40-44 years | Both sexes | 108.69 (-28.82 to 272.09) | 35.94 (-12.47 to 114.69) | 6.67 (0 to 23.36) |
| American Samoa | 40-44 years | Males | NA | 33.81 (-11.98 to 119) | 12.95 (0 to 45.37) |
| American Samoa | 40-44 years | Females | 224.12 (-59.42 to 561.05) | 38.21 (-10.56 to 131.7) | 0 (0 to 0) |
| American Samoa | 45-49 years | Both sexes | 110.91 (-28.97 to 285.42) | 35.66 (-12.72 to 128.76) | 0.53 (0 to 3.6) |
| American Samoa | 45-49 years | Males | NA | 34.24 (-12.84 to 124.28) | 1.03 (0 to 6.96) |
| American Samoa | 45-49 years | Females | 229.44 (-59.92 to 590.42) | 37.18 (-12.35 to 134.25) | 0 (0 to 0) |
| American Samoa | 50-54 years | Both sexes | 104.81 (-20.99 to 283.74) | 37.4 (-12.09 to 149.21) | 0 (0 to 0) |
| American Samoa | 50-54 years | Males | NA | 34.92 (-13.31 to 143.08) | 0 (0 to 0) |
| American Samoa | 50-54 years | Females | 217.68 (-43.6 to 589.3) | 40.08 (-13.38 to 157.4) | 0 (0 to 0) |
| American Samoa | 55-59 years | Both sexes | 98.72 (-19.72 to 263.21) | 34.85 (-11.72 to 131.3) | 0 (0 to 0) |
| American Samoa | 55-59 years | Males | NA | 35.06 (-13.47 to 138.26) | 0 (0 to 0) |
| American Samoa | 55-59 years | Females | 205.77 (-41.11 to 548.62) | 34.63 (-10.69 to 132.23) | 0 (0 to 0) |
| American Samoa | 60-64 years | Both sexes | 85.68 (-16.08 to 244.95) | 34.7 (-13.66 to 137.33) | 0 (0 to 0) |
| American Samoa | 60-64 years | Males | NA | 35.07 (-15.07 to 140.5) | 0 (0 to 0) |
| American Samoa | 60-64 years | Females | 174.28 (-32.7 to 498.22) | 34.33 (-11.76 to 134.31) | 0 (0 to 0) |
| American Samoa | 65-69 years | Both sexes | 71.11 (-11.24 to 214.1) | 32.17 (-11.64 to 128.49) | 0 (0 to 0) |
| American Samoa | 65-69 years | Males | NA | 34.33 (-13.14 to 145.67) | 0 (0 to 0) |
| American Samoa | 65-69 years | Females | 141.08 (-22.3 to 424.76) | 30.05 (-9.74 to 116.51) | 0 (0 to 0) |
| American Samoa | 70-74 years | Both sexes | 67.22 (-9.18 to 209.39) | 25.39 (-8.3 to 107.2) | 0 (0 to 0) |
| American Samoa | 70-74 years | Males | NA | 29.49 (-8.71 to 135.79) | 0 (0 to 0) |
| American Samoa | 70-74 years | Females | 128.73 (-17.59 to 401.01) | 21.64 (-6.7 to 87.65) | 0 (0 to 0) |
| American Samoa | 75-79 years | Both sexes | 57.12 (-6.15 to 191.18) | 22.58 (-7.2 to 85.35) | 0 (0 to 0) |
| American Samoa | 75-79 years | Males | NA | 26.31 (-7.44 to 108.47) | 0 (0 to 0) |
| American Samoa | 75-79 years | Females | 102.94 (-11.07 to 344.52) | 19.59 (-6.22 to 73.86) | 0 (0 to 0) |
| American Samoa | 80-84 years | Both sexes | 43.31 (-4.73 to 156.72) | 13.96 (-3.53 to 62.37) | 0 (0 to 0) |
| American Samoa | 80-84 years | Males | NA | 19.13 (-4.97 to 80.27) | 0 (0 to 0) |
| American Samoa | 80-84 years | Females | 73.15 (-7.99 to 264.74) | 10.39 (-2.57 to 47.74) | 0 (0 to 0) |
| American Samoa | 85-89 years | Both sexes | 47.38 (-5.51 to 169.88) | 11.49 (-3.2 to 51.59) | 0 (0 to 0) |
| American Samoa | 85-89 years | Males | NA | 17.15 (-5.07 to 74.07) | 0 (0 to 0) |
| American Samoa | 85-89 years | Females | 70.48 (-8.2 to 252.71) | 8.73 (-2.29 to 40.67) | 0 (0 to 0) |
| American Samoa | 90-94 years | Both sexes | 52.49 (-6.34 to 184.53) | 9.74 (-2.68 to 43.89) | 0 (0 to 0) |
| American Samoa | 90-94 years | Males | NA | 15.9 (-4.85 to 72.88) | 0 (0 to 0) |
| American Samoa | 90-94 years | Females | 69.51 (-8.4 to 244.37) | 7.74 (-2.08 to 34.85) | 0 (0 to 0) |
| American Samoa | 95+ years | Both sexes | 56.55 (-6.4 to 207.02) | 8.92 (-2.71 to 39.01) | 0 (0 to 0) |
| American Samoa | 95+ years | Males | NA | 15.62 (-5.01 to 64.76) | 0 (0 to 0) |
| American Samoa | 95+ years | Females | 70.2 (-7.95 to 257.02) | 7.3 (-2.19 to 32.82) | 0 (0 to 0) |
| Andorra | <5 years | Both sexes | NA | NA | 0 (0 to 0) |
| Andorra | <5 years | Males | NA | NA | 0 (0 to 0) |
| Andorra | <5 years | Females | NA | NA | 0 (0 to 0) |
| Andorra | 5-9 years | Both sexes | NA | NA | 18.72 (5.56 to 48.49) |
| Andorra | 5-9 years | Males | NA | NA | 23.99 (6.59 to 65.27) |
| Andorra | 5-9 years | Females | NA | NA | 13.16 (3.72 to 33.02) |
| Andorra | 10-14 years | Both sexes | NA | NA | 180.58 (70.46 to 384.69) |
| Andorra | 10-14 years | Males | NA | NA | 157.42 (63.07 to 327.22) |
| Andorra | 10-14 years | Females | NA | NA | 203.83 (75.44 to 437.72) |
| Andorra | 15-19 years | Both sexes | 86.28 (-7.67 to 334.88) | 191.17 (-73.96 to 697.31) | 187.74 (73.11 to 374.71) |
| Andorra | 15-19 years | Males | NA | 106.26 (-34.47 to 413.73) | 183.49 (79.24 to 374.77) |
| Andorra | 15-19 years | Females | 183.88 (-16.34 to 713.73) | 287.22 (-118.63 to 1044.92) | 192.54 (66.54 to 409.84) |
| Andorra | 20-24 years | Both sexes | 135.82 (-17.4 to 425.43) | 157.52 (-53.86 to 553.95) | 123.81 (41.68 to 273.04) |
| Andorra | 20-24 years | Males | NA | 91.93 (-28.26 to 336.95) | 128.04 (49.57 to 268.71) |
| Andorra | 20-24 years | Females | 285.01 (-36.51 to 892.75) | 229.58 (-81.58 to 797.57) | 119.16 (33.83 to 269.44) |
| Andorra | 25-29 years | Both sexes | 152.63 (-23.01 to 430.18) | 118.54 (-38.5 to 410.09) | 77.77 (21.99 to 160.94) |
| Andorra | 25-29 years | Males | NA | 74.24 (-21.94 to 266.67) | 86.07 (27.54 to 176.06) |
| Andorra | 25-29 years | Females | 339.95 (-51.24 to 958.1) | 172.91 (-58.83 to 586.11) | 67.58 (8.83 to 162.4) |
| Andorra | 30-34 years | Both sexes | 149.63 (-25.1 to 449.28) | 109.27 (-40.31 to 408.71) | 48.52 (7.17 to 114.19) |
| Andorra | 30-34 years | Males | NA | 70.26 (-23.14 to 276.55) | 58.89 (12.41 to 134.21) |
| Andorra | 30-34 years | Females | 324.9 (-54.51 to 975.58) | 154.97 (-60.43 to 567.29) | 36.36 (0 to 102.42) |
| Andorra | 35-39 years | Both sexes | 136.01 (-24.13 to 406.29) | 100.05 (-38.15 to 372.33) | 21.7 (0 to 61.7) |
| Andorra | 35-39 years | Males | NA | 64.7 (-22.39 to 261.48) | 35.56 (0 to 94.19) |
| Andorra | 35-39 years | Females | 285.79 (-50.71 to 853.7) | 138.97 (-55.49 to 493.41) | 6.43 (0 to 27.74) |
| Andorra | 40-44 years | Both sexes | 123.29 (-20.75 to 354.46) | 94.13 (-37.6 to 306.66) | 8.2 (0 to 31.44) |
| Andorra | 40-44 years | Males | NA | 61.86 (-21.85 to 209.44) | 15.9 (0 to 60.97) |
| Andorra | 40-44 years | Females | 254.58 (-42.84 to 731.94) | 128.5 (-54.37 to 413.84) | 0 (0 to 0) |
| Andorra | 45-49 years | Both sexes | 109.58 (-18.26 to 315.12) | 85.95 (-33.37 to 293.49) | 0.53 (0 to 3.85) |
| Andorra | 45-49 years | Males | NA | 57.48 (-20.17 to 214.04) | 1.02 (0 to 7.44) |
| Andorra | 45-49 years | Females | 227.32 (-37.89 to 653.74) | 116.55 (-47.76 to 383.1) | 0 (0 to 0) |
| Andorra | 50-54 years | Both sexes | 84.63 (-12.19 to 258.97) | 80.22 (-35.09 to 294.65) | 0 (0 to 0) |
| Andorra | 50-54 years | Males | NA | 54.74 (-22.21 to 214.98) | 0 (0 to 0) |
| Andorra | 50-54 years | Females | 179.19 (-25.81 to 548.32) | 108.69 (-48.96 to 384.8) | 0 (0 to 0) |
| Andorra | 55-59 years | Both sexes | 71.74 (-9.82 to 216.28) | 75.89 (-31.01 to 285.77) | 0 (0 to 0) |
| Andorra | 55-59 years | Males | NA | 52.06 (-19.88 to 199.34) | 0 (0 to 0) |
| Andorra | 55-59 years | Females | 152.11 (-20.82 to 458.62) | 102.59 (-43.47 to 381.91) | 0 (0 to 0) |
| Andorra | 60-64 years | Both sexes | 53.44 (-7.36 to 190.01) | 75.31 (-35.48 to 293.38) | 0 (0 to 0) |
| Andorra | 60-64 years | Males | NA | 52.04 (-21.66 to 213.4) | 0 (0 to 0) |
| Andorra | 60-64 years | Females | 112.54 (-15.49 to 400.11) | 101.04 (-49.67 to 377.59) | 0 (0 to 0) |
| Andorra | 65-69 years | Both sexes | 42 (-4.66 to 139.29) | 77.18 (-30.25 to 284.73) | 0 (0 to 0) |
| Andorra | 65-69 years | Males | NA | 52.51 (-19.03 to 212.29) | 0 (0 to 0) |
| Andorra | 65-69 years | Females | 86.58 (-9.6 to 287.15) | 103.37 (-42.16 to 367.94) | 0 (0 to 0) |
| Andorra | 70-74 years | Both sexes | 40.27 (-4.25 to 134.77) | 72.73 (-23.01 to 285.04) | 0 (0 to 0) |
| Andorra | 70-74 years | Males | NA | 46.9 (-13.58 to 192.94) | 0 (0 to 0) |
| Andorra | 70-74 years | Females | 81.46 (-8.59 to 272.61) | 99.15 (-32.67 to 379.25) | 0 (0 to 0) |
| Andorra | 75-79 years | Both sexes | 32.97 (-3.04 to 121.27) | 67.91 (-21.08 to 265.04) | 0 (0 to 0) |
| Andorra | 75-79 years | Males | NA | 43.79 (-12.69 to 172.08) | 0 (0 to 0) |
| Andorra | 75-79 years | Females | 69.09 (-6.38 to 254.15) | 94.33 (-30.33 to 364.37) | 0 (0 to 0) |
| Andorra | 80-84 years | Both sexes | 27.3 (-2.29 to 109.7) | 50.57 (-15.65 to 200) | 0 (0 to 0) |
| Andorra | 80-84 years | Males | NA | 30.26 (-8.51 to 127.91) | 0 (0 to 0) |
| Andorra | 80-84 years | Females | 48.94 (-4.1 to 196.63) | 66.67 (-21.13 to 260.79) | 0 (0 to 0) |
| Andorra | 85-89 years | Both sexes | 25.22 (-2.26 to 104.74) | 40.69 (-12.21 to 168.78) | 0 (0 to 0) |
| Andorra | 85-89 years | Males | NA | 25.18 (-7.51 to 109.38) | 0 (0 to 0) |
| Andorra | 85-89 years | Females | 43.98 (-3.94 to 182.67) | 52.22 (-16.13 to 213.13) | 0 (0 to 0) |
| Andorra | 90-94 years | Both sexes | 23.45 (-2.07 to 96.19) | 34.11 (-10.49 to 146.88) | 0 (0 to 0) |
| Andorra | 90-94 years | Males | NA | 22.08 (-6.44 to 96.04) | 0 (0 to 0) |
| Andorra | 90-94 years | Females | 39.57 (-3.49 to 162.34) | 42.37 (-13.57 to 181.47) | 0 (0 to 0) |
| Andorra | 95+ years | Both sexes | 20.87 (-1.96 to 88.05) | 30.49 (-10.48 to 125.81) | 0 (0 to 0) |
| Andorra | 95+ years | Males | NA | 20.9 (-6.94 to 88.54) | 0 (0 to 0) |
| Andorra | 95+ years | Females | 37.09 (-3.49 to 156.49) | 37.94 (-13.23 to 154.45) | 0 (0 to 0) |
| Angola | <5 years | Both sexes | NA | NA | 0 (0 to 0) |
| Angola | <5 years | Males | NA | NA | 0 (0 to 0) |
| Angola | <5 years | Females | NA | NA | 0 (0 to 0) |
| Angola | 5-9 years | Both sexes | NA | NA | 11.65 (3.23 to 32.2) |
| Angola | 5-9 years | Males | NA | NA | 15.16 (3.88 to 38.75) |
| Angola | 5-9 years | Females | NA | NA | 8.16 (1.96 to 25.97) |
| Angola | 10-14 years | Both sexes | NA | NA | 172.51 (72.72 to 333.51) |
| Angola | 10-14 years | Males | NA | NA | 206.26 (89.53 to 384.25) |
| Angola | 10-14 years | Females | NA | NA | 139.35 (53.97 to 291.83) |
| Angola | 15-19 years | Both sexes | 67.67 (-7.27 to 222.52) | 115.62 (-38.97 to 432.17) | 183.67 (72.64 to 363.26) |
| Angola | 15-19 years | Males | NA | 102.4 (-34.59 to 406.73) | 223.32 (90.3 to 442.76) |
| Angola | 15-19 years | Females | 132.8 (-14.26 to 436.71) | 128.34 (-43.53 to 456.94) | 145.5 (52.76 to 312) |
| Angola | 20-24 years | Both sexes | 150.96 (-31.72 to 412.14) | 112.6 (-35.03 to 401.12) | 133.54 (48.08 to 280.36) |
| Angola | 20-24 years | Males | NA | 92.01 (-29.44 to 327.35) | 163.69 (61.28 to 345.54) |
| Angola | 20-24 years | Females | 292.26 (-61.4 to 797.9) | 131.88 (-40.3 to 472.36) | 105.32 (29.57 to 250.46) |
| Angola | 25-29 years | Both sexes | 168.33 (-38.23 to 440.11) | 104.15 (-32.76 to 387.98) | 89.89 (25.2 to 192.2) |
| Angola | 25-29 years | Males | NA | 83.26 (-24.28 to 317.46) | 116.8 (38.57 to 232.8) |
| Angola | 25-29 years | Females | 321.12 (-72.94 to 839.6) | 123.11 (-40.46 to 470.32) | 65.47 (8.66 to 149.85) |
| Angola | 30-34 years | Both sexes | 169.62 (-33.86 to 459) | 100.04 (-35.84 to 400.68) | 54.55 (11.44 to 118.38) |
| Angola | 30-34 years | Males | NA | 81.7 (-26.52 to 334.57) | 78.79 (17.82 to 167.44) |
| Angola | 30-34 years | Females | 317.41 (-63.36 to 858.92) | 116.02 (-43.96 to 456.64) | 33.43 (0 to 91.26) |
| Angola | 35-39 years | Both sexes | 166.01 (-43.92 to 413.21) | 94.14 (-35.06 to 356.11) | 27.7 (0.3 to 66.25) |
| Angola | 35-39 years | Males | NA | 78.85 (-28.74 to 294.62) | 51.38 (0.5 to 119.79) |
| Angola | 35-39 years | Females | 311 (-82.27 to 774.11) | 107.49 (-40.7 to 410.11) | 7.01 (0 to 25.98) |
| Angola | 40-44 years | Both sexes | 148.88 (-29.16 to 400.43) | 93.27 (-36.95 to 334.74) | 11.94 (0 to 41.78) |
| Angola | 40-44 years | Males | NA | 79.25 (-29.28 to 275.01) | 25.43 (0 to 88.95) |
| Angola | 40-44 years | Females | 280.77 (-54.98 to 755.17) | 105.68 (-43.35 to 382.99) | 0 (0 to 0) |
| Angola | 45-49 years | Both sexes | 147.71 (-26.94 to 411.57) | 92.48 (-37.01 to 329.22) | 1.16 (0 to 7.09) |
| Angola | 45-49 years | Males | NA | 78.91 (-30 to 285.52) | 2.47 (0 to 15.09) |
| Angola | 45-49 years | Females | 278.53 (-50.8 to 776.09) | 104.49 (-43.09 to 372.83) | 0 (0 to 0) |
| Angola | 50-54 years | Both sexes | 143.28 (-25.35 to 410.66) | 92.9 (-40.21 to 368.93) | 0 (0 to 0) |
| Angola | 50-54 years | Males | NA | 79.49 (-32.43 to 319.71) | 0 (0 to 0) |
| Angola | 50-54 years | Females | 273.29 (-48.34 to 783.27) | 105.06 (-47.01 to 413.59) | 0 (0 to 0) |
| Angola | 55-59 years | Both sexes | 138.42 (-25.11 to 393.67) | 92.54 (-41.01 to 351.39) | 0 (0 to 0) |
| Angola | 55-59 years | Males | NA | 79.38 (-32.64 to 305.88) | 0 (0 to 0) |
| Angola | 55-59 years | Females | 259.29 (-47.03 to 737.42) | 104.02 (-48.2 to 391.96) | 0 (0 to 0) |
| Angola | 60-64 years | Both sexes | 118.25 (-19.39 to 355.58) | 92.27 (-42.57 to 375.5) | 0 (0 to 0) |
| Angola | 60-64 years | Males | NA | 79.07 (-33.67 to 318.42) | 0 (0 to 0) |
| Angola | 60-64 years | Females | 214.28 (-35.13 to 644.34) | 103 (-49.79 to 421.35) | 0 (0 to 0) |
| Angola | 65-69 years | Both sexes | 97.2 (-12.57 to 314.49) | 88.71 (-36.99 to 364.55) | 0 (0 to 0) |
| Angola | 65-69 years | Males | NA | 76.35 (-31.27 to 314.51) | 0 (0 to 0) |
| Angola | 65-69 years | Females | 172.59 (-22.32 to 558.42) | 98.29 (-41.43 to 405.5) | 0 (0 to 0) |
| Angola | 70-74 years | Both sexes | 93.7 (-10.34 to 319.22) | 76.74 (-23.92 to 327.12) | 0 (0 to 0) |
| Angola | 70-74 years | Males | NA | 66.53 (-20.6 to 282.72) | 0 (0 to 0) |
| Angola | 70-74 years | Females | 159.52 (-17.6 to 543.44) | 83.91 (-26.73 to 359.52) | 0 (0 to 0) |
| Angola | 75-79 years | Both sexes | 79.41 (-8.44 to 264.46) | 69.3 (-21.39 to 282.12) | 0 (0 to 0) |
| Angola | 75-79 years | Males | NA | 60.27 (-17.02 to 243.88) | 0 (0 to 0) |
| Angola | 75-79 years | Females | 128.36 (-13.64 to 427.47) | 74.87 (-24.07 to 303.31) | 0 (0 to 0) |
| Angola | 80-84 years | Both sexes | 58.93 (-5.75 to 226.43) | 51.19 (-14.17 to 212.89) | 0 (0 to 0) |
| Angola | 80-84 years | Males | NA | 44.47 (-12.2 to 183.07) | 0 (0 to 0) |
| Angola | 80-84 years | Females | 91.7 (-8.94 to 352.36) | 54.93 (-15.08 to 229.95) | 0 (0 to 0) |
| Angola | 85-89 years | Both sexes | 60.53 (-6.53 to 224.14) | 45.8 (-13.95 to 189.68) | 0 (0 to 0) |
| Angola | 85-89 years | Males | NA | 40.09 (-12.5 to 167.63) | 0 (0 to 0) |
| Angola | 85-89 years | Females | 89.22 (-9.63 to 330.38) | 48.51 (-14.91 to 200.78) | 0 (0 to 0) |
| Angola | 90-94 years | Both sexes | 66.05 (-6.8 to 251.95) | 42.93 (-14.39 to 179.29) | 0 (0 to 0) |
| Angola | 90-94 years | Males | NA | 37.49 (-12.53 to 163.4) | 0 (0 to 0) |
| Angola | 90-94 years | Females | 89.42 (-9.21 to 341.05) | 44.86 (-15.04 to 185.66) | 0 (0 to 0) |
| Angola | 95+ years | Both sexes | 73.99 (-7.63 to 278.07) | 42.93 (-14.64 to 181.33) | 0 (0 to 0) |
| Angola | 95+ years | Males | NA | 36.87 (-12.8 to 148.66) | 0 (0 to 0) |
| Angola | 95+ years | Females | 91.12 (-9.4 to 342.44) | 44.33 (-15.38 to 188.6) | 0 (0 to 0) |
| Antigua and Barbuda | <5 years | Both sexes | NA | NA | 0 (0 to 0) |
| Antigua and Barbuda | <5 years | Males | NA | NA | 0 (0 to 0) |
| Antigua and Barbuda | <5 years | Females | NA | NA | 0 (0 to 0) |
| Antigua and Barbuda | 5-9 years | Both sexes | NA | NA | 5.29 (1.36 to 15.24) |
| Antigua and Barbuda | 5-9 years | Males | NA | NA | 5.83 (1.47 to 16.09) |
| Antigua and Barbuda | 5-9 years | Females | NA | NA | 4.73 (1.03 to 14.16) |
| Antigua and Barbuda | 10-14 years | Both sexes | NA | NA | 70.93 (24.34 to 155.65) |
| Antigua and Barbuda | 10-14 years | Males | NA | NA | 69.77 (24.49 to 155.35) |
| Antigua and Barbuda | 10-14 years | Females | NA | NA | 72.13 (21.11 to 158.97) |
| Antigua and Barbuda | 15-19 years | Both sexes | 41.15 (-3.83 to 161.11) | 71.56 (-25.17 to 289.82) | 78.27 (27.82 to 168.2) |
| Antigua and Barbuda | 15-19 years | Males | NA | 48.16 (-16.9 to 189.69) | 63.93 (24.06 to 133.04) |
| Antigua and Barbuda | 15-19 years | Females | 84.17 (-7.83 to 329.56) | 96.02 (-33.01 to 384.58) | 93.27 (30.23 to 213.47) |
| Antigua and Barbuda | 20-24 years | Both sexes | 108.58 (-16.01 to 366.63) | 87.03 (-23.32 to 336.49) | 77.31 (26.43 to 178.69) |
| Antigua and Barbuda | 20-24 years | Males | NA | 55.51 (-15.05 to 202.5) | 61.52 (22.91 to 138.53) |
| Antigua and Barbuda | 20-24 years | Females | 221.36 (-32.63 to 747.42) | 119.77 (-31.93 to 476.94) | 93.7 (27.3 to 232.47) |
| Antigua and Barbuda | 25-29 years | Both sexes | 155.99 (-30.93 to 487.62) | 94.28 (-27.81 to 368.15) | 64.57 (17.44 to 147.75) |
| Antigua and Barbuda | 25-29 years | Males | NA | 58.74 (-15.76 to 232.15) | 53.18 (17.1 to 111.49) |
| Antigua and Barbuda | 25-29 years | Females | 315.78 (-62.61 to 987.11) | 130.7 (-40.24 to 507.46) | 76.25 (12.31 to 195.49) |
| Antigua and Barbuda | 30-34 years | Both sexes | 174.27 (-32.97 to 536.12) | 102.83 (-34.8 to 417.47) | 44.84 (5.56 to 110.38) |
| Antigua and Barbuda | 30-34 years | Males | NA | 63.6 (-20.25 to 264.7) | 40.94 (9.59 to 97.09) |
| Antigua and Barbuda | 30-34 years | Females | 343.66 (-65.02 to 1057.22) | 140.96 (-49.14 to 549.63) | 48.63 (0 to 139.87) |
| Antigua and Barbuda | 35-39 years | Both sexes | 177 (-36.65 to 511.89) | 101.08 (-35.78 to 414.04) | 19.14 (0.09 to 54.31) |
| Antigua and Barbuda | 35-39 years | Males | NA | 61.5 (-21.49 to 246.39) | 27.13 (0.19 to 68.88) |
| Antigua and Barbuda | 35-39 years | Females | 338.8 (-70.16 to 979.84) | 137.27 (-48.85 to 548.58) | 11.83 (0 to 44.77) |
| Antigua and Barbuda | 40-44 years | Both sexes | 167.11 (-33.7 to 456.17) | 97.89 (-36.42 to 345.48) | 6.4 (0 to 22.53) |
| Antigua and Barbuda | 40-44 years | Males | NA | 60.3 (-21.43 to 218.16) | 13.3 (0 to 46.78) |
| Antigua and Barbuda | 40-44 years | Females | 322.36 (-65.01 to 879.99) | 132.82 (-50.76 to 467.71) | 0 (0 to 0) |
| Antigua and Barbuda | 45-49 years | Both sexes | 163.07 (-30.61 to 443.84) | 93.44 (-35.41 to 340.71) | 0.54 (0 to 3.8) |
| Antigua and Barbuda | 45-49 years | Males | NA | 58.73 (-21.01 to 217.27) | 1.14 (0 to 8.02) |
| Antigua and Barbuda | 45-49 years | Females | 310 (-58.2 to 843.76) | 124.72 (-48.53 to 458.91) | 0 (0 to 0) |
| Antigua and Barbuda | 50-54 years | Both sexes | 139.5 (-22.92 to 409.26) | 89.94 (-36.97 to 351.35) | 0 (0 to 0) |
| Antigua and Barbuda | 50-54 years | Males | NA | 57.75 (-20.23 to 240.2) | 0 (0 to 0) |
| Antigua and Barbuda | 50-54 years | Females | 266.74 (-43.83 to 782.55) | 119.3 (-49.72 to 452.73) | 0 (0 to 0) |
| Antigua and Barbuda | 55-59 years | Both sexes | 122.8 (-18.86 to 361.96) | 84.49 (-36.72 to 342.9) | 0 (0 to 0) |
| Antigua and Barbuda | 55-59 years | Males | NA | 55.58 (-23.02 to 235.24) | 0 (0 to 0) |
| Antigua and Barbuda | 55-59 years | Females | 235.87 (-36.22 to 695.24) | 111.12 (-49.33 to 444.41) | 0 (0 to 0) |
| Antigua and Barbuda | 60-64 years | Both sexes | 93.7 (-13.77 to 289.7) | 79.64 (-36.23 to 325.04) | 0 (0 to 0) |
| Antigua and Barbuda | 60-64 years | Males | NA | 54.07 (-22.43 to 228.11) | 0 (0 to 0) |
| Antigua and Barbuda | 60-64 years | Females | 180.42 (-26.51 to 557.84) | 103.3 (-49 to 415.76) | 0 (0 to 0) |
| Antigua and Barbuda | 65-69 years | Both sexes | 69.91 (-8.67 to 227.89) | 73.44 (-28.82 to 299.68) | 0 (0 to 0) |
| Antigua and Barbuda | 65-69 years | Males | NA | 50.55 (-18.89 to 214.84) | 0 (0 to 0) |
| Antigua and Barbuda | 65-69 years | Females | 135.65 (-16.82 to 442.15) | 94.96 (-38.17 to 381.16) | 0 (0 to 0) |
| Antigua and Barbuda | 70-74 years | Both sexes | 61.87 (-6.5 to 211.8) | 60.11 (-18.33 to 265.45) | 0 (0 to 0) |
| Antigua and Barbuda | 70-74 years | Males | NA | 42.01 (-12.62 to 187.82) | 0 (0 to 0) |
| Antigua and Barbuda | 70-74 years | Females | 118.39 (-12.43 to 405.25) | 76.64 (-23.45 to 335.62) | 0 (0 to 0) |
| Antigua and Barbuda | 75-79 years | Both sexes | 48.69 (-5.03 to 167.09) | 52.51 (-15.47 to 213.07) | 0 (0 to 0) |
| Antigua and Barbuda | 75-79 years | Males | NA | 37.18 (-9.84 to 156.38) | 0 (0 to 0) |
| Antigua and Barbuda | 75-79 years | Females | 90.62 (-9.35 to 311.02) | 65.72 (-20.44 to 259) | 0 (0 to 0) |
| Antigua and Barbuda | 80-84 years | Both sexes | 36.09 (-3.42 to 140.4) | 38.03 (-10.69 to 164.2) | 0 (0 to 0) |
| Antigua and Barbuda | 80-84 years | Males | NA | 26.04 (-6.53 to 111.33) | 0 (0 to 0) |
| Antigua and Barbuda | 80-84 years | Females | 61.99 (-5.88 to 241.13) | 46.63 (-13.32 to 202.27) | 0 (0 to 0) |
| Antigua and Barbuda | 85-89 years | Both sexes | 36.19 (-3.44 to 145.09) | 34.03 (-9.58 to 139.07) | 0 (0 to 0) |
| Antigua and Barbuda | 85-89 years | Males | NA | 23.28 (-6.57 to 100.59) | 0 (0 to 0) |
| Antigua and Barbuda | 85-89 years | Females | 58.9 (-5.6 to 236.17) | 40.78 (-11.63 to 163.57) | 0 (0 to 0) |
| Antigua and Barbuda | 90-94 years | Both sexes | 42.28 (-4.24 to 165.23) | 32.64 (-9.78 to 139.76) | 0 (0 to 0) |
| Antigua and Barbuda | 90-94 years | Males | NA | 21.25 (-6.38 to 91.94) | 0 (0 to 0) |
| Antigua and Barbuda | 90-94 years | Females | 58.38 (-5.86 to 228.14) | 36.97 (-11.22 to 155.79) | 0 (0 to 0) |
| Antigua and Barbuda | 95+ years | Both sexes | 41.93 (-4.07 to 168.13) | 31.2 (-11.17 to 125.62) | 0 (0 to 0) |
| Antigua and Barbuda | 95+ years | Males | NA | 20.73 (-6.62 to 87.11) | 0 (0 to 0) |
| Antigua and Barbuda | 95+ years | Females | 58.28 (-5.66 to 233.7) | 35.28 (-12.95 to 141) | 0 (0 to 0) |
| Argentina | <5 years | Both sexes | NA | NA | 0 (0 to 0) |
| Argentina | <5 years | Males | NA | NA | 0 (0 to 0) |
| Argentina | <5 years | Females | NA | NA | 0 (0 to 0) |
| Argentina | 5-9 years | Both sexes | NA | NA | 19.05 (3.98 to 58.39) |
| Argentina | 5-9 years | Males | NA | NA | 19.26 (4.17 to 58.08) |
| Argentina | 5-9 years | Females | NA | NA | 18.83 (3.61 to 67.36) |
| Argentina | 10-14 years | Both sexes | NA | NA | 128.27 (43.32 to 280.16) |
| Argentina | 10-14 years | Males | NA | NA | 138.5 (48.46 to 285.95) |
| Argentina | 10-14 years | Females | NA | NA | 117.74 (34.89 to 279.95) |
| Argentina | 15-19 years | Both sexes | 97.78 (-10 to 331.98) | 117.2 (-42.07 to 450.41) | 151.13 (53.32 to 328.9) |
| Argentina | 15-19 years | Males | NA | 83.97 (-30.32 to 322.84) | 157.01 (57.19 to 316.81) |
| Argentina | 15-19 years | Females | 197.2 (-20.17 to 669.53) | 150.98 (-53.68 to 585.13) | 145.15 (44.15 to 337.37) |
| Argentina | 20-24 years | Both sexes | 231.46 (-53.72 to 615.79) | 130.17 (-43.91 to 484.29) | 132.11 (47.47 to 309.15) |
| Argentina | 20-24 years | Males | NA | 85.28 (-25.75 to 324.36) | 136.13 (54.25 to 273.35) |
| Argentina | 20-24 years | Females | 460.86 (-106.97 to 1226.1) | 174.66 (-61.92 to 631.68) | 128.13 (38.97 to 314.93) |
| Argentina | 25-29 years | Both sexes | 278.48 (-78.62 to 690.05) | 124.67 (-40.04 to 468.33) | 105.18 (28.41 to 229.15) |
| Argentina | 25-29 years | Males | NA | 90.15 (-28.78 to 339.02) | 112.75 (34.68 to 226.51) |
| Argentina | 25-29 years | Females | 548.44 (-154.84 to 1358.96) | 158.13 (-51.71 to 594) | 97.85 (14.63 to 247.37) |
| Argentina | 30-34 years | Both sexes | 282.44 (-73.69 to 663.95) | 133.21 (-52.06 to 502.67) | 78.6 (12.71 to 179.35) |
| Argentina | 30-34 years | Males | NA | 86.02 (-30.86 to 332.58) | 91.32 (23.82 to 189.38) |
| Argentina | 30-34 years | Females | 553.11 (-144.31 to 1300.24) | 178.44 (-73.29 to 665.67) | 66.41 (0 to 177.38) |
| Argentina | 35-39 years | Both sexes | 269.92 (-74.81 to 625.61) | 121.68 (-48 to 466.72) | 37.69 (0.12 to 97.96) |
| Argentina | 35-39 years | Males | NA | 82.19 (-31.12 to 307.13) | 61.34 (0.22 to 141.62) |
| Argentina | 35-39 years | Females | 528.01 (-146.34 to 1223.81) | 159.43 (-64.77 to 609.16) | 15.08 (0 to 57.06) |
| Argentina | 40-44 years | Both sexes | 253.45 (-71.66 to 578.34) | 123.9 (-53.71 to 425.6) | 12.87 (0 to 44.88) |
| Argentina | 40-44 years | Males | NA | 82.99 (-31.62 to 284.02) | 26.49 (0 to 92.37) |
| Argentina | 40-44 years | Females | 492.98 (-139.39 to 1124.93) | 162.56 (-74.12 to 561.33) | 0 (0 to 0) |
| Argentina | 45-49 years | Both sexes | 236.7 (-55.26 to 532.88) | 113.72 (-47.89 to 383.07) | 0.94 (0 to 5.64) |
| Argentina | 45-49 years | Males | NA | 77.79 (-28.87 to 268.91) | 1.93 (0 to 11.66) |
| Argentina | 45-49 years | Females | 458.33 (-107 to 1031.81) | 147.36 (-63.59 to 503.15) | 0 (0 to 0) |
| Argentina | 50-54 years | Both sexes | 191.49 (-31.47 to 486.73) | 112.51 (-50.61 to 424.2) | 0 (0 to 0) |
| Argentina | 50-54 years | Males | NA | 77.32 (-31.13 to 302.16) | 0 (0 to 0) |
| Argentina | 50-54 years | Females | 367.58 (-60.42 to 934.31) | 144.87 (-68.01 to 531.91) | 0 (0 to 0) |
| Argentina | 55-59 years | Both sexes | 166.37 (-24.67 to 451.87) | 102.12 (-46.49 to 385.68) | 0 (0 to 0) |
| Argentina | 55-59 years | Males | NA | 71.55 (-31.07 to 269.29) | 0 (0 to 0) |
| Argentina | 55-59 years | Females | 316.53 (-46.94 to 859.72) | 129.72 (-62.98 to 491.19) | 0 (0 to 0) |
| Argentina | 60-64 years | Both sexes | 122.67 (-16.29 to 344.67) | 93.77 (-43.12 to 339.72) | 0 (0 to 0) |
| Argentina | 60-64 years | Males | NA | 69.98 (-31.81 to 261.68) | 0 (0 to 0) |
| Argentina | 60-64 years | Females | 230.38 (-30.59 to 647.29) | 114.66 (-53.06 to 415.1) | 0 (0 to 0) |
| Argentina | 65-69 years | Both sexes | 91.8 (-12.69 to 290.62) | 89.05 (-37.23 to 353.77) | 0 (0 to 0) |
| Argentina | 65-69 years | Males | NA | 62.85 (-25.55 to 251.68) | 0 (0 to 0) |
| Argentina | 65-69 years | Females | 169.03 (-23.36 to 535.12) | 111.09 (-47.06 to 435.17) | 0 (0 to 0) |
| Argentina | 70-74 years | Both sexes | 97.54 (-11.08 to 306.59) | 73.71 (-23.12 to 327.95) | 0 (0 to 0) |
| Argentina | 70-74 years | Males | NA | 52.13 (-16.14 to 228.06) | 0 (0 to 0) |
| Argentina | 70-74 years | Females | 174.28 (-19.8 to 547.83) | 90.68 (-29.03 to 403.16) | 0 (0 to 0) |
| Argentina | 75-79 years | Both sexes | 79.45 (-8.2 to 271.31) | 63.82 (-19.41 to 245.58) | 0 (0 to 0) |
| Argentina | 75-79 years | Males | NA | 45.11 (-13.65 to 172.57) | 0 (0 to 0) |
| Argentina | 75-79 years | Females | 135.98 (-14.04 to 464.35) | 77.13 (-23.94 to 302.24) | 0 (0 to 0) |
| Argentina | 80-84 years | Both sexes | 58.02 (-5.81 to 214.08) | 46.58 (-12.92 to 197.89) | 0 (0 to 0) |
| Argentina | 80-84 years | Males | NA | 32.04 (-9.36 to 134.49) | 0 (0 to 0) |
| Argentina | 80-84 years | Females | 93.66 (-9.38 to 345.59) | 55.51 (-15.54 to 243.19) | 0 (0 to 0) |
| Argentina | 85-89 years | Both sexes | 58.85 (-6.05 to 213.61) | 41.42 (-12.77 to 172.25) | 0 (0 to 0) |
| Argentina | 85-89 years | Males | NA | 28.52 (-8.35 to 116.6) | 0 (0 to 0) |
| Argentina | 85-89 years | Females | 89.08 (-9.16 to 323.35) | 48.05 (-15.12 to 205.51) | 0 (0 to 0) |
| Argentina | 90-94 years | Both sexes | 61.14 (-6.8 to 221.41) | 37.95 (-11.55 to 167.33) | 0 (0 to 0) |
| Argentina | 90-94 years | Males | NA | 26.19 (-8.44 to 115.09) | 0 (0 to 0) |
| Argentina | 90-94 years | Females | 85.93 (-9.56 to 311.18) | 42.72 (-12.89 to 189.84) | 0 (0 to 0) |
| Argentina | 95+ years | Both sexes | 63.6 (-7 to 231.49) | 37.1 (-12.88 to 146.92) | 0 (0 to 0) |
| Argentina | 95+ years | Males | NA | 25.84 (-8.61 to 106.55) | 0 (0 to 0) |
| Argentina | 95+ years | Females | 85.89 (-9.46 to 312.63) | 41.04 (-14.4 to 161.2) | 0 (0 to 0) |
| Armenia | <5 years | Both sexes | NA | NA | 0 (0 to 0) |
| Armenia | <5 years | Males | NA | NA | 0 (0 to 0) |
| Armenia | <5 years | Females | NA | NA | 0 (0 to 0) |
| Armenia | 5-9 years | Both sexes | NA | NA | 2.42 (0.6 to 7.43) |
| Armenia | 5-9 years | Males | NA | NA | 3.17 (0.77 to 9.44) |
| Armenia | 5-9 years | Females | NA | NA | 1.58 (0.29 to 5.26) |
| Armenia | 10-14 years | Both sexes | NA | NA | 32.23 (11.72 to 71.89) |
| Armenia | 10-14 years | Males | NA | NA | 41.91 (14.27 to 90.24) |
| Armenia | 10-14 years | Females | NA | NA | 21.34 (7.43 to 51.45) |
| Armenia | 15-19 years | Both sexes | 12.04 (-0.92 to 51.53) | 55.96 (-17.97 to 217.94) | 30.42 (10.89 to 64.55) |
| Armenia | 15-19 years | Males | NA | 39.34 (-11.83 to 170.57) | 41.73 (14.49 to 86.52) |
| Armenia | 15-19 years | Females | 24.85 (-1.89 to 106.37) | 73.65 (-25.65 to 292.92) | 18.39 (5.26 to 42.23) |
| Armenia | 20-24 years | Both sexes | 22.92 (-2.3 to 93.84) | 54.51 (-17.54 to 190.68) | 20.09 (7 to 48.34) |
| Armenia | 20-24 years | Males | NA | 34.23 (-8.95 to 143.44) | 28.1 (9.85 to 64.3) |
| Armenia | 20-24 years | Females | 46.01 (-4.62 to 188.39) | 74.95 (-24.06 to 242.19) | 12.01 (3.08 to 30.01) |
| Armenia | 25-29 years | Both sexes | 35.59 (-4.85 to 129.64) | 50.92 (-16.3 to 154.22) | 13.66 (3.84 to 30.72) |
| Armenia | 25-29 years | Males | NA | 30.21 (-7.4 to 132.51) | 19.51 (5.85 to 41.63) |
| Armenia | 25-29 years | Females | 71.1 (-9.68 to 259.01) | 71.58 (-21.43 to 237.41) | 7.83 (1.24 to 20.45) |
| Armenia | 30-34 years | Both sexes | 44.56 (-4.9 to 143.49) | 50.06 (-15.27 to 163.91) | 9.38 (1.68 to 22.66) |
| Armenia | 30-34 years | Males | NA | 30.85 (-9.09 to 142.2) | 14.3 (2.99 to 32.95) |
| Armenia | 30-34 years | Females | 87.27 (-9.6 to 281.01) | 68.46 (-18.51 to 229.33) | 4.67 (0 to 14.13) |
| Armenia | 35-39 years | Both sexes | 73.32 (-9.55 to 205.81) | 53.94 (-17.33 to 178.47) | 5.12 (0.04 to 13.1) |
| Armenia | 35-39 years | Males | NA | 29.77 (-8.86 to 129.26) | 9.61 (0.06 to 24.37) |
| Armenia | 35-39 years | Females | 141.17 (-18.4 to 396.27) | 76.3 (-22.43 to 239.96) | 0.97 (0 to 4.11) |
| Armenia | 40-44 years | Both sexes | 56.29 (-5.81 to 177.6) | 54.24 (-15.83 to 169.37) | 2.02 (0 to 7.18) |
| Armenia | 40-44 years | Males | NA | 30.16 (-9.18 to 119.4) | 4.26 (0 to 15.16) |
| Armenia | 40-44 years | Females | 106.88 (-11.03 to 337.19) | 75.89 (-21.28 to 241.14) | 0 (0 to 0) |
| Armenia | 45-49 years | Both sexes | 76.32 (-8.16 to 232.45) | 58.92 (-19 to 197.04) | 0.15 (0 to 1.17) |
| Armenia | 45-49 years | Males | NA | 30.55 (-9.8 to 133.06) | 0.33 (0 to 2.52) |
| Armenia | 45-49 years | Females | 142.06 (-15.19 to 432.71) | 83.37 (-29.46 to 273.31) | 0 (0 to 0) |
| Armenia | 50-54 years | Both sexes | 62.04 (-8.57 to 209.43) | 62.13 (-21.28 to 236.65) | 0 (0 to 0) |
| Armenia | 50-54 years | Males | NA | 31.79 (-10.07 to 144.49) | 0 (0 to 0) |
| Armenia | 50-54 years | Females | 114.04 (-15.75 to 384.95) | 87.55 (-31.66 to 307.97) | 0 (0 to 0) |
| Armenia | 55-59 years | Both sexes | 57.1 (-7.34 to 207.88) | 58.85 (-18.8 to 211.99) | 0 (0 to 0) |
| Armenia | 55-59 years | Males | NA | 31.89 (-11.74 to 140.54) | 0 (0 to 0) |
| Armenia | 55-59 years | Females | 102.82 (-13.22 to 374.32) | 80.43 (-29.53 to 277.73) | 0 (0 to 0) |
| Armenia | 60-64 years | Both sexes | 38.07 (-3.79 to 148.02) | 56.13 (-20.29 to 241.97) | 0 (0 to 0) |
| Armenia | 60-64 years | Males | NA | 32.41 (-11.89 to 149.49) | 0 (0 to 0) |
| Armenia | 60-64 years | Females | 67.65 (-6.73 to 263.02) | 74.56 (-26.82 to 314.08) | 0 (0 to 0) |
| Armenia | 65-69 years | Both sexes | 28.71 (-2.35 to 112.3) | 54.13 (-20.42 to 242.52) | 0 (0 to 0) |
| Armenia | 65-69 years | Males | NA | 31.4 (-10.92 to 138.03) | 0 (0 to 0) |
| Armenia | 65-69 years | Females | 49.84 (-4.08 to 194.99) | 70.87 (-27.42 to 319.92) | 0 (0 to 0) |
| Armenia | 70-74 years | Both sexes | 26.32 (-1.94 to 113.66) | 46.47 (-13.45 to 219.43) | 0 (0 to 0) |
| Armenia | 70-74 years | Males | NA | 26.34 (-7.05 to 125.1) | 0 (0 to 0) |
| Armenia | 70-74 years | Females | 44.38 (-3.28 to 191.68) | 60.28 (-17.89 to 283.81) | 0 (0 to 0) |
| Armenia | 75-79 years | Both sexes | 20.33 (-1.48 to 85.14) | 41.33 (-11.56 to 173.46) | 0 (0 to 0) |
| Armenia | 75-79 years | Males | NA | 23.19 (-5.76 to 105.75) | 0 (0 to 0) |
| Armenia | 75-79 years | Females | 33.26 (-2.42 to 139.26) | 52.87 (-15.13 to 215.26) | 0 (0 to 0) |
| Armenia | 80-84 years | Both sexes | 14.22 (-1.01 to 64.86) | 30.14 (-8.1 to 134.27) | 0 (0 to 0) |
| Armenia | 80-84 years | Males | NA | 16.24 (-4.09 to 73.48) | 0 (0 to 0) |
| Armenia | 80-84 years | Females | 21.88 (-1.56 to 99.79) | 37.63 (-10.14 to 168.01) | 0 (0 to 0) |
| Armenia | 85-89 years | Both sexes | 13.82 (-1.02 to 59.16) | 27.07 (-7.73 to 128.42) | 0 (0 to 0) |
| Armenia | 85-89 years | Males | NA | 14.68 (-4.01 to 65.43) | 0 (0 to 0) |
| Armenia | 85-89 years | Females | 21.33 (-1.57 to 91.3) | 33.8 (-10.1 to 160.32) | 0 (0 to 0) |
| Armenia | 90-94 years | Both sexes | 12.26 (-0.98 to 52.44) | 23.4 (-6.94 to 108.12) | 0 (0 to 0) |
| Armenia | 90-94 years | Males | NA | 13.66 (-3.88 to 63.46) | 0 (0 to 0) |
| Armenia | 90-94 years | Females | 21.23 (-1.7 to 90.78) | 30.52 (-9.22 to 140.78) | 0 (0 to 0) |
| Armenia | 95+ years | Both sexes | 11.49 (-0.96 to 50.25) | 22.2 (-7.25 to 99.06) | 0 (0 to 0) |
| Armenia | 95+ years | Males | NA | 13.45 (-4.2 to 60.82) | 0 (0 to 0) |
| Armenia | 95+ years | Females | 21.37 (-1.79 to 93.43) | 29.71 (-9.88 to 130.47) | 0 (0 to 0) |
| Australia | <5 years | Both sexes | NA | NA | 0 (0 to 0) |
| Australia | <5 years | Males | NA | NA | 0 (0 to 0) |
| Australia | <5 years | Females | NA | NA | 0 (0 to 0) |
| Australia | 5-9 years | Both sexes | NA | NA | 36.29 (9.06 to 104.83) |
| Australia | 5-9 years | Males | NA | NA | 51.36 (13.45 to 151.63) |
| Australia | 5-9 years | Females | NA | NA | 20.33 (4.35 to 65.41) |
| Australia | 10-14 years | Both sexes | NA | NA | 156.43 (61.78 to 306.36) |
| Australia | 10-14 years | Males | NA | NA | 201.99 (79.36 to 381.76) |
| Australia | 10-14 years | Females | NA | NA | 108.15 (36.71 to 225.66) |
| Australia | 15-19 years | Both sexes | 83.35 (-7.56 to 297.15) | 189.74 (-73.02 to 605.97) | 226.07 (86.73 to 454.91) |
| Australia | 15-19 years | Males | NA | 98.43 (-35.04 to 349.81) | 245.52 (92.89 to 488.66) |
| Australia | 15-19 years | Females | 171.85 (-15.59 to 612.64) | 286.67 (-122.34 to 929.76) | 205.41 (66.99 to 450.71) |
| Australia | 20-24 years | Both sexes | 331.84 (-65.59 to 899.93) | 311.2 (-138.87 to 876.18) | 232.35 (85.6 to 512.18) |
| Australia | 20-24 years | Males | NA | 211.81 (-84.16 to 629.46) | 257.07 (108.25 to 560.61) |
| Australia | 20-24 years | Females | 679.66 (-134.34 to 1843.18) | 415.37 (-186.56 to 1176.62) | 206.45 (52.97 to 472.14) |
| Australia | 25-29 years | Both sexes | 384.6 (-92.16 to 1001.03) | 274.68 (-111.91 to 784.73) | 175.99 (49.08 to 389.41) |
| Australia | 25-29 years | Males | NA | 128.93 (-47.51 to 444.84) | 199.68 (67.42 to 432.69) |
| Australia | 25-29 years | Females | 774.85 (-185.67 to 2016.79) | 422.58 (-177.48 to 1247.11) | 151.95 (19.47 to 383.47) |
| Australia | 30-34 years | Both sexes | 324.94 (-81.31 to 827.9) | 270.5 (-112.13 to 780.22) | 98.5 (18.14 to 214.96) |
| Australia | 30-34 years | Males | NA | 172.9 (-75.72 to 529.78) | 120.46 (30.61 to 250.77) |
| Australia | 30-34 years | Females | 636.65 (-159.31 to 1622.1) | 364.14 (-140.59 to 986.8) | 77.43 (0 to 212.71) |
| Australia | 35-39 years | Both sexes | 278.06 (-70.9 to 690.42) | 230.41 (-120.63 to 683.07) | 39.32 (0.28 to 99.92) |
| Australia | 35-39 years | Males | NA | 115.42 (-41.54 to 386.21) | 66.69 (0 to 162.2) |
| Australia | 35-39 years | Females | 543.24 (-138.5 to 1348.84) | 340.07 (-166.36 to 966.22) | 13.22 (0 to 56.59) |
| Australia | 40-44 years | Both sexes | 235.6 (-58.13 to 552.79) | 211.55 (-106.15 to 618.84) | 14.62 (0 to 52.22) |
| Australia | 40-44 years | Males | NA | 122.28 (-50.68 to 360.57) | 29.6 (0 to 105.69) |
| Australia | 40-44 years | Females | 465.68 (-114.89 to 1092.64) | 298.73 (-158.36 to 856.4) | 0 (0 to 0) |
| Australia | 45-49 years | Both sexes | 210.45 (-43.21 to 501.58) | 192 (-91.32 to 529.63) | 0.79 (0 to 6.14) |
| Australia | 45-49 years | Males | NA | 112.97 (-47.61 to 341.22) | 1.61 (0 to 12.45) |
| Australia | 45-49 years | Females | 415.03 (-85.22 to 989.16) | 268.82 (-131.86 to 721.14) | 0 (0 to 0) |
| Australia | 50-54 years | Both sexes | 164.5 (-20.29 to 446.96) | 179.65 (-90.96 to 547.94) | 0 (0 to 0) |
| Australia | 50-54 years | Males | NA | 95.71 (-42.51 to 322.71) | 0 (0 to 0) |
| Australia | 50-54 years | Females | 321.57 (-39.67 to 873.74) | 259.8 (-136.57 to 738.85) | 0 (0 to 0) |
| Australia | 55-59 years | Both sexes | 131.02 (-14.03 to 382.05) | 162.33 (-81.15 to 499.4) | 0 (0 to 0) |
| Australia | 55-59 years | Males | NA | 95.21 (-39.97 to 310.43) | 0 (0 to 0) |
| Australia | 55-59 years | Females | 256.37 (-27.45 to 747.57) | 226.54 (-117.54 to 672.53) | 0 (0 to 0) |
| Australia | 60-64 years | Both sexes | 84.28 (-7.79 to 251.59) | 133.78 (-59.21 to 433.38) | 0 (0 to 0) |
| Australia | 60-64 years | Males | NA | 75.91 (-33.33 to 258.83) | 0 (0 to 0) |
| Australia | 60-64 years | Females | 163.84 (-15.15 to 489.1) | 188.4 (-83.84 to 594.32) | 0 (0 to 0) |
| Australia | 65-69 years | Both sexes | 51.83 (-4.57 to 175.53) | 120.76 (-63.49 to 375.45) | 0 (0 to 0) |
| Australia | 65-69 years | Males | NA | 75.68 (-31.26 to 254.78) | 0 (0 to 0) |
| Australia | 65-69 years | Females | 99.86 (-8.81 to 338.19) | 162.54 (-90.47 to 488.78) | 0 (0 to 0) |
| Australia | 70-74 years | Both sexes | 39.42 (-4.91 to 118.9) | 72.62 (-32.45 to 245.41) | 0 (0 to 0) |
| Australia | 70-74 years | Males | NA | 44.24 (-16.94 to 159.22) | 0 (0 to 0) |
| Australia | 70-74 years | Females | 75.91 (-9.45 to 228.95) | 98.88 (-46.29 to 328.91) | 0 (0 to 0) |
| Australia | 75-79 years | Both sexes | 25.51 (-3.37 to 87.43) | 44.65 (-16.3 to 153.27) | 0 (0 to 0) |
| Australia | 75-79 years | Males | NA | 22.62 (-7.21 to 90.49) | 0 (0 to 0) |
| Australia | 75-79 years | Females | 48.99 (-6.47 to 167.91) | 64.92 (-25.6 to 215.84) | 0 (0 to 0) |
| Australia | 80-84 years | Both sexes | 19.25 (-2.63 to 60.59) | 37.9 (-14.04 to 126.4) | 0 (0 to 0) |
| Australia | 80-84 years | Males | NA | 14.97 (-4.31 to 63.96) | 0 (0 to 0) |
| Australia | 80-84 years | Females | 35.6 (-4.87 to 112.06) | 57.37 (-22.97 to 187.46) | 0 (0 to 0) |
| Australia | 85-89 years | Both sexes | 18.08 (-2.62 to 60.7) | 34.9 (-13.57 to 114.95) | 0 (0 to 0) |
| Australia | 85-89 years | Males | NA | 13.21 (-3.82 to 56.92) | 0 (0 to 0) |
| Australia | 85-89 years | Females | 31.37 (-4.54 to 105.34) | 50.85 (-20.96 to 161.76) | 0 (0 to 0) |
| Australia | 90-94 years | Both sexes | 18.82 (-2.7 to 63.58) | 20.77 (-6.95 to 87.62) | 0 (0 to 0) |
| Australia | 90-94 years | Males | NA | 12.15 (-3.8 to 54.32) | 0 (0 to 0) |
| Australia | 90-94 years | Females | 29.77 (-4.27 to 100.56) | 25.78 (-8.81 to 103.33) | 0 (0 to 0) |
| Australia | 95+ years | Both sexes | 20.66 (-2.7 to 73.64) | 21.15 (-7.43 to 80.04) | 0 (0 to 0) |
| Australia | 95+ years | Males | NA | 11.88 (-3.8 to 48.55) | 0 (0 to 0) |
| Australia | 95+ years | Females | 29.81 (-3.9 to 106.27) | 25.26 (-9.08 to 94.29) | 0 (0 to 0) |
| Austria | <5 years | Both sexes | NA | NA | 0 (0 to 0) |
| Austria | <5 years | Males | NA | NA | 0 (0 to 0) |
| Austria | <5 years | Females | NA | NA | 0 (0 to 0) |
| Austria | 5-9 years | Both sexes | NA | NA | 23.95 (6.32 to 63.19) |
| Austria | 5-9 years | Males | NA | NA | 32.48 (9.02 to 83.84) |
| Austria | 5-9 years | Females | NA | NA | 14.89 (3.22 to 41.81) |
| Austria | 10-14 years | Both sexes | NA | NA | 186.3 (71.52 to 388.77) |
| Austria | 10-14 years | Males | NA | NA | 188 (69.9 to 379.49) |
| Austria | 10-14 years | Females | NA | NA | 184.49 (61.56 to 401.89) |
| Austria | 15-19 years | Both sexes | 62.27 (-4.27 to 247.89) | 154.7 (-50.87 to 666.71) | 243.94 (88.22 to 515.71) |
| Austria | 15-19 years | Males | NA | 95.69 (-30.5 to 417.48) | 242.57 (89 to 496.89) |
| Austria | 15-19 years | Females | 128.31 (-8.79 to 510.8) | 217.28 (-70.54 to 935.2) | 245.4 (73.79 to 560.24) |
| Austria | 20-24 years | Both sexes | 68.05 (-9.17 to 263.39) | 101.53 (-30.76 to 321.48) | 149.29 (51.02 to 335.59) |
| Austria | 20-24 years | Males | NA | 74.7 (-21.98 to 281.36) | 160.73 (58.84 to 314.05) |
| Austria | 20-24 years | Females | 140.82 (-18.97 to 545.08) | 130.22 (-36.68 to 435.69) | 137.04 (37.58 to 353.72) |
| Austria | 25-29 years | Both sexes | 93.37 (-8.7 to 289.81) | 80.6 (-27.05 to 249.21) | 88.25 (25.81 to 195.83) |
| Austria | 25-29 years | Males | NA | 60.41 (-18.42 to 243.08) | 103.13 (35.34 to 206.7) |
| Austria | 25-29 years | Females | 192.45 (-17.94 to 597.31) | 102.03 (-33.67 to 337.06) | 72.46 (10.8 to 178.29) |
| Austria | 30-34 years | Both sexes | 92.61 (-10.43 to 265.81) | 70.25 (-22.88 to 240.93) | 52.33 (10.27 to 117.41) |
| Austria | 30-34 years | Males | NA | 56.38 (-18.21 to 237.91) | 67.83 (15.85 to 139.86) |
| Austria | 30-34 years | Females | 189.91 (-21.38 to 545.1) | 84.82 (-24.64 to 284.8) | 36.04 (0 to 100.85) |
| Austria | 35-39 years | Both sexes | 84.69 (-9.35 to 246.2) | 66.39 (-22.88 to 226.15) | 23.16 (0.04 to 56.1) |
| Austria | 35-39 years | Males | NA | 51.19 (-18.39 to 208.95) | 40.44 (0 to 99.88) |
| Austria | 35-39 years | Females | 171.43 (-18.93 to 498.38) | 81.96 (-28.83 to 269.11) | 5.46 (0 to 23.64) |
| Austria | 40-44 years | Both sexes | 77.82 (-8.09 to 215.32) | 61.46 (-22.14 to 184.76) | 8.35 (0 to 31.83) |
| Austria | 40-44 years | Males | NA | 48.64 (-16.74 to 169.44) | 16.63 (0 to 63.36) |
| Austria | 40-44 years | Females | 156.36 (-16.25 to 432.65) | 74.4 (-26.9 to 240.71) | 0 (0 to 0) |
| Austria | 45-49 years | Both sexes | 73.14 (-7.73 to 206.66) | 56.71 (-21.63 to 180.77) | 0.44 (0 to 3.34) |
| Austria | 45-49 years | Males | NA | 45.26 (-16.77 to 160.09) | 0.88 (0 to 6.73) |
| Austria | 45-49 years | Females | 145.04 (-15.33 to 409.83) | 67.96 (-25.13 to 211.05) | 0 (0 to 0) |
| Austria | 50-54 years | Both sexes | 57.02 (-5.85 to 192.8) | 56.26 (-22.4 to 197.09) | 0 (0 to 0) |
| Austria | 50-54 years | Males | NA | 44.72 (-17.49 to 180.55) | 0 (0 to 0) |
| Austria | 50-54 years | Females | 113.16 (-11.61 to 382.64) | 67.62 (-25.17 to 211.87) | 0 (0 to 0) |
| Austria | 55-59 years | Both sexes | 48.8 (-4.6 to 160.62) | 50.19 (-19.69 to 177.28) | 0 (0 to 0) |
| Austria | 55-59 years | Males | NA | 42.19 (-17.2 to 174.04) | 0 (0 to 0) |
| Austria | 55-59 years | Females | 97.38 (-9.18 to 320.48) | 58.14 (-20.68 to 199.15) | 0 (0 to 0) |
| Austria | 60-64 years | Both sexes | 35.21 (-4.49 to 133.78) | 48.49 (-20.46 to 177.75) | 0 (0 to 0) |
| Austria | 60-64 years | Males | NA | 41.19 (-17.71 to 172.38) | 0 (0 to 0) |
| Austria | 60-64 years | Females | 69.42 (-8.85 to 263.74) | 55.57 (-19.35 to 209.96) | 0 (0 to 0) |
| Austria | 65-69 years | Both sexes | 26.22 (-2.81 to 93.65) | 45.02 (-16.57 to 163.87) | 0 (0 to 0) |
| Austria | 65-69 years | Males | NA | 39.81 (-15.38 to 169.48) | 0 (0 to 0) |
| Austria | 65-69 years | Females | 49.96 (-5.35 to 178.47) | 49.73 (-13.87 to 181.89) | 0 (0 to 0) |
| Austria | 70-74 years | Both sexes | 24.59 (-2.35 to 95.59) | 36.43 (-11.2 to 138.61) | 0 (0 to 0) |
| Austria | 70-74 years | Males | NA | 33.72 (-9.91 to 152.72) | 0 (0 to 0) |
| Austria | 70-74 years | Females | 45.49 (-4.36 to 176.86) | 38.73 (-9.14 to 158.37) | 0 (0 to 0) |
| Austria | 75-79 years | Both sexes | 21.94 (-1.89 to 87.9) | 36.9 (-10.81 to 151.16) | 0 (0 to 0) |
| Austria | 75-79 years | Males | NA | 29.13 (-8.43 to 113.17) | 0 (0 to 0) |
| Austria | 75-79 years | Females | 39.12 (-3.37 to 156.75) | 42.99 (-12.73 to 177.96) | 0 (0 to 0) |
| Austria | 80-84 years | Both sexes | 15.08 (-1.27 to 67) | 26.38 (-7.53 to 111.56) | 0 (0 to 0) |
| Austria | 80-84 years | Males | NA | 20.91 (-5.91 to 87.99) | 0 (0 to 0) |
| Austria | 80-84 years | Females | 25.87 (-2.18 to 114.89) | 30.29 (-8.8 to 128.28) | 0 (0 to 0) |
| Austria | 85-89 years | Both sexes | 15.07 (-1.29 to 65.73) | 23.14 (-6.42 to 98.44) | 0 (0 to 0) |
| Austria | 85-89 years | Males | NA | 18.29 (-5.55 to 78.35) | 0 (0 to 0) |
| Austria | 85-89 years | Females | 23.96 (-2.05 to 104.49) | 26 (-6.92 to 110.15) | 0 (0 to 0) |
| Austria | 90-94 years | Both sexes | 15.7 (-1.39 to 73.89) | 20.74 (-6.07 to 87.83) | 0 (0 to 0) |
| Austria | 90-94 years | Males | NA | 16.46 (-4.96 to 74.33) | 0 (0 to 0) |
| Austria | 90-94 years | Females | 23.07 (-2.04 to 108.55) | 22.74 (-6.64 to 94.58) | 0 (0 to 0) |
| Austria | 95+ years | Both sexes | 17.61 (-1.51 to 76.86) | 20.01 (-6.89 to 84.13) | 0 (0 to 0) |
| Austria | 95+ years | Males | NA | 15.74 (-5.36 to 64.24) | 0 (0 to 0) |
| Austria | 95+ years | Females | 22.78 (-1.96 to 99.43) | 21.26 (-7.33 to 90.19) | 0 (0 to 0) |
| Azerbaijan | <5 years | Both sexes | NA | NA | 0 (0 to 0) |
| Azerbaijan | <5 years | Males | NA | NA | 0 (0 to 0) |
| Azerbaijan | <5 years | Females | NA | NA | 0 (0 to 0) |
| Azerbaijan | 5-9 years | Both sexes | NA | NA | 2.34 (0.54 to 6.76) |
| Azerbaijan | 5-9 years | Males | NA | NA | 3.01 (0.7 to 8.67) |
| Azerbaijan | 5-9 years | Females | NA | NA | 1.59 (0.32 to 4.91) |
| Azerbaijan | 10-14 years | Both sexes | NA | NA | 32.4 (11.01 to 80.14) |
| Azerbaijan | 10-14 years | Males | NA | NA | 40.44 (13.6 to 101.31) |
| Azerbaijan | 10-14 years | Females | NA | NA | 23.16 (6.91 to 55.97) |
| Azerbaijan | 15-19 years | Both sexes | 10.81 (-1.13 to 47.36) | 38.14 (-12.09 to 155.68) | 30.69 (11.01 to 70.63) |
| Azerbaijan | 15-19 years | Males | NA | 29.29 (-8.69 to 122.97) | 38.64 (14.12 to 89.86) |
| Azerbaijan | 15-19 years | Females | 23.1 (-2.41 to 101.18) | 48.2 (-16.05 to 197.37) | 21.65 (6.37 to 54.7) |
| Azerbaijan | 20-24 years | Both sexes | 27.13 (-2.87 to 93.79) | 29.34 (-8.02 to 120.27) | 20.19 (6.65 to 46.79) |
| Azerbaijan | 20-24 years | Males | NA | 20.15 (-5.71 to 84.67) | 25.98 (8.53 to 58.59) |
| Azerbaijan | 20-24 years | Females | 55.72 (-5.89 to 192.66) | 39.03 (-10.63 to 153.48) | 14.08 (3.7 to 35.47) |
| Azerbaijan | 25-29 years | Both sexes | 38.93 (-6.8 to 123.06) | 27.47 (-6.78 to 107.03) | 13.7 (3.76 to 33.68) |
| Azerbaijan | 25-29 years | Males | NA | 17.94 (-4.55 to 71.51) | 18.47 (5.43 to 42.63) |
| Azerbaijan | 25-29 years | Females | 76.76 (-13.41 to 242.65) | 36.72 (-9.65 to 137.19) | 9.07 (1.3 to 23.94) |
| Azerbaijan | 30-34 years | Both sexes | 36.47 (-6.09 to 114.48) | 30.04 (-7.75 to 115.84) | 9.42 (1.64 to 24.41) |
| Azerbaijan | 30-34 years | Males | NA | 24.14 (-6.6 to 97.23) | 13.48 (2.42 to 35.26) |
| Azerbaijan | 30-34 years | Females | 71.08 (-11.86 to 223.1) | 35.64 (-9.08 to 144.5) | 5.56 (0 to 16.44) |
| Azerbaijan | 35-39 years | Both sexes | 36.06 (-6 to 115.63) | 30.02 (-7.67 to 113.66) | 5.06 (0.04 to 14.18) |
| Azerbaijan | 35-39 years | Males | NA | 21.22 (-6.06 to 78.36) | 8.87 (0.08 to 22.89) |
| Azerbaijan | 35-39 years | Females | 71.21 (-11.85 to 228.37) | 38.6 (-9.49 to 145.19) | 1.34 (0 to 5.57) |
| Azerbaijan | 40-44 years | Both sexes | 39.63 (-6.6 to 113.04) | 32.48 (-9.82 to 110.9) | 2.21 (0 to 8.28) |
| Azerbaijan | 40-44 years | Males | NA | 27.16 (-8.94 to 91.57) | 4.36 (0 to 16.37) |
| Azerbaijan | 40-44 years | Females | 80.18 (-13.36 to 228.72) | 37.93 (-10.75 to 131.05) | 0 (0 to 0) |
| Azerbaijan | 45-49 years | Both sexes | 37.27 (-5.56 to 112.31) | 31.06 (-9.49 to 110.17) | 0.19 (0 to 1.39) |
| Azerbaijan | 45-49 years | Males | NA | 24.84 (-7.93 to 93.34) | 0.38 (0 to 2.79) |
| Azerbaijan | 45-49 years | Females | 74.21 (-11.06 to 223.66) | 37.23 (-11.34 to 133.94) | 0 (0 to 0) |
| Azerbaijan | 50-54 years | Both sexes | 41.29 (-4.48 to 139.27) | 32.04 (-9.99 to 126.81) | 0 (0 to 0) |
| Azerbaijan | 50-54 years | Males | NA | 23.69 (-7.87 to 95.65) | 0 (0 to 0) |
| Azerbaijan | 50-54 years | Females | 79.93 (-8.68 to 269.61) | 39.85 (-12.22 to 154.96) | 0 (0 to 0) |
| Azerbaijan | 55-59 years | Both sexes | 38.51 (-4.32 to 132.61) | 32.85 (-10.4 to 127.67) | 0 (0 to 0) |
| Azerbaijan | 55-59 years | Males | NA | 29.3 (-10.02 to 110.34) | 0 (0 to 0) |
| Azerbaijan | 55-59 years | Females | 72.38 (-8.13 to 249.24) | 35.98 (-10.32 to 145.29) | 0 (0 to 0) |
| Azerbaijan | 60-64 years | Both sexes | 30.34 (-3.09 to 114.28) | 31.96 (-12.14 to 142.96) | 0 (0 to 0) |
| Azerbaijan | 60-64 years | Males | NA | 24.05 (-8.32 to 108.04) | 0 (0 to 0) |
| Azerbaijan | 60-64 years | Females | 56.62 (-5.77 to 213.27) | 38.82 (-15.45 to 171.26) | 0 (0 to 0) |
| Azerbaijan | 65-69 years | Both sexes | 23.24 (-2.38 to 93.44) | 30.65 (-11.05 to 133.03) | 0 (0 to 0) |
| Azerbaijan | 65-69 years | Males | NA | 23.43 (-7.73 to 99.66) | 0 (0 to 0) |
| Azerbaijan | 65-69 years | Females | 42.58 (-4.37 to 171.16) | 36.66 (-13.41 to 159.74) | 0 (0 to 0) |
| Azerbaijan | 70-74 years | Both sexes | 21.56 (-2.13 to 95.73) | 25.85 (-7.45 to 119.74) | 0 (0 to 0) |
| Azerbaijan | 70-74 years | Males | NA | 19.44 (-4.94 to 89.99) | 0 (0 to 0) |
| Azerbaijan | 70-74 years | Females | 39 (-3.86 to 173.21) | 31.04 (-8.91 to 146.33) | 0 (0 to 0) |
| Azerbaijan | 75-79 years | Both sexes | 17.17 (-1.84 to 75.83) | 22.95 (-6.37 to 94.33) | 0 (0 to 0) |
| Azerbaijan | 75-79 years | Males | NA | 17.46 (-4.61 to 73.59) | 0 (0 to 0) |
| Azerbaijan | 75-79 years | Females | 29.74 (-3.19 to 131.33) | 26.97 (-7.54 to 109.6) | 0 (0 to 0) |
| Azerbaijan | 80-84 years | Both sexes | 12.36 (-1.5 to 56.35) | 16.52 (-4.38 to 71.65) | 0 (0 to 0) |
| Azerbaijan | 80-84 years | Males | NA | 12.5 (-3.35 to 53.24) | 0 (0 to 0) |
| Azerbaijan | 80-84 years | Females | 20.02 (-2.43 to 91.24) | 19.01 (-5.13 to 82.77) | 0 (0 to 0) |
| Azerbaijan | 85-89 years | Both sexes | 12.25 (-1.35 to 55.49) | 14.88 (-4.1 to 63.53) | 0 (0 to 0) |
| Azerbaijan | 85-89 years | Males | NA | 11.25 (-3.08 to 48.81) | 0 (0 to 0) |
| Azerbaijan | 85-89 years | Females | 19.55 (-2.15 to 88.59) | 17.05 (-4.64 to 73.07) | 0 (0 to 0) |
| Azerbaijan | 90-94 years | Both sexes | 11.46 (-1.23 to 53.14) | 13.37 (-4.04 to 60.28) | 0 (0 to 0) |
| Azerbaijan | 90-94 years | Males | NA | 10.37 (-3.15 to 45.81) | 0 (0 to 0) |
| Azerbaijan | 90-94 years | Females | 19.36 (-2.08 to 89.77) | 15.45 (-4.58 to 69.63) | 0 (0 to 0) |
| Azerbaijan | 95+ years | Both sexes | 13.35 (-1.34 to 56.61) | 13.52 (-4.39 to 55.67) | 0 (0 to 0) |
| Azerbaijan | 95+ years | Males | NA | 10.28 (-3.31 to 43.56) | 0 (0 to 0) |
| Azerbaijan | 95+ years | Females | 19.59 (-1.97 to 83.07) | 15.04 (-4.89 to 60.75) | 0 (0 to 0) |
| Bahamas | <5 years | Both sexes | NA | NA | 0 (0 to 0) |
| Bahamas | <5 years | Males | NA | NA | 0 (0 to 0) |
| Bahamas | <5 years | Females | NA | NA | 0 (0 to 0) |
| Bahamas | 5-9 years | Both sexes | NA | NA | 5.93 (1.53 to 16.66) |
| Bahamas | 5-9 years | Males | NA | NA | 7.85 (1.97 to 21.13) |
| Bahamas | 5-9 years | Females | NA | NA | 3.97 (0.9 to 11.6) |
| Bahamas | 10-14 years | Both sexes | NA | NA | 84.19 (30.02 to 175.09) |
| Bahamas | 10-14 years | Males | NA | NA | 100.81 (39.25 to 200.72) |
| Bahamas | 10-14 years | Females | NA | NA | 67.49 (22.27 to 154.82) |
| Bahamas | 15-19 years | Both sexes | 44.51 (-3.95 to 168.09) | 72.25 (-25.89 to 270.23) | 87.99 (34.79 to 186.72) |
| Bahamas | 15-19 years | Males | NA | 48.04 (-16.41 to 180.16) | 90.77 (36.72 to 186.32) |
| Bahamas | 15-19 years | Females | 88.66 (-7.86 to 334.78) | 96.25 (-33.26 to 355.22) | 85.24 (29.04 to 191.78) |
| Bahamas | 20-24 years | Both sexes | 117.43 (-17.06 to 385.77) | 88.35 (-24.41 to 351.94) | 87.71 (31.9 to 191.23) |
| Bahamas | 20-24 years | Males | NA | 55.39 (-15.5 to 215.21) | 89.65 (35.44 to 192.79) |
| Bahamas | 20-24 years | Females | 231.84 (-33.68 to 761.61) | 120.46 (-34 to 466.67) | 85.83 (25.44 to 206.65) |
| Bahamas | 25-29 years | Both sexes | 168.53 (-32.76 to 527.14) | 95.76 (-28.92 to 366.27) | 72.42 (20.74 to 161.4) |
| Bahamas | 25-29 years | Males | NA | 59.08 (-16.06 to 233.78) | 77.14 (25.47 to 170.19) |
| Bahamas | 25-29 years | Females | 329.48 (-64.04 to 1030.57) | 130.79 (-41.2 to 500.22) | 67.92 (10.22 to 171.46) |
| Bahamas | 30-34 years | Both sexes | 185.12 (-35.95 to 564.52) | 103.84 (-33.96 to 438.38) | 54.36 (10.32 to 127.59) |
| Bahamas | 30-34 years | Males | NA | 63.08 (-19.63 to 244.95) | 62.28 (13.78 to 136.88) |
| Bahamas | 30-34 years | Females | 357.23 (-69.38 to 1089.39) | 141.74 (-47.32 to 608.83) | 46.99 (0 to 128.58) |
| Bahamas | 35-39 years | Both sexes | 183.48 (-39.18 to 509.37) | 101.38 (-36.46 to 419.09) | 21.78 (0.1 to 60.5) |
| Bahamas | 35-39 years | Males | NA | 61.95 (-20.57 to 259.15) | 35.73 (0.21 to 88.43) |
| Bahamas | 35-39 years | Females | 350.89 (-74.93 to 974.13) | 137.36 (-50.99 to 561.72) | 9.05 (0 to 36.99) |
| Bahamas | 40-44 years | Both sexes | 175.09 (-37.32 to 466.41) | 98.3 (-36.48 to 356.65) | 8.85 (0 to 32.45) |
| Bahamas | 40-44 years | Males | NA | 60.78 (-21.13 to 224.81) | 18.61 (0 to 68.21) |
| Bahamas | 40-44 years | Females | 334.01 (-71.19 to 889.72) | 132.35 (-50.42 to 471.63) | 0 (0 to 0) |
| Bahamas | 45-49 years | Both sexes | 168.99 (-32.85 to 461.57) | 93.05 (-35.15 to 326.82) | 0.59 (0 to 4.13) |
| Bahamas | 45-49 years | Males | NA | 58.07 (-21.32 to 206.03) | 1.24 (0 to 8.64) |
| Bahamas | 45-49 years | Females | 323.38 (-62.87 to 883.25) | 125.01 (-47.57 to 441.62) | 0 (0 to 0) |
| Bahamas | 50-54 years | Both sexes | 144.37 (-21.31 to 434.74) | 89.7 (-36.19 to 358.49) | 0 (0 to 0) |
| Bahamas | 50-54 years | Males | NA | 57.46 (-20.22 to 223.19) | 0 (0 to 0) |
| Bahamas | 50-54 years | Females | 277.45 (-40.95 to 835.47) | 119.42 (-50.07 to 485.98) | 0 (0 to 0) |
| Bahamas | 55-59 years | Both sexes | 130.47 (-19.67 to 393.27) | 85.11 (-36.85 to 349.81) | 0 (0 to 0) |
| Bahamas | 55-59 years | Males | NA | 55.22 (-22.38 to 218.91) | 0 (0 to 0) |
| Bahamas | 55-59 years | Females | 247.84 (-37.37 to 747.06) | 112.01 (-48.68 to 467.65) | 0 (0 to 0) |
| Bahamas | 60-64 years | Both sexes | 100.96 (-14.97 to 310.45) | 80.94 (-35.72 to 331.26) | 0 (0 to 0) |
| Bahamas | 60-64 years | Males | NA | 53.65 (-22.9 to 213.89) | 0 (0 to 0) |
| Bahamas | 60-64 years | Females | 188.77 (-27.98 to 580.47) | 104.67 (-46.88 to 425.65) | 0 (0 to 0) |
| Bahamas | 65-69 years | Both sexes | 77.63 (-9.01 to 259.81) | 74.96 (-30.28 to 318.42) | 0 (0 to 0) |
| Bahamas | 65-69 years | Males | NA | 50.8 (-19.7 to 202.26) | 0 (0 to 0) |
| Bahamas | 65-69 years | Females | 141.81 (-16.46 to 474.61) | 94.93 (-39.03 to 393.6) | 0 (0 to 0) |
| Bahamas | 70-74 years | Both sexes | 68.86 (-7.14 to 247.37) | 61.53 (-18.91 to 277.4) | 0 (0 to 0) |
| Bahamas | 70-74 years | Males | NA | 42.41 (-12.78 to 187.83) | 0 (0 to 0) |
| Bahamas | 70-74 years | Females | 123.27 (-12.79 to 442.8) | 76.64 (-23.75 to 348.16) | 0 (0 to 0) |
| Bahamas | 75-79 years | Both sexes | 54.65 (-5.3 to 196.72) | 53.53 (-15.48 to 215.83) | 0 (0 to 0) |
| Bahamas | 75-79 years | Males | NA | 37.06 (-10.15 to 153.15) | 0 (0 to 0) |
| Bahamas | 75-79 years | Females | 94.36 (-9.14 to 339.64) | 65.5 (-19.25 to 263.44) | 0 (0 to 0) |
| Bahamas | 80-84 years | Both sexes | 37.79 (-3.52 to 150.01) | 38.18 (-11.08 to 160.93) | 0 (0 to 0) |
| Bahamas | 80-84 years | Males | NA | 26.3 (-7.11 to 107.6) | 0 (0 to 0) |
| Bahamas | 80-84 years | Females | 64.33 (-5.99 to 255.36) | 46.53 (-13.76 to 195.59) | 0 (0 to 0) |
| Bahamas | 85-89 years | Both sexes | 37.29 (-3.5 to 147.97) | 33.8 (-9.98 to 146.49) | 0 (0 to 0) |
| Bahamas | 85-89 years | Males | NA | 23.21 (-6.76 to 97.04) | 0 (0 to 0) |
| Bahamas | 85-89 years | Females | 61.53 (-5.78 to 244.2) | 40.69 (-12.22 to 176.2) | 0 (0 to 0) |
| Bahamas | 90-94 years | Both sexes | 40.91 (-3.96 to 158.55) | 31.9 (-9.83 to 141.14) | 0 (0 to 0) |
| Bahamas | 90-94 years | Males | NA | 21.19 (-6.4 to 92.15) | 0 (0 to 0) |
| Bahamas | 90-94 years | Females | 60.51 (-5.86 to 234.52) | 37.03 (-11.64 to 161.9) | 0 (0 to 0) |
| Bahamas | 95+ years | Both sexes | 51.96 (-5.16 to 212.36) | 33.45 (-11.54 to 138.15) | 0 (0 to 0) |
| Bahamas | 95+ years | Males | NA | 20.56 (-6.96 to 85.18) | 0 (0 to 0) |
| Bahamas | 95+ years | Females | 61.41 (-6.1 to 250.98) | 35.79 (-12.35 to 148.4) | 0 (0 to 0) |
| Bahrain | <5 years | Both sexes | NA | NA | 0 (0 to 0) |
| Bahrain | <5 years | Males | NA | NA | 0 (0 to 0) |
| Bahrain | <5 years | Females | NA | NA | 0 (0 to 0) |
| Bahrain | 5-9 years | Both sexes | NA | NA | 4.73 (1.38 to 12.67) |
| Bahrain | 5-9 years | Males | NA | NA | 7.49 (2.18 to 19.89) |
| Bahrain | 5-9 years | Females | NA | NA | 1.84 (0.41 to 5.42) |
| Bahrain | 10-14 years | Both sexes | NA | NA | 96.77 (41.4 to 189.15) |
| Bahrain | 10-14 years | Males | NA | NA | 144.43 (61.66 to 263.98) |
| Bahrain | 10-14 years | Females | NA | NA | 46.37 (16.73 to 104.04) |
| Bahrain | 15-19 years | Both sexes | 38.39 (-3.32 to 151.03) | 81.7 (-28.33 to 346.38) | 106.49 (44.04 to 203.73) |
| Bahrain | 15-19 years | Males | NA | 74.39 (-25.26 to 288.64) | 146.89 (61.81 to 276.33) |
| Bahrain | 15-19 years | Females | 84.54 (-7.32 to 332.59) | 90.48 (-31.73 to 406.74) | 57.91 (19.72 to 133.1) |
| Bahrain | 20-24 years | Both sexes | 61.52 (-8.38 to 196.8) | 72.98 (-25.86 to 281.41) | 82.12 (30.9 to 183.22) |
| Bahrain | 20-24 years | Males | NA | 63.48 (-21.78 to 246.74) | 106.78 (42.17 to 225.08) |
| Bahrain | 20-24 years | Females | 152.87 (-20.83 to 489.06) | 87.07 (-31.92 to 358.36) | 45.51 (12.08 to 113.54) |
| Bahrain | 25-29 years | Both sexes | 67.46 (-12.18 to 193.08) | 62.37 (-20.39 to 241.05) | 59.03 (18.99 to 119) |
| Bahrain | 25-29 years | Males | NA | 54.04 (-16.08 to 198.89) | 74.35 (25.88 to 146.81) |
| Bahrain | 25-29 years | Females | 195.52 (-35.3 to 559.61) | 78.18 (-28.58 to 314.51) | 29.95 (5.18 to 74.89) |
| Bahrain | 30-34 years | Both sexes | 60.53 (-11.42 to 177.6) | 60.4 (-22.65 to 236.29) | 38.84 (7.93 to 94.67) |
| Bahrain | 30-34 years | Males | NA | 53.38 (-18.7 to 204.23) | 48.82 (10.28 to 112.49) |
| Bahrain | 30-34 years | Females | 199.04 (-37.54 to 584) | 76.46 (-30.84 to 316.34) | 16 (0 to 49.45) |
| Bahrain | 35-39 years | Both sexes | 61.89 (-11.66 to 171.01) | 58.45 (-19.9 to 224.31) | 23.72 (0.17 to 63.9) |
| Bahrain | 35-39 years | Males | NA | 52.49 (-17.06 to 193.66) | 32.95 (0.24 to 85.11) |
| Bahrain | 35-39 years | Females | 195.67 (-36.86 to 540.64) | 71.33 (-26.56 to 300.78) | 3.76 (0 to 15.14) |
| Bahrain | 40-44 years | Both sexes | 60.45 (-12.09 to 155.71) | 59.64 (-20.71 to 217.41) | 12.99 (0 to 43.47) |
| Bahrain | 40-44 years | Males | NA | 54.75 (-18.03 to 192.39) | 18.93 (0 to 63.32) |
| Bahrain | 40-44 years | Females | 192.85 (-38.58 to 496.73) | 70.34 (-26.57 to 272.05) | 0 (0 to 0) |
| Bahrain | 45-49 years | Both sexes | 52.82 (-9.73 to 144.55) | 59.67 (-21.58 to 222.92) | 2 (0 to 11.19) |
| Bahrain | 45-49 years | Males | NA | 56.64 (-20.83 to 198.74) | 2.72 (0 to 15.25) |
| Bahrain | 45-49 years | Females | 198.52 (-36.55 to 543.25) | 68.04 (-24.6 to 266.44) | 0 (0 to 0) |
| Bahrain | 50-54 years | Both sexes | 64.36 (-9.68 to 189.16) | 61.78 (-23.02 to 246.53) | 0 (0 to 0) |
| Bahrain | 50-54 years | Males | NA | 58.93 (-22.86 to 225.38) | 0 (0 to 0) |
| Bahrain | 50-54 years | Females | 181.04 (-27.22 to 532.11) | 66.95 (-25.39 to 271.77) | 0 (0 to 0) |
| Bahrain | 55-59 years | Both sexes | 63.64 (-10.16 to 184.19) | 61.09 (-25.31 to 247.66) | 0 (0 to 0) |
| Bahrain | 55-59 years | Males | NA | 59.26 (-25.14 to 227.95) | 0 (0 to 0) |
| Bahrain | 55-59 years | Females | 169.1 (-27 to 489.45) | 64.12 (-25.58 to 265.47) | 0 (0 to 0) |
| Bahrain | 60-64 years | Both sexes | 50.73 (-7.81 to 161.22) | 59.77 (-22.35 to 255.92) | 0 (0 to 0) |
| Bahrain | 60-64 years | Males | NA | 58.6 (-21.29 to 239.76) | 0 (0 to 0) |
| Bahrain | 60-64 years | Females | 136.89 (-21.08 to 435.02) | 61.75 (-24.29 to 278.04) | 0 (0 to 0) |
| Bahrain | 65-69 years | Both sexes | 43.47 (-4.85 to 150.85) | 55.95 (-20.55 to 245.97) | 0 (0 to 0) |
| Bahrain | 65-69 years | Males | NA | 55.81 (-21.93 to 234.24) | 0 (0 to 0) |
| Bahrain | 65-69 years | Females | 105.28 (-11.75 to 365.36) | 56.15 (-18.59 to 261.44) | 0 (0 to 0) |
| Bahrain | 70-74 years | Both sexes | 40.16 (-4.03 to 145.74) | 46.5 (-14.39 to 211.1) | 0 (0 to 0) |
| Bahrain | 70-74 years | Males | NA | 46.92 (-14.23 to 216.74) | 0 (0 to 0) |
| Bahrain | 70-74 years | Females | 94.62 (-9.49 to 343.36) | 45.92 (-14.21 to 197.51) | 0 (0 to 0) |
| Bahrain | 75-79 years | Both sexes | 38.23 (-3.81 to 135.53) | 40.13 (-12.02 to 167.55) | 0 (0 to 0) |
| Bahrain | 75-79 years | Males | NA | 41.13 (-12.49 to 166.96) | 0 (0 to 0) |
| Bahrain | 75-79 years | Females | 74.13 (-7.4 to 262.79) | 39.2 (-11.58 to 166.55) | 0 (0 to 0) |
| Bahrain | 80-84 years | Both sexes | 30.32 (-2.9 to 119.4) | 28.69 (-8.07 to 131) | 0 (0 to 0) |
| Bahrain | 80-84 years | Males | NA | 30.07 (-8.65 to 126.72) | 0 (0 to 0) |
| Bahrain | 80-84 years | Females | 51.78 (-4.95 to 203.91) | 27.71 (-7.56 to 134.53) | 0 (0 to 0) |
| Bahrain | 85-89 years | Both sexes | 30.25 (-2.85 to 119.28) | 25.7 (-7.65 to 114.33) | 0 (0 to 0) |
| Bahrain | 85-89 years | Males | NA | 27.22 (-8.17 to 109.83) | 0 (0 to 0) |
| Bahrain | 85-89 years | Females | 50.79 (-4.79 to 200.32) | 24.66 (-7.41 to 113.85) | 0 (0 to 0) |
| Bahrain | 90-94 years | Both sexes | 32.52 (-2.99 to 133.6) | 23.5 (-7.62 to 114.87) | 0 (0 to 0) |
| Bahrain | 90-94 years | Males | NA | 25.33 (-7.96 to 116.88) | 0 (0 to 0) |
| Bahrain | 90-94 years | Females | 50.74 (-4.66 to 208.45) | 22.48 (-7.41 to 113.83) | 0 (0 to 0) |
| Bahrain | 95+ years | Both sexes | 40.4 (-3.9 to 164.61) | 22.85 (-7.76 to 101.85) | 0 (0 to 0) |
| Bahrain | 95+ years | Males | NA | 25.32 (-8.8 to 106.33) | 0 (0 to 0) |
| Bahrain | 95+ years | Females | 52.44 (-5.06 to 213.67) | 22.11 (-7.45 to 100.43) | 0 (0 to 0) |
| Bangladesh | <5 years | Both sexes | NA | NA | 0 (0 to 0) |
| Bangladesh | <5 years | Males | NA | NA | 0 (0 to 0) |
| Bangladesh | <5 years | Females | NA | NA | 0 (0 to 0) |
| Bangladesh | 5-9 years | Both sexes | NA | NA | 1.16 (0.24 to 3.34) |
| Bangladesh | 5-9 years | Males | NA | NA | 1.61 (0.34 to 4.6) |
| Bangladesh | 5-9 years | Females | NA | NA | 0.67 (0.12 to 2.13) |
| Bangladesh | 10-14 years | Both sexes | NA | NA | 22.37 (6.68 to 54.2) |
| Bangladesh | 10-14 years | Males | NA | NA | 31.14 (9.59 to 75.51) |
| Bangladesh | 10-14 years | Females | NA | NA | 13.1 (3.65 to 35.32) |
| Bangladesh | 15-19 years | Both sexes | 84.94 (-15.47 to 254.09) | 48.38 (-18.11 to 180.75) | 18.65 (6.57 to 41.69) |
| Bangladesh | 15-19 years | Males | NA | 60.22 (-24.02 to 222.03) | 26.44 (9.4 to 56.98) |
| Bangladesh | 15-19 years | Females | 169.14 (-30.81 to 505.98) | 36.64 (-12.87 to 144.63) | 10.92 (3.18 to 26.52) |
| Bangladesh | 20-24 years | Both sexes | 126.39 (-36.95 to 311.65) | 39.08 (-15.27 to 132.56) | 14.12 (4.74 to 32.07) |
| Bangladesh | 20-24 years | Males | NA | 49.34 (-21.51 to 166.85) | 20.86 (7.36 to 47.33) |
| Bangladesh | 20-24 years | Females | 231.44 (-67.66 to 570.7) | 30.55 (-9.3 to 108.93) | 8.52 (2.3 to 22.04) |
| Bangladesh | 25-29 years | Both sexes | 137.82 (-47.34 to 324.74) | 41.99 (-15.71 to 148.9) | 10.43 (2.67 to 24.62) |
| Bangladesh | 25-29 years | Males | NA | 51.2 (-21.69 to 182.72) | 16 (4.58 to 37.1) |
| Bangladesh | 25-29 years | Females | 247.75 (-85.1 to 583.75) | 34.65 (-11.65 to 122.45) | 5.98 (0.85 to 15.85) |
| Bangladesh | 30-34 years | Both sexes | 142.17 (-43.83 to 331.18) | 53.46 (-23.3 to 178.34) | 6.89 (1.23 to 17.88) |
| Bangladesh | 30-34 years | Males | NA | 73.51 (-30.66 to 229.47) | 10.91 (2.08 to 25.13) |
| Bangladesh | 30-34 years | Females | 264.41 (-81.52 to 615.92) | 36.21 (-11.34 to 125.21) | 3.44 (0 to 10.08) |
| Bangladesh | 35-39 years | Both sexes | 133.89 (-41.18 to 299.74) | 55.54 (-22.8 to 170.26) | 3.2 (0.02 to 8.68) |
| Bangladesh | 35-39 years | Males | NA | 69.06 (-31.06 to 191.8) | 6.11 (0.02 to 15.62) |
| Bangladesh | 35-39 years | Females | 255.69 (-78.64 to 572.42) | 43.25 (-15.32 to 148.06) | 0.56 (0 to 2.32) |
| Bangladesh | 40-44 years | Both sexes | 127.01 (-35.69 to 301.06) | 63.8 (-31.58 to 182.14) | 1.47 (0 to 5.84) |
| Bangladesh | 40-44 years | Males | NA | 83.93 (-50.53 to 230.02) | 2.94 (0 to 11.65) |
| Bangladesh | 40-44 years | Females | 254.68 (-71.57 to 603.68) | 43.57 (-13.25 to 146.42) | 0 (0 to 0) |
| Bangladesh | 45-49 years | Both sexes | 128.03 (-33.56 to 307.76) | 60.11 (-29.8 to 183.99) | 0.08 (0 to 0.56) |
| Bangladesh | 45-49 years | Males | NA | 78.63 (-45.19 to 225.84) | 0.15 (0 to 1.09) |
| Bangladesh | 45-49 years | Females | 262.81 (-68.89 to 631.76) | 40.62 (-14.01 to 134.13) | 0 (0 to 0) |
| Bangladesh | 50-54 years | Both sexes | 106.36 (-21.47 to 295.28) | 59.19 (-25.47 to 202.47) | 0 (0 to 0) |
| Bangladesh | 50-54 years | Males | NA | 69.85 (-30.64 to 252.79) | 0 (0 to 0) |
| Bangladesh | 50-54 years | Females | 217.93 (-44 to 605.02) | 47.99 (-17.84 to 168.76) | 0 (0 to 0) |
| Bangladesh | 55-59 years | Both sexes | 92.31 (-14.27 to 254.06) | 54.55 (-25.66 to 199.27) | 0 (0 to 0) |
| Bangladesh | 55-59 years | Males | NA | 68.2 (-36.04 to 244.98) | 0 (0 to 0) |
| Bangladesh | 55-59 years | Females | 191.98 (-29.68 to 528.35) | 39.81 (-14.83 to 142.96) | 0 (0 to 0) |
| Bangladesh | 60-64 years | Both sexes | 88.91 (-16.79 to 253.98) | 53.12 (-26.07 to 190.75) | 0 (0 to 0) |
| Bangladesh | 60-64 years | Males | NA | 65.26 (-33.39 to 244.87) | 0 (0 to 0) |
| Bangladesh | 60-64 years | Females | 192.78 (-36.4 to 550.71) | 38.93 (-14.28 to 139.26) | 0 (0 to 0) |
| Bangladesh | 65-69 years | Both sexes | 71.49 (-11.38 to 205.27) | 53.33 (-24.11 to 211.22) | 0 (0 to 0) |
| Bangladesh | 65-69 years | Males | NA | 63.08 (-30.65 to 242.92) | 0 (0 to 0) |
| Bangladesh | 65-69 years | Females | 158.6 (-25.26 to 455.4) | 41.45 (-14.65 to 181.16) | 0 (0 to 0) |
| Bangladesh | 70-74 years | Both sexes | 64.42 (-9.59 to 200.25) | 46.2 (-16.33 to 195.13) | 0 (0 to 0) |
| Bangladesh | 70-74 years | Males | NA | 55.63 (-20.8 to 225.41) | 0 (0 to 0) |
| Bangladesh | 70-74 years | Females | 145.63 (-21.69 to 452.68) | 34.32 (-10.69 to 158.32) | 0 (0 to 0) |
| Bangladesh | 75-79 years | Both sexes | 53.09 (-7.84 to 172.69) | 41.08 (-14.13 to 153.98) | 0 (0 to 0) |
| Bangladesh | 75-79 years | Males | NA | 50.46 (-18.27 to 180.63) | 0 (0 to 0) |
| Bangladesh | 75-79 years | Females | 119.07 (-17.59 to 387.3) | 29.42 (-9.03 to 131.57) | 0 (0 to 0) |
| Bangladesh | 80-84 years | Both sexes | 39.52 (-6.08 to 147.26) | 30.62 (-10.06 to 121.4) | 0 (0 to 0) |
| Bangladesh | 80-84 years | Males | NA | 38.89 (-13.83 to 142.71) | 0 (0 to 0) |
| Bangladesh | 80-84 years | Females | 87.55 (-13.46 to 326.22) | 20.56 (-5.67 to 98.9) | 0 (0 to 0) |
| Bangladesh | 85-89 years | Both sexes | 36.83 (-6.06 to 120.75) | 27.96 (-9.34 to 113.24) | 0 (0 to 0) |
| Bangladesh | 85-89 years | Males | NA | 35.52 (-12.06 to 138.38) | 0 (0 to 0) |
| Bangladesh | 85-89 years | Females | 84.9 (-13.97 to 278.32) | 18.1 (-5.67 to 83.03) | 0 (0 to 0) |
| Bangladesh | 90-94 years | Both sexes | 33.99 (-4.64 to 118.52) | 26.59 (-9.91 to 110.05) | 0 (0 to 0) |
| Bangladesh | 90-94 years | Males | NA | 33.4 (-12.6 to 139.67) | 0 (0 to 0) |
| Bangladesh | 90-94 years | Females | 84.74 (-11.57 to 295.52) | 16.42 (-5.71 to 71.64) | 0 (0 to 0) |
| Bangladesh | 95+ years | Both sexes | 30.43 (-3.94 to 98.75) | 26.77 (-10.64 to 107.66) | 0 (0 to 0) |
| Bangladesh | 95+ years | Males | NA | 32.86 (-13.37 to 124.98) | 0 (0 to 0) |
| Bangladesh | 95+ years | Females | 84.52 (-10.93 to 274.25) | 15.94 (-5.59 to 77.86) | 0 (0 to 0) |
| Barbados | <5 years | Both sexes | NA | NA | 0 (0 to 0) |
| Barbados | <5 years | Males | NA | NA | 0 (0 to 0) |
| Barbados | <5 years | Females | NA | NA | 0 (0 to 0) |
| Barbados | 5-9 years | Both sexes | NA | NA | 3.44 (0.8 to 10.07) |
| Barbados | 5-9 years | Males | NA | NA | 4.83 (1.09 to 13.21) |
| Barbados | 5-9 years | Females | NA | NA | 1.99 (0.44 to 5.99) |
| Barbados | 10-14 years | Both sexes | NA | NA | 47.38 (16.08 to 104.49) |
| Barbados | 10-14 years | Males | NA | NA | 61.69 (20.97 to 129.61) |
| Barbados | 10-14 years | Females | NA | NA | 32.36 (9.57 to 73.06) |
| Barbados | 15-19 years | Both sexes | 40.93 (-3.82 to 157.63) | 71.69 (-25.42 to 284.13) | 49.92 (17.6 to 107.1) |
| Barbados | 15-19 years | Males | NA | 48.2 (-16.14 to 181.03) | 57.05 (20.97 to 119.62) |
| Barbados | 15-19 years | Females | 83.7 (-7.8 to 322.31) | 96.24 (-33.72 to 378.97) | 42.47 (12.85 to 101.97) |
| Barbados | 20-24 years | Both sexes | 109.13 (-16.29 to 358.31) | 87.82 (-25.84 to 325.99) | 48.84 (17 to 115.66) |
| Barbados | 20-24 years | Males | NA | 55.72 (-15.3 to 209.13) | 55.01 (21.78 to 125.07) |
| Barbados | 20-24 years | Females | 220.91 (-32.97 to 725.37) | 120.7 (-36.29 to 455.21) | 42.53 (11.63 to 109.09) |
| Barbados | 25-29 years | Both sexes | 158.7 (-31.81 to 516.11) | 95.38 (-27.83 to 380.26) | 38.84 (11.85 to 85.33) |
| Barbados | 25-29 years | Males | NA | 58.73 (-16.38 to 244.13) | 45.31 (14.31 to 97.55) |
| Barbados | 25-29 years | Females | 314.15 (-62.97 to 1021.64) | 131.28 (-38.86 to 518.48) | 32.51 (4.43 to 79.87) |
| Barbados | 30-34 years | Both sexes | 175.73 (-33.68 to 545.32) | 103.98 (-33.52 to 434.34) | 28.43 (5.15 to 69.21) |
| Barbados | 30-34 years | Males | NA | 63.57 (-20.22 to 263.83) | 35.83 (7.79 to 82.97) |
| Barbados | 30-34 years | Females | 343.53 (-65.85 to 1066.05) | 142.56 (-46.38 to 597.57) | 21.35 (0 to 59.15) |
| Barbados | 35-39 years | Both sexes | 174.48 (-36.69 to 496.26) | 101.57 (-34.62 to 420.94) | 13.16 (0.07 to 34.47) |
| Barbados | 35-39 years | Males | NA | 61.99 (-20.66 to 249.09) | 22.66 (0.15 to 56.29) |
| Barbados | 35-39 years | Females | 338.1 (-71.1 to 961.62) | 138.69 (-47.7 to 576.75) | 4.26 (0 to 19.08) |
| Barbados | 40-44 years | Both sexes | 167.57 (-33.42 to 462.85) | 98.36 (-36.44 to 354.63) | 5.49 (0 to 19.23) |
| Barbados | 40-44 years | Males | NA | 60.78 (-21.02 to 219.52) | 11.42 (0 to 40.04) |
| Barbados | 40-44 years | Females | 322.41 (-64.3 to 890.53) | 133.09 (-50.69 to 479.47) | 0 (0 to 0) |
| Barbados | 45-49 years | Both sexes | 161.58 (-31.95 to 444.03) | 93.53 (-35.09 to 339.42) | 0.36 (0 to 2.68) |
| Barbados | 45-49 years | Males | NA | 58.98 (-19.36 to 214.6) | 0.75 (0 to 5.6) |
| Barbados | 45-49 years | Females | 310.2 (-61.34 to 852.41) | 125.3 (-49.56 to 453.42) | 0 (0 to 0) |
| Barbados | 50-54 years | Both sexes | 138.75 (-21.94 to 422.23) | 90.16 (-37.11 to 341.32) | 0 (0 to 0) |
| Barbados | 50-54 years | Males | NA | 57.68 (-22.35 to 226.09) | 0 (0 to 0) |
| Barbados | 50-54 years | Females | 263.64 (-41.69 to 802.26) | 119.4 (-50.72 to 449.33) | 0 (0 to 0) |
| Barbados | 55-59 years | Both sexes | 124.63 (-19.4 to 357.44) | 85.27 (-36.69 to 333.91) | 0 (0 to 0) |
| Barbados | 55-59 years | Males | NA | 55.86 (-22.97 to 225.03) | 0 (0 to 0) |
| Barbados | 55-59 years | Females | 234.86 (-36.56 to 673.58) | 111.28 (-48.83 to 439.19) | 0 (0 to 0) |
| Barbados | 60-64 years | Both sexes | 96.48 (-14.06 to 306.49) | 80.87 (-35.4 to 337.13) | 0 (0 to 0) |
| Barbados | 60-64 years | Males | NA | 54.25 (-22.74 to 220.4) | 0 (0 to 0) |
| Barbados | 60-64 years | Females | 179.16 (-26.11 to 569.11) | 103.68 (-46.25 to 430.76) | 0 (0 to 0) |
| Barbados | 65-69 years | Both sexes | 72.58 (-9.1 to 240.57) | 74.37 (-29.34 to 316.38) | 0 (0 to 0) |
| Barbados | 65-69 years | Males | NA | 50.87 (-19.48 to 216.57) | 0 (0 to 0) |
| Barbados | 65-69 years | Females | 133.17 (-16.7 to 441.44) | 94 (-37.58 to 377.58) | 0 (0 to 0) |
| Barbados | 70-74 years | Both sexes | 64.08 (-6.68 to 227.58) | 60.94 (-19.17 to 273.38) | 0 (0 to 0) |
| Barbados | 70-74 years | Males | NA | 42.33 (-12.35 to 200.54) | 0 (0 to 0) |
| Barbados | 70-74 years | Females | 116.8 (-12.17 to 414.79) | 76.26 (-24.39 to 333.31) | 0 (0 to 0) |
| Barbados | 75-79 years | Both sexes | 50.75 (-5.13 to 177.78) | 52.95 (-15.59 to 215.02) | 0 (0 to 0) |
| Barbados | 75-79 years | Males | NA | 36.88 (-10.52 to 152.38) | 0 (0 to 0) |
| Barbados | 75-79 years | Females | 89.25 (-9.02 to 312.68) | 65.15 (-19.81 to 262.71) | 0 (0 to 0) |
| Barbados | 80-84 years | Both sexes | 36.15 (-3.12 to 141.36) | 38.21 (-10.65 to 161.48) | 0 (0 to 0) |
| Barbados | 80-84 years | Males | NA | 26.33 (-6.76 to 111.26) | 0 (0 to 0) |
| Barbados | 80-84 years | Females | 61.13 (-5.28 to 239.05) | 46.42 (-13.64 to 196.35) | 0 (0 to 0) |
| Barbados | 85-89 years | Both sexes | 36.47 (-3.64 to 144.66) | 34.17 (-10.01 to 145.15) | 0 (0 to 0) |
| Barbados | 85-89 years | Males | NA | 23.31 (-6.59 to 100.83) | 0 (0 to 0) |
| Barbados | 85-89 years | Females | 58.26 (-5.82 to 231.12) | 40.65 (-12.17 to 171.63) | 0 (0 to 0) |
| Barbados | 90-94 years | Both sexes | 38.46 (-3.86 to 156.13) | 31.68 (-9.31 to 135.5) | 0 (0 to 0) |
| Barbados | 90-94 years | Males | NA | 21.44 (-6.38 to 93.54) | 0 (0 to 0) |
| Barbados | 90-94 years | Females | 57.61 (-5.78 to 233.85) | 36.78 (-10.81 to 155.72) | 0 (0 to 0) |
| Barbados | 95+ years | Both sexes | 37.72 (-3.61 to 150.46) | 30.33 (-10.01 to 126.85) | 0 (0 to 0) |
| Barbados | 95+ years | Males | NA | 20.96 (-6.54 to 88.45) | 0 (0 to 0) |
| Barbados | 95+ years | Females | 58.13 (-5.57 to 231.89) | 35.4 (-11.98 to 147.83) | 0 (0 to 0) |
| Belarus | <5 years | Both sexes | NA | NA | 0 (0 to 0) |
| Belarus | <5 years | Males | NA | NA | 0 (0 to 0) |
| Belarus | <5 years | Females | NA | NA | 0 (0 to 0) |
| Belarus | 5-9 years | Both sexes | NA | NA | 9.32 (2.33 to 25.98) |
| Belarus | 5-9 years | Males | NA | NA | 9.01 (2.3 to 24.33) |
| Belarus | 5-9 years | Females | NA | NA | 9.64 (2.07 to 29.05) |
| Belarus | 10-14 years | Both sexes | NA | NA | 109.41 (38.16 to 240.04) |
| Belarus | 10-14 years | Males | NA | NA | 106.26 (37.96 to 211.52) |
| Belarus | 10-14 years | Females | NA | NA | 112.73 (37.38 to 254) |
| Belarus | 15-19 years | Both sexes | 12.89 (-1.25 to 52.06) | 55.21 (-21.01 to 206.62) | 92.93 (34.11 to 191.07) |
| Belarus | 15-19 years | Males | NA | 38.66 (-13.38 to 148.41) | 92.72 (33.92 to 189.36) |
| Belarus | 15-19 years | Females | 26.47 (-2.58 to 106.96) | 72.66 (-27.39 to 274.05) | 93.16 (31.55 to 209.53) |
| Belarus | 20-24 years | Both sexes | 25.46 (-3.07 to 99.96) | 47.86 (-15.1 to 177.27) | 64.6 (22.71 to 136.86) |
| Belarus | 20-24 years | Males | NA | 32.73 (-9.98 to 123.29) | 65.61 (23.33 to 133.41) |
| Belarus | 20-24 years | Females | 52.09 (-6.29 to 204.56) | 63.69 (-20.42 to 232.08) | 63.53 (17.27 to 142.37) |
| Belarus | 25-29 years | Both sexes | 36.2 (-5.34 to 117.4) | 43.2 (-13.18 to 162.28) | 44.2 (11.27 to 99.5) |
| Belarus | 25-29 years | Males | NA | 29.15 (-8.7 to 113.7) | 46.84 (15.35 to 96.5) |
| Belarus | 25-29 years | Females | 72.47 (-10.69 to 235.01) | 57.28 (-18.08 to 221.58) | 41.54 (5.77 to 107.1) |
| Belarus | 30-34 years | Both sexes | 44.22 (-7.47 to 147.63) | 43.07 (-14.6 to 174.65) | 29.8 (5.79 to 75.27) |
| Belarus | 30-34 years | Males | NA | 28.49 (-8.84 to 109.82) | 34.16 (8.08 to 75.78) |
| Belarus | 30-34 years | Females | 87.37 (-14.76 to 291.68) | 57.3 (-20.22 to 231.63) | 25.55 (0 to 75.8) |
| Belarus | 35-39 years | Both sexes | 48.64 (-9.54 to 149.61) | 41.16 (-15.17 to 165.23) | 14.3 (0.09 to 41.72) |
| Belarus | 35-39 years | Males | NA | 27.1 (-9.82 to 103.2) | 22.71 (0.17 to 57.75) |
| Belarus | 35-39 years | Females | 95.77 (-18.78 to 294.61) | 54.78 (-20.55 to 224) | 6.15 (0 to 27.52) |
| Belarus | 40-44 years | Both sexes | 48.68 (-8.34 to 148.05) | 40.8 (-14.73 to 145.59) | 5.23 (0 to 19.62) |
| Belarus | 40-44 years | Males | NA | 27.08 (-9.95 to 100.18) | 10.74 (0 to 40.26) |
| Belarus | 40-44 years | Females | 94.93 (-16.26 to 288.73) | 53.84 (-20.72 to 190.58) | 0 (0 to 0) |
| Belarus | 45-49 years | Both sexes | 55.4 (-9.37 to 162.21) | 39.78 (-15.1 to 141.53) | 0.47 (0 to 3.05) |
| Belarus | 45-49 years | Males | NA | 26.29 (-9.5 to 98.92) | 0.98 (0 to 6.39) |
| Belarus | 45-49 years | Females | 106.07 (-17.94 to 310.59) | 52.13 (-20.26 to 186.27) | 0 (0 to 0) |
| Belarus | 50-54 years | Both sexes | 44.08 (-5.78 to 155.64) | 40.37 (-15.95 to 161.16) | 0 (0 to 0) |
| Belarus | 50-54 years | Males | NA | 26.67 (-10.24 to 110.08) | 0 (0 to 0) |
| Belarus | 50-54 years | Females | 82.28 (-10.8 to 290.55) | 52.24 (-20.71 to 209.36) | 0 (0 to 0) |
| Belarus | 55-59 years | Both sexes | 41.15 (-6.03 to 144.13) | 39.62 (-17.12 to 153.13) | 0 (0 to 0) |
| Belarus | 55-59 years | Males | NA | 26.12 (-9.9 to 102.92) | 0 (0 to 0) |
| Belarus | 55-59 years | Females | 75.1 (-11.01 to 263.02) | 50.76 (-22.55 to 194.32) | 0 (0 to 0) |
| Belarus | 60-64 years | Both sexes | 32.89 (-4.12 to 125) | 39.85 (-17.74 to 152.24) | 0 (0 to 0) |
| Belarus | 60-64 years | Males | NA | 26.13 (-11.54 to 107.65) | 0 (0 to 0) |
| Belarus | 60-64 years | Females | 58.23 (-7.29 to 221.28) | 50.41 (-22.51 to 188.56) | 0 (0 to 0) |
| Belarus | 65-69 years | Both sexes | 26.3 (-2.6 to 95.65) | 39.14 (-15.66 to 168.86) | 0 (0 to 0) |
| Belarus | 65-69 years | Males | NA | 24.94 (-8.6 to 111.11) | 0 (0 to 0) |
| Belarus | 65-69 years | Females | 43.97 (-4.35 to 159.94) | 48.68 (-20.57 to 207.95) | 0 (0 to 0) |
| Belarus | 70-74 years | Both sexes | 25.58 (-2.11 to 100.46) | 34.5 (-10.61 to 154.17) | 0 (0 to 0) |
| Belarus | 70-74 years | Males | NA | 21.44 (-6.21 to 94.2) | 0 (0 to 0) |
| Belarus | 70-74 years | Females | 40.03 (-3.3 to 157.2) | 41.87 (-13.18 to 188.13) | 0 (0 to 0) |
| Belarus | 75-79 years | Both sexes | 21.12 (-1.75 to 80.45) | 31.37 (-9.2 to 121.79) | 0 (0 to 0) |
| Belarus | 75-79 years | Males | NA | 19.01 (-5.18 to 77.48) | 0 (0 to 0) |
| Belarus | 75-79 years | Females | 30.94 (-2.56 to 117.86) | 37.12 (-11.09 to 146.58) | 0 (0 to 0) |
| Belarus | 80-84 years | Both sexes | 15.73 (-1.23 to 64.94) | 23.89 (-6.87 to 97.64) | 0 (0 to 0) |
| Belarus | 80-84 years | Males | NA | 13.88 (-3.6 to 55.5) | 0 (0 to 0) |
| Belarus | 80-84 years | Females | 21 (-1.65 to 86.71) | 27.25 (-7.95 to 111.93) | 0 (0 to 0) |
| Belarus | 85-89 years | Both sexes | 16.27 (-1.31 to 67.96) | 21.98 (-6.29 to 89.08) | 0 (0 to 0) |
| Belarus | 85-89 years | Males | NA | 12.62 (-3.81 to 51.71) | 0 (0 to 0) |
| Belarus | 85-89 years | Females | 20.54 (-1.66 to 85.8) | 24.44 (-6.94 to 99.39) | 0 (0 to 0) |
| Belarus | 90-94 years | Both sexes | 16.84 (-1.54 to 74.01) | 20.67 (-6.38 to 91.12) | 0 (0 to 0) |
| Belarus | 90-94 years | Males | NA | 11.72 (-3.62 to 49.76) | 0 (0 to 0) |
| Belarus | 90-94 years | Females | 20.64 (-1.89 to 90.7) | 22.69 (-7.1 to 99.29) | 0 (0 to 0) |
| Belarus | 95+ years | Both sexes | 17.93 (-1.62 to 78.45) | 20.58 (-6.54 to 85.46) | 0 (0 to 0) |
| Belarus | 95+ years | Males | NA | 11.55 (-3.67 to 49.17) | 0 (0 to 0) |
| Belarus | 95+ years | Females | 21.28 (-1.92 to 93.15) | 22.27 (-7.1 to 92.46) | 0 (0 to 0) |
| Belgium | <5 years | Both sexes | NA | NA | 0 (0 to 0) |
| Belgium | <5 years | Males | NA | NA | 0 (0 to 0) |
| Belgium | <5 years | Females | NA | NA | 0 (0 to 0) |
| Belgium | 5-9 years | Both sexes | NA | NA | 27 (6.89 to 76.83) |
| Belgium | 5-9 years | Males | NA | NA | 36.77 (9.26 to 102.78) |
| Belgium | 5-9 years | Females | NA | NA | 16.76 (3.66 to 49.93) |
| Belgium | 10-14 years | Both sexes | NA | NA | 181.48 (60.71 to 399.75) |
| Belgium | 10-14 years | Males | NA | NA | 198.52 (67.4 to 401.19) |
| Belgium | 10-14 years | Females | NA | NA | 163.63 (47.26 to 395.25) |
| Belgium | 15-19 years | Both sexes | 48.97 (-3.28 to 210.53) | 141 (-50.8 to 627.36) | 220.49 (75.18 to 495.69) |
| Belgium | 15-19 years | Males | NA | 69.41 (-20.34 to 292.58) | 240.49 (92.94 to 501.07) |
| Belgium | 15-19 years | Females | 100.32 (-6.71 to 431.32) | 216.09 (-83.85 to 967.94) | 199.51 (58.89 to 477.91) |
| Belgium | 20-24 years | Both sexes | 83.37 (-11.68 to 303.98) | 110.64 (-37.56 to 355.19) | 149.07 (51.68 to 332.91) |
| Belgium | 20-24 years | Males | NA | 52.11 (-15.07 to 212.26) | 172.5 (66.05 to 345.12) |
| Belgium | 20-24 years | Females | 169.75 (-23.79 to 618.96) | 171.29 (-60.02 to 514.75) | 124.78 (33.23 to 316.91) |
| Belgium | 25-29 years | Both sexes | 133.65 (-24 to 411.73) | 99.78 (-37.68 to 300.27) | 96.77 (29.35 to 216.34) |
| Belgium | 25-29 years | Males | NA | 44.78 (-13.11 to 202.61) | 119.38 (37 to 243.6) |
| Belgium | 25-29 years | Females | 268.82 (-48.27 to 828.15) | 155.41 (-57.4 to 463.77) | 73.9 (10.56 to 183.39) |
| Belgium | 30-34 years | Both sexes | 143.61 (-27.27 to 393.24) | 94.12 (-37.66 to 307.61) | 62.07 (11.52 to 139.78) |
| Belgium | 30-34 years | Males | NA | 50.61 (-16.12 to 203.08) | 83.46 (18.84 to 174.17) |
| Belgium | 30-34 years | Females | 287.17 (-54.53 to 786.35) | 137.62 (-58.06 to 421.34) | 40.68 (0 to 114.17) |
| Belgium | 35-39 years | Both sexes | 134.83 (-23.61 to 344.97) | 92.23 (-40.31 to 285.87) | 28.45 (0.02 to 72.97) |
| Belgium | 35-39 years | Males | NA | 44.58 (-14.96 to 176.16) | 50.05 (0 to 124.96) |
| Belgium | 35-39 years | Females | 268.98 (-47.11 to 688.2) | 139.64 (-63.31 to 421.81) | 6.96 (0 to 28.34) |
| Belgium | 40-44 years | Both sexes | 121.93 (-20.65 to 301.57) | 87.98 (-39.94 to 258.25) | 11.32 (0 to 41.78) |
| Belgium | 40-44 years | Males | NA | 49.09 (-17.9 to 174.46) | 22.59 (0 to 83.41) |
| Belgium | 40-44 years | Females | 244.28 (-41.38 to 604.17) | 126.99 (-62.33 to 382.18) | 0 (0 to 0) |
| Belgium | 45-49 years | Both sexes | 114.94 (-19.78 to 307.93) | 79.99 (-35.35 to 249.09) | 0.74 (0 to 5.57) |
| Belgium | 45-49 years | Males | NA | 43.72 (-16.13 to 155.22) | 1.46 (0 to 11.03) |
| Belgium | 45-49 years | Females | 232.08 (-39.95 to 621.74) | 116.96 (-57.15 to 340.15) | 0 (0 to 0) |
| Belgium | 50-54 years | Both sexes | 87.93 (-16.11 to 253.46) | 78.73 (-40.92 to 273.07) | 0 (0 to 0) |
| Belgium | 50-54 years | Males | NA | 40.01 (-16.62 to 154.75) | 0 (0 to 0) |
| Belgium | 50-54 years | Females | 177.67 (-32.56 to 512.13) | 118.25 (-65.4 to 374.9) | 0 (0 to 0) |
| Belgium | 55-59 years | Both sexes | 74.29 (-12.46 to 213.93) | 71.65 (-35.62 to 247.38) | 0 (0 to 0) |
| Belgium | 55-59 years | Males | NA | 43.87 (-18.36 to 164.86) | 0 (0 to 0) |
| Belgium | 55-59 years | Females | 149.79 (-25.13 to 431.35) | 99.89 (-48.61 to 305.53) | 0 (0 to 0) |
| Belgium | 60-64 years | Both sexes | 52.64 (-7.59 to 171.34) | 70.2 (-29.88 to 234.26) | 0 (0 to 0) |
| Belgium | 60-64 years | Males | NA | 41.53 (-18.34 to 159.87) | 0 (0 to 0) |
| Belgium | 60-64 years | Females | 104.37 (-15.06 to 339.74) | 98.38 (-46.69 to 321.49) | 0 (0 to 0) |
| Belgium | 65-69 years | Both sexes | 38.24 (-5.09 to 129.01) | 65.13 (-28.18 to 219.68) | 0 (0 to 0) |
| Belgium | 65-69 years | Males | NA | 42.25 (-16.35 to 163.14) | 0 (0 to 0) |
| Belgium | 65-69 years | Females | 74.23 (-9.88 to 250.43) | 86.65 (-36.23 to 287.43) | 0 (0 to 0) |
| Belgium | 70-74 years | Both sexes | 36 (-4.41 to 121.22) | 50 (-17.64 to 186.01) | 0 (0 to 0) |
| Belgium | 70-74 years | Males | NA | 32.08 (-9.89 to 138.89) | 0 (0 to 0) |
| Belgium | 70-74 years | Females | 68.35 (-8.38 to 230.16) | 66.1 (-19.16 to 226.06) | 0 (0 to 0) |
| Belgium | 75-79 years | Both sexes | 25.18 (-2.27 to 104.18) | 49.56 (-15.09 to 201.53) | 0 (0 to 0) |
| Belgium | 75-79 years | Males | NA | 28.02 (-7.11 to 113.55) | 0 (0 to 0) |
| Belgium | 75-79 years | Females | 46.04 (-4.15 to 190.47) | 67.39 (-21.64 to 277.57) | 0 (0 to 0) |
| Belgium | 80-84 years | Both sexes | 17.48 (-1.62 to 76.39) | 36.68 (-11.66 to 156.03) | 0 (0 to 0) |
| Belgium | 80-84 years | Males | NA | 19.92 (-5.17 to 79.6) | 0 (0 to 0) |
| Belgium | 80-84 years | Females | 30.03 (-2.79 to 131.21) | 48.7 (-16.73 to 211.47) | 0 (0 to 0) |
| Belgium | 85-89 years | Both sexes | 17.61 (-1.6 to 71.02) | 32.95 (-9.62 to 145.8) | 0 (0 to 0) |
| Belgium | 85-89 years | Males | NA | 17.19 (-4.66 to 73.38) | 0 (0 to 0) |
| Belgium | 85-89 years | Females | 28.14 (-2.56 to 113.5) | 42.38 (-12.52 to 192.35) | 0 (0 to 0) |
| Belgium | 90-94 years | Both sexes | 18.67 (-1.69 to 80.71) | 30.69 (-8.86 to 140.23) | 0 (0 to 0) |
| Belgium | 90-94 years | Males | NA | 15.57 (-4.72 to 69.13) | 0 (0 to 0) |
| Belgium | 90-94 years | Females | 26.95 (-2.44 to 116.5) | 37.4 (-10.69 to 171.62) | 0 (0 to 0) |
| Belgium | 95+ years | Both sexes | 20.53 (-2.07 to 90.81) | 30.7 (-9.7 to 131.9) | 0 (0 to 0) |
| Belgium | 95+ years | Males | NA | 14.96 (-4.49 to 62.97) | 0 (0 to 0) |
| Belgium | 95+ years | Females | 26.44 (-2.67 to 116.99) | 35.24 (-11.21 to 154.18) | 0 (0 to 0) |
| Belize | <5 years | Both sexes | NA | NA | 0 (0 to 0) |
| Belize | <5 years | Males | NA | NA | 0 (0 to 0) |
| Belize | <5 years | Females | NA | NA | 0 (0 to 0) |
| Belize | 5-9 years | Both sexes | NA | NA | 8.09 (2.16 to 23.16) |
| Belize | 5-9 years | Males | NA | NA | 10.74 (2.77 to 30.22) |
| Belize | 5-9 years | Females | NA | NA | 5.33 (1.2 to 15.92) |
| Belize | 10-14 years | Both sexes | NA | NA | 97.96 (37.57 to 205.64) |
| Belize | 10-14 years | Males | NA | NA | 117.41 (43.54 to 242.87) |
| Belize | 10-14 years | Females | NA | NA | 77.66 (24.75 to 182.19) |
| Belize | 15-19 years | Both sexes | 37.25 (-3.03 to 136.11) | 56.52 (-24.68 to 240.41) | 106.15 (41.82 to 216.35) |
| Belize | 15-19 years | Males | NA | 44.63 (-17.38 to 185.02) | 110.73 (44.21 to 211.15) |
| Belize | 15-19 years | Females | 75.26 (-6.12 to 274.97) | 68.66 (-30.27 to 270.38) | 101.48 (34.27 to 229.49) |
| Belize | 20-24 years | Both sexes | 90.59 (-12.04 to 316.25) | 71.61 (-26.93 to 256.68) | 102.41 (38.57 to 228.02) |
| Belize | 20-24 years | Males | NA | 55.09 (-20.8 to 195.76) | 105.41 (43.18 to 220.45) |
| Belize | 20-24 years | Females | 178.11 (-23.68 to 621.75) | 87.57 (-33.34 to 320.4) | 99.5 (30.15 to 244.24) |
| Belize | 25-29 years | Both sexes | 131.51 (-19.87 to 455.67) | 80.08 (-33.18 to 307.4) | 85.72 (23.36 to 183.32) |
| Belize | 25-29 years | Males | NA | 59.38 (-24.3 to 218.07) | 92.42 (29.96 to 180.05) |
| Belize | 25-29 years | Females | 249.92 (-37.76 to 865.99) | 98.71 (-40.34 to 387.84) | 79.7 (12.22 to 194.25) |
| Belize | 30-34 years | Both sexes | 150.4 (-22.22 to 474.29) | 83.23 (-28.05 to 298.05) | 60.95 (9.85 to 141.37) |
| Belize | 30-34 years | Males | NA | 60.4 (-18.91 to 220.33) | 71.99 (16.26 to 150.78) |
| Belize | 30-34 years | Females | 281.76 (-41.63 to 888.55) | 103.17 (-35.82 to 384.96) | 51.31 (0 to 136.97) |
| Belize | 35-39 years | Both sexes | 146.13 (-23.02 to 457.38) | 79.53 (-25.17 to 273.43) | 26.8 (0.17 to 77.72) |
| Belize | 35-39 years | Males | NA | 58.87 (-19.89 to 205.73) | 45.86 (0.26 to 116.55) |
| Belize | 35-39 years | Females | 276.71 (-43.58 to 866.09) | 97.98 (-29.88 to 337.82) | 9.78 (0 to 41.39) |
| Belize | 40-44 years | Both sexes | 148.7 (-25.92 to 420.07) | 78.37 (-27.37 to 255.14) | 10.89 (0 to 40.14) |
| Belize | 40-44 years | Males | NA | 54.37 (-20.16 to 179.33) | 22.78 (0 to 83.94) |
| Belize | 40-44 years | Females | 284.96 (-49.66 to 804.98) | 100.37 (-34.05 to 326.78) | 0 (0 to 0) |
| Belize | 45-49 years | Both sexes | 126.92 (-19.94 to 385.41) | 71.81 (-25.55 to 252.47) | 0.74 (0 to 4.73) |
| Belize | 45-49 years | Males | NA | 49.38 (-17.47 to 173.21) | 1.55 (0 to 9.88) |
| Belize | 45-49 years | Females | 243.62 (-38.27 to 739.76) | 92.44 (-32.97 to 321.85) | 0 (0 to 0) |
| Belize | 50-54 years | Both sexes | 101.6 (-13.63 to 333.87) | 69.32 (-25.57 to 264.51) | 0 (0 to 0) |
| Belize | 50-54 years | Males | NA | 54.65 (-20.4 to 210.92) | 0 (0 to 0) |
| Belize | 50-54 years | Females | 195.25 (-26.19 to 641.6) | 82.84 (-30.33 to 313.9) | 0 (0 to 0) |
| Belize | 55-59 years | Both sexes | 92.76 (-13.16 to 282.37) | 64.98 (-23.66 to 238.07) | 0 (0 to 0) |
| Belize | 55-59 years | Males | NA | 50.87 (-18.11 to 204.32) | 0 (0 to 0) |
| Belize | 55-59 years | Females | 182.59 (-25.9 to 555.82) | 78.65 (-29.65 to 287.74) | 0 (0 to 0) |
| Belize | 60-64 years | Both sexes | 64.4 (-8.28 to 220.29) | 59.1 (-23.83 to 227.02) | 0 (0 to 0) |
| Belize | 60-64 years | Males | NA | 48.85 (-19.04 to 189.07) | 0 (0 to 0) |
| Belize | 60-64 years | Females | 130.9 (-16.82 to 447.76) | 69.68 (-28.04 to 268.7) | 0 (0 to 0) |
| Belize | 65-69 years | Both sexes | 49.64 (-5.26 to 164.02) | 59.62 (-21.98 to 232.6) | 0 (0 to 0) |
| Belize | 65-69 years | Males | NA | 47.38 (-16.53 to 190.94) | 0 (0 to 0) |
| Belize | 65-69 years | Females | 101.8 (-10.8 to 336.4) | 72.48 (-27.79 to 277.16) | 0 (0 to 0) |
| Belize | 70-74 years | Both sexes | 50.04 (-5.12 to 173.18) | 48.53 (-16.09 to 209.39) | 0 (0 to 0) |
| Belize | 70-74 years | Males | NA | 42.42 (-12.27 to 189.66) | 0 (0 to 0) |
| Belize | 70-74 years | Females | 102.09 (-10.45 to 353.32) | 54.89 (-19.26 to 226.81) | 0 (0 to 0) |
| Belize | 75-79 years | Both sexes | 33.55 (-3.08 to 127.4) | 44.45 (-13.32 to 181.39) | 0 (0 to 0) |
| Belize | 75-79 years | Males | NA | 35.99 (-10.07 to 153.23) | 0 (0 to 0) |
| Belize | 75-79 years | Females | 67.03 (-6.15 to 254.55) | 52.9 (-16.21 to 206.15) | 0 (0 to 0) |
| Belize | 80-84 years | Both sexes | 23.68 (-2.22 to 96.64) | 29.15 (-7.55 to 125.65) | 0 (0 to 0) |
| Belize | 80-84 years | Males | NA | 24.66 (-6.41 to 109.2) | 0 (0 to 0) |
| Belize | 80-84 years | Females | 45.42 (-4.25 to 185.37) | 33.26 (-8.67 to 142.56) | 0 (0 to 0) |
| Belize | 85-89 years | Both sexes | 23.73 (-2.2 to 97.4) | 25.64 (-7.19 to 108.25) | 0 (0 to 0) |
| Belize | 85-89 years | Males | NA | 21.8 (-6.28 to 93.11) | 0 (0 to 0) |
| Belize | 85-89 years | Females | 43.09 (-4 to 176.85) | 28.76 (-8.26 to 127.06) | 0 (0 to 0) |
| Belize | 90-94 years | Both sexes | 23.04 (-2.15 to 91.91) | 23.33 (-7.02 to 107.01) | 0 (0 to 0) |
| Belize | 90-94 years | Males | NA | 19.85 (-6.27 to 93.1) | 0 (0 to 0) |
| Belize | 90-94 years | Females | 42.54 (-3.96 to 169.69) | 26.29 (-7.74 to 119.96) | 0 (0 to 0) |
| Belize | 95+ years | Both sexes | 26.13 (-2.36 to 102.93) | 22.87 (-7.6 to 96.28) | 0 (0 to 0) |
| Belize | 95+ years | Males | NA | 19.24 (-6.17 to 80.13) | 0 (0 to 0) |
| Belize | 95+ years | Females | 43.05 (-3.89 to 169.62) | 25.22 (-8.55 to 106.11) | 0 (0 to 0) |
| Benin | <5 years | Both sexes | NA | NA | 0 (0 to 0) |
| Benin | <5 years | Males | NA | NA | 0 (0 to 0) |
| Benin | <5 years | Females | NA | NA | 0 (0 to 0) |
| Benin | 5-9 years | Both sexes | NA | NA | 9.56 (2.63 to 23.11) |
| Benin | 5-9 years | Males | NA | NA | 10.17 (2.97 to 24.71) |
| Benin | 5-9 years | Females | NA | NA | 8.92 (2.35 to 23.07) |
| Benin | 10-14 years | Both sexes | NA | NA | 224.82 (82.25 to 472.03) |
| Benin | 10-14 years | Males | NA | NA | 183.72 (64.42 to 401.2) |
| Benin | 10-14 years | Females | NA | NA | 266.49 (85.39 to 581.18) |
| Benin | 15-19 years | Both sexes | 61.53 (-5.97 to 272.92) | 136.44 (-49.65 to 459.63) | 178.84 (69.73 to 378.19) |
| Benin | 15-19 years | Males | NA | 113.41 (-42.75 to 417.29) | 158.63 (61.8 to 313.07) |
| Benin | 15-19 years | Females | 120.91 (-11.73 to 536.26) | 158.67 (-59.69 to 554.35) | 198.33 (59.16 to 430.79) |
| Benin | 20-24 years | Both sexes | 112.79 (-15.11 to 340.8) | 130.64 (-43.98 to 489.71) | 126.45 (47.32 to 276.25) |
| Benin | 20-24 years | Males | NA | 98.85 (-30.62 to 348.57) | 118.05 (46.08 to 238.28) |
| Benin | 20-24 years | Females | 215.43 (-28.86 to 650.94) | 159.57 (-55.73 to 620.26) | 134.1 (40.04 to 309.66) |
| Benin | 25-29 years | Both sexes | 145.44 (-25.18 to 397.04) | 115.1 (-41.63 to 415.77) | 84.53 (24.96 to 191.46) |
| Benin | 25-29 years | Males | NA | 89.94 (-31.12 to 310.47) | 88.84 (31.33 to 184.36) |
| Benin | 25-29 years | Females | 279.93 (-48.47 to 764.21) | 138.37 (-49.08 to 514.33) | 80.54 (13.8 to 205.15) |
| Benin | 30-34 years | Both sexes | 134.13 (-23.45 to 356.48) | 102.89 (-39.65 to 371.14) | 47.05 (8.1 to 106.18) |
| Benin | 30-34 years | Males | NA | 87.05 (-33.34 to 323.26) | 59.67 (13.4 to 129.68) |
| Benin | 30-34 years | Females | 262.1 (-45.83 to 696.6) | 118.01 (-46.69 to 424.58) | 35.01 (0 to 96.17) |
| Benin | 35-39 years | Both sexes | 120.97 (-21.94 to 317.53) | 91.78 (-35.68 to 342.86) | 20.97 (0.21 to 54.08) |
| Benin | 35-39 years | Males | NA | 81.77 (-30.86 to 285.1) | 35.93 (0.26 to 85.5) |
| Benin | 35-39 years | Females | 234.99 (-42.63 to 616.84) | 101.22 (-40.22 to 386.14) | 6.87 (0 to 24.61) |
| Benin | 40-44 years | Both sexes | 116.37 (-25.15 to 300.03) | 89.44 (-33.61 to 299.67) | 7.28 (0 to 25.97) |
| Benin | 40-44 years | Males | NA | 81.67 (-30.66 to 258.72) | 15.34 (0 to 54.74) |
| Benin | 40-44 years | Females | 221.43 (-47.85 to 570.88) | 96.46 (-36.28 to 336.67) | 0 (0 to 0) |
| Benin | 45-49 years | Both sexes | 110.65 (-20.99 to 288.28) | 87.31 (-34.32 to 293.81) | 0.71 (0 to 4.32) |
| Benin | 45-49 years | Males | NA | 80.68 (-34.33 to 262.02) | 1.49 (0 to 9.08) |
| Benin | 45-49 years | Females | 210.96 (-40.02 to 549.62) | 93.32 (-36.94 to 327.67) | 0 (0 to 0) |
| Benin | 50-54 years | Both sexes | 106.68 (-14.91 to 332.89) | 87.89 (-38.71 to 342.27) | 0 (0 to 0) |
| Benin | 50-54 years | Males | NA | 81.58 (-35.83 to 304.13) | 0 (0 to 0) |
| Benin | 50-54 years | Females | 209.67 (-29.31 to 654.26) | 93.99 (-39.94 to 380.27) | 0 (0 to 0) |
| Benin | 55-59 years | Both sexes | 97.37 (-13.76 to 305.78) | 86.65 (-38.34 to 322.79) | 0 (0 to 0) |
| Benin | 55-59 years | Males | NA | 80.38 (-36.06 to 288.69) | 0 (0 to 0) |
| Benin | 55-59 years | Females | 191.91 (-27.12 to 602.68) | 92.75 (-40.84 to 355.96) | 0 (0 to 0) |
| Benin | 60-64 years | Both sexes | 78.28 (-11 to 242.72) | 85.34 (-41.84 to 331.24) | 0 (0 to 0) |
| Benin | 60-64 years | Males | NA | 79.33 (-39.85 to 289.75) | 0 (0 to 0) |
| Benin | 60-64 years | Females | 151.61 (-21.31 to 470.07) | 90.98 (-43.71 to 371.91) | 0 (0 to 0) |
| Benin | 65-69 years | Both sexes | 61.25 (-7.31 to 219.48) | 82.05 (-34.98 to 338.81) | 0 (0 to 0) |
| Benin | 65-69 years | Males | NA | 76.57 (-31.03 to 300.43) | 0 (0 to 0) |
| Benin | 65-69 years | Females | 116.17 (-13.86 to 416.25) | 86.97 (-38.53 to 369.64) | 0 (0 to 0) |
| Benin | 70-74 years | Both sexes | 56.83 (-5.47 to 207.77) | 70.71 (-22.32 to 311.56) | 0 (0 to 0) |
| Benin | 70-74 years | Males | NA | 66.55 (-22.62 to 286.53) | 0 (0 to 0) |
| Benin | 70-74 years | Females | 105.35 (-10.14 to 385.15) | 74.27 (-22.88 to 323.91) | 0 (0 to 0) |
| Benin | 75-79 years | Both sexes | 45.37 (-4.36 to 169.19) | 62.78 (-18.94 to 247.38) | 0 (0 to 0) |
| Benin | 75-79 years | Males | NA | 59.62 (-18.95 to 229.68) | 0 (0 to 0) |
| Benin | 75-79 years | Females | 81.57 (-7.83 to 304.17) | 65.3 (-19.32 to 258.53) | 0 (0 to 0) |
| Benin | 80-84 years | Both sexes | 33.57 (-3.01 to 141.58) | 46.63 (-13.35 to 183.73) | 0 (0 to 0) |
| Benin | 80-84 years | Males | NA | 44.54 (-13.31 to 162.73) | 0 (0 to 0) |
| Benin | 80-84 years | Females | 56.69 (-5.07 to 239.09) | 48.07 (-13.64 to 194.52) | 0 (0 to 0) |
| Benin | 85-89 years | Both sexes | 35.11 (-3.52 to 142.09) | 42.1 (-12.54 to 176.26) | 0 (0 to 0) |
| Benin | 85-89 years | Males | NA | 40.97 (-12.4 to 159.46) | 0 (0 to 0) |
| Benin | 85-89 years | Females | 54.93 (-5.51 to 222.27) | 42.74 (-13.16 to 182.02) | 0 (0 to 0) |
| Benin | 90-94 years | Both sexes | 38.19 (-3.93 to 153.92) | 39.01 (-12.18 to 165.85) | 0 (0 to 0) |
| Benin | 90-94 years | Males | NA | 38.2 (-12.37 to 155.58) | 0 (0 to 0) |
| Benin | 90-94 years | Females | 55.08 (-5.67 to 221.99) | 39.37 (-12.1 to 171.91) | 0 (0 to 0) |
| Benin | 95+ years | Both sexes | 42.01 (-4.13 to 175.32) | 38.3 (-13.22 to 158.37) | 0 (0 to 0) |
| Benin | 95+ years | Males | NA | 37.68 (-13.11 to 151.15) | 0 (0 to 0) |
| Benin | 95+ years | Females | 55.57 (-5.46 to 231.9) | 38.5 (-13.25 to 157.42) | 0 (0 to 0) |
| Bermuda | <5 years | Both sexes | NA | NA | 0 (0 to 0) |
| Bermuda | <5 years | Males | NA | NA | 0 (0 to 0) |
| Bermuda | <5 years | Females | NA | NA | 0 (0 to 0) |
| Bermuda | 5-9 years | Both sexes | NA | NA | 7.42 (1.86 to 21.92) |
| Bermuda | 5-9 years | Males | NA | NA | 9.86 (2.46 to 27.1) |
| Bermuda | 5-9 years | Females | NA | NA | 4.9 (1.09 to 14.57) |
| Bermuda | 10-14 years | Both sexes | NA | NA | 92.2 (31.65 to 211.76) |
| Bermuda | 10-14 years | Males | NA | NA | 108.39 (37.87 to 241.5) |
| Bermuda | 10-14 years | Females | NA | NA | 75.6 (24.75 to 179.14) |
| Bermuda | 15-19 years | Both sexes | 46.12 (-4.5 to 177.73) | 71.89 (-26.1 to 291.52) | 98.65 (36.34 to 211.91) |
| Bermuda | 15-19 years | Males | NA | 48.21 (-17.74 to 184.49) | 98.41 (38.36 to 203.74) |
| Bermuda | 15-19 years | Females | 94.02 (-9.18 to 362.32) | 96.48 (-34.68 to 374.28) | 98.9 (31.44 to 228.22) |
| Bermuda | 20-24 years | Both sexes | 135.69 (-23.51 to 434.1) | 87.9 (-24.36 to 345.56) | 94.27 (32.85 to 203.7) |
| Bermuda | 20-24 years | Males | NA | 55.11 (-15.13 to 210.06) | 92.82 (36.28 to 194.52) |
| Bermuda | 20-24 years | Females | 270.86 (-46.93 to 866.51) | 120.57 (-33.74 to 478.99) | 95.7 (26.47 to 231.55) |
| Bermuda | 25-29 years | Both sexes | 183.06 (-41.57 to 530.18) | 95.69 (-28.18 to 351.55) | 79.66 (21.63 to 176.76) |
| Bermuda | 25-29 years | Males | NA | 58.46 (-16.51 to 218.19) | 80.95 (24.51 to 166.4) |
| Bermuda | 25-29 years | Females | 357.06 (-81.07 to 1034.12) | 131.08 (-39.28 to 481.03) | 78.44 (11.2 to 193.56) |
| Bermuda | 30-34 years | Both sexes | 191.94 (-45.76 to 536.46) | 103.6 (-35.38 to 427.47) | 58.04 (10.07 to 143.61) |
| Bermuda | 30-34 years | Males | NA | 63.97 (-20.12 to 268.76) | 64.73 (13.99 to 140.22) |
| Bermuda | 30-34 years | Females | 378.69 (-90.28 to 1058.4) | 142.15 (-50.57 to 581.88) | 51.53 (0 to 141.03) |
| Bermuda | 35-39 years | Both sexes | 186.25 (-44.66 to 499.97) | 100.58 (-35.07 to 407.83) | 27.16 (0.14 to 76.21) |
| Bermuda | 35-39 years | Males | NA | 62.27 (-20.8 to 242.94) | 43.15 (0.28 to 105.28) |
| Bermuda | 35-39 years | Females | 368.37 (-88.33 to 988.87) | 138.05 (-49.06 to 552.52) | 11.53 (0 to 50.21) |
| Bermuda | 40-44 years | Both sexes | 178.39 (-37.63 to 472.02) | 97.67 (-35.72 to 355.39) | 10.5 (0 to 37.53) |
| Bermuda | 40-44 years | Males | NA | 60.91 (-20.63 to 219.52) | 21.2 (0 to 75.78) |
| Bermuda | 40-44 years | Females | 353.4 (-74.55 to 935.11) | 133.74 (-50.53 to 484.25) | 0 (0 to 0) |
| Bermuda | 45-49 years | Both sexes | 165.91 (-34.28 to 438.29) | 92.01 (-35.07 to 323.93) | 0.64 (0 to 4.98) |
| Bermuda | 45-49 years | Males | NA | 58.67 (-21.82 to 211.14) | 1.26 (0 to 9.87) |
| Bermuda | 45-49 years | Females | 334.59 (-69.13 to 883.89) | 125.9 (-48.41 to 442.47) | 0 (0 to 0) |
| Bermuda | 50-54 years | Both sexes | 135.96 (-24.64 to 394.83) | 88.72 (-35.46 to 361.77) | 0 (0 to 0) |
| Bermuda | 50-54 years | Males | NA | 57.63 (-22.36 to 230.74) | 0 (0 to 0) |
| Bermuda | 50-54 years | Females | 276.96 (-50.19 to 804.28) | 120.97 (-47.61 to 480.07) | 0 (0 to 0) |
| Bermuda | 55-59 years | Both sexes | 119.73 (-19.17 to 388.54) | 83.95 (-33.67 to 345.09) | 0 (0 to 0) |
| Bermuda | 55-59 years | Males | NA | 55.62 (-22.2 to 226.88) | 0 (0 to 0) |
| Bermuda | 55-59 years | Females | 239.75 (-38.38 to 778.03) | 112.36 (-45.64 to 466.85) | 0 (0 to 0) |
| Bermuda | 60-64 years | Both sexes | 90.93 (-13.35 to 311.96) | 80.57 (-35.96 to 324.24) | 0 (0 to 0) |
| Bermuda | 60-64 years | Males | NA | 54.39 (-23.29 to 224.47) | 0 (0 to 0) |
| Bermuda | 60-64 years | Females | 174.74 (-25.65 to 599.47) | 104.71 (-47.37 to 416.2) | 0 (0 to 0) |
| Bermuda | 65-69 years | Both sexes | 69.88 (-7.98 to 239.38) | 75.41 (-29.39 to 306.9) | 0 (0 to 0) |
| Bermuda | 65-69 years | Males | NA | 51.07 (-18.83 to 208.07) | 0 (0 to 0) |
| Bermuda | 65-69 years | Females | 128.51 (-14.67 to 440.23) | 95.84 (-38.25 to 395.73) | 0 (0 to 0) |
| Bermuda | 70-74 years | Both sexes | 61.19 (-6.72 to 216.42) | 61.56 (-19.23 to 272.76) | 0 (0 to 0) |
| Bermuda | 70-74 years | Males | NA | 42.56 (-13.05 to 197.79) | 0 (0 to 0) |
| Bermuda | 70-74 years | Females | 111.13 (-12.21 to 393.1) | 77.06 (-24.27 to 332.8) | 0 (0 to 0) |
| Bermuda | 75-79 years | Both sexes | 49.46 (-5.73 to 174.35) | 53.68 (-15.97 to 211.23) | 0 (0 to 0) |
| Bermuda | 75-79 years | Males | NA | 36.8 (-10.23 to 145.05) | 0 (0 to 0) |
| Bermuda | 75-79 years | Females | 86.66 (-10.04 to 305.45) | 66.36 (-20.29 to 258.72) | 0 (0 to 0) |
| Bermuda | 80-84 years | Both sexes | 39.93 (-4.9 to 146.01) | 38.57 (-10.44 to 153.34) | 0 (0 to 0) |
| Bermuda | 80-84 years | Males | NA | 26.28 (-6.75 to 109.37) | 0 (0 to 0) |
| Bermuda | 80-84 years | Females | 66.83 (-8.21 to 244.38) | 46.85 (-13.15 to 187.42) | 0 (0 to 0) |
| Bermuda | 85-89 years | Both sexes | 39.83 (-4.87 to 155.24) | 34.23 (-9.94 to 138.79) | 0 (0 to 0) |
| Bermuda | 85-89 years | Males | NA | 23.31 (-6.69 to 99.12) | 0 (0 to 0) |
| Bermuda | 85-89 years | Females | 64.29 (-7.87 to 250.55) | 40.94 (-12.18 to 164.31) | 0 (0 to 0) |
| Bermuda | 90-94 years | Both sexes | 42.01 (-4.83 to 161.57) | 31.61 (-9.24 to 141.23) | 0 (0 to 0) |
| Bermuda | 90-94 years | Males | NA | 21.33 (-6.66 to 97.09) | 0 (0 to 0) |
| Bermuda | 90-94 years | Females | 63.88 (-7.34 to 245.72) | 36.96 (-11.04 to 164.21) | 0 (0 to 0) |
| Bermuda | 95+ years | Both sexes | 49.86 (-5.08 to 184.42) | 32.38 (-10.26 to 129.01) | 0 (0 to 0) |
| Bermuda | 95+ years | Males | NA | 20.79 (-6.51 to 87.27) | 0 (0 to 0) |
| Bermuda | 95+ years | Females | 64.13 (-6.53 to 237.22) | 35.69 (-11.4 to 141.8) | 0 (0 to 0) |
| Bhutan | <5 years | Both sexes | NA | NA | 0 (0 to 0) |
| Bhutan | <5 years | Males | NA | NA | 0 (0 to 0) |
| Bhutan | <5 years | Females | NA | NA | 0 (0 to 0) |
| Bhutan | 5-9 years | Both sexes | NA | NA | 2.27 (0.5 to 6.67) |
| Bhutan | 5-9 years | Males | NA | NA | 2.58 (0.59 to 7.52) |
| Bhutan | 5-9 years | Females | NA | NA | 1.96 (0.36 to 6.4) |
| Bhutan | 10-14 years | Both sexes | NA | NA | 39.63 (14.3 to 90.2) |
| Bhutan | 10-14 years | Males | NA | NA | 44.38 (14.82 to 101.53) |
| Bhutan | 10-14 years | Females | NA | NA | 34.76 (11.13 to 82.08) |
| Bhutan | 15-19 years | Both sexes | 11.17 (-0.97 to 49.26) | 47.41 (-18.74 to 165.51) | 33.91 (13.2 to 71.55) |
| Bhutan | 15-19 years | Males | NA | 49.8 (-22.67 to 183.29) | 37.28 (13.13 to 75.57) |
| Bhutan | 15-19 years | Females | 22.8 (-1.98 to 100.56) | 44.91 (-16.32 to 151.84) | 30.39 (10.15 to 67.59) |
| Bhutan | 20-24 years | Both sexes | 29.28 (-3.87 to 95.65) | 43.99 (-15.37 to 155.13) | 25.4 (9.5 to 58.53) |
| Bhutan | 20-24 years | Males | NA | 46.78 (-18.47 to 151.37) | 27.82 (10.2 to 61.25) |
| Bhutan | 20-24 years | Females | 61.47 (-8.13 to 200.81) | 40.92 (-12.03 to 155.46) | 22.75 (6.18 to 52.63) |
| Bhutan | 25-29 years | Both sexes | 52.87 (-7.65 to 153.16) | 47.3 (-15.68 to 178.69) | 18.06 (5.03 to 41.02) |
| Bhutan | 25-29 years | Males | NA | 48.24 (-17.97 to 179) | 20.39 (6.52 to 44.88) |
| Bhutan | 25-29 years | Females | 112.86 (-16.34 to 326.94) | 46.24 (-12.99 to 168.85) | 15.41 (2.25 to 38.95) |
| Bhutan | 30-34 years | Both sexes | 64.59 (-11.9 to 176.63) | 53.76 (-18.86 to 204.02) | 10 (1.7 to 25.63) |
| Bhutan | 30-34 years | Males | NA | 56.39 (-21.64 to 215.21) | 12.42 (2.85 to 30.31) |
| Bhutan | 30-34 years | Females | 135.78 (-25.02 to 371.3) | 50.86 (-14.32 to 189.83) | 7.33 (0 to 21.7) |
| Bhutan | 35-39 years | Both sexes | 68.04 (-12.11 to 172.81) | 61.23 (-20.74 to 218.34) | 4.72 (0.02 to 13.28) |
| Bhutan | 35-39 years | Males | NA | 61.18 (-23.57 to 219.14) | 7.78 (0.04 to 21.16) |
| Bhutan | 35-39 years | Females | 143.01 (-25.46 to 363.22) | 61.28 (-17.9 to 228.19) | 1.35 (0 to 5.34) |
| Bhutan | 40-44 years | Both sexes | 70.94 (-11.47 to 187.06) | 66.34 (-22.86 to 225.45) | 2.08 (0 to 7.66) |
| Bhutan | 40-44 years | Males | NA | 67.12 (-27.86 to 222.52) | 3.93 (0 to 14.47) |
| Bhutan | 40-44 years | Females | 150.78 (-24.38 to 397.6) | 65.47 (-18.79 to 232.94) | 0 (0 to 0) |
| Bhutan | 45-49 years | Both sexes | 66.29 (-9.38 to 178.52) | 65.93 (-22.95 to 224.15) | 0.15 (0 to 0.96) |
| Bhutan | 45-49 years | Males | NA | 67.6 (-27.8 to 229.35) | 0.28 (0 to 1.83) |
| Bhutan | 45-49 years | Females | 139.95 (-19.81 to 376.86) | 64.07 (-19.51 to 219.89) | 0 (0 to 0) |
| Bhutan | 50-54 years | Both sexes | 52.94 (-7.94 to 161.57) | 64.01 (-23.36 to 233.28) | 0 (0 to 0) |
| Bhutan | 50-54 years | Males | NA | 64.16 (-25.16 to 231.07) | 0 (0 to 0) |
| Bhutan | 50-54 years | Females | 111.69 (-16.75 to 340.88) | 63.84 (-19.17 to 226.67) | 0 (0 to 0) |
| Bhutan | 55-59 years | Both sexes | 48.02 (-6.44 to 147.94) | 56 (-23.37 to 194.88) | 0 (0 to 0) |
| Bhutan | 55-59 years | Males | NA | 59.29 (-26.36 to 211.55) | 0 (0 to 0) |
| Bhutan | 55-59 years | Females | 99.45 (-13.34 to 306.39) | 52.49 (-15.68 to 191.68) | 0 (0 to 0) |
| Bhutan | 60-64 years | Both sexes | 36.69 (-5.16 to 116.56) | 51.61 (-20.73 to 197.31) | 0 (0 to 0) |
| Bhutan | 60-64 years | Males | NA | 54.72 (-24.47 to 212.4) | 0 (0 to 0) |
| Bhutan | 60-64 years | Females | 74.86 (-10.53 to 237.82) | 48.37 (-15.66 to 179.18) | 0 (0 to 0) |
| Bhutan | 65-69 years | Both sexes | 45.75 (-6.37 to 140.9) | 48.74 (-18.97 to 205.33) | 0 (0 to 0) |
| Bhutan | 65-69 years | Males | NA | 50.13 (-21.65 to 195.52) | 0 (0 to 0) |
| Bhutan | 65-69 years | Females | 92.71 (-12.9 to 285.55) | 47.31 (-16.22 to 213.79) | 0 (0 to 0) |
| Bhutan | 70-74 years | Both sexes | 31.73 (-3.84 to 99.34) | 40.82 (-13.28 to 180.81) | 0 (0 to 0) |
| Bhutan | 70-74 years | Males | NA | 43.42 (-14.59 to 183.18) | 0 (0 to 0) |
| Bhutan | 70-74 years | Females | 64.6 (-7.82 to 202.23) | 38.12 (-11.92 to 168.18) | 0 (0 to 0) |
| Bhutan | 75-79 years | Both sexes | 20.8 (-1.84 to 74.07) | 36.45 (-11.7 to 147.47) | 0 (0 to 0) |
| Bhutan | 75-79 years | Males | NA | 39.26 (-13.4 to 156.02) | 0 (0 to 0) |
| Bhutan | 75-79 years | Females | 42.05 (-3.71 to 149.74) | 33.58 (-10.14 to 141.14) | 0 (0 to 0) |
| Bhutan | 80-84 years | Both sexes | 14.39 (-1.21 to 58.83) | 26.95 (-7.96 to 117.73) | 0 (0 to 0) |
| Bhutan | 80-84 years | Males | NA | 29.92 (-8.95 to 123.86) | 0 (0 to 0) |
| Bhutan | 80-84 years | Females | 28.63 (-2.4 to 117.05) | 24.01 (-7.02 to 113.91) | 0 (0 to 0) |
| Bhutan | 85-89 years | Both sexes | 14.47 (-1.32 to 58.93) | 24.62 (-8.04 to 110.38) | 0 (0 to 0) |
| Bhutan | 85-89 years | Males | NA | 27.68 (-9.34 to 114.91) | 0 (0 to 0) |
| Bhutan | 85-89 years | Females | 28.3 (-2.58 to 115.24) | 21.7 (-6.8 to 106.43) | 0 (0 to 0) |
| Bhutan | 90-94 years | Both sexes | 14.73 (-1.49 to 58.89) | 22.7 (-7.73 to 104.15) | 0 (0 to 0) |
| Bhutan | 90-94 years | Males | NA | 25.99 (-8.93 to 109.15) | 0 (0 to 0) |
| Bhutan | 90-94 years | Females | 28.42 (-2.88 to 113.64) | 19.64 (-6.31 to 98.05) | 0 (0 to 0) |
| Bhutan | 95+ years | Both sexes | 13.66 (-1.35 to 50.84) | 22.97 (-8.37 to 103.53) | 0 (0 to 0) |
| Bhutan | 95+ years | Males | NA | 26.07 (-9.33 to 114) | 0 (0 to 0) |
| Bhutan | 95+ years | Females | 29.01 (-2.87 to 107.92) | 19.48 (-6.21 to 91.78) | 0 (0 to 0) |
| Bolivia (Plurinational State of) | <5 years | Both sexes | NA | NA | 0 (0 to 0) |
| Bolivia (Plurinational State of) | <5 years | Males | NA | NA | 0 (0 to 0) |
| Bolivia (Plurinational State of) | <5 years | Females | NA | NA | 0 (0 to 0) |
| Bolivia (Plurinational State of) | 5-9 years | Both sexes | NA | NA | 6.33 (1.55 to 19.38) |
| Bolivia (Plurinational State of) | 5-9 years | Males | NA | NA | 8.08 (1.92 to 24.5) |
| Bolivia (Plurinational State of) | 5-9 years | Females | NA | NA | 4.5 (1.02 to 13.9) |
| Bolivia (Plurinational State of) | 10-14 years | Both sexes | NA | NA | 90.23 (32.47 to 191.16) |
| Bolivia (Plurinational State of) | 10-14 years | Males | NA | NA | 102.26 (35.45 to 212.39) |
| Bolivia (Plurinational State of) | 10-14 years | Females | NA | NA | 77.61 (24.12 to 179.04) |
| Bolivia (Plurinational State of) | 15-19 years | Both sexes | 121.49 (-14.11 to 392.65) | 90.29 (-30.52 to 355.74) | 103.7 (37.85 to 221.91) |
| Bolivia (Plurinational State of) | 15-19 years | Males | NA | 66.64 (-23.61 to 255.47) | 102.63 (39.03 to 214.77) |
| Bolivia (Plurinational State of) | 15-19 years | Females | 248.25 (-28.83 to 802.31) | 114.95 (-36.93 to 451.53) | 104.8 (32.73 to 230.33) |
| Bolivia (Plurinational State of) | 20-24 years | Both sexes | 245.03 (-46.6 to 640.01) | 108.86 (-30.12 to 431.35) | 102.7 (38.14 to 232.78) |
| Bolivia (Plurinational State of) | 20-24 years | Males | NA | 77.71 (-21.87 to 301.99) | 102.19 (39.15 to 226.03) |
| Bolivia (Plurinational State of) | 20-24 years | Females | 499.3 (-94.96 to 1304.16) | 141.19 (-39.3 to 560.43) | 103.24 (30.21 to 247.83) |
| Bolivia (Plurinational State of) | 25-29 years | Both sexes | 314.38 (-76.2 to 772.05) | 117.75 (-35.41 to 461.07) | 88.39 (24.19 to 197.31) |
| Bolivia (Plurinational State of) | 25-29 years | Males | NA | 84.13 (-24.63 to 326.7) | 92.46 (30.45 to 195.8) |
| Bolivia (Plurinational State of) | 25-29 years | Females | 632.51 (-153.3 to 1553.32) | 151.76 (-46.32 to 597.14) | 84.27 (13.34 to 209.68) |
| Bolivia (Plurinational State of) | 30-34 years | Both sexes | 328.19 (-88.69 to 814.83) | 128.09 (-41.94 to 524.78) | 63.21 (12.16 to 152.95) |
| Bolivia (Plurinational State of) | 30-34 years | Males | NA | 92.22 (-30.43 to 354.78) | 72.54 (16.11 to 160.28) |
| Bolivia (Plurinational State of) | 30-34 years | Females | 651.88 (-176.17 to 1618.5) | 163.46 (-53.32 to 687.48) | 54.01 (0 to 150.03) |
| Bolivia (Plurinational State of) | 35-39 years | Both sexes | 326.98 (-86.61 to 743.72) | 125.4 (-45.21 to 496.73) | 28.12 (0.17 to 74.59) |
| Bolivia (Plurinational State of) | 35-39 years | Males | NA | 90.22 (-32.16 to 341.85) | 46.28 (0.34 to 112.01) |
| Bolivia (Plurinational State of) | 35-39 years | Females | 637.93 (-168.97 to 1450.99) | 158.85 (-58.38 to 633.55) | 10.84 (0 to 42.43) |
| Bolivia (Plurinational State of) | 40-44 years | Both sexes | 307.62 (-93.86 to 688.72) | 122.51 (-43.92 to 442.77) | 11.68 (0 to 41.58) |
| Bolivia (Plurinational State of) | 40-44 years | Males | NA | 90.05 (-31.31 to 327.53) | 23.74 (0 to 84.51) |
| Bolivia (Plurinational State of) | 40-44 years | Females | 605.58 (-184.78 to 1355.83) | 153.95 (-56.14 to 559.53) | 0 (0 to 0) |
| Bolivia (Plurinational State of) | 45-49 years | Both sexes | 299.71 (-68.52 to 667.31) | 117.85 (-44.96 to 421.77) | 1.14 (0 to 6.48) |
| Bolivia (Plurinational State of) | 45-49 years | Males | NA | 88.3 (-32.92 to 308.54) | 2.36 (0 to 13.46) |
| Bolivia (Plurinational State of) | 45-49 years | Females | 578.01 (-132.14 to 1286.95) | 145.29 (-56.7 to 526.22) | 0 (0 to 0) |
| Bolivia (Plurinational State of) | 50-54 years | Both sexes | 251.03 (-50.29 to 626.26) | 113.66 (-45.25 to 469.13) | 0 (0 to 0) |
| Bolivia (Plurinational State of) | 50-54 years | Males | NA | 87.54 (-33.88 to 346.52) | 0 (0 to 0) |
| Bolivia (Plurinational State of) | 50-54 years | Females | 493.41 (-98.85 to 1230.94) | 138.89 (-53.6 to 581.65) | 0 (0 to 0) |
| Bolivia (Plurinational State of) | 55-59 years | Both sexes | 223.75 (-38.65 to 588.49) | 107.89 (-46.23 to 434.95) | 0 (0 to 0) |
| Bolivia (Plurinational State of) | 55-59 years | Males | NA | 84.74 (-36.58 to 336.9) | 0 (0 to 0) |
| Bolivia (Plurinational State of) | 55-59 years | Females | 434.87 (-75.11 to 1143.73) | 129.73 (-55.34 to 527.45) | 0 (0 to 0) |
| Bolivia (Plurinational State of) | 60-64 years | Both sexes | 183.77 (-29.57 to 505.89) | 102.44 (-43.87 to 420.02) | 0 (0 to 0) |
| Bolivia (Plurinational State of) | 60-64 years | Males | NA | 82.38 (-34.59 to 339.76) | 0 (0 to 0) |
| Bolivia (Plurinational State of) | 60-64 years | Females | 352 (-56.63 to 968.98) | 120.81 (-52.36 to 488.2) | 0 (0 to 0) |
| Bolivia (Plurinational State of) | 65-69 years | Both sexes | 138.36 (-20.5 to 423.3) | 95.95 (-38 to 404.41) | 0 (0 to 0) |
| Bolivia (Plurinational State of) | 65-69 years | Males | NA | 79.11 (-30.52 to 333.35) | 0 (0 to 0) |
| Bolivia (Plurinational State of) | 65-69 years | Females | 260.74 (-38.63 to 797.69) | 110.84 (-44.61 to 467.71) | 0 (0 to 0) |
| Bolivia (Plurinational State of) | 70-74 years | Both sexes | 131.93 (-17.3 to 398.25) | 79.53 (-24.25 to 349.58) | 0 (0 to 0) |
| Bolivia (Plurinational State of) | 70-74 years | Males | NA | 66.22 (-20.59 to 279.07) | 0 (0 to 0) |
| Bolivia (Plurinational State of) | 70-74 years | Females | 241.73 (-31.7 to 729.67) | 90.61 (-27.35 to 405.64) | 0 (0 to 0) |
| Bolivia (Plurinational State of) | 75-79 years | Both sexes | 125.43 (-15.31 to 398.37) | 69.82 (-20.54 to 292.02) | 0 (0 to 0) |
| Bolivia (Plurinational State of) | 75-79 years | Males | NA | 58.46 (-17.36 to 243.41) | 0 (0 to 0) |
| Bolivia (Plurinational State of) | 75-79 years | Females | 223.15 (-27.23 to 708.74) | 78.66 (-22.37 to 329.7) | 0 (0 to 0) |
| Bolivia (Plurinational State of) | 80-84 years | Both sexes | 95.43 (-10.59 to 338.19) | 50.5 (-13.97 to 214.02) | 0 (0 to 0) |
| Bolivia (Plurinational State of) | 80-84 years | Males | NA | 42.39 (-11.65 to 180.18) | 0 (0 to 0) |
| Bolivia (Plurinational State of) | 80-84 years | Females | 160.2 (-17.78 to 567.71) | 56 (-15.06 to 240.4) | 0 (0 to 0) |
| Bolivia (Plurinational State of) | 85-89 years | Both sexes | 103.03 (-11.85 to 354.23) | 45.45 (-13.34 to 195.11) | 0 (0 to 0) |
| Bolivia (Plurinational State of) | 85-89 years | Males | NA | 37.93 (-11.32 to 156.47) | 0 (0 to 0) |
| Bolivia (Plurinational State of) | 85-89 years | Females | 155.98 (-17.93 to 536.29) | 49.32 (-14.45 to 213.4) | 0 (0 to 0) |
| Bolivia (Plurinational State of) | 90-94 years | Both sexes | 117.03 (-14.87 to 396.95) | 42.27 (-12.85 to 187.51) | 0 (0 to 0) |
| Bolivia (Plurinational State of) | 90-94 years | Males | NA | 34.81 (-10.54 to 150.39) | 0 (0 to 0) |
| Bolivia (Plurinational State of) | 90-94 years | Females | 152.55 (-19.39 to 517.41) | 44.54 (-13.62 to 199.92) | 0 (0 to 0) |
| Bolivia (Plurinational State of) | 95+ years | Both sexes | 129.94 (-15.79 to 467.57) | 41.88 (-14.41 to 174.9) | 0 (0 to 0) |
| Bolivia (Plurinational State of) | 95+ years | Males | NA | 34.07 (-11.47 to 134.62) | 0 (0 to 0) |
| Bolivia (Plurinational State of) | 95+ years | Females | 154.53 (-18.77 to 556.07) | 43.35 (-15 to 181.37) | 0 (0 to 0) |
| Bosnia and Herzegovina | <5 years | Both sexes | NA | NA | 0 (0 to 0) |
| Bosnia and Herzegovina | <5 years | Males | NA | NA | 0 (0 to 0) |
| Bosnia and Herzegovina | <5 years | Females | NA | NA | 0 (0 to 0) |
| Bosnia and Herzegovina | 5-9 years | Both sexes | NA | NA | 13.29 (3.23 to 39.42) |
| Bosnia and Herzegovina | 5-9 years | Males | NA | NA | 12.27 (2.9 to 35.74) |
| Bosnia and Herzegovina | 5-9 years | Females | NA | NA | 14.37 (3.06 to 44.88) |
| Bosnia and Herzegovina | 10-14 years | Both sexes | NA | NA | 171.91 (58.4 to 374.57) |
| Bosnia and Herzegovina | 10-14 years | Males | NA | NA | 155.02 (54.05 to 330.11) |
| Bosnia and Herzegovina | 10-14 years | Females | NA | NA | 189.64 (58.24 to 422.6) |
| Bosnia and Herzegovina | 15-19 years | Both sexes | 19.88 (-1.7 to 79.6) | 104.92 (-42.95 to 380.16) | 149.7 (56.38 to 319.68) |
| Bosnia and Herzegovina | 15-19 years | Males | NA | 84.74 (-29.96 to 293.18) | 138.27 (53.5 to 284.34) |
| Bosnia and Herzegovina | 15-19 years | Females | 40.66 (-3.47 to 162.74) | 126.01 (-46.72 to 462.73) | 161.64 (52.17 to 367.76) |
| Bosnia and Herzegovina | 20-24 years | Both sexes | 35.06 (-4.22 to 129.98) | 85.92 (-28.64 to 316.13) | 107.72 (40.56 to 228.6) |
| Bosnia and Herzegovina | 20-24 years | Males | NA | 73.42 (-25.34 to 244.98) | 103.05 (39.06 to 213.26) |
| Bosnia and Herzegovina | 20-24 years | Females | 71.56 (-8.61 to 265.32) | 98.93 (-33.77 to 377.5) | 112.59 (34.36 to 262.42) |
| Bosnia and Herzegovina | 25-29 years | Both sexes | 62.72 (-8.2 to 177.8) | 78.27 (-26.27 to 286.55) | 80.36 (21.85 to 172.46) |
| Bosnia and Herzegovina | 25-29 years | Males | NA | 64.6 (-22.15 to 225.12) | 78.88 (27.37 to 153.86) |
| Bosnia and Herzegovina | 25-29 years | Females | 128.05 (-16.74 to 363.01) | 92.51 (-30.73 to 360.67) | 81.89 (12.8 to 195.45) |
| Bosnia and Herzegovina | 30-34 years | Both sexes | 61.11 (-8.4 to 183.92) | 79.19 (-27.57 to 309.33) | 52.87 (8.53 to 124.31) |
| Bosnia and Herzegovina | 30-34 years | Males | NA | 64.04 (-24.28 to 231.59) | 56.55 (12.23 to 122.89) |
| Bosnia and Herzegovina | 30-34 years | Females | 123.28 (-16.95 to 371.02) | 94.6 (-31.6 to 384.59) | 49.13 (0 to 129.77) |
| Bosnia and Herzegovina | 35-39 years | Both sexes | 59.78 (-8.38 to 161.97) | 74.72 (-28.62 to 287.44) | 24.36 (0.13 to 61.52) |
| Bosnia and Herzegovina | 35-39 years | Males | NA | 59.29 (-24.58 to 205.2) | 37.26 (0.19 to 87.46) |
| Bosnia and Herzegovina | 35-39 years | Females | 121.74 (-17.06 to 329.87) | 90.72 (-32.6 to 357.09) | 10.99 (0 to 44.77) |
| Bosnia and Herzegovina | 40-44 years | Both sexes | 61.11 (-7.56 to 180.76) | 74.13 (-27.82 to 257.98) | 9.26 (0 to 33.44) |
| Bosnia and Herzegovina | 40-44 years | Males | NA | 58.26 (-23.36 to 187.82) | 18.2 (0 to 65.72) |
| Bosnia and Herzegovina | 40-44 years | Females | 124.41 (-15.39 to 367.98) | 90.56 (-33.6 to 315.32) | 0 (0 to 0) |
| Bosnia and Herzegovina | 45-49 years | Both sexes | 61.05 (-7.39 to 176.51) | 72.7 (-28.3 to 257.39) | 0.72 (0 to 4.77) |
| Bosnia and Herzegovina | 45-49 years | Males | NA | 55.72 (-23.05 to 191.63) | 1.44 (0 to 9.56) |
| Bosnia and Herzegovina | 45-49 years | Females | 121.8 (-14.74 to 352.11) | 89.59 (-35.64 to 324.12) | 0 (0 to 0) |
| Bosnia and Herzegovina | 50-54 years | Both sexes | 68.65 (-7.51 to 213.75) | 73.83 (-30.65 to 292.6) | 0 (0 to 0) |
| Bosnia and Herzegovina | 50-54 years | Males | NA | 57.16 (-24.72 to 211.22) | 0 (0 to 0) |
| Bosnia and Herzegovina | 50-54 years | Females | 136.36 (-14.92 to 424.54) | 90.26 (-37.62 to 372.72) | 0 (0 to 0) |
| Bosnia and Herzegovina | 55-59 years | Both sexes | 62.02 (-6.33 to 193.41) | 73.59 (-31.46 to 277.76) | 0 (0 to 0) |
| Bosnia and Herzegovina | 55-59 years | Males | NA | 56.9 (-26.8 to 205.57) | 0 (0 to 0) |
| Bosnia and Herzegovina | 55-59 years | Females | 120.78 (-12.32 to 376.65) | 89.4 (-36.24 to 350.65) | 0 (0 to 0) |
| Bosnia and Herzegovina | 60-64 years | Both sexes | 93.93 (-12.57 to 280.9) | 73.13 (-31.41 to 278.11) | 0 (0 to 0) |
| Bosnia and Herzegovina | 60-64 years | Males | NA | 57.24 (-24.84 to 213.45) | 0 (0 to 0) |
| Bosnia and Herzegovina | 60-64 years | Females | 178.52 (-23.89 to 533.87) | 87.45 (-37.33 to 349.59) | 0 (0 to 0) |
| Bosnia and Herzegovina | 65-69 years | Both sexes | 64.57 (-7.61 to 206.45) | 70.27 (-28.37 to 287.33) | 0 (0 to 0) |
| Bosnia and Herzegovina | 65-69 years | Males | NA | 55.87 (-22.47 to 219.32) | 0 (0 to 0) |
| Bosnia and Herzegovina | 65-69 years | Females | 118.84 (-14.01 to 379.96) | 82.36 (-33.33 to 348.15) | 0 (0 to 0) |
| Bosnia and Herzegovina | 70-74 years | Both sexes | 67.49 (-7.95 to 225.53) | 59.99 (-19.24 to 263.74) | 0 (0 to 0) |
| Bosnia and Herzegovina | 70-74 years | Males | NA | 48.35 (-15.61 to 209.42) | 0 (0 to 0) |
| Bosnia and Herzegovina | 70-74 years | Females | 119.1 (-14.03 to 398) | 68.88 (-21.71 to 300.74) | 0 (0 to 0) |
| Bosnia and Herzegovina | 75-79 years | Both sexes | 34.82 (-2.89 to 128.81) | 53.8 (-16.33 to 218.12) | 0 (0 to 0) |
| Bosnia and Herzegovina | 75-79 years | Males | NA | 43.85 (-14.31 to 161.88) | 0 (0 to 0) |
| Bosnia and Herzegovina | 75-79 years | Females | 58.82 (-4.88 to 217.6) | 60.66 (-17.62 to 255.71) | 0 (0 to 0) |
| Bosnia and Herzegovina | 80-84 years | Both sexes | 24.48 (-2.02 to 102.4) | 39.53 (-11.37 to 156.99) | 0 (0 to 0) |
| Bosnia and Herzegovina | 80-84 years | Males | NA | 33.21 (-9.49 to 125.9) | 0 (0 to 0) |
| Bosnia and Herzegovina | 80-84 years | Females | 39.21 (-3.24 to 164) | 43.33 (-12.51 to 178.77) | 0 (0 to 0) |
| Bosnia and Herzegovina | 85-89 years | Both sexes | 23.88 (-2.02 to 100.46) | 35.48 (-10.06 to 145.86) | 0 (0 to 0) |
| Bosnia and Herzegovina | 85-89 years | Males | NA | 30.47 (-8.7 to 116.29) | 0 (0 to 0) |
| Bosnia and Herzegovina | 85-89 years | Females | 37.92 (-3.2 to 159.49) | 38.43 (-10.91 to 163.92) | 0 (0 to 0) |
| Bosnia and Herzegovina | 90-94 years | Both sexes | 24.48 (-2.06 to 101.56) | 33.13 (-9.85 to 146.22) | 0 (0 to 0) |
| Bosnia and Herzegovina | 90-94 years | Males | NA | 28.84 (-9.08 to 116.3) | 0 (0 to 0) |
| Bosnia and Herzegovina | 90-94 years | Females | 38.01 (-3.2 to 157.69) | 35.5 (-10.28 to 160.86) | 0 (0 to 0) |
| Bosnia and Herzegovina | 95+ years | Both sexes | 26.01 (-2.53 to 110.79) | 32.48 (-11.21 to 134.15) | 0 (0 to 0) |
| Bosnia and Herzegovina | 95+ years | Males | NA | 28.92 (-9.94 to 117.57) | 0 (0 to 0) |
| Bosnia and Herzegovina | 95+ years | Females | 38.24 (-3.71 to 162.88) | 34.15 (-11.87 to 141.31) | 0 (0 to 0) |
| Botswana | <5 years | Both sexes | NA | NA | 0 (0 to 0) |
| Botswana | <5 years | Males | NA | NA | 0 (0 to 0) |
| Botswana | <5 years | Females | NA | NA | 0 (0 to 0) |
| Botswana | 5-9 years | Both sexes | NA | NA | 8.84 (2.49 to 22.57) |
| Botswana | 5-9 years | Males | NA | NA | 11.07 (3.23 to 29.01) |
| Botswana | 5-9 years | Females | NA | NA | 6.59 (1.73 to 17.38) |
| Botswana | 10-14 years | Both sexes | NA | NA | 144.97 (52.27 to 337.52) |
| Botswana | 10-14 years | Males | NA | NA | 158.42 (58.44 to 369.57) |
| Botswana | 10-14 years | Females | NA | NA | 131.3 (40.64 to 323.15) |
| Botswana | 15-19 years | Both sexes | 68.21 (-7.33 to 238.17) | 95.68 (-44.03 to 337.3) | 143.63 (55.82 to 290.6) |
| Botswana | 15-19 years | Males | NA | 74.49 (-32.98 to 285.44) | 163.01 (64.38 to 329.49) |
| Botswana | 15-19 years | Females | 137.09 (-14.73 to 478.68) | 117.07 (-50.43 to 407.66) | 124.06 (41.41 to 265.11) |
| Botswana | 20-24 years | Both sexes | 110.85 (-19.34 to 337.57) | 79.85 (-35.22 to 273.7) | 123.39 (50.62 to 259.1) |
| Botswana | 20-24 years | Males | NA | 61.5 (-24.54 to 219.97) | 143.84 (63.17 to 286.76) |
| Botswana | 20-24 years | Females | 218.24 (-38.08 to 664.58) | 97.63 (-45.55 to 327.1) | 103.59 (34.91 to 234.94) |
| Botswana | 25-29 years | Both sexes | 122.57 (-19.85 to 297.65) | 74.33 (-26.06 to 258.43) | 93.7 (28.98 to 202.97) |
| Botswana | 25-29 years | Males | NA | 55.78 (-19.46 to 203.7) | 113.78 (39.62 to 228.4) |
| Botswana | 25-29 years | Females | 239.16 (-38.73 to 580.78) | 91.98 (-32.78 to 307.3) | 74.6 (14.44 to 190.33) |
| Botswana | 30-34 years | Both sexes | 121.06 (-31.24 to 309.81) | 92.9 (-41.79 to 285.94) | 65.92 (12.74 to 149.98) |
| Botswana | 30-34 years | Males | NA | 79.45 (-34 to 251.29) | 86.93 (21.58 to 183.59) |
| Botswana | 30-34 years | Females | 236.32 (-60.98 to 604.77) | 105.72 (-48.76 to 318.94) | 45.92 (0 to 124.46) |
| Botswana | 35-39 years | Both sexes | 119.07 (-29.37 to 285.22) | 90.26 (-43.89 to 277.9) | 35.15 (0.32 to 84.35) |
| Botswana | 35-39 years | Males | NA | 72.39 (-31.66 to 235.13) | 59.74 (0.65 to 133.64) |
| Botswana | 35-39 years | Females | 234.14 (-57.75 to 560.87) | 107.52 (-52.95 to 318.33) | 11.38 (0 to 44.44) |
| Botswana | 40-44 years | Both sexes | 113.81 (-24.81 to 287.7) | 94.09 (-49.64 to 273) | 14.82 (0 to 47.67) |
| Botswana | 40-44 years | Males | NA | 83.46 (-44.74 to 242.06) | 29.98 (0 to 96.42) |
| Botswana | 40-44 years | Females | 225.11 (-49.07 to 569.09) | 104.48 (-53.15 to 303.49) | 0 (0 to 0) |
| Botswana | 45-49 years | Both sexes | 113.82 (-22.18 to 280.17) | 90.37 (-50.24 to 275.81) | 1.1 (0 to 6.11) |
| Botswana | 45-49 years | Males | NA | 78.77 (-41.94 to 236.96) | 2.19 (0 to 12.16) |
| Botswana | 45-49 years | Females | 228.82 (-44.59 to 563.23) | 102.08 (-58.62 to 312.47) | 0 (0 to 0) |
| Botswana | 50-54 years | Both sexes | 103.9 (-20.74 to 266.2) | 91.8 (-51.54 to 318.16) | 0 (0 to 0) |
| Botswana | 50-54 years | Males | NA | 76.66 (-40.66 to 269.37) | 0 (0 to 0) |
| Botswana | 50-54 years | Females | 203.3 (-40.58 to 520.88) | 106.28 (-62.89 to 351.35) | 0 (0 to 0) |
| Botswana | 55-59 years | Both sexes | 109.78 (-25.14 to 280.52) | 91.42 (-52.87 to 302.76) | 0 (0 to 0) |
| Botswana | 55-59 years | Males | NA | 85.32 (-51.01 to 285.9) | 0 (0 to 0) |
| Botswana | 55-59 years | Females | 200.15 (-45.83 to 511.41) | 96.44 (-54.4 to 318.1) | 0 (0 to 0) |
| Botswana | 60-64 years | Both sexes | 95.48 (-19.01 to 295.18) | 90.96 (-54.97 to 292.84) | 0 (0 to 0) |
| Botswana | 60-64 years | Males | NA | 83.13 (-50.58 to 254.79) | 0 (0 to 0) |
| Botswana | 60-64 years | Females | 168.55 (-33.56 to 521.1) | 96.94 (-57.25 to 327.88) | 0 (0 to 0) |
| Botswana | 65-69 years | Both sexes | 73.87 (-12.33 to 218.38) | 88.33 (-44.37 to 288.52) | 0 (0 to 0) |
| Botswana | 65-69 years | Males | NA | 86.98 (-46.39 to 276.09) | 0 (0 to 0) |
| Botswana | 65-69 years | Females | 128.84 (-21.5 to 380.91) | 89.33 (-43.79 to 301.43) | 0 (0 to 0) |
| Botswana | 70-74 years | Both sexes | 68.24 (-9.03 to 210.22) | 72.26 (-26.93 to 272.6) | 0 (0 to 0) |
| Botswana | 70-74 years | Males | NA | 69.79 (-27.81 to 256.78) | 0 (0 to 0) |
| Botswana | 70-74 years | Females | 118.98 (-15.75 to 366.49) | 74.1 (-27.27 to 285.29) | 0 (0 to 0) |
| Botswana | 75-79 years | Both sexes | 57.73 (-6.81 to 194.25) | 61.32 (-21.64 to 214.48) | 0 (0 to 0) |
| Botswana | 75-79 years | Males | NA | 52.9 (-17.44 to 182.99) | 0 (0 to 0) |
| Botswana | 75-79 years | Females | 98.28 (-11.59 to 330.65) | 67.23 (-25.9 to 237.67) | 0 (0 to 0) |
| Botswana | 80-84 years | Both sexes | 46.26 (-4.62 to 173.84) | 45.33 (-14.09 to 170.07) | 0 (0 to 0) |
| Botswana | 80-84 years | Males | NA | 41.02 (-12.6 to 150.35) | 0 (0 to 0) |
| Botswana | 80-84 years | Females | 75.54 (-7.54 to 283.91) | 48.06 (-15.47 to 182.98) | 0 (0 to 0) |
| Botswana | 85-89 years | Both sexes | 47.08 (-5.41 to 174.18) | 41.63 (-14.63 to 163.64) | 0 (0 to 0) |
| Botswana | 85-89 years | Males | NA | 38.35 (-13.58 to 148.33) | 0 (0 to 0) |
| Botswana | 85-89 years | Females | 73.99 (-8.5 to 273.71) | 43.5 (-15.31 to 171.96) | 0 (0 to 0) |
| Botswana | 90-94 years | Both sexes | 49.24 (-6.42 to 174.38) | 38.93 (-14.31 to 162.67) | 0 (0 to 0) |
| Botswana | 90-94 years | Males | NA | 36.15 (-12.65 to 144.1) | 0 (0 to 0) |
| Botswana | 90-94 years | Females | 73.93 (-9.63 to 261.82) | 40.33 (-15.19 to 172.61) | 0 (0 to 0) |
| Botswana | 95+ years | Both sexes | 52.05 (-6.72 to 186.97) | 38.08 (-15.44 to 143.56) | 0 (0 to 0) |
| Botswana | 95+ years | Males | NA | 35.65 (-14.45 to 132.58) | 0 (0 to 0) |
| Botswana | 95+ years | Females | 75.29 (-9.72 to 270.44) | 39.16 (-15.95 to 154.58) | 0 (0 to 0) |
| Brazil | <5 years | Both sexes | NA | NA | 0 (0 to 0) |
| Brazil | <5 years | Males | NA | NA | 0 (0 to 0) |
| Brazil | <5 years | Females | NA | NA | 0 (0 to 0) |
| Brazil | 5-9 years | Both sexes | NA | NA | 6.98 (2.06 to 18.75) |
| Brazil | 5-9 years | Males | NA | NA | 8.7 (2.46 to 22.82) |
| Brazil | 5-9 years | Females | NA | NA | 5.19 (1.29 to 14.54) |
| Brazil | 10-14 years | Both sexes | NA | NA | 107.83 (43.52 to 221.32) |
| Brazil | 10-14 years | Males | NA | NA | 114.15 (45.96 to 229.44) |
| Brazil | 10-14 years | Females | NA | NA | 101.23 (36.47 to 202.32) |
| Brazil | 15-19 years | Both sexes | 63.01 (-6.34 to 236.26) | 131.26 (-55.68 to 426.21) | 130.95 (49.31 to 267.03) |
| Brazil | 15-19 years | Males | NA | 73.25 (-28.79 to 244.11) | 105.52 (42.22 to 218.44) |
| Brazil | 15-19 years | Females | 128.52 (-12.93 to 481.93) | 191.59 (-70.79 to 626.9) | 157.38 (56.46 to 345.2) |
| Brazil | 20-24 years | Both sexes | 150.93 (-24.11 to 433.83) | 144.47 (-51.78 to 417.33) | 140.95 (51.64 to 301.36) |
| Brazil | 20-24 years | Males | NA | 70.27 (-25.69 to 256.01) | 118.18 (48.37 to 236.01) |
| Brazil | 20-24 years | Females | 303.81 (-48.53 to 873.28) | 219.63 (-73.8 to 653.69) | 164.02 (47.05 to 373.15) |
| Brazil | 25-29 years | Both sexes | 157.41 (-18.07 to 455.68) | 170.73 (-63 to 519.21) | 126.64 (32.26 to 277.6) |
| Brazil | 25-29 years | Males | NA | 78.9 (-28.13 to 283.03) | 111.76 (37.99 to 214.15) |
| Brazil | 25-29 years | Females | 311.09 (-35.71 to 900.58) | 260.4 (-92.6 to 773.56) | 141.17 (20.14 to 338.96) |
| Brazil | 30-34 years | Both sexes | 204.08 (-29.06 to 570.03) | 184.35 (-69.9 to 600.89) | 88.08 (14.14 to 218.81) |
| Brazil | 30-34 years | Males | NA | 87.96 (-33.97 to 322.7) | 87.09 (19.86 to 187.84) |
| Brazil | 30-34 years | Females | 398.34 (-56.71 to 1112.65) | 276.11 (-92.16 to 841.67) | 89.03 (0 to 248.5) |
| Brazil | 35-39 years | Both sexes | 173.69 (-19.81 to 521.23) | 195.39 (-85.3 to 581.34) | 32.15 (0.36 to 86.36) |
| Brazil | 35-39 years | Males | NA | 83.9 (-31.46 to 304.23) | 51.94 (0.18 to 122.08) |
| Brazil | 35-39 years | Females | 336.93 (-38.43 to 1011.11) | 300.18 (-128.24 to 860.14) | 13.56 (0 to 56.41) |
| Brazil | 40-44 years | Both sexes | 173.72 (-19.54 to 512.59) | 167.64 (-67.55 to 521.33) | 14.59 (0 to 48.26) |
| Brazil | 40-44 years | Males | NA | 86.64 (-34.62 to 288.26) | 30.12 (0 to 99.6) |
| Brazil | 40-44 years | Females | 336.99 (-37.9 to 994.39) | 243.78 (-88.88 to 715.6) | 0 (0 to 0) |
| Brazil | 45-49 years | Both sexes | 173.38 (-18.3 to 516.93) | 157.19 (-58.29 to 481.55) | 1.25 (0 to 7.49) |
| Brazil | 45-49 years | Males | NA | 81.04 (-32.29 to 288.07) | 2.61 (0 to 15.57) |
| Brazil | 45-49 years | Females | 334.01 (-35.25 to 995.83) | 227.75 (-79.77 to 682.13) | 0 (0 to 0) |
| Brazil | 50-54 years | Both sexes | 212.38 (-28.87 to 625.9) | 148.68 (-57.86 to 511.5) | 0 (0 to 0) |
| Brazil | 50-54 years | Males | NA | 79.62 (-31.46 to 298.1) | 0 (0 to 0) |
| Brazil | 50-54 years | Females | 406.66 (-55.29 to 1198.45) | 211.86 (-80.84 to 687.11) | 0 (0 to 0) |
| Brazil | 55-59 years | Both sexes | 168.43 (-19.21 to 507.14) | 135.18 (-52.87 to 476.72) | 0 (0 to 0) |
| Brazil | 55-59 years | Males | NA | 87.17 (-36.28 to 329.75) | 0 (0 to 0) |
| Brazil | 55-59 years | Females | 317.46 (-36.2 to 955.86) | 177.66 (-65.24 to 582.53) | 0 (0 to 0) |
| Brazil | 60-64 years | Both sexes | 153.33 (-13.92 to 463.24) | 113.61 (-53.06 to 408.13) | 0 (0 to 0) |
| Brazil | 60-64 years | Males | NA | 71.68 (-32.45 to 274.95) | 0 (0 to 0) |
| Brazil | 60-64 years | Females | 285.83 (-25.94 to 863.55) | 149.84 (-69.31 to 526.38) | 0 (0 to 0) |
| Brazil | 65-69 years | Both sexes | 137.31 (-19.27 to 435.35) | 100.55 (-41.16 to 370.11) | 0 (0 to 0) |
| Brazil | 65-69 years | Males | NA | 70.9 (-27.65 to 275.91) | 0 (0 to 0) |
| Brazil | 65-69 years | Females | 252.29 (-35.41 to 799.89) | 125.37 (-54.01 to 451.48) | 0 (0 to 0) |
| Brazil | 70-74 years | Both sexes | 127.28 (-16.71 to 383.83) | 95.38 (-33.38 to 379.84) | 0 (0 to 0) |
| Brazil | 70-74 years | Males | NA | 80.87 (-27.14 to 331.46) | 0 (0 to 0) |
| Brazil | 70-74 years | Females | 230.35 (-30.24 to 694.68) | 107.14 (-38.43 to 417.73) | 0 (0 to 0) |
| Brazil | 75-79 years | Both sexes | 83.05 (-10.9 to 276.44) | 76.88 (-27.04 to 298.22) | 0 (0 to 0) |
| Brazil | 75-79 years | Males | NA | 54.36 (-16.86 to 208.76) | 0 (0 to 0) |
| Brazil | 75-79 years | Females | 145.94 (-19.15 to 485.77) | 93.93 (-35.04 to 369.48) | 0 (0 to 0) |
| Brazil | 80-84 years | Both sexes | 42.61 (-4.04 to 166.91) | 51.78 (-15.42 to 224.66) | 0 (0 to 0) |
| Brazil | 80-84 years | Males | NA | 37.91 (-10.57 to 159.39) | 0 (0 to 0) |
| Brazil | 80-84 years | Females | 72.08 (-6.84 to 282.36) | 61.38 (-18.88 to 270.88) | 0 (0 to 0) |
| Brazil | 85-89 years | Both sexes | 42.75 (-4.29 to 165.96) | 46.95 (-14.93 to 201.56) | 0 (0 to 0) |
| Brazil | 85-89 years | Males | NA | 33.81 (-9.74 to 143.24) | 0 (0 to 0) |
| Brazil | 85-89 years | Females | 68.39 (-6.86 to 265.49) | 54.84 (-17.65 to 238.75) | 0 (0 to 0) |
| Brazil | 90-94 years | Both sexes | 44.13 (-4.84 to 172.4) | 43.7 (-14.29 to 199.81) | 0 (0 to 0) |
| Brazil | 90-94 years | Males | NA | 30.97 (-9.62 to 139.48) | 0 (0 to 0) |
| Brazil | 90-94 years | Females | 66.18 (-7.26 to 258.52) | 50.06 (-16.68 to 231.42) | 0 (0 to 0) |
| Brazil | 95+ years | Both sexes | 43.46 (-4.36 to 182.45) | 41.74 (-15.47 to 172.31) | 0 (0 to 0) |
| Brazil | 95+ years | Males | NA | 29.85 (-10.14 to 119.65) | 0 (0 to 0) |
| Brazil | 95+ years | Females | 66.23 (-6.64 to 278.04) | 47.97 (-18.26 to 202.25) | 0 (0 to 0) |
| Brunei Darussalam | <5 years | Both sexes | NA | NA | 0 (0 to 0) |
| Brunei Darussalam | <5 years | Males | NA | NA | 0 (0 to 0) |
| Brunei Darussalam | <5 years | Females | NA | NA | 0 (0 to 0) |
| Brunei Darussalam | 5-9 years | Both sexes | NA | NA | 20.34 (4.83 to 60.97) |
| Brunei Darussalam | 5-9 years | Males | NA | NA | 26.18 (6.01 to 75.38) |
| Brunei Darussalam | 5-9 years | Females | NA | NA | 13.94 (2.86 to 45.99) |
| Brunei Darussalam | 10-14 years | Both sexes | NA | NA | 117.84 (40.07 to 247.19) |
| Brunei Darussalam | 10-14 years | Males | NA | NA | 145.33 (49.82 to 294.66) |
| Brunei Darussalam | 10-14 years | Females | NA | NA | 88.2 (26.5 to 201.75) |
| Brunei Darussalam | 15-19 years | Both sexes | 25.24 (-2.08 to 99.94) | 81.59 (-30.07 to 315.11) | 120.67 (40.59 to 253.47) |
| Brunei Darussalam | 15-19 years | Males | NA | 54.51 (-18.02 to 214.84) | 139.8 (49.86 to 268.52) |
| Brunei Darussalam | 15-19 years | Females | 51.78 (-4.27 to 205.07) | 110.08 (-40.97 to 427.5) | 100.56 (31.39 to 240.96) |
| Brunei Darussalam | 20-24 years | Both sexes | 56.39 (-7.62 to 194.89) | 75.71 (-23.82 to 269.95) | 93.07 (34.97 to 200.5) |
| Brunei Darussalam | 20-24 years | Males | NA | 46.66 (-13.22 to 181.49) | 103.35 (37.41 to 219.35) |
| Brunei Darussalam | 20-24 years | Females | 120.15 (-16.23 to 415.31) | 108.57 (-35.8 to 366.61) | 81.44 (24.98 to 191.91) |
| Brunei Darussalam | 25-29 years | Both sexes | 70.56 (-10.61 to 222.5) | 67.52 (-21.02 to 243.02) | 65.92 (19.13 to 140.09) |
| Brunei Darussalam | 25-29 years | Males | NA | 40.19 (-10.77 to 160.91) | 73.67 (25.8 to 151.1) |
| Brunei Darussalam | 25-29 years | Females | 157.63 (-23.69 to 497.08) | 101.25 (-35.15 to 346.71) | 56.34 (8.82 to 136.88) |
| Brunei Darussalam | 30-34 years | Both sexes | 69.88 (-12.92 to 225.63) | 64.85 (-22.81 to 243.95) | 42.71 (9.01 to 96.24) |
| Brunei Darussalam | 30-34 years | Males | NA | 38.79 (-12.48 to 158.38) | 50.87 (11.87 to 105.02) |
| Brunei Darussalam | 30-34 years | Females | 159.38 (-29.46 to 514.62) | 98.24 (-37.5 to 348.48) | 32.27 (0 to 85.85) |
| Brunei Darussalam | 35-39 years | Both sexes | 65.44 (-11.4 to 205.77) | 60.23 (-22.24 to 212.56) | 21.81 (0.04 to 56.23) |
| Brunei Darussalam | 35-39 years | Males | NA | 35.63 (-11.54 to 142.95) | 33.7 (0.07 to 82.5) |
| Brunei Darussalam | 35-39 years | Females | 148.66 (-25.89 to 467.44) | 91.51 (-36.34 to 305.73) | 6.68 (0 to 27.46) |
| Brunei Darussalam | 40-44 years | Both sexes | 60.95 (-10.52 to 174.79) | 56.89 (-22.17 to 189.8) | 8.57 (0 to 30.16) |
| Brunei Darussalam | 40-44 years | Males | NA | 33.65 (-11.49 to 117.81) | 15.56 (0 to 54.75) |
| Brunei Darussalam | 40-44 years | Females | 135.73 (-23.44 to 389.24) | 85.4 (-35.4 to 275.28) | 0 (0 to 0) |
| Brunei Darussalam | 45-49 years | Both sexes | 59.74 (-9.36 to 175) | 54.07 (-21.95 to 175.43) | 0.66 (0 to 4.1) |
| Brunei Darussalam | 45-49 years | Males | NA | 31.46 (-12.36 to 111.43) | 1.25 (0 to 7.74) |
| Brunei Darussalam | 45-49 years | Females | 126.88 (-19.88 to 371.66) | 79.48 (-32.85 to 254.88) | 0 (0 to 0) |
| Brunei Darussalam | 50-54 years | Both sexes | 51.94 (-8.01 to 164.42) | 53.2 (-24.54 to 199.76) | 0 (0 to 0) |
| Brunei Darussalam | 50-54 years | Males | NA | 30.87 (-12.35 to 115.24) | 0 (0 to 0) |
| Brunei Darussalam | 50-54 years | Females | 106.93 (-16.49 to 338.51) | 76.83 (-38.69 to 284.94) | 0 (0 to 0) |
| Brunei Darussalam | 55-59 years | Both sexes | 45.7 (-6.23 to 142.79) | 50.77 (-22.7 to 190.58) | 0 (0 to 0) |
| Brunei Darussalam | 55-59 years | Males | NA | 29.16 (-12.27 to 111.94) | 0 (0 to 0) |
| Brunei Darussalam | 55-59 years | Females | 93.18 (-12.69 to 291.12) | 73.22 (-33.39 to 267.12) | 0 (0 to 0) |
| Brunei Darussalam | 60-64 years | Both sexes | 35.3 (-5.06 to 126.07) | 49.75 (-25.22 to 192.8) | 0 (0 to 0) |
| Brunei Darussalam | 60-64 years | Males | NA | 28.62 (-12.37 to 114.93) | 0 (0 to 0) |
| Brunei Darussalam | 60-64 years | Females | 68.97 (-9.88 to 246.34) | 69.91 (-36.91 to 258.26) | 0 (0 to 0) |
| Brunei Darussalam | 65-69 years | Both sexes | 26.74 (-2.95 to 89.45) | 47.14 (-19.85 to 181.91) | 0 (0 to 0) |
| Brunei Darussalam | 65-69 years | Males | NA | 26.62 (-10.18 to 102.13) | 0 (0 to 0) |
| Brunei Darussalam | 65-69 years | Females | 51.02 (-5.64 to 170.63) | 65.77 (-28.64 to 252.44) | 0 (0 to 0) |
| Brunei Darussalam | 70-74 years | Both sexes | 23.45 (-2.18 to 88) | 38.97 (-13.49 to 156.24) | 0 (0 to 0) |
| Brunei Darussalam | 70-74 years | Males | NA | 22.03 (-6.99 to 92.34) | 0 (0 to 0) |
| Brunei Darussalam | 70-74 years | Females | 44.37 (-4.12 to 166.56) | 54.1 (-19.17 to 211.22) | 0 (0 to 0) |
| Brunei Darussalam | 75-79 years | Both sexes | 17.21 (-1.49 to 61.14) | 33.03 (-10.99 to 125.37) | 0 (0 to 0) |
| Brunei Darussalam | 75-79 years | Males | NA | 18.84 (-5.77 to 71.04) | 0 (0 to 0) |
| Brunei Darussalam | 75-79 years | Females | 32.86 (-2.84 to 116.73) | 45.94 (-15.94 to 170.69) | 0 (0 to 0) |
| Brunei Darussalam | 80-84 years | Both sexes | 12.02 (-0.94 to 47.85) | 24.37 (-7.51 to 97.04) | 0 (0 to 0) |
| Brunei Darussalam | 80-84 years | Males | NA | 13.54 (-3.73 to 54.28) | 0 (0 to 0) |
| Brunei Darussalam | 80-84 years | Females | 21.82 (-1.7 to 86.85) | 33.2 (-10.59 to 131.6) | 0 (0 to 0) |
| Brunei Darussalam | 85-89 years | Both sexes | 11.54 (-0.95 to 46.41) | 21.76 (-6.95 to 87.02) | 0 (0 to 0) |
| Brunei Darussalam | 85-89 years | Males | NA | 11.9 (-3.85 to 47.22) | 0 (0 to 0) |
| Brunei Darussalam | 85-89 years | Females | 20.31 (-1.67 to 81.68) | 29.25 (-9.34 to 115.78) | 0 (0 to 0) |
| Brunei Darussalam | 90-94 years | Both sexes | 10.71 (-0.93 to 42.57) | 19.17 (-6.47 to 79.78) | 0 (0 to 0) |
| Brunei Darussalam | 90-94 years | Males | NA | 11.1 (-3.76 to 48.37) | 0 (0 to 0) |
| Brunei Darussalam | 90-94 years | Females | 19.6 (-1.7 to 77.88) | 25.86 (-8.72 to 104.13) | 0 (0 to 0) |
| Brunei Darussalam | 95+ years | Both sexes | 12.94 (-1.16 to 51.53) | 20.18 (-7.57 to 79.97) | 0 (0 to 0) |
| Brunei Darussalam | 95+ years | Males | NA | 11.01 (-3.75 to 45.88) | 0 (0 to 0) |
| Brunei Darussalam | 95+ years | Females | 19.7 (-1.76 to 78.49) | 24.97 (-9.77 to 98.62) | 0 (0 to 0) |
| Bulgaria | <5 years | Both sexes | NA | NA | 0 (0 to 0) |
| Bulgaria | <5 years | Males | NA | NA | 0 (0 to 0) |
| Bulgaria | <5 years | Females | NA | NA | 0 (0 to 0) |
| Bulgaria | 5-9 years | Both sexes | NA | NA | 6.87 (1.6 to 19.53) |
| Bulgaria | 5-9 years | Males | NA | NA | 6.9 (1.74 to 18.26) |
| Bulgaria | 5-9 years | Females | NA | NA | 6.84 (1.52 to 20.83) |
| Bulgaria | 10-14 years | Both sexes | NA | NA | 96.62 (31.7 to 215.4) |
| Bulgaria | 10-14 years | Males | NA | NA | 92.95 (30.62 to 209.38) |
| Bulgaria | 10-14 years | Females | NA | NA | 100.55 (30.61 to 235.07) |
| Bulgaria | 15-19 years | Both sexes | 28.93 (-2.56 to 110.45) | 60.94 (-25.16 to 211.59) | 89.95 (32.73 to 182.39) |
| Bulgaria | 15-19 years | Males | NA | 33.18 (-12.92 to 127.09) | 88.46 (34.62 to 179.64) |
| Bulgaria | 15-19 years | Females | 60.13 (-5.33 to 229.56) | 90.88 (-39.52 to 309.61) | 91.56 (28.69 to 201.72) |
| Bulgaria | 20-24 years | Both sexes | 51.88 (-7.24 to 182.15) | 43.1 (-13.94 to 146.76) | 66.32 (23.6 to 152.56) |
| Bulgaria | 20-24 years | Males | NA | 34.09 (-11.27 to 128.89) | 67.58 (25.54 to 143.94) |
| Bulgaria | 20-24 years | Females | 107.38 (-14.99 to 377.03) | 52.75 (-16.53 to 178.96) | 64.98 (19.44 to 159.86) |
| Bulgaria | 25-29 years | Both sexes | 85.61 (-17.39 to 251.08) | 41.87 (-14.32 to 139.79) | 47.65 (13.33 to 107.32) |
| Bulgaria | 25-29 years | Males | NA | 31.48 (-10.78 to 121.58) | 50.79 (17.27 to 104.73) |
| Bulgaria | 25-29 years | Females | 177.57 (-36.06 to 520.78) | 53.03 (-17.7 to 188.69) | 44.28 (7.2 to 110) |
| Bulgaria | 30-34 years | Both sexes | 96.88 (-15.36 to 249.74) | 42.11 (-13.47 to 144.37) | 33.77 (5.85 to 76.94) |
| Bulgaria | 30-34 years | Males | NA | 31.77 (-10.37 to 129.46) | 38.93 (9.82 to 85.81) |
| Bulgaria | 30-34 years | Females | 201.55 (-31.95 to 519.55) | 53.28 (-14.7 to 180.26) | 28.21 (0 to 77.65) |
| Bulgaria | 35-39 years | Both sexes | 96.74 (-14.45 to 246.45) | 43.35 (-15.22 to 142) | 16.8 (0.09 to 42.68) |
| Bulgaria | 35-39 years | Males | NA | 30.51 (-11.88 to 120.51) | 26.87 (0.13 to 66.4) |
| Bulgaria | 35-39 years | Females | 201.6 (-30.11 to 513.56) | 57.27 (-18.41 to 187.83) | 5.89 (0 to 24.75) |
| Bulgaria | 40-44 years | Both sexes | 96.76 (-13.77 to 234.38) | 42.68 (-15.08 to 132.17) | 6.05 (0 to 21.28) |
| Bulgaria | 40-44 years | Males | NA | 29.93 (-9.9 to 111.23) | 11.61 (0 to 40.84) |
| Bulgaria | 40-44 years | Females | 202 (-28.75 to 489.27) | 56.54 (-19.36 to 182.45) | 0 (0 to 0) |
| Bulgaria | 45-49 years | Both sexes | 98.4 (-14.99 to 260.98) | 41.8 (-16 to 138.02) | 0.45 (0 to 3.22) |
| Bulgaria | 45-49 years | Males | NA | 29.07 (-11.59 to 105.93) | 0.88 (0 to 6.27) |
| Bulgaria | 45-49 years | Females | 202.27 (-30.81 to 536.46) | 55.24 (-18.81 to 187.39) | 0 (0 to 0) |
| Bulgaria | 50-54 years | Both sexes | 85.81 (-14.03 to 255.88) | 43.96 (-16.86 to 160.79) | 0 (0 to 0) |
| Bulgaria | 50-54 years | Males | NA | 29.75 (-11.91 to 118.18) | 0 (0 to 0) |
| Bulgaria | 50-54 years | Females | 174.87 (-28.59 to 521.46) | 58.7 (-19.74 to 183.42) | 0 (0 to 0) |
| Bulgaria | 55-59 years | Both sexes | 80.68 (-12.34 to 252.16) | 41 (-15.93 to 150.27) | 0 (0 to 0) |
| Bulgaria | 55-59 years | Males | NA | 29.25 (-11.19 to 115.25) | 0 (0 to 0) |
| Bulgaria | 55-59 years | Females | 159.67 (-24.42 to 499.05) | 52.52 (-18.18 to 186.75) | 0 (0 to 0) |
| Bulgaria | 60-64 years | Both sexes | 64.27 (-9.25 to 227.23) | 41.56 (-14.48 to 156.54) | 0 (0 to 0) |
| Bulgaria | 60-64 years | Males | NA | 29.74 (-11.53 to 121.73) | 0 (0 to 0) |
| Bulgaria | 60-64 years | Females | 122.18 (-17.59 to 431.99) | 52.21 (-17.15 to 197.42) | 0 (0 to 0) |
| Bulgaria | 65-69 years | Both sexes | 50.89 (-6.09 to 179.9) | 38.85 (-12.8 to 144.69) | 0 (0 to 0) |
| Bulgaria | 65-69 years | Males | NA | 28.59 (-10.82 to 124.26) | 0 (0 to 0) |
| Bulgaria | 65-69 years | Females | 91.8 (-10.98 to 324.49) | 47.1 (-12.42 to 171.12) | 0 (0 to 0) |
| Bulgaria | 70-74 years | Both sexes | 49.77 (-5.56 to 155.25) | 31.36 (-10.08 to 130.94) | 0 (0 to 0) |
| Bulgaria | 70-74 years | Males | NA | 24.57 (-7.27 to 114.16) | 0 (0 to 0) |
| Bulgaria | 70-74 years | Females | 84.89 (-9.48 to 264.8) | 36.15 (-13.07 to 150.73) | 0 (0 to 0) |
| Bulgaria | 75-79 years | Both sexes | 43.04 (-4.19 to 156.69) | 33.05 (-9.24 to 134.1) | 0 (0 to 0) |
| Bulgaria | 75-79 years | Males | NA | 22.15 (-6.21 to 93.94) | 0 (0 to 0) |
| Bulgaria | 75-79 years | Females | 69.36 (-6.75 to 252.51) | 39.72 (-10.75 to 165.07) | 0 (0 to 0) |
| Bulgaria | 80-84 years | Both sexes | 30.1 (-2.51 to 119.31) | 23.69 (-6.75 to 112.03) | 0 (0 to 0) |
| Bulgaria | 80-84 years | Males | NA | 16.29 (-4.74 to 69.72) | 0 (0 to 0) |
| Bulgaria | 80-84 years | Females | 47.7 (-3.98 to 189.1) | 28.02 (-7.76 to 135.28) | 0 (0 to 0) |
| Bulgaria | 85-89 years | Both sexes | 30.31 (-2.88 to 120.89) | 21.04 (-6.69 to 90.98) | 0 (0 to 0) |
| Bulgaria | 85-89 years | Males | NA | 14.68 (-4.11 to 61.63) | 0 (0 to 0) |
| Bulgaria | 85-89 years | Females | 45.51 (-4.33 to 181.52) | 24.24 (-7.72 to 105.25) | 0 (0 to 0) |
| Bulgaria | 90-94 years | Both sexes | 31.39 (-3.14 to 125) | 19.47 (-6.37 to 86.1) | 0 (0 to 0) |
| Bulgaria | 90-94 years | Males | NA | 13.49 (-4.33 to 63.09) | 0 (0 to 0) |
| Bulgaria | 90-94 years | Females | 44.77 (-4.47 to 178.24) | 22.01 (-7.26 to 94.97) | 0 (0 to 0) |
| Bulgaria | 95+ years | Both sexes | 32.62 (-3.09 to 125.46) | 19.1 (-6.63 to 86.29) | 0 (0 to 0) |
| Bulgaria | 95+ years | Males | NA | 13.43 (-4.57 to 58.59) | 0 (0 to 0) |
| Bulgaria | 95+ years | Females | 45.37 (-4.3 to 174.5) | 21.32 (-7.3 to 95.96) | 0 (0 to 0) |
| Burkina Faso | <5 years | Both sexes | NA | NA | 0 (0 to 0) |
| Burkina Faso | <5 years | Males | NA | NA | 0 (0 to 0) |
| Burkina Faso | <5 years | Females | NA | NA | 0 (0 to 0) |
| Burkina Faso | 5-9 years | Both sexes | NA | NA | 10.06 (2.92 to 24.32) |
| Burkina Faso | 5-9 years | Males | NA | NA | 11.59 (3.48 to 29.04) |
| Burkina Faso | 5-9 years | Females | NA | NA | 8.52 (2.21 to 20.96) |
| Burkina Faso | 10-14 years | Both sexes | NA | NA | 204.66 (73.94 to 470.86) |
| Burkina Faso | 10-14 years | Males | NA | NA | 184.91 (63.01 to 399.19) |
| Burkina Faso | 10-14 years | Females | NA | NA | 224.07 (69.26 to 512.72) |
| Burkina Faso | 15-19 years | Both sexes | 62.88 (-6.34 to 256.52) | 142.87 (-60.21 to 460.89) | 170.52 (67.6 to 348.17) |
| Burkina Faso | 15-19 years | Males | NA | 63.36 (-22.77 to 250.97) | 164.86 (63.74 to 341.13) |
| Burkina Faso | 15-19 years | Females | 121.11 (-12.21 to 494.08) | 216.51 (-102.95 to 695.86) | 175.76 (55.37 to 379.2) |
| Burkina Faso | 20-24 years | Both sexes | 104.73 (-13.69 to 310.87) | 128.99 (-48.97 to 479.57) | 109.08 (38.82 to 226.94) |
| Burkina Faso | 20-24 years | Males | NA | 62.73 (-17.91 to 237.76) | 112.22 (44.88 to 238.07) |
| Burkina Faso | 20-24 years | Females | 192.49 (-25.17 to 571.39) | 184.52 (-74.5 to 690.62) | 106.44 (29.09 to 247.19) |
| Burkina Faso | 25-29 years | Both sexes | 133.85 (-21.4 to 360.6) | 113.18 (-43.65 to 403.03) | 60.08 (16.12 to 130.79) |
| Burkina Faso | 25-29 years | Males | NA | 58.23 (-17.84 to 221.63) | 70.41 (23.16 to 149.11) |
| Burkina Faso | 25-29 years | Females | 239.34 (-38.26 to 644.76) | 156.49 (-63.99 to 548.81) | 51.93 (7.43 to 129.49) |
| Burkina Faso | 30-34 years | Both sexes | 131.68 (-23.39 to 341.42) | 98.19 (-36.99 to 367.3) | 28.19 (4.82 to 67.74) |
| Burkina Faso | 30-34 years | Males | NA | 58.01 (-18.39 to 238.5) | 39.29 (7.69 to 87.03) |
| Burkina Faso | 30-34 years | Females | 241.93 (-42.98 to 627.28) | 131.83 (-53.09 to 477.71) | 18.91 (0 to 56.68) |
| Burkina Faso | 35-39 years | Both sexes | 118.56 (-23.02 to 302.52) | 85.25 (-31.41 to 316.07) | 11.41 (0.01 to 31.01) |
| Burkina Faso | 35-39 years | Males | NA | 55.86 (-20.06 to 223.05) | 21.37 (0.03 to 54.38) |
| Burkina Faso | 35-39 years | Females | 225.37 (-43.76 to 575.04) | 111.73 (-41.71 to 397.17) | 2.45 (0 to 10.2) |
| Burkina Faso | 40-44 years | Both sexes | 99.18 (-20.69 to 248.18) | 82.02 (-31.5 to 280.11) | 4.65 (0 to 17.58) |
| Burkina Faso | 40-44 years | Males | NA | 56.84 (-19.39 to 204.77) | 9.61 (0 to 36.37) |
| Burkina Faso | 40-44 years | Females | 191.95 (-40.05 to 480.35) | 105.57 (-44.44 to 357.27) | 0 (0 to 0) |
| Burkina Faso | 45-49 years | Both sexes | 96.23 (-17.31 to 268.25) | 80.09 (-31.63 to 281.28) | 0.35 (0 to 2.21) |
| Burkina Faso | 45-49 years | Males | NA | 56.58 (-20.51 to 207.81) | 0.71 (0 to 4.49) |
| Burkina Faso | 45-49 years | Females | 189.77 (-34.15 to 529.03) | 102.95 (-43.11 to 361.69) | 0 (0 to 0) |
| Burkina Faso | 50-54 years | Both sexes | 78.12 (-10.06 to 267.89) | 80.37 (-34.02 to 316.39) | 0 (0 to 0) |
| Burkina Faso | 50-54 years | Males | NA | 57.66 (-22.48 to 230.72) | 0 (0 to 0) |
| Burkina Faso | 50-54 years | Females | 153.32 (-19.75 to 525.77) | 102.23 (-45.24 to 394.56) | 0 (0 to 0) |
| Burkina Faso | 55-59 years | Both sexes | 72.17 (-9.2 to 241.55) | 79.3 (-37.59 to 307.33) | 0 (0 to 0) |
| Burkina Faso | 55-59 years | Males | NA | 56.88 (-23.67 to 230.02) | 0 (0 to 0) |
| Burkina Faso | 55-59 years | Females | 139.67 (-17.8 to 467.47) | 100.26 (-48.19 to 379.64) | 0 (0 to 0) |
| Burkina Faso | 60-64 years | Both sexes | 55.16 (-7.09 to 206.53) | 78.32 (-34.52 to 292.58) | 0 (0 to 0) |
| Burkina Faso | 60-64 years | Males | NA | 56.27 (-24.23 to 223.25) | 0 (0 to 0) |
| Burkina Faso | 60-64 years | Females | 106.57 (-13.69 to 399.01) | 98.88 (-44.11 to 357.62) | 0 (0 to 0) |
| Burkina Faso | 65-69 years | Both sexes | 41.86 (-4.35 to 156.25) | 75.87 (-32.23 to 306.85) | 0 (0 to 0) |
| Burkina Faso | 65-69 years | Males | NA | 54.01 (-21.22 to 215.2) | 0 (0 to 0) |
| Burkina Faso | 65-69 years | Females | 80.72 (-8.4 to 301.28) | 96.16 (-42.44 to 390.03) | 0 (0 to 0) |
| Burkina Faso | 70-74 years | Both sexes | 37.66 (-3.39 to 133.12) | 65.29 (-21.99 to 286.87) | 0 (0 to 0) |
| Burkina Faso | 70-74 years | Males | NA | 45.96 (-14.42 to 207.96) | 0 (0 to 0) |
| Burkina Faso | 70-74 years | Females | 71.78 (-6.47 to 253.73) | 82.8 (-27.98 to 356.88) | 0 (0 to 0) |
| Burkina Faso | 75-79 years | Both sexes | 29.88 (-2.48 to 108.85) | 58.49 (-18.66 to 232.13) | 0 (0 to 0) |
| Burkina Faso | 75-79 years | Males | NA | 40.71 (-11.28 to 162.41) | 0 (0 to 0) |
| Burkina Faso | 75-79 years | Females | 55.39 (-4.6 to 201.79) | 73.66 (-24 to 286.75) | 0 (0 to 0) |
| Burkina Faso | 80-84 years | Both sexes | 20.81 (-1.74 to 85.2) | 43.85 (-12.51 to 181.89) | 0 (0 to 0) |
| Burkina Faso | 80-84 years | Males | NA | 29.76 (-7.95 to 127.74) | 0 (0 to 0) |
| Burkina Faso | 80-84 years | Females | 37.49 (-3.14 to 153.45) | 55.13 (-16.02 to 222.64) | 0 (0 to 0) |
| Burkina Faso | 85-89 years | Both sexes | 20.97 (-1.89 to 90.36) | 40.08 (-12.74 to 163.41) | 0 (0 to 0) |
| Burkina Faso | 85-89 years | Males | NA | 26.68 (-7.78 to 112.9) | 0 (0 to 0) |
| Burkina Faso | 85-89 years | Females | 36.58 (-3.3 to 157.6) | 50.05 (-16.53 to 203.99) | 0 (0 to 0) |
| Burkina Faso | 90-94 years | Both sexes | 21.53 (-1.92 to 93.3) | 37.46 (-12.39 to 165.22) | 0 (0 to 0) |
| Burkina Faso | 90-94 years | Males | NA | 24.65 (-7.55 to 108.21) | 0 (0 to 0) |
| Burkina Faso | 90-94 years | Females | 36.51 (-3.26 to 158.21) | 46.37 (-16.58 to 204.87) | 0 (0 to 0) |
| Burkina Faso | 95+ years | Both sexes | 22.88 (-2.1 to 98.05) | 37.33 (-14.26 to 149.81) | 0 (0 to 0) |
| Burkina Faso | 95+ years | Males | NA | 24.43 (-8.44 to 99.06) | 0 (0 to 0) |
| Burkina Faso | 95+ years | Females | 37.11 (-3.4 to 159.01) | 45.35 (-17.88 to 180.32) | 0 (0 to 0) |
| Burundi | <5 years | Both sexes | NA | NA | 0 (0 to 0) |
| Burundi | <5 years | Males | NA | NA | 0 (0 to 0) |
| Burundi | <5 years | Females | NA | NA | 0 (0 to 0) |
| Burundi | 5-9 years | Both sexes | NA | NA | 4.8 (1.48 to 12.08) |
| Burundi | 5-9 years | Males | NA | NA | 5.73 (1.75 to 13.9) |
| Burundi | 5-9 years | Females | NA | NA | 3.88 (0.94 to 10.83) |
| Burundi | 10-14 years | Both sexes | NA | NA | 77.57 (31.39 to 160.28) |
| Burundi | 10-14 years | Males | NA | NA | 87.9 (36.14 to 176.25) |
| Burundi | 10-14 years | Females | NA | NA | 67.44 (24.06 to 148.8) |
| Burundi | 15-19 years | Both sexes | 47.51 (-5.3 to 169.95) | 70.45 (-26.12 to 271.3) | 78.96 (30.34 to 171.56) |
| Burundi | 15-19 years | Males | NA | 57.67 (-18.68 to 237.43) | 95 (37.23 to 199.01) |
| Burundi | 15-19 years | Females | 93.19 (-10.4 to 333.31) | 82.73 (-33.67 to 301.93) | 63.54 (21.76 to 153.28) |
| Burundi | 20-24 years | Both sexes | 100.24 (-20.68 to 273.07) | 65.28 (-21.45 to 230.47) | 56.99 (20.76 to 128.58) |
| Burundi | 20-24 years | Males | NA | 50.58 (-15.26 to 193.52) | 69.71 (25.36 to 155.83) |
| Burundi | 20-24 years | Females | 194.96 (-40.22 to 531.12) | 79.18 (-27.3 to 270.65) | 44.96 (13.17 to 109.45) |
| Burundi | 25-29 years | Both sexes | 122.86 (-25.56 to 319.78) | 61.62 (-19.84 to 225.6) | 38.85 (11.04 to 83.56) |
| Burundi | 25-29 years | Males | NA | 47.47 (-14.33 to 180.8) | 50.09 (15.91 to 103.51) |
| Burundi | 25-29 years | Females | 240.75 (-50.1 to 626.62) | 75.2 (-25.25 to 267.18) | 28.06 (4.24 to 68.35) |
| Burundi | 30-34 years | Both sexes | 126.71 (-31.74 to 303.03) | 62.28 (-23.28 to 240.76) | 24.15 (4.79 to 60.49) |
| Burundi | 30-34 years | Males | NA | 48.03 (-16.99 to 194.48) | 33.55 (7.53 to 79.29) |
| Burundi | 30-34 years | Females | 254.22 (-63.68 to 607.97) | 76.62 (-29.25 to 277.23) | 14.69 (0 to 45.67) |
| Burundi | 35-39 years | Both sexes | 126.6 (-32.38 to 273.71) | 59.08 (-21.34 to 223.93) | 11.35 (0.07 to 28.63) |
| Burundi | 35-39 years | Males | NA | 45.54 (-15.69 to 185.19) | 19.59 (0.14 to 46.04) |
| Burundi | 35-39 years | Females | 259.92 (-66.49 to 561.96) | 73.34 (-27.28 to 264.8) | 2.68 (0 to 10.01) |
| Burundi | 40-44 years | Both sexes | 119.65 (-29.16 to 267.52) | 57.4 (-21.84 to 202.62) | 4.57 (0 to 16.94) |
| Burundi | 40-44 years | Males | NA | 44.55 (-15.44 to 160.79) | 8.62 (0 to 31.93) |
| Burundi | 40-44 years | Females | 254.93 (-62.12 to 569.98) | 71.94 (-28.49 to 244.7) | 0 (0 to 0) |
| Burundi | 45-49 years | Both sexes | 120.76 (-26.02 to 276) | 56.13 (-21.74 to 200.99) | 0.31 (0 to 1.94) |
| Burundi | 45-49 years | Males | NA | 43.65 (-16.03 to 159.08) | 0.58 (0 to 3.63) |
| Burundi | 45-49 years | Females | 259.51 (-55.91 to 593.11) | 70.47 (-28.62 to 248.37) | 0 (0 to 0) |
| Burundi | 50-54 years | Both sexes | 103.29 (-22.11 to 274.22) | 57.17 (-23.47 to 226.87) | 0 (0 to 0) |
| Burundi | 50-54 years | Males | NA | 44.39 (-15.91 to 179.18) | 0 (0 to 0) |
| Burundi | 50-54 years | Females | 220.99 (-47.3 to 586.69) | 71.74 (-32.12 to 274.16) | 0 (0 to 0) |
| Burundi | 55-59 years | Both sexes | 98.74 (-20.67 to 272.01) | 56.3 (-25.97 to 219.33) | 0 (0 to 0) |
| Burundi | 55-59 years | Males | NA | 43.85 (-18.48 to 169.5) | 0 (0 to 0) |
| Burundi | 55-59 years | Females | 210.48 (-44.07 to 579.83) | 70.38 (-33.95 to 278.46) | 0 (0 to 0) |
| Burundi | 60-64 years | Both sexes | 83.25 (-16.04 to 239.95) | 55.54 (-24.19 to 223.83) | 0 (0 to 0) |
| Burundi | 60-64 years | Males | NA | 43.35 (-17.39 to 180.77) | 0 (0 to 0) |
| Burundi | 60-64 years | Females | 179.12 (-34.51 to 516.27) | 69.59 (-32.03 to 270.44) | 0 (0 to 0) |
| Burundi | 65-69 years | Both sexes | 70.05 (-10.86 to 211.41) | 54.34 (-23.01 to 225.25) | 0 (0 to 0) |
| Burundi | 65-69 years | Males | NA | 42.19 (-15.05 to 189.96) | 0 (0 to 0) |
| Burundi | 65-69 years | Females | 149.32 (-23.15 to 450.65) | 68.1 (-31.62 to 273.49) | 0 (0 to 0) |
| Burundi | 70-74 years | Both sexes | 65.25 (-8.45 to 202.31) | 46.3 (-14.27 to 216.48) | 0 (0 to 0) |
| Burundi | 70-74 years | Males | NA | 35.7 (-10.73 to 167.62) | 0 (0 to 0) |
| Burundi | 70-74 years | Females | 137.78 (-17.84 to 427.18) | 58.07 (-19.27 to 268.53) | 0 (0 to 0) |
| Burundi | 75-79 years | Both sexes | 56.21 (-7.16 to 184.94) | 41.56 (-12.62 to 161.56) | 0 (0 to 0) |
| Burundi | 75-79 years | Males | NA | 31.62 (-9.01 to 130.17) | 0 (0 to 0) |
| Burundi | 75-79 years | Females | 112.72 (-14.36 to 370.87) | 51.56 (-16.26 to 199.66) | 0 (0 to 0) |
| Burundi | 80-84 years | Both sexes | 45.32 (-5.13 to 166.03) | 31.36 (-8.75 to 121.9) | 0 (0 to 0) |
| Burundi | 80-84 years | Males | NA | 22.91 (-5.77 to 92.88) | 0 (0 to 0) |
| Burundi | 80-84 years | Females | 82.76 (-9.36 to 303.19) | 38.34 (-11.06 to 148.06) | 0 (0 to 0) |
| Burundi | 85-89 years | Both sexes | 47.31 (-5.17 to 172.57) | 28.68 (-8.56 to 117) | 0 (0 to 0) |
| Burundi | 85-89 years | Males | NA | 20.69 (-6.15 to 87.77) | 0 (0 to 0) |
| Burundi | 85-89 years | Females | 80.22 (-8.77 to 292.61) | 34.24 (-10.26 to 139.96) | 0 (0 to 0) |
| Burundi | 90-94 years | Both sexes | 50 (-6.09 to 183.91) | 26.89 (-8.65 to 113.5) | 0 (0 to 0) |
| Burundi | 90-94 years | Males | NA | 18.99 (-5.88 to 81.17) | 0 (0 to 0) |
| Burundi | 90-94 years | Females | 80.28 (-9.78 to 295.27) | 31.68 (-10.39 to 132.95) | 0 (0 to 0) |
| Burundi | 95+ years | Both sexes | 58.57 (-6.71 to 216.33) | 27.6 (-9.33 to 113.55) | 0 (0 to 0) |
| Burundi | 95+ years | Males | NA | 18.69 (-6.3 to 79.34) | 0 (0 to 0) |
| Burundi | 95+ years | Females | 81.74 (-9.36 to 301.91) | 31.12 (-10.55 to 127.46) | 0 (0 to 0) |
| Cabo Verde | <5 years | Both sexes | NA | NA | 0 (0 to 0) |
| Cabo Verde | <5 years | Males | NA | NA | 0 (0 to 0) |
| Cabo Verde | <5 years | Females | NA | NA | 0 (0 to 0) |
| Cabo Verde | 5-9 years | Both sexes | NA | NA | 12.89 (3.81 to 32.95) |
| Cabo Verde | 5-9 years | Males | NA | NA | 14.49 (4.3 to 40) |
| Cabo Verde | 5-9 years | Females | NA | NA | 11.23 (2.85 to 27.65) |
| Cabo Verde | 10-14 years | Both sexes | NA | NA | 259.81 (96.74 to 570.89) |
| Cabo Verde | 10-14 years | Males | NA | NA | 230.39 (79.96 to 500.47) |
| Cabo Verde | 10-14 years | Females | NA | NA | 290.9 (96.31 to 669.24) |
| Cabo Verde | 15-19 years | Both sexes | 65.14 (-6.21 to 264.07) | 139.83 (-59.11 to 509.82) | 212.67 (84.96 to 449.07) |
| Cabo Verde | 15-19 years | Males | NA | 102.96 (-41.07 to 394.53) | 199.72 (80 to 404.89) |
| Cabo Verde | 15-19 years | Females | 135.28 (-12.9 to 548.38) | 179.53 (-79.96 to 638.23) | 226.61 (72.04 to 487.21) |
| Cabo Verde | 20-24 years | Both sexes | 66.55 (-8.39 to 219.91) | 117.8 (-40.32 to 421.4) | 147.91 (56.19 to 300.56) |
| Cabo Verde | 20-24 years | Males | NA | 86.62 (-28.36 to 307.76) | 144.96 (60.78 to 290.68) |
| Cabo Verde | 20-24 years | Females | 138.88 (-17.5 to 458.89) | 151.69 (-53.63 to 548.13) | 151.11 (41.11 to 326.87) |
| Cabo Verde | 25-29 years | Both sexes | 84.14 (-10.75 to 259.2) | 103.18 (-35.31 to 382.88) | 105.27 (29.8 to 216.88) |
| Cabo Verde | 25-29 years | Males | NA | 78.18 (-25.67 to 280.56) | 110.52 (36.8 to 215) |
| Cabo Verde | 25-29 years | Females | 174.8 (-22.33 to 538.46) | 130.11 (-46.42 to 495.26) | 99.62 (14.49 to 224.74) |
| Cabo Verde | 30-34 years | Both sexes | 97.83 (-15.67 to 272.5) | 93.3 (-36.82 to 337.83) | 65.14 (12.04 to 151.86) |
| Cabo Verde | 30-34 years | Males | NA | 76.74 (-29.69 to 254.45) | 78.39 (17.68 to 162.59) |
| Cabo Verde | 30-34 years | Females | 200.03 (-32.03 to 557.16) | 110.6 (-44.28 to 407.14) | 51.3 (0 to 136.94) |
| Cabo Verde | 35-39 years | Both sexes | 88.38 (-16.43 to 261.22) | 82.49 (-33.48 to 299.28) | 29.96 (0.13 to 73.13) |
| Cabo Verde | 35-39 years | Males | NA | 71.84 (-26.75 to 252.48) | 50.04 (0.26 to 113.29) |
| Cabo Verde | 35-39 years | Females | 180.22 (-33.5 to 532.66) | 93.55 (-40.47 to 347.92) | 9.09 (0 to 36.75) |
| Cabo Verde | 40-44 years | Both sexes | 76.73 (-13.26 to 232.67) | 79.98 (-30.71 to 274.77) | 12.41 (0 to 43.39) |
| Cabo Verde | 40-44 years | Males | NA | 71.7 (-27.9 to 238.63) | 23.79 (0 to 83.2) |
| Cabo Verde | 40-44 years | Females | 160.36 (-27.71 to 486.29) | 89.01 (-33.82 to 314.16) | 0 (0 to 0) |
| Cabo Verde | 45-49 years | Both sexes | 73.14 (-11.01 to 216.61) | 77.64 (-31.88 to 267.13) | 0.59 (0 to 4.14) |
| Cabo Verde | 45-49 years | Males | NA | 70.86 (-29.89 to 238.38) | 1.13 (0 to 7.91) |
| Cabo Verde | 45-49 years | Females | 153.69 (-23.13 to 455.18) | 85.1 (-33.6 to 293.91) | 0 (0 to 0) |
| Cabo Verde | 50-54 years | Both sexes | 82.11 (-11.09 to 267.01) | 78.75 (-34.03 to 298.92) | 0 (0 to 0) |
| Cabo Verde | 50-54 years | Males | NA | 72.29 (-31.02 to 267.92) | 0 (0 to 0) |
| Cabo Verde | 50-54 years | Females | 167.04 (-22.57 to 543.21) | 85.43 (-36.74 to 325.02) | 0 (0 to 0) |
| Cabo Verde | 55-59 years | Both sexes | 77.5 (-11.11 to 252.69) | 76.95 (-35.02 to 288.4) | 0 (0 to 0) |
| Cabo Verde | 55-59 years | Males | NA | 70.46 (-32.98 to 257.12) | 0 (0 to 0) |
| Cabo Verde | 55-59 years | Females | 152.09 (-21.81 to 495.92) | 83.2 (-36.74 to 319.5) | 0 (0 to 0) |
| Cabo Verde | 60-64 years | Both sexes | 62.59 (-8.54 to 220.39) | 76.19 (-35.21 to 289.36) | 0 (0 to 0) |
| Cabo Verde | 60-64 years | Males | NA | 70 (-32.67 to 258.12) | 0 (0 to 0) |
| Cabo Verde | 60-64 years | Females | 116.59 (-15.9 to 410.52) | 81.52 (-37.4 to 319.73) | 0 (0 to 0) |
| Cabo Verde | 65-69 years | Both sexes | 49.97 (-5.33 to 174.16) | 73.95 (-31.96 to 286.92) | 0 (0 to 0) |
| Cabo Verde | 65-69 years | Males | NA | 67.85 (-29.88 to 254.51) | 0 (0 to 0) |
| Cabo Verde | 65-69 years | Females | 88.44 (-9.43 to 308.24) | 78.66 (-33.39 to 314.86) | 0 (0 to 0) |
| Cabo Verde | 70-74 years | Both sexes | 46.88 (-4.1 to 171.3) | 64.04 (-21.29 to 281.05) | 0 (0 to 0) |
| Cabo Verde | 70-74 years | Males | NA | 59.04 (-19.06 to 250.77) | 0 (0 to 0) |
| Cabo Verde | 70-74 years | Females | 80.49 (-7.03 to 294.14) | 67.63 (-22.8 to 295.52) | 0 (0 to 0) |
| Cabo Verde | 75-79 years | Both sexes | 37.93 (-3.17 to 143.07) | 57.21 (-18.26 to 223.98) | 0 (0 to 0) |
| Cabo Verde | 75-79 years | Males | NA | 53.1 (-16.26 to 198.45) | 0 (0 to 0) |
| Cabo Verde | 75-79 years | Females | 62.22 (-5.2 to 234.72) | 59.85 (-19.47 to 234.95) | 0 (0 to 0) |
| Cabo Verde | 80-84 years | Both sexes | 27.71 (-2.17 to 118.26) | 42.71 (-12.03 to 169.08) | 0 (0 to 0) |
| Cabo Verde | 80-84 years | Males | NA | 39.54 (-11.23 to 154.03) | 0 (0 to 0) |
| Cabo Verde | 80-84 years | Females | 42.53 (-3.33 to 181.51) | 44.41 (-12.5 to 179.66) | 0 (0 to 0) |
| Cabo Verde | 85-89 years | Both sexes | 28.33 (-2.46 to 112.64) | 39.03 (-11.83 to 160.96) | 0 (0 to 0) |
| Cabo Verde | 85-89 years | Males | NA | 36.5 (-11.24 to 149.76) | 0 (0 to 0) |
| Cabo Verde | 85-89 years | Females | 41.5 (-3.6 to 164.97) | 40.21 (-12.2 to 166.32) | 0 (0 to 0) |
| Cabo Verde | 90-94 years | Both sexes | 29.85 (-2.86 to 120.04) | 36.09 (-12.05 to 159.31) | 0 (0 to 0) |
| Cabo Verde | 90-94 years | Males | NA | 33.78 (-11.6 to 144.76) | 0 (0 to 0) |
| Cabo Verde | 90-94 years | Females | 41.58 (-3.99 to 167.24) | 37 (-12 to 166.19) | 0 (0 to 0) |
| Cabo Verde | 95+ years | Both sexes | 32.27 (-2.99 to 131.98) | 35.34 (-12.07 to 141.63) | 0 (0 to 0) |
| Cabo Verde | 95+ years | Males | NA | 33.51 (-12.5 to 132.07) | 0 (0 to 0) |
| Cabo Verde | 95+ years | Females | 42.34 (-3.93 to 173.16) | 35.92 (-11.94 to 145.11) | 0 (0 to 0) |
| Cambodia | <5 years | Both sexes | NA | NA | 0 (0 to 0) |
| Cambodia | <5 years | Males | NA | NA | 0 (0 to 0) |
| Cambodia | <5 years | Females | NA | NA | 0 (0 to 0) |
| Cambodia | 5-9 years | Both sexes | NA | NA | 2.06 (0.5 to 5.77) |
| Cambodia | 5-9 years | Males | NA | NA | 2.04 (0.53 to 5.86) |
| Cambodia | 5-9 years | Females | NA | NA | 2.08 (0.46 to 6.07) |
| Cambodia | 10-14 years | Both sexes | NA | NA | 35.01 (13.86 to 73.8) |
| Cambodia | 10-14 years | Males | NA | NA | 34.72 (13.16 to 69.37) |
| Cambodia | 10-14 years | Females | NA | NA | 35.32 (12.66 to 81.49) |
| Cambodia | 15-19 years | Both sexes | 11.59 (-1.09 to 52.73) | 34.73 (-12.59 to 123.45) | 30.05 (11.42 to 64.8) |
| Cambodia | 15-19 years | Males | NA | 32.42 (-14.66 to 112.9) | 28.53 (10.77 to 58.34) |
| Cambodia | 15-19 years | Females | 23.3 (-2.2 to 106.03) | 37.07 (-11.06 to 135.87) | 31.58 (10.28 to 71.86) |
| Cambodia | 20-24 years | Both sexes | 40.06 (-3.86 to 118) | 32.93 (-9.69 to 117.85) | 22.47 (7.75 to 49.71) |
| Cambodia | 20-24 years | Males | NA | 29.56 (-10.81 to 106.08) | 21.3 (7.61 to 46.99) |
| Cambodia | 20-24 years | Females | 78.76 (-7.58 to 232) | 36.18 (-9.92 to 126) | 23.59 (6.99 to 56.74) |
| Cambodia | 25-29 years | Both sexes | 45.08 (-6.2 to 130.72) | 32.87 (-10.89 to 110.27) | 15.88 (3.83 to 36.04) |
| Cambodia | 25-29 years | Males | NA | 28.32 (-9.04 to 97.52) | 15.53 (5.07 to 35.05) |
| Cambodia | 25-29 years | Females | 87.7 (-12.06 to 254.29) | 37.17 (-11.81 to 128.74) | 16.21 (2.26 to 42.62) |
| Cambodia | 30-34 years | Both sexes | 51.22 (-7.57 to 148.36) | 39.89 (-14.29 to 130.78) | 8.97 (1.08 to 23.72) |
| Cambodia | 30-34 years | Males | NA | 42.17 (-14.46 to 139.79) | 9.68 (1.83 to 25.92) |
| Cambodia | 30-34 years | Females | 100.09 (-14.8 to 289.91) | 37.72 (-11.45 to 134.31) | 8.29 (0 to 26.6) |
| Cambodia | 35-39 years | Both sexes | 54.97 (-7.78 to 146.76) | 39.97 (-15.61 to 129.9) | 2.93 (0 to 8.5) |
| Cambodia | 35-39 years | Males | NA | 39.78 (-15.78 to 132.3) | 5.23 (0 to 14.34) |
| Cambodia | 35-39 years | Females | 108.45 (-15.35 to 289.52) | 40.16 (-12.64 to 130.75) | 0.7 (0 to 3.36) |
| Cambodia | 40-44 years | Both sexes | 60.69 (-9.22 to 160.33) | 44.18 (-18.24 to 131.91) | 1.22 (0 to 5.01) |
| Cambodia | 40-44 years | Males | NA | 48.33 (-22.36 to 135.63) | 2.48 (0 to 10.23) |
| Cambodia | 40-44 years | Females | 118.96 (-18.06 to 314.26) | 40.19 (-11.91 to 128.5) | 0 (0 to 0) |
| Cambodia | 45-49 years | Both sexes | 64.11 (-9.18 to 174.8) | 41.96 (-16.67 to 134.49) | 0.03 (0 to 0.23) |
| Cambodia | 45-49 years | Males | NA | 46.82 (-22.52 to 142.95) | 0.05 (0 to 0.48) |
| Cambodia | 45-49 years | Females | 125.53 (-17.97 to 342.27) | 37.32 (-13.26 to 125.73) | 0 (0 to 0) |
| Cambodia | 50-54 years | Both sexes | 82.75 (-11.8 to 232.83) | 37.79 (-14.13 to 147.15) | 0 (0 to 0) |
| Cambodia | 50-54 years | Males | NA | 39.86 (-16.66 to 156.17) | 0 (0 to 0) |
| Cambodia | 50-54 years | Females | 157.68 (-22.48 to 443.68) | 35.92 (-12.78 to 133.93) | 0 (0 to 0) |
| Cambodia | 55-59 years | Both sexes | 92.17 (-12.8 to 246.04) | 37.91 (-14.74 to 142.28) | 0 (0 to 0) |
| Cambodia | 55-59 years | Males | NA | 39.98 (-17.91 to 144.06) | 0 (0 to 0) |
| Cambodia | 55-59 years | Females | 172.84 (-23.99 to 461.35) | 36.09 (-11.99 to 132.08) | 0 (0 to 0) |
| Cambodia | 60-64 years | Both sexes | 78.92 (-10.31 to 235.74) | 37.53 (-14.38 to 147.95) | 0 (0 to 0) |
| Cambodia | 60-64 years | Males | NA | 40.62 (-16.26 to 162.72) | 0 (0 to 0) |
| Cambodia | 60-64 years | Females | 141.53 (-18.49 to 422.74) | 35.07 (-11.17 to 134.12) | 0 (0 to 0) |
| Cambodia | 65-69 years | Both sexes | 33.12 (-3.46 to 116.14) | 37.92 (-13.47 to 142.95) | 0 (0 to 0) |
| Cambodia | 65-69 years | Males | NA | 40.27 (-15.32 to 167.57) | 0 (0 to 0) |
| Cambodia | 65-69 years | Females | 56.34 (-5.89 to 197.56) | 36.27 (-10.94 to 131.44) | 0 (0 to 0) |
| Cambodia | 70-74 years | Both sexes | 30.25 (-2.73 to 115.65) | 32.13 (-9.59 to 147.99) | 0 (0 to 0) |
| Cambodia | 70-74 years | Males | NA | 34.88 (-11.08 to 148.83) | 0 (0 to 0) |
| Cambodia | 70-74 years | Females | 49.89 (-4.51 to 190.74) | 30.35 (-8.61 to 137.29) | 0 (0 to 0) |
| Cambodia | 75-79 years | Both sexes | 22.89 (-2.44 to 83.77) | 27.96 (-8.41 to 113.7) | 0 (0 to 0) |
| Cambodia | 75-79 years | Males | NA | 31.71 (-10.4 to 125.05) | 0 (0 to 0) |
| Cambodia | 75-79 years | Females | 37.71 (-4.02 to 138.01) | 25.53 (-7.12 to 105.63) | 0 (0 to 0) |
| Cambodia | 80-84 years | Both sexes | 15.79 (-1.64 to 61.97) | 19.78 (-5.87 to 88.4) | 0 (0 to 0) |
| Cambodia | 80-84 years | Males | NA | 23.37 (-7.47 to 94.47) | 0 (0 to 0) |
| Cambodia | 80-84 years | Females | 25.66 (-2.67 to 100.69) | 17.55 (-4.65 to 82.52) | 0 (0 to 0) |
| Cambodia | 85-89 years | Both sexes | 15.48 (-1.61 to 65.87) | 17.77 (-5.13 to 75.48) | 0 (0 to 0) |
| Cambodia | 85-89 years | Males | NA | 21.73 (-6.74 to 88.86) | 0 (0 to 0) |
| Cambodia | 85-89 years | Females | 24.6 (-2.56 to 104.68) | 15.44 (-4.2 to 67.89) | 0 (0 to 0) |
| Cambodia | 90-94 years | Both sexes | 15.35 (-1.65 to 70.62) | 16.23 (-5.04 to 70.63) | 0 (0 to 0) |
| Cambodia | 90-94 years | Males | NA | 20.39 (-6.61 to 81.22) | 0 (0 to 0) |
| Cambodia | 90-94 years | Females | 24.17 (-2.59 to 111.2) | 13.84 (-4.02 to 62.39) | 0 (0 to 0) |
| Cambodia | 95+ years | Both sexes | 14.12 (-1.26 to 59.52) | 16.31 (-5.84 to 67.52) | 0 (0 to 0) |
| Cambodia | 95+ years | Males | NA | 20.04 (-7.31 to 83.13) | 0 (0 to 0) |
| Cambodia | 95+ years | Females | 24.44 (-2.18 to 103.06) | 13.59 (-4.49 to 56.39) | 0 (0 to 0) |
| Cameroon | <5 years | Both sexes | NA | NA | 0 (0 to 0) |
| Cameroon | <5 years | Males | NA | NA | 0 (0 to 0) |
| Cameroon | <5 years | Females | NA | NA | 0 (0 to 0) |
| Cameroon | 5-9 years | Both sexes | NA | NA | 13.18 (3.99 to 32.94) |
| Cameroon | 5-9 years | Males | NA | NA | 15.86 (5.04 to 42.48) |
| Cameroon | 5-9 years | Females | NA | NA | 10.4 (2.72 to 25.16) |
| Cameroon | 10-14 years | Both sexes | NA | NA | 235.5 (88.85 to 526.77) |
| Cameroon | 10-14 years | Males | NA | NA | 225.56 (75.29 to 492.55) |
| Cameroon | 10-14 years | Females | NA | NA | 245.74 (77.75 to 585.09) |
| Cameroon | 15-19 years | Both sexes | 113.41 (-12.53 to 377.3) | 118.57 (-46.16 to 451.04) | 200.08 (79.08 to 408.64) |
| Cameroon | 15-19 years | Males | NA | 113.05 (-45.68 to 413.35) | 199.92 (80.83 to 395.18) |
| Cameroon | 15-19 years | Females | 226.77 (-25.05 to 754.4) | 124.09 (-45.89 to 473.29) | 200.24 (63.52 to 436.41) |
| Cameroon | 20-24 years | Both sexes | 160.72 (-23.7 to 410.07) | 110.18 (-33.25 to 376.79) | 142.7 (53.66 to 292.92) |
| Cameroon | 20-24 years | Males | NA | 97.03 (-32.95 to 342.43) | 150.78 (58.87 to 310.38) |
| Cameroon | 20-24 years | Females | 315.3 (-46.5 to 804.46) | 122.83 (-41.62 to 433.54) | 134.94 (38.03 to 302.17) |
| Cameroon | 25-29 years | Both sexes | 184.1 (-37.26 to 443.12) | 88.46 (-25.91 to 303.13) | 99.87 (29.84 to 202.77) |
| Cameroon | 25-29 years | Males | NA | 86.9 (-28.53 to 300.36) | 114.1 (39.67 to 219.26) |
| Cameroon | 25-29 years | Females | 358.24 (-72.5 to 862.29) | 89.94 (-27.12 to 303.83) | 86.42 (12.72 to 203.36) |
| Cameroon | 30-34 years | Both sexes | 177.82 (-40.6 to 416.03) | 76.29 (-24.21 to 261.52) | 62.05 (11.36 to 141.31) |
| Cameroon | 30-34 years | Males | NA | 83.72 (-32.58 to 302.43) | 80.88 (18.79 to 168.16) |
| Cameroon | 30-34 years | Females | 347.3 (-79.29 to 812.53) | 69.21 (-21.34 to 254.32) | 44.1 (0 to 118.98) |
| Cameroon | 35-39 years | Both sexes | 162.29 (-35.28 to 363.8) | 70.6 (-24.89 to 241.21) | 29.75 (0.14 to 77.16) |
| Cameroon | 35-39 years | Males | NA | 78.69 (-29.52 to 271.26) | 52.63 (0.28 to 129.83) |
| Cameroon | 35-39 years | Females | 318.29 (-69.2 to 713.49) | 62.82 (-16.69 to 223.96) | 7.76 (0 to 31.89) |
| Cameroon | 40-44 years | Both sexes | 141.85 (-27.59 to 334.5) | 69.42 (-25.94 to 219.82) | 12.88 (0 to 44.39) |
| Cameroon | 40-44 years | Males | NA | 78.74 (-31.67 to 263.72) | 26 (0 to 89.61) |
| Cameroon | 40-44 years | Females | 281.08 (-54.66 to 662.8) | 60.28 (-19.06 to 205.17) | 0 (0 to 0) |
| Cameroon | 45-49 years | Both sexes | 149.69 (-30.4 to 352.09) | 66.51 (-26.51 to 217.72) | 1.1 (0 to 6.73) |
| Cameroon | 45-49 years | Males | NA | 77.51 (-31.9 to 257.05) | 2.17 (0 to 13.3) |
| Cameroon | 45-49 years | Females | 302.85 (-61.51 to 712.35) | 55.25 (-17.89 to 197.75) | 0 (0 to 0) |
| Cameroon | 50-54 years | Both sexes | 136.82 (-26.89 to 367.95) | 62.02 (-26.76 to 240.52) | 0 (0 to 0) |
| Cameroon | 50-54 years | Males | NA | 78.72 (-36.29 to 292.25) | 0 (0 to 0) |
| Cameroon | 50-54 years | Females | 277.58 (-54.55 to 746.49) | 44.84 (-15.49 to 185.89) | 0 (0 to 0) |
| Cameroon | 55-59 years | Both sexes | 130.59 (-25.73 to 375.91) | 63.21 (-26.54 to 239.31) | 0 (0 to 0) |
| Cameroon | 55-59 years | Males | NA | 77.51 (-34.63 to 284.52) | 0 (0 to 0) |
| Cameroon | 55-59 years | Females | 263.38 (-51.9 to 758.15) | 48.66 (-14.87 to 199.11) | 0 (0 to 0) |
| Cameroon | 60-64 years | Both sexes | 111.02 (-20.22 to 317.92) | 61.51 (-26.72 to 238.68) | 0 (0 to 0) |
| Cameroon | 60-64 years | Males | NA | 76.76 (-36.24 to 270.85) | 0 (0 to 0) |
| Cameroon | 60-64 years | Females | 222.91 (-40.6 to 638.3) | 46.16 (-15.72 to 181.92) | 0 (0 to 0) |
| Cameroon | 65-69 years | Both sexes | 93.22 (-14.71 to 287.87) | 62.49 (-25.66 to 263.5) | 0 (0 to 0) |
| Cameroon | 65-69 years | Males | NA | 73.94 (-31.93 to 288.86) | 0 (0 to 0) |
| Cameroon | 65-69 years | Females | 183.96 (-29.03 to 568.11) | 51.34 (-20.07 to 235.57) | 0 (0 to 0) |
| Cameroon | 70-74 years | Both sexes | 87.93 (-10.59 to 280.56) | 53.3 (-16.47 to 237.54) | 0 (0 to 0) |
| Cameroon | 70-74 years | Males | NA | 64.17 (-20.33 to 259.39) | 0 (0 to 0) |
| Cameroon | 70-74 years | Females | 170.07 (-20.48 to 542.64) | 43.15 (-12.81 to 197.95) | 0 (0 to 0) |
| Cameroon | 75-79 years | Both sexes | 71.72 (-8.12 to 237.76) | 47.5 (-13.84 to 193.22) | 0 (0 to 0) |
| Cameroon | 75-79 years | Males | NA | 58.03 (-17.43 to 205.29) | 0 (0 to 0) |
| Cameroon | 75-79 years | Females | 138.86 (-15.73 to 460.33) | 37.64 (-10.2 to 155.2) | 0 (0 to 0) |
| Cameroon | 80-84 years | Both sexes | 54.12 (-5.5 to 194.68) | 34.5 (-9.86 to 143.94) | 0 (0 to 0) |
| Cameroon | 80-84 years | Males | NA | 43.34 (-13.03 to 169.47) | 0 (0 to 0) |
| Cameroon | 80-84 years | Females | 102.1 (-10.38 to 367.26) | 26.66 (-6.96 to 121.17) | 0 (0 to 0) |
| Cameroon | 85-89 years | Both sexes | 53.04 (-5.61 to 181.82) | 31.03 (-9.65 to 125.95) | 0 (0 to 0) |
| Cameroon | 85-89 years | Males | NA | 39.28 (-13.14 to 149.28) | 0 (0 to 0) |
| Cameroon | 85-89 years | Females | 99.98 (-10.57 to 342.7) | 23.72 (-6.43 to 106.06) | 0 (0 to 0) |
| Cameroon | 90-94 years | Both sexes | 50.99 (-6.34 to 171.21) | 29.22 (-9.14 to 122.51) | 0 (0 to 0) |
| Cameroon | 90-94 years | Males | NA | 37.19 (-12.55 to 155.32) | 0 (0 to 0) |
| Cameroon | 90-94 years | Females | 99.18 (-12.34 to 333.04) | 21.68 (-5.91 to 91.49) | 0 (0 to 0) |
| Cameroon | 95+ years | Both sexes | 49.03 (-5.6 to 176.55) | 29.05 (-10.13 to 116.44) | 0 (0 to 0) |
| Cameroon | 95+ years | Males | NA | 36.35 (-13.52 to 139.8) | 0 (0 to 0) |
| Cameroon | 95+ years | Females | 101.11 (-11.54 to 364.12) | 21.31 (-6.6 to 90.49) | 0 (0 to 0) |
| Canada | <5 years | Both sexes | NA | NA | 0 (0 to 0) |
| Canada | <5 years | Males | NA | NA | 0 (0 to 0) |
| Canada | <5 years | Females | NA | NA | 0 (0 to 0) |
| Canada | 5-9 years | Both sexes | NA | NA | 24.6 (5.72 to 73.11) |
| Canada | 5-9 years | Males | NA | NA | 27.8 (6.79 to 82.7) |
| Canada | 5-9 years | Females | NA | NA | 21.23 (3.81 to 72.79) |
| Canada | 10-14 years | Both sexes | NA | NA | 130.79 (45.77 to 269.69) |
| Canada | 10-14 years | Males | NA | NA | 142.78 (51.93 to 287.57) |
| Canada | 10-14 years | Females | NA | NA | 118.07 (36.54 to 279.03) |
| Canada | 15-19 years | Both sexes | 37.22 (-4.25 to 149.89) | 94 (-34.85 to 355.28) | 135.48 (48.9 to 283.97) |
| Canada | 15-19 years | Males | NA | 46.45 (-16.97 to 189.97) | 136.79 (55.8 to 271.34) |
| Canada | 15-19 years | Females | 76.9 (-8.79 to 309.69) | 144.7 (-54.06 to 530.32) | 134.08 (42.61 to 307.44) |
| Canada | 20-24 years | Both sexes | 56.57 (-5.11 to 179.29) | 106.72 (-33.43 to 327.71) | 110.11 (37.59 to 245.94) |
| Canada | 20-24 years | Males | NA | 42.78 (-14.95 to 147.32) | 109.66 (41.63 to 231.75) |
| Canada | 20-24 years | Females | 117.26 (-10.6 to 371.64) | 175.32 (-59.04 to 538.98) | 110.59 (32.04 to 263.89) |
| Canada | 25-29 years | Both sexes | 73.15 (-6.61 to 233.98) | 111.62 (-40.98 to 341.2) | 85.52 (23.94 to 177.89) |
| Canada | 25-29 years | Males | NA | 42.97 (-15.26 to 153.76) | 89.46 (30.61 to 170.55) |
| Canada | 25-29 years | Females | 149.69 (-13.52 to 478.81) | 183.45 (-65.63 to 534.93) | 81.4 (11.93 to 203.41) |
| Canada | 30-34 years | Both sexes | 77.61 (-7.14 to 252.56) | 118.28 (-49.11 to 389.44) | 59.1 (10.14 to 136.53) |
| Canada | 30-34 years | Males | NA | 59.39 (-18.67 to 224.98) | 68.56 (15.72 to 142.76) |
| Canada | 30-34 years | Females | 155.32 (-14.28 to 505.41) | 177.25 (-68.71 to 561.51) | 49.62 (0 to 135.33) |
| Canada | 35-39 years | Both sexes | 75.49 (-6.02 to 240.71) | 113.3 (-46.67 to 356.25) | 26.96 (0.11 to 69.02) |
| Canada | 35-39 years | Males | NA | 50.21 (-19.07 to 168.39) | 45.43 (0 to 106.65) |
| Canada | 35-39 years | Females | 149.64 (-11.94 to 477.14) | 175.27 (-70.03 to 542.62) | 8.81 (0 to 38.12) |
| Canada | 40-44 years | Both sexes | 72.88 (-5.6 to 244.25) | 116.41 (-47.36 to 343.48) | 10.02 (0 to 35.21) |
| Canada | 40-44 years | Males | NA | 59.12 (-24.22 to 203.26) | 20.38 (0 to 71.61) |
| Canada | 40-44 years | Females | 143.4 (-11.02 to 480.59) | 171.85 (-67.13 to 485.27) | 0 (0 to 0) |
| Canada | 45-49 years | Both sexes | 67.11 (-6.12 to 213.89) | 111.09 (-41.02 to 331.67) | 0.62 (0 to 4.35) |
| Canada | 45-49 years | Males | NA | 56.75 (-20.9 to 176.43) | 1.26 (0 to 8.88) |
| Canada | 45-49 years | Females | 131.56 (-11.99 to 419.3) | 163.27 (-60.4 to 485.66) | 0 (0 to 0) |
| Canada | 50-54 years | Both sexes | 56.03 (-5.19 to 197.03) | 110.5 (-46.04 to 350.19) | 0 (0 to 0) |
| Canada | 50-54 years | Males | NA | 53.93 (-18.81 to 190.19) | 0 (0 to 0) |
| Canada | 50-54 years | Females | 110.09 (-10.19 to 387.09) | 165.07 (-71.9 to 520.41) | 0 (0 to 0) |
| Canada | 55-59 years | Both sexes | 48.93 (-5.05 to 162.95) | 106.27 (-43.08 to 352.43) | 0 (0 to 0) |
| Canada | 55-59 years | Males | NA | 59.51 (-23.57 to 211.21) | 0 (0 to 0) |
| Canada | 55-59 years | Females | 96 (-9.9 to 319.72) | 151.25 (-61.85 to 489.77) | 0 (0 to 0) |
| Canada | 60-64 years | Both sexes | 37.66 (-4.12 to 135.92) | 100.51 (-45.37 to 325.24) | 0 (0 to 0) |
| Canada | 60-64 years | Males | NA | 52.93 (-20.02 to 175.2) | 0 (0 to 0) |
| Canada | 60-64 years | Females | 73.5 (-8.03 to 265.29) | 145.8 (-69.61 to 469.84) | 0 (0 to 0) |
| Canada | 65-69 years | Both sexes | 30.12 (-3.84 to 129.45) | 93.26 (-38.93 to 311.05) | 0 (0 to 0) |
| Canada | 65-69 years | Males | NA | 51.82 (-18.41 to 181.6) | 0 (0 to 0) |
| Canada | 65-69 years | Females | 58.22 (-7.42 to 250.23) | 131.93 (-55.96 to 430.24) | 0 (0 to 0) |
| Canada | 70-74 years | Both sexes | 27.38 (-2.6 to 129.13) | 79.2 (-27.59 to 274.15) | 0 (0 to 0) |
| Canada | 70-74 years | Males | NA | 44.24 (-12.77 to 163.73) | 0 (0 to 0) |
| Canada | 70-74 years | Females | 52.23 (-4.95 to 246.29) | 110.92 (-38.84 to 373.02) | 0 (0 to 0) |
| Canada | 75-79 years | Both sexes | 22.77 (-2.7 to 106.63) | 61.65 (-23.76 to 214.86) | 0 (0 to 0) |
| Canada | 75-79 years | Males | NA | 34.96 (-12.19 to 121.44) | 0 (0 to 0) |
| Canada | 75-79 years | Females | 42.78 (-5.07 to 200.32) | 85.11 (-32.14 to 292.36) | 0 (0 to 0) |
| Canada | 80-84 years | Both sexes | 16.26 (-1.57 to 65.53) | 48.92 (-18.66 to 174.66) | 0 (0 to 0) |
| Canada | 80-84 years | Males | NA | 29.21 (-11.25 to 109.78) | 0 (0 to 0) |
| Canada | 80-84 years | Females | 29.36 (-2.84 to 118.3) | 64.79 (-24.62 to 229.8) | 0 (0 to 0) |
| Canada | 85-89 years | Both sexes | 16.29 (-1.59 to 68.49) | 40.26 (-13.74 to 154.67) | 0 (0 to 0) |
| Canada | 85-89 years | Males | NA | 22.02 (-7.38 to 85.18) | 0 (0 to 0) |
| Canada | 85-89 years | Females | 27.61 (-2.69 to 116.08) | 52.92 (-18.16 to 205.97) | 0 (0 to 0) |
| Canada | 90-94 years | Both sexes | 16.91 (-2 to 70.73) | 30.35 (-12.12 to 130.87) | 0 (0 to 0) |
| Canada | 90-94 years | Males | NA | 15.7 (-5.4 to 70.58) | 0 (0 to 0) |
| Canada | 90-94 years | Females | 26.18 (-3.1 to 109.49) | 38.37 (-15.8 to 164.16) | 0 (0 to 0) |
| Canada | 95+ years | Both sexes | 19.38 (-2.29 to 80.46) | 31.28 (-13.82 to 127.81) | 0 (0 to 0) |
| Canada | 95+ years | Males | NA | 15.34 (-6.19 to 64.49) | 0 (0 to 0) |
| Canada | 95+ years | Females | 26 (-3.08 to 107.92) | 36.72 (-16.43 to 149.87) | 0 (0 to 0) |
| Central African Republic | <5 years | Both sexes | NA | NA | 0 (0 to 0) |
| Central African Republic | <5 years | Males | NA | NA | 0 (0 to 0) |
| Central African Republic | <5 years | Females | NA | NA | 0 (0 to 0) |
| Central African Republic | 5-9 years | Both sexes | NA | NA | 11.81 (3.33 to 32.15) |
| Central African Republic | 5-9 years | Males | NA | NA | 15.35 (4.14 to 39.73) |
| Central African Republic | 5-9 years | Females | NA | NA | 8.16 (1.84 to 26.26) |
| Central African Republic | 10-14 years | Both sexes | NA | NA | 157.03 (64.67 to 310.81) |
| Central African Republic | 10-14 years | Males | NA | NA | 191.4 (78.61 to 384.59) |
| Central African Republic | 10-14 years | Females | NA | NA | 122.11 (44.96 to 265.79) |
| Central African Republic | 15-19 years | Both sexes | 119.84 (-13.87 to 395.47) | 99.86 (-38.18 to 392.15) | 146.31 (54.56 to 298.25) |
| Central African Republic | 15-19 years | Males | NA | 103.03 (-34.5 to 408.28) | 187.47 (73.53 to 373.05) |
| Central African Republic | 15-19 years | Females | 237.93 (-27.54 to 785.19) | 96.74 (-39.81 to 395.44) | 105.75 (35.59 to 229.29) |
| Central African Republic | 20-24 years | Both sexes | 169.6 (-25.38 to 438.49) | 93.68 (-25.24 to 356.08) | 99.75 (35.45 to 208.11) |
| Central African Republic | 20-24 years | Males | NA | 90.17 (-24.76 to 330.74) | 130.04 (49.42 to 271.54) |
| Central African Republic | 20-24 years | Females | 327.95 (-49.08 to 847.91) | 96.96 (-25.84 to 382.52) | 71.46 (19.4 to 172.86) |
| Central African Republic | 25-29 years | Both sexes | 176.76 (-28.6 to 444.66) | 86.39 (-26.21 to 340.12) | 66.41 (17.81 to 135.51) |
| Central African Republic | 25-29 years | Males | NA | 81.02 (-24.5 to 298.42) | 92.33 (28.87 to 190.76) |
| Central African Republic | 25-29 years | Females | 331.56 (-53.65 to 834.07) | 91.09 (-27.7 to 371.25) | 43.71 (5.66 to 103.52) |
| Central African Republic | 30-34 years | Both sexes | 171.58 (-29.74 to 437.65) | 84.75 (-27.55 to 341.83) | 42.33 (8.2 to 101.59) |
| Central African Republic | 30-34 years | Males | NA | 81.35 (-26.54 to 314.55) | 65.12 (13.94 to 146.33) |
| Central African Republic | 30-34 years | Females | 319.65 (-55.41 to 815.33) | 87.68 (-28.57 to 362.69) | 22.67 (0 to 65.18) |
| Central African Republic | 35-39 years | Both sexes | 155.96 (-32.95 to 430.61) | 80.29 (-27.38 to 327.81) | 21.76 (0.09 to 52.17) |
| Central African Republic | 35-39 years | Males | NA | 78.7 (-26.9 to 305.55) | 42.01 (0.15 to 103.79) |
| Central African Republic | 35-39 years | Females | 295.77 (-62.48 to 816.61) | 81.72 (-28.39 to 339.3) | 3.6 (0 to 14.98) |
| Central African Republic | 40-44 years | Both sexes | 142.53 (-26.82 to 377.62) | 80.3 (-28.3 to 285.06) | 10.27 (0 to 35.2) |
| Central African Republic | 40-44 years | Males | NA | 79.84 (-27.23 to 277.34) | 21.02 (0 to 72.05) |
| Central African Republic | 40-44 years | Females | 278.63 (-52.44 to 738.22) | 80.74 (-29.37 to 299.49) | 0 (0 to 0) |
| Central African Republic | 45-49 years | Both sexes | 128.53 (-21.2 to 381.39) | 79.97 (-29.35 to 296.4) | 0.65 (0 to 3.93) |
| Central African Republic | 45-49 years | Males | NA | 79.77 (-31.06 to 289.44) | 1.25 (0 to 7.63) |
| Central African Republic | 45-49 years | Females | 265.16 (-43.74 to 786.8) | 80.19 (-28.6 to 303.22) | 0 (0 to 0) |
| Central African Republic | 50-54 years | Both sexes | 127.01 (-22.27 to 387.6) | 81.11 (-31.54 to 326.16) | 0 (0 to 0) |
| Central African Republic | 50-54 years | Males | NA | 81.28 (-31.61 to 326.98) | 0 (0 to 0) |
| Central African Republic | 50-54 years | Females | 273.85 (-48.02 to 835.71) | 80.93 (-31.16 to 332.68) | 0 (0 to 0) |
| Central African Republic | 55-59 years | Both sexes | 123.33 (-21.52 to 371.21) | 79.96 (-34.54 to 325.76) | 0 (0 to 0) |
| Central African Republic | 55-59 years | Males | NA | 80.47 (-34.33 to 305.77) | 0 (0 to 0) |
| Central African Republic | 55-59 years | Females | 260.4 (-45.43 to 783.74) | 79.39 (-33.09 to 329.51) | 0 (0 to 0) |
| Central African Republic | 60-64 years | Both sexes | 106.33 (-16.7 to 323.01) | 78.61 (-33.83 to 318.89) | 0 (0 to 0) |
| Central African Republic | 60-64 years | Males | NA | 79.84 (-34.64 to 305.89) | 0 (0 to 0) |
| Central African Republic | 60-64 years | Females | 211.26 (-33.19 to 641.75) | 77.41 (-33.04 to 332.99) | 0 (0 to 0) |
| Central African Republic | 65-69 years | Both sexes | 91.6 (-11.9 to 317.99) | 76.12 (-29.14 to 315.04) | 0 (0 to 0) |
| Central African Republic | 65-69 years | Males | NA | 77.6 (-31.06 to 321.59) | 0 (0 to 0) |
| Central African Republic | 65-69 years | Females | 169.75 (-22.04 to 589.3) | 74.86 (-27.51 to 311.36) | 0 (0 to 0) |
| Central African Republic | 70-74 years | Both sexes | 88.65 (-9.51 to 302.29) | 64.41 (-18.95 to 289.35) | 0 (0 to 0) |
| Central African Republic | 70-74 years | Males | NA | 67.18 (-20.75 to 275.02) | 0 (0 to 0) |
| Central African Republic | 70-74 years | Females | 156.07 (-16.75 to 532.14) | 62.31 (-18.64 to 285.77) | 0 (0 to 0) |
| Central African Republic | 75-79 years | Both sexes | 73.74 (-7.88 to 254.77) | 57.29 (-16.05 to 239.75) | 0 (0 to 0) |
| Central African Republic | 75-79 years | Males | NA | 60.41 (-18.1 to 247.17) | 0 (0 to 0) |
| Central African Republic | 75-79 years | Females | 124.41 (-13.29 to 429.86) | 55.14 (-15.2 to 230.65) | 0 (0 to 0) |
| Central African Republic | 80-84 years | Both sexes | 54.88 (-4.91 to 209.85) | 41.35 (-11.21 to 166.59) | 0 (0 to 0) |
| Central African Republic | 80-84 years | Males | NA | 44.84 (-12.19 to 177.61) | 0 (0 to 0) |
| Central African Republic | 80-84 years | Females | 88 (-7.87 to 336.52) | 39.25 (-10.34 to 162.76) | 0 (0 to 0) |
| Central African Republic | 85-89 years | Both sexes | 54.51 (-5.35 to 206.57) | 36.83 (-10.8 to 158.9) | 0 (0 to 0) |
| Central African Republic | 85-89 years | Males | NA | 40.37 (-12.01 to 169.82) | 0 (0 to 0) |
| Central African Republic | 85-89 years | Females | 86.37 (-8.47 to 327.29) | 34.76 (-9.77 to 153.14) | 0 (0 to 0) |
| Central African Republic | 90-94 years | Both sexes | 53.6 (-5.67 to 202.97) | 33.93 (-10.21 to 152.85) | 0 (0 to 0) |
| Central African Republic | 90-94 years | Males | NA | 37.65 (-11.74 to 168.29) | 0 (0 to 0) |
| Central African Republic | 90-94 years | Females | 85.67 (-9.06 to 324.45) | 31.7 (-9.34 to 144.01) | 0 (0 to 0) |
| Central African Republic | 95+ years | Both sexes | 53.09 (-5.13 to 209.99) | 33.44 (-11.43 to 139.42) | 0 (0 to 0) |
| Central African Republic | 95+ years | Males | NA | 37.21 (-13.03 to 155.57) | 0 (0 to 0) |
| Central African Republic | 95+ years | Females | 87.7 (-8.48 to 346.85) | 30.99 (-10.34 to 128.9) | 0 (0 to 0) |
| Chad | <5 years | Both sexes | NA | NA | 0 (0 to 0) |
| Chad | <5 years | Males | NA | NA | 0 (0 to 0) |
| Chad | <5 years | Females | NA | NA | 0 (0 to 0) |
| Chad | 5-9 years | Both sexes | NA | NA | 11.68 (3.39 to 30.58) |
| Chad | 5-9 years | Males | NA | NA | 14.17 (3.81 to 37.48) |
| Chad | 5-9 years | Females | NA | NA | 9.14 (2.29 to 24.43) |
| Chad | 10-14 years | Both sexes | NA | NA | 209 (72.75 to 463.96) |
| Chad | 10-14 years | Males | NA | NA | 208.01 (72.33 to 452.87) |
| Chad | 10-14 years | Females | NA | NA | 210 (64.79 to 495.82) |
| Chad | 15-19 years | Both sexes | 113.83 (-15.25 to 386.6) | 161.99 (-69.08 to 586.03) | 161.81 (63.84 to 339.65) |
| Chad | 15-19 years | Males | NA | 117.06 (-44.82 to 432.37) | 172.7 (64.66 to 351.14) |
| Chad | 15-19 years | Females | 224.84 (-30.13 to 763.61) | 205.81 (-90.61 to 736.43) | 151.18 (47.43 to 339.27) |
| Chad | 20-24 years | Both sexes | 196.31 (-35.67 to 500.22) | 131.72 (-44.7 to 477.13) | 104.01 (37.18 to 227.09) |
| Chad | 20-24 years | Males | NA | 95.99 (-30.88 to 341.95) | 118.42 (45.94 to 246.16) |
| Chad | 20-24 years | Females | 379.31 (-68.92 to 966.52) | 165.02 (-58.54 to 605.22) | 90.57 (23.62 to 223.64) |
| Chad | 25-29 years | Both sexes | 194.76 (-40.77 to 486.29) | 114.79 (-40.22 to 427.73) | 66.93 (19.74 to 145.97) |
| Chad | 25-29 years | Males | NA | 86.43 (-28.02 to 309.94) | 84.36 (29.98 to 165.13) |
| Chad | 25-29 years | Females | 369.47 (-77.35 to 922.54) | 140.22 (-51.16 to 538.91) | 51.3 (7.32 to 138.28) |
| Chad | 30-34 years | Both sexes | 187.76 (-45.18 to 451.88) | 104.81 (-41.24 to 382.28) | 38.12 (7.72 to 89.69) |
| Chad | 30-34 years | Males | NA | 85.11 (-33.65 to 297.37) | 56.48 (12.39 to 122.11) |
| Chad | 30-34 years | Females | 351.48 (-84.59 to 845.92) | 121.99 (-48.16 to 455.02) | 22.11 (0 to 67.07) |
| Chad | 35-39 years | Both sexes | 175.61 (-42.59 to 393.97) | 94.2 (-38.06 to 351.42) | 17.17 (0.06 to 43.73) |
| Chad | 35-39 years | Males | NA | 81.92 (-31.98 to 296.27) | 33.79 (0.12 to 82.58) |
| Chad | 35-39 years | Females | 326.93 (-79.29 to 733.43) | 104.79 (-43.22 to 378.89) | 2.84 (0 to 12.17) |
| Chad | 40-44 years | Both sexes | 137.58 (-25.8 to 332.87) | 90.78 (-35.68 to 302) | 7.4 (0 to 26.31) |
| Chad | 40-44 years | Males | NA | 80.76 (-32.52 to 261.9) | 15.54 (0 to 55.24) |
| Chad | 40-44 years | Females | 262.7 (-49.27 to 635.59) | 99.88 (-39.39 to 338.47) | 0 (0 to 0) |
| Chad | 45-49 years | Both sexes | 149.45 (-32.01 to 373.15) | 88.6 (-35.73 to 297.63) | 0.51 (0 to 3.24) |
| Chad | 45-49 years | Males | NA | 80.65 (-32.8 to 265.57) | 1.03 (0 to 6.56) |
| Chad | 45-49 years | Females | 295.49 (-63.3 to 737.79) | 96.37 (-37.67 to 332.21) | 0 (0 to 0) |
| Chad | 50-54 years | Both sexes | 124.53 (-20.41 to 352.29) | 88.36 (-39.87 to 326.57) | 0 (0 to 0) |
| Chad | 50-54 years | Males | NA | 80.31 (-35.21 to 289.21) | 0 (0 to 0) |
| Chad | 50-54 years | Females | 256.85 (-42.1 to 726.64) | 96.91 (-42.02 to 370.79) | 0 (0 to 0) |
| Chad | 55-59 years | Both sexes | 110.27 (-17.84 to 313.9) | 86.1 (-39.86 to 315.2) | 0 (0 to 0) |
| Chad | 55-59 years | Males | NA | 79.21 (-36.53 to 289.49) | 0 (0 to 0) |
| Chad | 55-59 years | Females | 239.63 (-38.76 to 682.15) | 94.19 (-43.93 to 360.48) | 0 (0 to 0) |
| Chad | 60-64 years | Both sexes | 86.17 (-11.9 to 259.43) | 84.59 (-41.08 to 310.03) | 0 (0 to 0) |
| Chad | 60-64 years | Males | NA | 79.01 (-40.1 to 281.76) | 0 (0 to 0) |
| Chad | 60-64 years | Females | 192.98 (-26.65 to 581) | 91.52 (-42.29 to 346.78) | 0 (0 to 0) |
| Chad | 65-69 years | Both sexes | 67.2 (-7.9 to 216.22) | 81.75 (-35.03 to 312.66) | 0 (0 to 0) |
| Chad | 65-69 years | Males | NA | 76.98 (-32.19 to 292.78) | 0 (0 to 0) |
| Chad | 65-69 years | Females | 153.86 (-18.08 to 495.07) | 87.9 (-38.7 to 352.25) | 0 (0 to 0) |
| Chad | 70-74 years | Both sexes | 61.78 (-6.98 to 222.01) | 70.75 (-22.12 to 300.47) | 0 (0 to 0) |
| Chad | 70-74 years | Males | NA | 66.88 (-21.54 to 275.14) | 0 (0 to 0) |
| Chad | 70-74 years | Females | 141.71 (-16.02 to 509.26) | 75.76 (-24.22 to 331.8) | 0 (0 to 0) |
| Chad | 75-79 years | Both sexes | 50.53 (-4.93 to 178.22) | 63.41 (-18.96 to 255.22) | 0 (0 to 0) |
| Chad | 75-79 years | Males | NA | 60.07 (-17.91 to 235.55) | 0 (0 to 0) |
| Chad | 75-79 years | Females | 112.36 (-10.96 to 396.29) | 67.5 (-20.48 to 271.61) | 0 (0 to 0) |
| Chad | 80-84 years | Both sexes | 38.33 (-3.9 to 150.73) | 47.27 (-13.7 to 186.73) | 0 (0 to 0) |
| Chad | 80-84 years | Males | NA | 44.74 (-12.78 to 174.97) | 0 (0 to 0) |
| Chad | 80-84 years | Females | 80.54 (-8.2 to 316.74) | 50.06 (-14.78 to 205.88) | 0 (0 to 0) |
| Chad | 85-89 years | Both sexes | 39.69 (-4.03 to 144.25) | 42.87 (-12.83 to 179.41) | 0 (0 to 0) |
| Chad | 85-89 years | Males | NA | 41.04 (-12.31 to 168.61) | 0 (0 to 0) |
| Chad | 85-89 years | Females | 78.4 (-7.97 to 284.98) | 44.65 (-13.01 to 184.91) | 0 (0 to 0) |
| Chad | 90-94 years | Both sexes | 41.98 (-4.65 to 158.59) | 39.83 (-12.82 to 167.96) | 0 (0 to 0) |
| Chad | 90-94 years | Males | NA | 37.95 (-13.07 to 162.31) | 0 (0 to 0) |
| Chad | 90-94 years | Females | 77.9 (-8.63 to 294.27) | 41.43 (-12.7 to 172.8) | 0 (0 to 0) |
| Chad | 95+ years | Both sexes | 45.83 (-4.96 to 174.53) | 39.33 (-13.75 to 161.48) | 0 (0 to 0) |
| Chad | 95+ years | Males | NA | 37.6 (-13.81 to 143.53) | 0 (0 to 0) |
| Chad | 95+ years | Females | 79.13 (-8.57 to 301.34) | 40.59 (-14.23 to 174.25) | 0 (0 to 0) |
| Chile | <5 years | Both sexes | NA | NA | 0 (0 to 0) |
| Chile | <5 years | Males | NA | NA | 0 (0 to 0) |
| Chile | <5 years | Females | NA | NA | 0 (0 to 0) |
| Chile | 5-9 years | Both sexes | NA | NA | 37.38 (9.21 to 111.7) |
| Chile | 5-9 years | Males | NA | NA | 35.71 (9.03 to 98.68) |
| Chile | 5-9 years | Females | NA | NA | 39.12 (8.51 to 126.5) |
| Chile | 10-14 years | Both sexes | NA | NA | 197.16 (64.94 to 420.12) |
| Chile | 10-14 years | Males | NA | NA | 194.95 (66.22 to 398.73) |
| Chile | 10-14 years | Females | NA | NA | 199.49 (60.91 to 458.34) |
| Chile | 15-19 years | Both sexes | 91.89 (-13 to 334.28) | 171.37 (-75.68 to 537.95) | 207.19 (80.95 to 404.82) |
| Chile | 15-19 years | Males | NA | 71.64 (-30.77 to 283.43) | 205.71 (84.45 to 388.97) |
| Chile | 15-19 years | Females | 189 (-26.73 to 687.59) | 276.78 (-121.69 to 819.32) | 208.75 (70.32 to 437.02) |
| Chile | 20-24 years | Both sexes | 160.64 (-23.96 to 467.02) | 159.64 (-63.73 to 524.71) | 205.74 (79.92 to 434.91) |
| Chile | 20-24 years | Males | NA | 66.28 (-21.89 to 272.68) | 203.46 (88.17 to 405.48) |
| Chile | 20-24 years | Females | 331.9 (-49.49 to 964.91) | 259.18 (-110.88 to 805.18) | 208.17 (63.38 to 462.56) |
| Chile | 25-29 years | Both sexes | 196.94 (-32.48 to 498.8) | 159.83 (-64.47 to 563.43) | 172.71 (57.34 to 360.75) |
| Chile | 25-29 years | Males | NA | 66.56 (-19.79 to 279.85) | 179.06 (64.61 to 336.06) |
| Chile | 25-29 years | Females | 404.59 (-66.73 to 1024.72) | 258.17 (-110.32 to 830.66) | 166.01 (23.42 to 395.26) |
| Chile | 30-34 years | Both sexes | 203.15 (-37.26 to 518.45) | 171.64 (-75.6 to 548.93) | 130.9 (25.71 to 289.76) |
| Chile | 30-34 years | Males | NA | 83.24 (-28.36 to 302.38) | 148.25 (38.45 to 307.71) |
| Chile | 30-34 years | Females | 416.35 (-76.36 to 1062.55) | 264.41 (-127.13 to 784.71) | 112.7 (0 to 281.94) |
| Chile | 35-39 years | Both sexes | 197.17 (-37.79 to 485.44) | 163.22 (-69.16 to 534.54) | 69.84 (0.83 to 165.63) |
| Chile | 35-39 years | Males | NA | 76.07 (-26.01 to 277.25) | 108.7 (0.22 to 234.71) |
| Chile | 35-39 years | Females | 396.98 (-76.09 to 977.37) | 251.54 (-117.74 to 784.51) | 30.47 (0 to 106.91) |
| Chile | 40-44 years | Both sexes | 188.64 (-31.79 to 492.6) | 164.09 (-73.22 to 495.05) | 27.32 (0 to 83.69) |
| Chile | 40-44 years | Males | NA | 86.01 (-36.18 to 273) | 55.05 (0 to 168.62) |
| Chile | 40-44 years | Females | 374.5 (-63.11 to 977.94) | 241.03 (-111.04 to 717.2) | 0 (0 to 0) |
| Chile | 45-49 years | Both sexes | 176.79 (-26.22 to 449.7) | 153.66 (-69.87 to 474.81) | 1.5 (0 to 11.63) |
| Chile | 45-49 years | Males | NA | 77.73 (-34.55 to 260.21) | 3.07 (0 to 23.82) |
| Chile | 45-49 years | Females | 345.59 (-51.25 to 879.07) | 226.16 (-103.69 to 696.13) | 0 (0 to 0) |
| Chile | 50-54 years | Both sexes | 148.21 (-18.06 to 414.95) | 146.34 (-65.88 to 508.93) | 0 (0 to 0) |
| Chile | 50-54 years | Males | NA | 70.8 (-32.14 to 252.88) | 0 (0 to 0) |
| Chile | 50-54 years | Females | 286.4 (-34.9 to 801.85) | 216.78 (-100.16 to 731.71) | 0 (0 to 0) |
| Chile | 55-59 years | Both sexes | 126.38 (-17.17 to 371.97) | 142.45 (-69.52 to 484.87) | 0 (0 to 0) |
| Chile | 55-59 years | Males | NA | 76.02 (-37.64 to 257.23) | 0 (0 to 0) |
| Chile | 55-59 years | Females | 240.88 (-32.73 to 708.99) | 202.64 (-106.12 to 704.03) | 0 (0 to 0) |
| Chile | 60-64 years | Both sexes | 106.49 (-16.48 to 340.78) | 130.21 (-61.53 to 475.06) | 0 (0 to 0) |
| Chile | 60-64 years | Males | NA | 62.35 (-26.15 to 252.49) | 0 (0 to 0) |
| Chile | 60-64 years | Females | 200.24 (-30.98 to 640.78) | 189.95 (-98.35 to 681.17) | 0 (0 to 0) |
| Chile | 65-69 years | Both sexes | 135.03 (-21.55 to 386.65) | 120.76 (-57.55 to 439.59) | 0 (0 to 0) |
| Chile | 65-69 years | Males | NA | 57.41 (-23.31 to 228.79) | 0 (0 to 0) |
| Chile | 65-69 years | Females | 251.15 (-40.09 to 719.15) | 175.24 (-86.24 to 615.78) | 0 (0 to 0) |
| Chile | 70-74 years | Both sexes | 129.38 (-19.91 to 346.98) | 103.32 (-39.14 to 395.93) | 0 (0 to 0) |
| Chile | 70-74 years | Males | NA | 46.85 (-14.44 to 206.88) | 0 (0 to 0) |
| Chile | 70-74 years | Females | 235.78 (-36.28 to 632.36) | 149.76 (-58.86 to 551.44) | 0 (0 to 0) |
| Chile | 75-79 years | Both sexes | 100.15 (-13.88 to 312.97) | 91.05 (-33.27 to 327.63) | 0 (0 to 0) |
| Chile | 75-79 years | Males | NA | 40.59 (-11.29 to 163.14) | 0 (0 to 0) |
| Chile | 75-79 years | Females | 176.97 (-24.53 to 553.02) | 129.76 (-49.24 to 451.47) | 0 (0 to 0) |
| Chile | 80-84 years | Both sexes | 95.08 (-15.05 to 296.13) | 68.84 (-24.48 to 257.7) | 0 (0 to 0) |
| Chile | 80-84 years | Males | NA | 28.4 (-7.07 to 115.47) | 0 (0 to 0) |
| Chile | 80-84 years | Females | 161.14 (-25.5 to 501.87) | 96.93 (-36.83 to 357.93) | 0 (0 to 0) |
| Chile | 85-89 years | Both sexes | 96.6 (-14.45 to 299.48) | 62.85 (-20.44 to 248.77) | 0 (0 to 0) |
| Chile | 85-89 years | Males | NA | 25.05 (-6.86 to 99.85) | 0 (0 to 0) |
| Chile | 85-89 years | Females | 154.9 (-23.18 to 480.25) | 85.67 (-29.14 to 335.58) | 0 (0 to 0) |
| Chile | 90-94 years | Both sexes | 100.88 (-15.44 to 306.68) | 59.66 (-20.45 to 226.97) | 0 (0 to 0) |
| Chile | 90-94 years | Males | NA | 23.08 (-7.22 to 97.11) | 0 (0 to 0) |
| Chile | 90-94 years | Females | 149.23 (-22.84 to 453.65) | 77.2 (-26.64 to 284.86) | 0 (0 to 0) |
| Chile | 95+ years | Both sexes | 103.91 (-16.12 to 318.38) | 58.27 (-22.1 to 220.84) | 0 (0 to 0) |
| Chile | 95+ years | Males | NA | 22.52 (-7.33 to 86.86) | 0 (0 to 0) |
| Chile | 95+ years | Females | 148.2 (-22.98 to 454.09) | 73.5 (-27.75 to 280.83) | 0 (0 to 0) |
| China | <5 years | Both sexes | NA | NA | 0 (0 to 0) |
| China | <5 years | Males | NA | NA | 0 (0 to 0) |
| China | <5 years | Females | NA | NA | 0 (0 to 0) |
| China | 5-9 years | Both sexes | NA | NA | 7 (2.12 to 16.56) |
| China | 5-9 years | Males | NA | NA | 8.24 (2.66 to 19.94) |
| China | 5-9 years | Females | NA | NA | 5.61 (1.52 to 13.83) |
| China | 10-14 years | Both sexes | NA | NA | 146.65 (50.18 to 393.09) |
| China | 10-14 years | Males | NA | NA | 169.05 (56.83 to 434.26) |
| China | 10-14 years | Females | NA | NA | 121.25 (37.44 to 296.61) |
| China | 15-19 years | Both sexes | 32.91 (-3.17 to 119.97) | 57.05 (-21.5 to 204.07) | 80.79 (32.24 to 166.29) |
| China | 15-19 years | Males | NA | 48.08 (-18.64 to 169.88) | 93.61 (38.75 to 185.4) |
| China | 15-19 years | Females | 70.75 (-6.81 to 257.95) | 67.36 (-25.35 to 239.24) | 66.05 (23.31 to 146.2) |
| China | 20-24 years | Both sexes | 33.15 (-3.49 to 103.57) | 35.12 (-11.7 to 122.41) | 50.59 (18.89 to 107.9) |
| China | 20-24 years | Males | NA | 30.04 (-12.14 to 101.65) | 58.56 (23.28 to 117.96) |
| China | 20-24 years | Females | 71.19 (-7.5 to 222.43) | 40.95 (-12.06 to 156.54) | 41.45 (12.17 to 96.6) |
| China | 25-29 years | Both sexes | 55.99 (-8.66 to 142.97) | 34.02 (-10.28 to 107.55) | 36.79 (10.98 to 75.27) |
| China | 25-29 years | Males | NA | 27.93 (-8.66 to 89.61) | 43.64 (14.54 to 84.39) |
| China | 25-29 years | Females | 119.87 (-18.54 to 306.1) | 40.97 (-9.97 to 152.93) | 28.98 (3.89 to 68.92) |
| China | 30-34 years | Both sexes | 64.52 (-11.4 to 160.04) | 40.31 (-13.36 to 124.57) | 25.84 (5.29 to 58.85) |
| China | 30-34 years | Males | NA | 31.44 (-9.76 to 97.79) | 32.42 (7.2 to 68.74) |
| China | 30-34 years | Females | 135.91 (-24.01 to 337.15) | 50.12 (-13.94 to 168.27) | 18.56 (0 to 49.18) |
| China | 35-39 years | Both sexes | 71.69 (-12.91 to 170.29) | 46.72 (-18.86 to 142.99) | 14 (0.14 to 36.61) |
| China | 35-39 years | Males | NA | 33.81 (-12.2 to 102.54) | 22.66 (0.28 to 54.1) |
[truncated: 1,032,147 more chars]
